# Regioselective molybdenum-catalyzed allylic substitution of tertiary allylic electrophiles: methodology development and applications†

**DOI:** 10.1039/d0sc01763a

**Published:** 2020-05-06

**Authors:** Muhammad Salman, Yaoyao Xu, Shahid Khan, Junjie Zhang, Ajmal Khan

**Affiliations:** Department of Applied Chemistry, School of Science, Xi'an Key Laboratory of Sustainable Energy Materials Chemistry, Xi'an Jiao Tong University Xi'an 710049 P. R. China ajmalkhan@xjtu.edu.cn

## Abstract

The first molybdenum-catalyzed allylic sulfonylation of tertiary allylic electrophiles is described. The method employs a readily accessible catalyst (Mo(CO)_6_/2,2′-bipyridine, both are commercially available) and represents the first example of the use of a group 6 transition metal-catalyst for allylic sulfonylation of substituted tertiary allylic electrophiles to form carbon–sulfur bonds. This atom economic and operationally simple methodology is characterized by its relatively mild conditions, wide substrate scope, and excellent regioselectivity profile, thus unlocking a new platform to forge sulfone moieties, even in the context of late-stage functionalization and providing ample opportunities for further derivatization through traditional Suzuki cross-coupling reactions.

## Abstract 2

The first general example of Mo-catalyzed allylic sulfonylation of tertiary allylic electrophile provides an efficient way to forge sulfone moieties, and providing ample opportunities for further transformation through traditional Suzuki cross-coupling.

## Graphical abstract

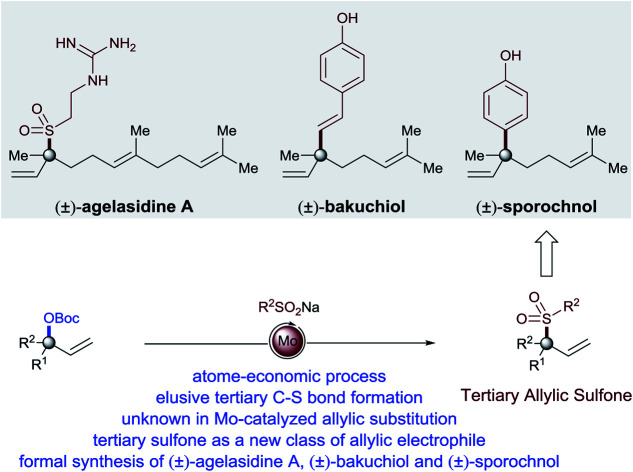

## Introduction

**Fig. 1:**
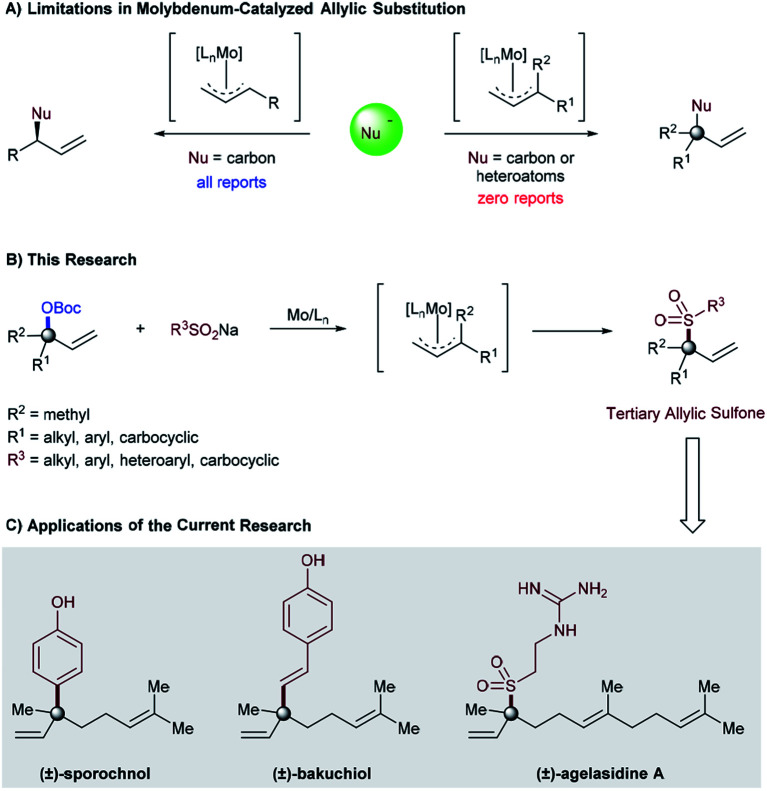
(A) Limitations in molybdenum-catalyzed allylic substitution, (B) our research, and (C) applications of the current research.

The concept of the π-allyl metal-complex was first formulated by Tsuji in 1965 (ref. 1*a*) and, later, properly adopted by Trost in 1973.^1*b*^ Since then, this technology has enabled organic chemists to create a host of novel procedures for the synthesis of simple to complex molecules.^2^ Among these is the development and utilization of heteroatom nucleophile reagents, such as oxygen, nitrogen, and sulfur-based nucleophiles.^2,3^ Despite the massive development that has been made in this area, there still remain untapped opportunities in the potential application of these heteroatom nucleophile reagents in transition metal-catalyzed allylic substitution. For example, molybdenum-catalyzed allylic substitution reactions of heteroatom nucleophiles are unknown and largely limited only to carbon–carbon bond formation procedures (Fig. 1A, left).^4^ Furthermore, the substrate scope with respect to the allylic electrophile has also remained unchanged and restricted to the ones that provide products containing a tertiary center at the allylic position.^5^ Regardless of the longstanding interest in the formation of carbon–heteroatom bonds within the synthetic organic community, as well as the advancement of other transition-metal-catalyzed reactions to provide heteroatom bearing quaternary and/or tertiary allylic centers,^6^ molybdenum-catalyzed allylic substitution reactions that provide products containing such a stereocenter remain prominently absent from the literature and yet to be discovered (Fig. 1A, right).^7^

Due to the high importance of allylic sulfones as pharmaceuticals^8^ and synthetic candidates,^9^ organic chemists have recently been designing catalytic C–S bond cleavage procedures as a new tool for carbon–carbon bond formation through Suzuki cross-coupling^10^ and/or allylic substitution reactions.^11^ Despite the considerable development realized in this area, allylic sulfone formation is still a challenging task and confined to the use of transition metal-catalyzed allylic sulfonylation procedures.^12,13^ However, using these procedures for the synthesis of allylic sulfones containing tetrasubstituted carbon centers is limited and largely unexplored.^14^ Therefore, at the beginning of our study it was unclear whether a molybdenum-catalyzed allylic substitution could ever be implemented with a heteroatom (sodium sulfinate) nucleophile or even with α,α-disubstituted allylic precursors. If successful, such unexplored areas of allylic substitution chemistry might not only provide an opportunity to realize currently inaccessible chemical space (carbon–heteroatom bond formation) in molybdenum-catalyzed allylic substitution, but also provide a new synthetic approach for rapidly generating quaternary all-carbon centers through Suzuki cross-coupling of the sulfone functionality. As part of our ongoing program in developing molybdenum-catalyzed allylic substitution technology and our continued interest in the catalytic asymmetric synthesis of quaternary stereocenters,^14*a*,15^ we were attracted to this unmet challenge and report herein the successful implementation of this idea (Fig. 1B). The salient features of this process are the atom-economic procedures, high regioselectivity, and excellent functional group tolerance for both sulfinate salt and tertiary allylic carbonates, even in the context of late-stage functionalization. Furthermore, the high reactivity of tertiary allylic sulfones as a new class of electrophiles to yield structurally diverse products containing quaternary all-carbon centers through Suzuki cross-coupling is a special characteristic feature of this catalytic system (Fig. 1C).^10*a*^

## Results and discussion

Our optimization began by evaluating the allylic substitution of tertiary allylic carbonate **1a**, readily prepared from the corresponding alcohol on a large scale, with sodium benzenesulfinate **2a** (Table 1). Interestingly, a disappointing amount of either **3aa** or **4aa** was detected under reaction conditions previously reported for other molybdenum-catalyzed allylic substitution reactions.^4^ After several experiments,^16^ we concluded that a combination of the inexpensive commercially available Mo(CO)_6_ precursor and 2,2′-bipyridine as a ligand (**L1**)^17^ in EtOH at 60 °C afforded **3aa** in 92% yield upon isolation with excellent branched to linear selectivity (**3aa**/**4aa** = >99 : 1). Amongst all of the ligands utilized, 2,2′-bipyridine motifs were crucial for achieving the targeted transformation. While excellent reactivity towards **3aa** was found with 2,2′-bipyridines and 6,6′-dimethyl-2,2′-bipyridine, better yields were obtained for the first one (entries 1–7). Interestingly, the bench-stable terpyridine **L7** failed to provide product **3aa**. These results indicate that the coordination geometry of the ligand dictates the reactivity, with 2,2′-bipyridine ligands being particularly suited for the high yield and selectivity of **3aa**. Subtle changes in the molybdenum precursor and/or solvent, however, had a negative influence on the reaction, consistently providing lower yields if any (entries 8–14). As anticipated, control experiments revealed that all of the reaction parameters were necessary for the reaction to occur (entry 15).

**Table 1:** Optimization of the reaction parameters^a^

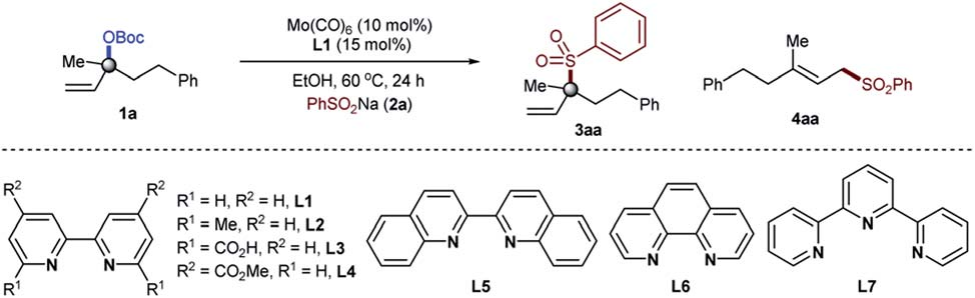			
Entry	Deviation in conditions	**3aa**/**4aa**^b^	**3aa** c (%)
1	None	99 : 1	92
2	**L2** was used instead of **L1**	99 : 1	87
3	**L3** was used instead of **L1**	99 : 1	35
4	**L4** was used instead of **L1**	99 : 1	52
5	**L5** was used instead of **L1**	25 : 1	16
6	**L6** was used instead of **L1**	—	0
7	**L7** was used instead of **L1**	—	>5
8	(C_7_H_8_)_3_Mo(CO)_3_ was used	99 : 1	82
9	THF was used as solvent	—	>5
10	Toluene was used as solvent	—	>5
11	DCE was used as solvent	25 : 1	35
12	^i^PrOH was used as solvent	99 : 1	77
13	THF/EtOH (5 : 1) as solvent	25 : 1	25
14	DCE/EtOH (5 : 1) as solvent	25 : 1	63
15	Without Mo or **L1**	—	0

a Reaction conditions: Mo-catalyst (10 mol%), ligand (15 mol%), **1a** (0.2 mmol), PhSO_2_Na **2a** (0.3 mmol), solvent (1.0 mL, 0.2 M), 60 °C, 24 hours.

b Determined by ^1^H-NMR of the crude reaction mixture.

c Isolated yields.

With reliable access to **3aa**, we next turned our attention to examine the generality of our newly developed molybdenum-catalyzed regioselective sulfonylation of tertiary allylic electrophiles with sodium sulfinate by using the Mo/**L1** catalyst system as shown in Table 2. In all cases analysed for sulfinate salts (**2**), excellent reactivity and selectivity was observed. Both the electron-withdrawing and electron-donating substituents on the aromatic ring of the sulfinate salts react smoothly with **1a**, affording the corresponding α,α-disubstituted allylic products in high yields (**3aa–3ap**). Sodium sulfinates with bulky naphthyl (**3aq**), quinoline (**3ar**), 2,3-dihydrobenzofuran (**3as**), and heteroaryl (**3at**, **3au**) moieties were also tolerated under the current optimized conditions. Likewise, the targeted tertiary allylic sulfone formation could be extended to sulfinate salts with alkyl substituents. Both primary and secondary alkyl substituted sodium sulfinates worked well to provide α,α-disubstituted allylic sulfones in high yields (**3av–3ay**). Furthermore, a more functionalized sodium sulfinate **2z**, when used as the sulfonylation partner, the branched product **3az** was obtained in 72% of isolated yield. The reaction leading to tertiary allylic sulfone **3aa** was easily scaled up to gram-scale without significant decrease in yield. Of particular note is that, almost in all cases, the reactions proceeded with excellent branched regioselectivity (>99 : 1).

**Table 2:** Sodium sulfinate substrate scope^a^^,^^b^^,^^c^

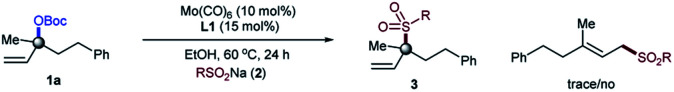			
Entry	**2**	**3** b	Yield^c^ (%)
1	**2a** (R = Ph)	**3aa**	92
2	**2b** (R = 4-MeC_6_H_4_)	**3ab**	93
3	**2c** (R = 4-MeOC_6_H_4_)	**3ac**	90
4	**2d** (R = 4-ClC_6_H_4_)	**3ad**	87
5	**2e** (R = 4-FC_6_H_4_)	**3ae**	85
6	**2f** (R = 4-NO_2_C_6_H_4_)	**3af**	75
7	**2g** (R = 4-CNC_6_H_4_)	**3ag**	72
8	**2h** (R = 2-FC_6_H_4_)	**3ah**	88
9	**2i** (R = 2-ClC_6_H_4_)	**3ai**	87
10	**2j** (R = 2-OCF_3_C_6_H_4_)	**3aj**	72
11	**2k** (R = 3-BrC_6_H_4_)	**3ak**	82
12	**2l** (R = 3-CNC_6_H_4_)	**3al**	78
13	**2m** (R = 2,4-MeOC_6_H_3_)	**3am**	94
14	**2n** (R = 3,5-CF_3_C_6_H_3_)	**3an**	95
15	**2o** (R = 2-MeO, 5-BrC_6_H_3_)	**3ao**	84
16	**2p** (R = 3,4-ClC_6_H_3_)	**3ap**	87
17	**2q** (R = 2-naphthyl)	**3aq**	82
18	**2r** (R = 1-quinoline)	**3ar**	78
19	**2s** (R = 2,3-dihydrobenzofuran)	**3as**	92
20	**2t** (R = 3-pyridine)	**3at**	82
21	**2u** (R = 2-thiophene)	**3au**	86
22	**2v** (R = Me)	**3av**	72
23	**2w** (R = Et)	**3aw**	78
24	**2x** (R = ^i^Pr)	**3ax**	82
25	**2y** (R = cyclopropyl)	**3ay**	78
26	**2z** (R = CH_3_OCOCH_2_CH_2_)	**3az**	72

a Reaction conditions: Mo(CO)_6_ (10 mol%), **L1** (15 mol%), **1a** (0.2 mmol), RSO_2_Na **2** (0.3 mmol), EtOH (1.0 mL, 0.2 M), 60 °C, 24 hours.

b Determined by ^1^H-NMR of the crude reaction mixture.

c Isolated yields.

We then focused on investigating the scope of the α,α-disubstituted allylic carbonates and the results obtained were compiled in Table 3. Tertiary allylic carbonate with simple propyl substituent (**1b**) reacted efficiently with sodium benzenesulfinate (**2a**) to deliver the branched allylic sulfone **3ba** in high yield (87%). However, allylic carbonate with a cyclohexyl moiety afforded the desired branched product in comparatively low yield (24%, **3ca**) due to the steric hindrance problem. However, tertiary allylic carbonate (**1d**) having a longer alkyl chain provided the desired product even at high yield (91%, **3da**). When tertiary allylic carbonates **1e**, **1f**, **1g** and **1h** with different groups on the alkyl chain were coupled with sulfinate salt **2a**, high yields of the branched allylic products were obtained (85–96%, **3ea**, **3fa**, **3ga** and **3ha**). Notably, various common functional groups such as Cl (**1i**), benzyl (**1j**), benzoyl (**1k**), thioether (**1l**), acetal (**1m**), and carbonate (**1n**) on the alkyl chain of the tertiary allylic carbonates were tolerated, and the sulfonylation branched products (**3ia–3na**) were isolated in high yields (82–94%). In addition, the unprotected hydroxy group on the alkyl chain of the tertiary allylic carbonates **1o** and **1p** do not interfere with productive tertiary allylic sulfone formation (**3oa** and **3pa**), thus providing opportunities for further derivatization. Notably, the reaction can be easily applied within the context of late-stage functionalization, supported by the formation of branched allylic sulfone **3qa**, derived from pentoxifylline. As expected, the allylic sulfonylation of phenyl substituted allylic carbonate occurred exclusively at the less-hindered position. The present optimized conditions were unsatisfactory with such substrates and provided the desired branched product (**3ra**) with a low branched to linear ratio (b/l = 1 : 5); indicating some (steric) limitation of the current protocol. Besides methyl-substituted tertiary allylic substrates **1a–1r**, other alkyl or aryl substituted substrates provide only starting materials when used under the optimized conditions, indicating some limitation of the present protocol.

**Table 3:** Allylic carbonate substrate scope^a^^,^^b^^,^^c^

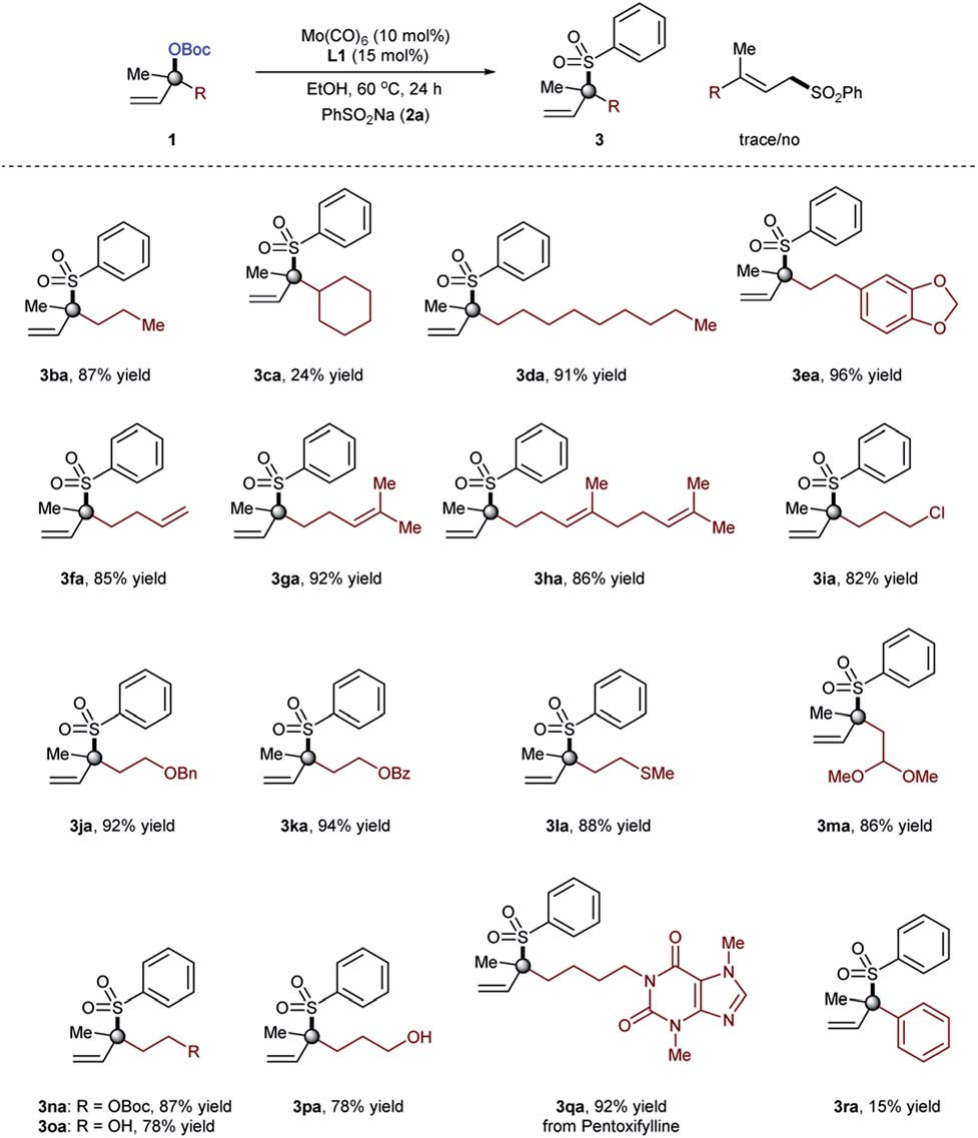

a Reaction conditions: Mo(CO)_6_ (10 mol%), **L1** (15 mol%), **1** (0.2 mmol), PhSO_2_Na **2a** (0.3 mmol), EtOH (1.0 mL, 0.2 M), 60 °C, 24 hours.

b Regioselectivity was determined by ^1^H-NMR of the crude reaction mixture.

c Isolated yields of the products.

In order to illustrate the synthetic utility of these elusive tertiary allylic sulfones, we focused on the reaction of α,α-disubstituted allylic carbonate (**1h**), and sodium sulfinate **2az**, to achieve the formal synthesis of (±)-agelasidine A.^18^ The desired tertiary allylic sulfone **3haz** was isolated in 84% yield under the standard conditions (Fig. 1A). This compound (**3haz**) can be readily converted to (±)-agelasidine A by following the literature procedure.^13*f*^ We further demonstrate that the current methodology can be utilized to prepare other related compounds containing sulfone-bearing quaternary carbon centers.^19^

Due to their ambiphilic nature, allylic sulfones are synthetically important electrophiles and have recently been utilized in Suzuki cross-coupling^10*a*^ as well as allylic substitution reactions.^11*d*^ However, selective cross-coupling of tertiary allylic sulfones remains highly challenging in Suzuki–Miyaura cross-coupling reactions.^10^ Indeed, we employed our tertiary allylic sulfone product **3ga** along with typical boronic acids as a coupling partner in order to achieve the formal synthesis of (±)-sporochnol,^20^ and (±)-bakuchiol,^21^ both of which are natural products possessing a quaternary all-carbon center. Our synthesis is illustrated in Fig. 1B. The key step involves a previously reported Suzuki–Miyaura cross-coupling reaction of tertiary allylic sulfone **3ga** to afford **4ga**, and **4gb** efficiently with 62% and 58% of isolated yields respectively. Subsequent deprotection of phenol then could complete the formal synthesis of (±)-sporochnol and (±)-bakuchiol (Fig. 1B).^20,21^ Starting from **3ga** in 2 steps our tertiary allylic sulfones can be used to prepare such natural products and other related compounds bearing all-carbon quaternary centers in a modular way.^22^

To gain mechanistic insight and the initial understanding on how the reaction works, we decided to study the reactivity of [Mo^0^L_*n*_] species (Fig. 3). The [Mo(bpy)(CO)_4_] complex^23^ was prepared on a large scale by reacting Mo(CO)_6_ and 2,2′-bipyridine (**L1**) in THF at 60 °C.^16^ As shown in Fig. 3, the structure was confirmed and further analyzed.^24^ Interestingly, the [Mo(bpy)(CO)_4_] complex was found to be catalytically more efficient when used under the standard conditions, supported by the formation of branched allylic sulfone product **3aa** in 96% yield. A small decline in yield of **3aa** in the [Mo(CO)_6_]/**L1** catalyst system was observed, thus providing evidence that a [Mo(bpy)(CO)_4_] complex is likely to be the active precatalyst species in this allylic sulfonylation reaction.^25^

**Fig. 2:**
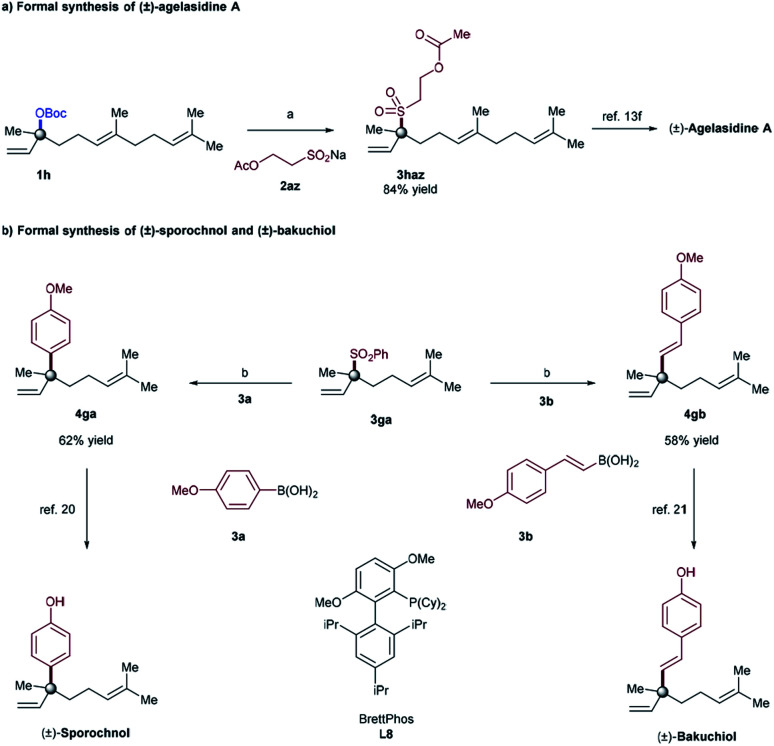
Importance of current research towards the synthesis of agelasidine A, sporochnol, and bakuchiol. Reaction conditions: (a) Mo(CO)_6_ (10 mol%), **L1** (15 mol%), **1h** (0.2 mmol), **2az** (0.3 mmol), EtOH (1.0 mL, 0.2 M), 60 °C, 24 hours. (b) Ni(cod)_2_ (10 mol%) ligand **L8** (12 mol%), **3ga** (0.2 mmol), **3a** or **3b** (0.7 equiv.), NaOEt (2.2 equiv.), PhMe (0.2 M), 24 h, 80 °C.

**Fig. 3:**
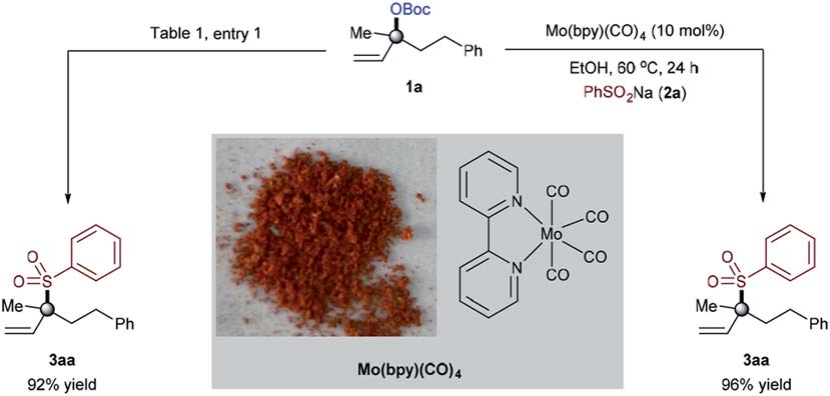
Mechanistic experiments.

## Conclusions

In conclusion we have developed a method for the allylic sulfonylation of α,α-disubstituted allylic electrophiles, using inexpensive and commercially available catalyst components (Mo(CO)_6_/2,2′-bipyridine). To the best of our knowledge, the presented methodology is the first example of the use of sodium sulfinates as the heteroatom nucleophile reagent with tertiary allylic electrophiles to employ the group 6 catalyst in allylic substitution of tertiary allylic electrophiles to form C–S bonds. The process is characterized by its atom economic procedure, wide substrate scope, and excellent regioselectivity profile even in the context of late-stage functionalization, thus providing ample opportunities for further derivatization through traditional Suzuki cross-coupling reactions (as presented in Fig. 2b). Investigations of enantioselective reactions, the mechanism and extension to other heteroatom nucleophiles are currently ongoing and will be reported in due course.

## Conflicts of interest

The authors declare no conflicts of interest.

## Supplementary Material

SC-011-D0SC01763A-s001

SC-011-D0SC01763A-s002
