# Supplementary material for: Regioselective molybdenum-catalyzed allylic substitution of tertiary allylic electrophiles: methodology development and applications
Source: Chem Sci. 2020 May 6;11(21):5481–6. doi: 10.1039/d0sc01763a (PMC8159339; doi:10.1039/d0sc01763a)

Electronic Supplementary Material (ESI) for Chemical Science.
This journal is © The Royal Society of Chemistry 2020

Regioselective molybdenum-catalyzed allylic sulfonylation of tertiary allylic electrophiles: Methodology development and applications

Muhammad Salman, Yaoyao Xu, Shahid Khan, Junjie Zhang and Ajmal Khan*

Department of Applied Chemistry, School of Science, and Xi'an Key Laboratory of Sustainable Energy Materials Chemistry, Xi'an Jiaotong University, Xi'an 710049, P. R. China

E-mail: ajmalkhan@xjtu.edu.cn

Table of Contents

General experimental details ……………………………………………………………........S2

Characterization of allylic carbonates **1a-1r**………………………………………………. S3-S7

Details for the optimization conditions……………………………………………………. S8-S9

General procedure for the Mo-catalyzed allylic sulfonylation of allylic carbonates **1** with sodium sulfinate **2** ……………………………………………………………………………………S10

Characterization of products **3**………………………………………………………….... S10-S27

Formal synthesis of (±)-agelasidine A………………………………………………………...S27

Formal synthesis of (±)-sporochnol and (±)-bakuchiol…………………………………....S28-S29

Synthesis of Mo(bpy)($CO)_4$ complex…………………………………………………....S30

Mechanistic experiments………………………………………………………………....S30-31

References ……………………………………………………………………………………..S32

NMR charts …………………………………………………………………………….S33-S93

General experimental details

Analytical thin-layer chromatography (TLC) was carried out using 0.2 mm commercial silica gel plates (Yantai Jiangyou Silica Gel Development Co., Ltd., silica gel HSGF 254). Preparative column chromatography employing silica gel (Qingdao Shenghai Fine Silica Gel Chemical Co., Ltd., 200-300 mesh) was performed according to the method of Still. Solvents for the chromatography are listed as volume/volume ratios. High-resolution mass spectra (HRMS) were performed at Instrumental Analysis Center of Xi'an Jiao Tong University using ESI method. Proton nuclear magnetic resonance (^{1}H NMR) spectra were recorded with a Varian Mercuryplus 400 (400 MHz) spectrometer. Chemical shifts are reported in delta (δ) units, parts per million (ppm) downfield from tetramethylsilane or ppm relative to the center of the singlet at 7.26 ppm for deuteriochloroform. Coupling constants are reported in Hertz (Hz). Carbon-13 nuclear magnetic resonance (^{13}C NMR) spectra were recorded with a Varian Gemini 400 (100 MHz) spectrometer. Chemical shifts are reported in delta (δ) units, ppm relative to the center of the triplet at 77.0 ppm for deuteriochloroform. ^{13}C NMR spectra were routinely run with broadband decoupling. $Mo(CO)_6$, $(C_7H_8)_3Mo(CO)_3$, $(CH_3CN)_3Mo(CO)_3$, and bipyridyne compounds were purchased from Energy Chemicals and Aladin/Sigma-Aldrich companies and used as received. Tertiary allylic carbonates were synthesized according to the previously reported procedure.[1] Sodium sulfinates were prepared according to a method reported in literature.[2] All other chemicals were used as received from commercial resources.

Synthesis and Characterization of Tertiary Allylic Carbonates

Tertiary allylic carbonates **1** were synthesized according to the previously reported procedures.[1] All characterization data are in accordance with the literature. New substrates have been fully characterized.

tert-butyl (3-methyl-5-phenylpent-1-en-3-yl) carbonate (1a)

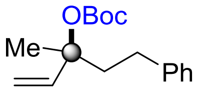

1a

All spectral data matched the published values.[1c]

tert-butyl (3-methylhex-1-en-3-yl) carbonate (1b)

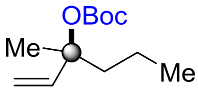

1b

Obtained as a colorless oil; ^{1}H NMR (400 MHz, $CDCl_3$) δ 5.93 (dd, *J* = 10.8, 17.6 Hz, 1H), 5.14 (d, *J* = 17.6 Hz, 1H), 5.05 (d, *J* = 10.8 Hz, 1H), 1.72–1.67 (m, 2H), 1.45 (s, 3H), 1.38 (s, 9H), 0.83 (t, *J* = 7.3 Hz, 3H); ^{13}C NMR (100 MHz, $CDCl_3$) δ 150.8, 140.7, 112.2, 82.5, 80.0, 40.9, 26.7, 22.0, 15.7, 13.1; HRMS (ESI-MS): Calcd. for $C_{12}H_{22}O_3$ (M + Na): 237.1461, Found: 237.1462.

tert-butyl (2-cyclohexylbut-3-en-2-yl) carbonate (1c)

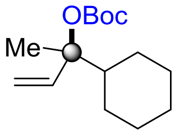

1c

Obtained as a colorless oil; ^{1}H NMR (400 MHz, $CDCl_3$) δ 5.91 (dd, *J* = 11.0, 17.6 Hz, 1H), 5.12 (d, *J* = 11.0 Hz, 1H), 5.05 (d, *J* = 17.6 Hz, 1H), 1.78–1.65 (m, 6H), 1.45 (s, 3H), 1.42–1.12 (m, 14H); ^{13}C NMR (100 MHz, $CDCl_3$) δ 149.7, 139.6, 111.1, 81.4, 78.9, 39.9, 25.6, 21.0, 14.7, 12.1; HRMS (ESI-MS): Calcd. for $C_{15}H_{26}O_3$ (M + Na): 277.1774, Found: 277.1773.

tert-butyl (3-methyldodec-1-en-3-yl) carbonate (1d)

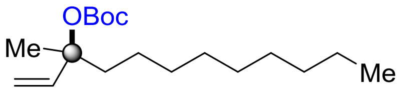

1d

Obtained as a colorless oil; ^{1}H NMR (400 MHz, $CDCl_3$) δ 6.00 (dd, *J* = 11.0, 17.6 Hz, 1H), 5.15 (d, *J* = 17.6 Hz, 1H), 5.12 (d, *J* = 11.0 Hz, 1H), 1.81–1.77 (m, 2H), 1.52 (s, 3H), 1.46 (9H), 1.33–1.26 (m, 14H), 0.88 (t, J = 6.4 Hz, 3H); ^{13}C NMR (100 MHz, $CDCl_3$) δ 151.8, 141.7,

113.2, 83.6, 81.0, 39.7, 31.8, 29.7, 29.4, 29.2, 27.7, 23.4, 23.1, 22.6, 14.0; HRMS (ESI-MS): Calcd. for $C_{18}H_{34}O_3$ (M + Na): 321.2400, Found: 321.2399.

5-(benzo[d][1,3]dioxol-5-yl)-3-methylpent-1-en-3-yl tert-butyl carbonate (1e)

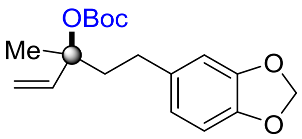

1e

Obtained as a colorless oil; ^{1}H NMR (400 MHz, $CDCl_3$) δ 6.70–6.59 (m, 3H), 6.05 (dd, *J* = 17.6, 10.8 Hz, 1H), 5.88 (s, 2H), 5.21 (d, *J* = 17.8 Hz, 1H), 5.17 (d, *J* = 10.8 Hz, 1H), 2.57–2.53 (m, 2H), 2.10–2.05 (m, 2H), 1.58 (s, 3H), 1.48 (s, 9); ^{13}C NMR (100 MHz, $CDCl_3$) δ 151.7, 147.4, 145.4, 141.3, 135.5, 120.8, 113.5, 108.6, 107.9, 100.5, 82.9, 81.1, 41.7, 29.6, 27.6, 23.3; HRMS (ESI-MS): Calcd. for $C_{18}H_{24}O_5$ (M + Na): 343.1516, Found: 343.1508.

5-(benzo[d][1,3]dioxol-5-yl)-3-methylpent-1-en-3-yl tert-butyl carbonate (1f)

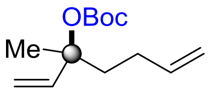

1f

Obtained as a colorless oil; ^{1}H NMR (400 MHz, $CDCl_3$) δ 6.01 (dd, *J* = 17.6, 11.0 Hz, 1H), 5.85–5.75 (m, 1H), 5.20–5.14 (m, 2H), 5.00 (d, *J* = 17.8 Hz, 1H), 4.96 (d, *J* = 11.0 Hz, 1H), 2.12–2.06 (m, 2H), 1.91–1.87 (m, 2H), 1.55 (s, 3H), 1.47 (s, 9); ^{13}C NMR (100 MHz, $CDCl_3$) δ 151.7, 141.4, 138.0, 114.5, 113.5, 83.1, 81.2, 38.4, 27.8, 27.7, 23.2; HRMS (ESI-MS): Calcd. for $C_{13}H_{22}O_3$ (M + Na): 249.1461, Found: 249.1460.

tert-butyl (3,7-dimethylocta-1,6-dien-3-yl) carbonate (1g)

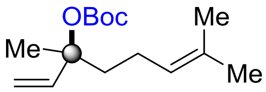

1g

All spectral data matched the published values.[1d]

tert-butyl (3,7,11-trimethyldodeca-1,6,10-trien-3-yl) carbonate (1h)

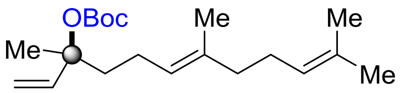

1h

All spectral data matched the published values.[1b]

tert-butyl (6-chloro-3-methylhex-1-en-3-yl) carbonate (1i)

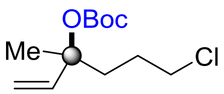

1i

Obtained as a colorless oil; ^{1}H NMR (400 MHz, $CDCl_3$) δ 6.01 (dd, J = 17.2, 11.3 Hz, 1H), 5.19 (d, J = 17.2 Hz, 1H) , 5.17 (d, J = 11.3 Hz, 1H), 3.56–3.51 (m, 2H), 1.98–1.92 (m, 2H), 1.87–1.79 (m, 2H), 1.56 (s, 3H), 1.46 (s, 9); ^{13}C NMR (100 MHz, $CDCl_3$) δ 151.6, 141.0, 113.7, 82.7, 81.2, 44.7, 37.0, 27.6, 26.8, 23.3; HRMS (ESI-MS): Calcd. for $C_{12}H_{21}ClO_3$ (M + Na): 271.1071, Found: 271.1059.

5-(benzyloxy)-3-methylpent-1-en-3-yl tert-butyl carbonate (1j)

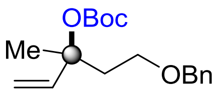

1j

Obtained as a colorless oil; ^{1}H NMR (400 MHz, $CDCl_3$) δ 7.35–7.25 (m, 5H), 6.03 (dd, J = 17.5, 11.0 Hz, 1H), 5.18 (d, J = 17.5 Hz, 1H) , 5.15 (d, J = 11.0 Hz, 1H), 4.48 (s, 2H), 3.61–3.52 (m, 2H), 2.26–2.14 (m, 2H), 1.57 (s, 3H), 1.44 (s, 9); ^{13}C NMR (100 MHz, $CDCl_3$) δ 151.7, 141.3, 138.3, 128.2, 127.5, 127.4, 113.5, 82.3, 81.3, 72.8, 65.9, 38.9, 27.7, 23.9; HRMS (ESI-MS): Calcd. for $C_{18}H_{26}O_4$ (M + Na): 329.1723, Found: 329.1719.

3-((tert-butoxycarbonyl)oxy)-3-methylpent-4-en-1-yl benzoate (1k)

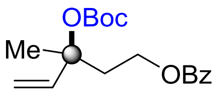

1k

Obtained as a colorless oil; ^{1}H NMR (400 MHz, $CDCl_3$) δ 8.05–8.02 (m, 2H), 7.57–7.53 (m, 1), 7.45–7.41 (m, 2H), 6.09 (dd, J = 17.5, 11.0 Hz, 1H), 5.25 (d, J = 17.5 Hz, 1H) , 5.21 (d, J = 11.0 Hz, 1H), 4.48–4.38 (m, 2H), 2.33 (t, J = 6.8 Hz, 2H), 1.66 (s, 3H), 1.46 (s, 9); ^{13}C NMR (100 MHz, $CDCl_3$) δ 166.3, 151.6, 140.8, 132.7, 130.0, 129.4, 128.2, 114.0, 81.8, 81.5, 60.7, 38.2, 27.6, 23.6; HRMS (ESI-MS): Calcd. for $C_{18}H_{24}O_5$ (M + Na): 343.1515, Found: 343.1516.

tert-butyl (3-methyl-5-(methylthio)pent-1-en-3-yl) carbonate (1l)

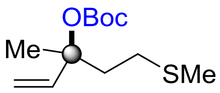

1l

Obtained as a colorless oil; ^{1}H NMR (400 MHz, $CDCl_3$) δ 6.00 (dd, J = 17.5, 11.0 Hz, 1H), 5.20 (d, J = 17.5 Hz, 1H) , 5.18 (d, J = 11.0 Hz, 1H), 2.51–2.47 (m, 2H), 2.14–2.09 (m, 5H), 1.56 (s, 3H), 1.47 (s, 9); ^{13}C NMR (100 MHz, $CDCl_3$) δ 151.6, 140.9, 113.9, 82.7, 81.5, 39.5,

28.1, 27.7, 23.4, 15.4; HRMS (ESI-MS): Calcd. for $C_{12}H_{22}O_3S$ (M + Na): 269.1182, Found: 269.1177.

tert-butyl (5,5-dimethoxy-3-methylpent-1-en-3-yl) carbonate (1m)

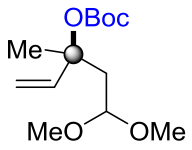

1m

Obtained as a colorless oil; ^{1}H NMR (400 MHz, $CDCl_3$) δ 6.04 (dd, *J* = 17.5, 11.0 Hz, 1H), 5.21 (d, *J* = 17.5 Hz, 1H) , 5.15 (d, *J* = 11.0 Hz, 1H), 4.52 (t, *J* = 6.8 Hz, 2H), 3.31 (s, 3H), 3.29 (m, 3H), 2.25–2.16 (m, 2H), 1.57 (s, 3H), 1.47 (s, 9); ^{13}C NMR (100 MHz, $CDCl_3$) δ 151.5, 141.5, 113.3, 101.3, 81.3, 52.5, 52.2, 41.2, 27.6, 24.1; HRMS (ESI-MS): Calcd. for $C_{13}H_{24}O_5$ (M + Na): 283.1516, Found: 283.1510.

di-tert-butyl (3-methylpent-4-ene-1,3-diyl) bis(carbonate) (1n)

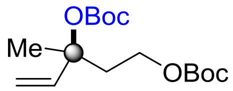

1n

Obtained as a colorless oil; ^{1}H NMR (400 MHz, $CDCl_3$) δ 6.02 (dd, *J* = 17.6, 11.0 Hz, 1H), 5.22 (d, *J* = 17.6 Hz, 1H) , 5.17 (d, *J* = 11.0 Hz, 1H), 4.21–4.11 (m, 2H), 2.22 (t, *J* = 7.2 Hz, 2H), 1.59 (s, 3H), 1.53 (s, 3H), 1.48 (s, 9H), 1.47 (s, 9); ^{13}C NMR (100 MHz, $CDCl_3$) δ 153.4, 151.6, 140.8, 114.1, 81.9, 81.7, 81.6, 62.8, 38.1, 27.8, 27.7, 27.3, 23.7; HRMS (ESI-MS): Calcd. for $C_{16}H_{28}O_6$ (M + Na): 339.1778, Found: 339.1774.

***tert*-butyl (5-hydroxy-3-methylpent-1-en-3-yl) carbonate (1o)**

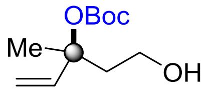

1o

Obtained as a colorless oil; ^{1}H NMR (400 MHz, $CDCl_3$) δ 5.91 (dd, *J* = 17.6, 11.0 Hz, 1H), 5.26 (d, *J* = 17.6 Hz, 1H) , 5.07 (d, *J* = 11.0 Hz, 1H), 4.24–4.14 (m, 2H), 2.49 (brs, 1H), 1.99–1.84 (m, 2H), 1.56 (s, 3H), 1.47 (s, 9); ^{13}C NMR (100 MHz, $CDCl_3$) δ 151.6, 142.1, 112.1, 82.1, 81.9, 63.6, 40.0, 27.6, 23.6; HRMS (ESI-MS): Calcd. for $C_{11}H_{20}O_4$ (M + Na): 239.1254, Found: 239.1258.

tert-butyl (6-hydroxy-3-methylhex-1-en-3-yl) carbonate (1p)

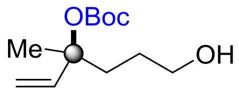

1p

Obtained as a colorless oil; ^{1}H NMR (400 MHz, $CDCl_3$) δ 6.01 (dd, *J* = 17.6, 11.0 Hz, 1H), 5.16 (d, *J* = 17.6 Hz, 1H) , 5.14 (d, *J* = 11.0 Hz, 1H), 3.91–3.80 (m, 2H), 3.64 (brs, 1H), 1.92–1.78 (m, 4H), 1.54 (s, 3H), 1.45 (s, 9H); ^{13}C NMR (100 MHz, $CDCl_3$) δ 151.8, 141.4, 113.6, 83.3, 81.2, 67.3, 41.5, 27.7, 26.0, 23.2; HRMS (ESI-MS): Calcd. for $C_{12}H_{22}O_4$ (M + Na): 253.1410, Found: 253.1408.

tert-butyl(7-(3,7-dimethyl-2,6-dioxo-2,3,4,5,6,7-hexahydro-1H-purin-1-yl)-3-methylhept-1-en-3-yl) carbonate (1q)

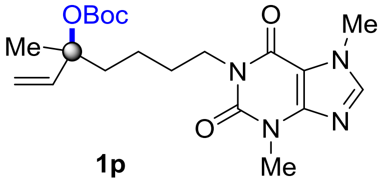

1p

Obtained as an orange solid; ^{1}H NMR (400 MHz, $CDCl_3$) δ 7.51 (s, 1H), 6.01 (dd, *J* = 11.0, 17.6 Hz, 1H), 5.17 (d, *J* = 11.0 Hz, 1H) , 5.14 (d, *J* = 17.6 Hz, 1H), 4.01–3.97 (m, 5H), 3.57 (s, 3H), 1.86–1.81 (m, 2H), 1.68–1.60 (m, 2H), 1.54 (s, 3H), 1.46 (s, 9H), 1.26 (t, *J* = 7.2 Hz, 2H); ^{13}C NMR (100 MHz, $CDCl_3$) δ 155.2, 151.8, 151.4, 148.7, 141.5, 141.3, 113.5, 107.6, 83.5, 81.3, 60.3, 41.1, 39.7, 33.5, 29.6, 27.8, 23.0, 21.0; HRMS (ESI-MS): Calcd. for $C_{20}H_{30}N_4O_5$ (M + Na): 429.2108, Found: 429.2106.

tert-butyl (2-phenylbut-3-en-2-yl) carbonate (1r)

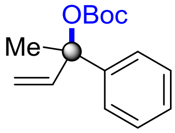

1r

All spectral data matched the published values.[1a]

Details for the Optimization Conditions

Table S1. Evaluation of ligand for the regioselective Mo-catalyzed sulfonylation of allylic carbonate **1a** with sodium sulfinate **2a**[a]

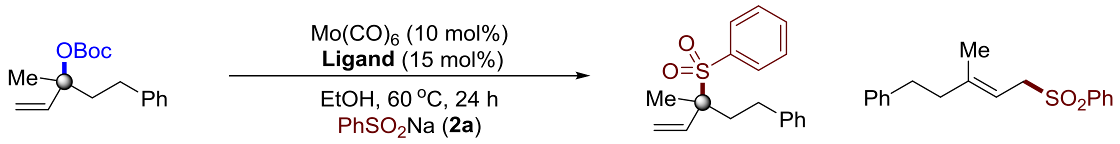

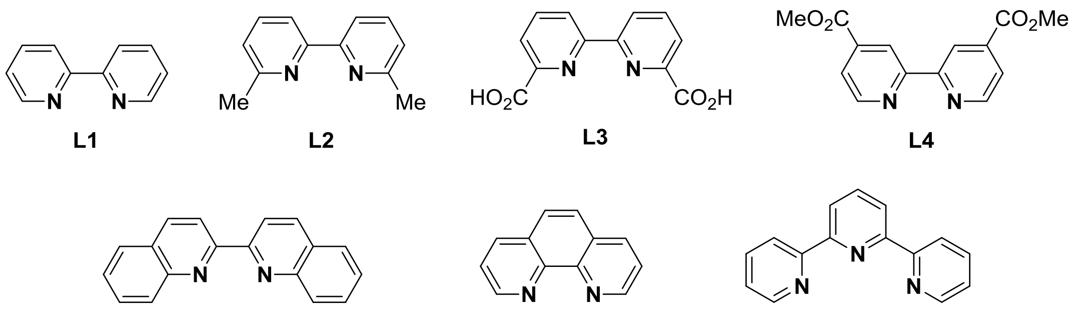

entry	ligand	**3aa/4aa**[b]	Yield (%)[c]
1	**L1**	99:1	92
2	**L2**	99:1	87
3	**L3**	99:1	35
4	**L4**	99:1	52
5	**L5**	25:1	16
6	**L6**	--	0
7	**L7**	--	< 10

[a] Reaction conditions: $Mo(CO)_6$ (10 mol%), mmol), ligand (15 mol%), **1a** (0.2 mmol), $PhSO_2Na$ **2a** (0.3 mmol), EtOH (1.0 mL, 0.2 M), 60 ℃, 24 hours. [b] Determined by ^{1}H-NMR of the crude reaction mixture. [c] Isolated yields.

Table S2. Evaluation of solvent for the regioselective Mo-catalyzed sulfonylation of allylic carbonate **1a** with sodium sulfinate **2a**[a]

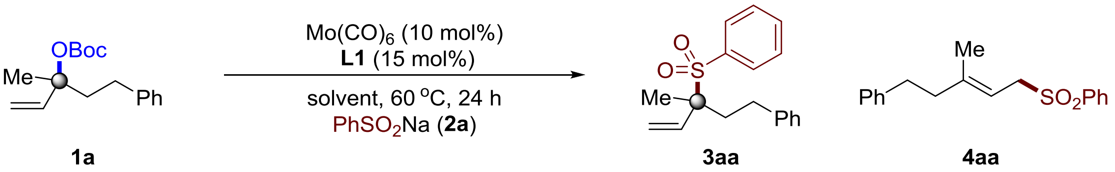

entry	solvent	3aa/4aa[b]	Yield (%)[c]
1	CH_3CN	--	10
2	THF	--	< 5
3	DCE	25:1	35
4	1,4-dioxane	--	0
5	toluene	--	< 5
6	DCM	--	< 10
7	^{i}PrOH	99:1	77
8	THF/EtOH (5:1)	25:1	25
9	DCE/EtOH (5:1)	25:1	63

[a] Reaction conditions: $Mo(CO)_6$ (10 mol%), mmol), ligand (**L1**) (15 mol%), **1a** (0.2 mmol), $PhSO_2Na$ **2a** (0.3 mmol), solvent (1.0 mL, 0.2 M), 60 °C, 24 hours. [b] Determined by ^{1}H-NMR of the crude reaction mixture. [c] Isolated yields.

Table S3. Evaluation of molybdenum-catalyst for Mo-catalyzed sulfonylation of allylic carbonate **1a** with sodium sulfinate **2a**[a]

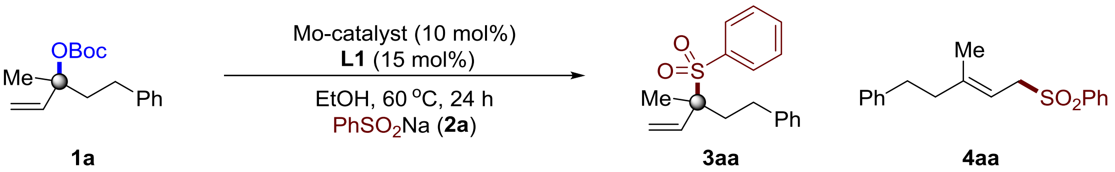

entry	Mo-catalyst	3aa/4aa[b]	Yield (%)[c]
1	Without molybdenum catalyst	--	--
2	Without ligand	--	--
3	$(C_7H_8)_3Mo(CO)_3$	99:1	82
4	$(CH_3CN)_3Mo(CO)_3$	99:1	86

[a] Reaction conditions: Mo-catalyst (10 mol%), mmol), ligand (**L1**) (15 mol%), **1a** (0.2 mmol), $PhSO_2Na$ **2a** (0.3 mmol), EtOH (1.0 mL, 0.2 M), 60 °C, 24 hours. [b] Determined by ^{1}H-NMR of the crude reaction mixture. [c] Isolated yields.

General procedure for the allylic sulfonylation of α,α-disubstituted allylic carbonate 1a with sodium sulfinates 2

To an oven dried screw-cap reaction tube equipped with a magnetic stir bar, $Mo(CO)_6$ (5.3 mg, 10 mol%), 2,2'-bipyridyne ligand (**L1**) (4.7 mg, 15 mol%), allylic carbonate **1a** (55.28 mg, 0.2 mmol), and sodium benzenesulfinate **2a** (49.25 mg, 0.3 mmol) were added. The reaction tube was sealed with rubber-septum, then evacuated and backfilled with nitrogen. Anhydrous ethanol (0.2 M, 1 mL) was added via syringe. The resulting mixture was stirred at 60 ˚C for 24 hours. The reaction mixture was cooled to room temperature and the residue was purified by flash column chromatography on silica gel to afford the pure tertiary allylic sulfone **3aa**.

Scale-up Experiment: In a 100 mL round-bottom flask equipped with a magnetic stir bar, $Mo(CO)_6$ (132.0 mg, 10 mol%), 2,2'-bipyridyne ligand (**L1**) (117.1 mg, 15 mol%), allylic carbonate **1g** (1.38 g, 5.0 mmol), and sodium benzenesulfinate **2a** (1.23 g, 7.5 mmol) were added. The reaction tube was sealed with rubber-septum, then evacuated and backfilled with nitrogen. Anhydrous ethanol (0.2 M, 25 mL) was added via syringe. The resulting mixture was stirred at 60 ˚C for 24 hours. The reaction mixture was cooled to room temperature and then quenched with water (10 mL). The organic portion was extracted with CH_2Cl_2 and the solvent was removed *in vacuo* with the aid of a rotary evaporator. The obtained residue was purified by flash column chromatography on silica gel to afford the pure sulfone **3aa** in 87% isolated yield.

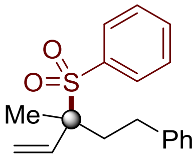

3aa

(3-methyl-3-(phenylsulfonyl)pent-4-en-1-yl)benzene (3aa) was prepared according to the general procedure from **1a** and **2a**. The crude product was purified by flash column chromatography (Petroleum ether/EtOAc = 50:1) on silica gel to provide the title compound as a white solid in 92% yield (55.2 mg); ^{1}H NMR (400 MHz, $CDCl_3$) δ 7.82–7.81 (m, 2H), 7.64–7.61 (m, 1H), 7.53–7.49 (m, 2H), 7.30–7.26 (m, 2H), 7.21–7.14 (m, 3H), 6.00 (dd, *J* = 10.7, 17.4 Hz, 1H), 5.43 (d, *J* = 10.7 Hz, 1H), 5.13 (d, *J* = 17.4 Hz, 1H), 2.60–2.48 (m, 2H), 2.25–2.21 (m, 2H), 1.45 (s, 3H); ^{13}C NMR (100 MHz, $CDCl_3$) δ 141.0, 135.2, 135.1, 133.6, 130.8, 128.5, 128.4, 128.3, 126.2, 120.6, 68.2, 34.7, 30.1, 16.4; HRMS (ESI-MS): Calcd. for $C_{18}H_{20}O_2S$ (M + Na): 323.1082, Found: 323.1081.

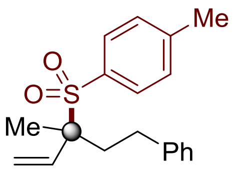

3ab

1-methyl-4-((3-methyl-5-phenylpent-1-en-3-yl)sulfonyl)benzene (3ab) was prepared according to the general procedure from **1a** and **2b**. The crude product was purified by flash column chromatography (Petroleum ether/EtOAc = 50:1) on silica gel to provide the title compound as a white solid in 93% yield (58.4 mg); ^{1}H NMR (400 MHz, $CDCl_3$) δ 7.69–7.67 (m, 2H), 7.30–7.26 (m, 4H), 7.21–7.14 (m, 3H), 5.99 (dd, *J* = 10.8, 17.5 Hz, 1H), 5.42 (d, *J* = 10.8 Hz, 1H), 5.13 (d, *J* = 17.5 Hz, 1H), 2.59–2.49 (m, 2H), 2.43 (s, 3H), 2.24–2.19 (m, 2H), 1.44 (s, 3H); ^{13}C NMR (100 MHz, $CDCl_3$) δ 144.5, 141.1, 135.2, 132.2, 130.7, 129.0, 128.5, 128.3, 126.1, 120.7, 68.1, 34.7, 30.2, 21.6, 16.4; HRMS (ESI-MS): Calcd. for $C_{19}H_{22}O_2S$ (M + Na): 337.1238, Found: 337.1232.

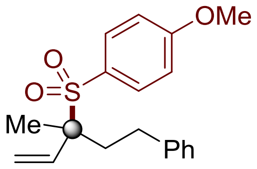

3ac

1-methoxy-4-((3-methyl-5-phenylpent-1-en-3-yl)sulfonyl)benzene (3ac) was prepared according to the general procedure from **1a** and **2c**. The crude product was purified by flash column chromatography (Petroleum ether/EtOAc = 50:1) on silica gel to provide the title compound as a white solid in 90% yield (59.5 mg); ^{1}H NMR (400 MHz, $CDCl_3$) δ 7.74–7.72 (m, 2H), 7.30–7.26 (m, 2H), 7.21–7.14 (m, 3H), 6.97–6.95 (m, 2H), 5.99 (dd, *J* = 10.8, 17.5 Hz, 1H), 5.42 (d, *J* = 10.8 Hz, 1H), 5.14 (d, *J* = 17.5 Hz, 1H), 3.87 (s, 3H), 2.61–2.47 (m, 2H), 2.23–2.17 (m, 2H), 1.44 (s, 3H); ^{13}C NMR (100 MHz, $CDCl_3$) δ 163.7, 141.1, 135.3, 132.8, 128.5, 128.3, 126.7, 126.1, 120.6, 113.6, 68.2, 55.6, 34.8, 30.2, 16.4; HRMS (ESI-MS): Calcd. for $C_{19}H_{22}O_3S$ (M + Na): 353.1187, Found: 353.1188.

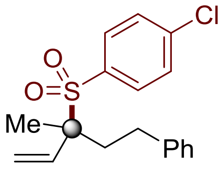

3ad

1-chloro-4-((3-methyl-5-phenylpent-1-en-3-yl)sulfonyl)benzene (3ad) was prepared according to the general procedure from **1a** and **2d**. The crude product was purified by flash col-

umn chromatography (Petroleum ether/EtOAc = 50:1) on silica gel to provide the title compound as a white solid in 87% yield (58.2 mg); ^{1}H NMR (400 MHz, $CDCl_3$) δ 7.76–7.72 (m, 2H), 7.50–7.46 (m, 2H), 7.31–7.26 (m, 2H), 7.22–7.15 (m, 3H), 5.99 (dd, J = 10.8, 17.5 Hz, 1H), 5.44 (d, J = 10.8 Hz, 1H), 5.14 (d, J = 17.5 Hz, 1H), 2.63–2.48 (m, 2H), 2.24–2.20 (m, 2H), 1.44 (s, 3H); ^{13}C NMR (100 MHz, $CDCl_3$) δ 140.9, 140.4, 134.9, 133.7, 132.1, 128.8, 128.5, 128.3, 126.2, 121.2, 68.4, 34.6, 30.1, 16.4; HRMS (ESI-MS): Calcd. for $C_{18}H_{19}ClO_2S$ (M + Na): 357.0692, Found: 357.0686.

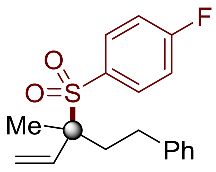

3ae

1-fluoro-4-((3-methyl-5-phenylpent-1-en-3-yl)sulfonyl)benzene (3ae) was prepared according to the general procedure from **1a** and **2e**. The crude product was purified by flash column chromatography (Petroleum ether/EtOAc = 50:1) on silica gel to provide the title compound as a white solid in 85% yield (54.1 mg); ^{1}H NMR (400 MHz, $CDCl_3$) δ 7.84–7.80 (m, 2H), 7.30–7.26 (m, 2H), 7.22–7.14 (m, 5H), 6.00 (dd, J = 10.8, 17.5 Hz, 1H), 5.43 (d, J = 10.8 Hz, 1H), 5.13 (d, J = 17.5 Hz, 1H), 2.63–2.48 (m, 2H), 2.25–2.20 (m, 2H), 1.44 (s, 3H); ^{13}C NMR (100 MHz, $CDCl_3$) δ 166.7, 165.0, 140.9, 135.1, 133.5, 131.2, 131.1, 128.5, 128.3, 126.2, 121.0, 115.8, 115.7, 68.3, 34.6, 30.1, 16.4; HRMS (ESI-MS): Calcd. for $C_{18}H_{19}FO_2S$ (M + Na): 341.0982, Found: 341.0987.

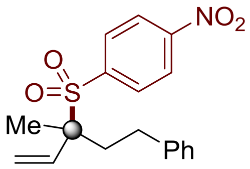

3af

1-((3-methyl-5-phenylpent-1-en-3-yl)sulfonyl)-4-nitrobenzene (3af) was prepared according to the general procedure from **1a** and **2f**. The crude product was purified by flash column chromatography (Petroleum ether/EtOAc = 50:1) on silica gel to provide the title compound as a white solid in 75% yield (51.8 mg); ^{1}H NMR (400 MHz, $CDCl_3$) δ 8.36–8.34 (m, 2H), 8.02–8.01 (m, 2H), 7.31–7.28 (m, 2H), 7.23–7.15 (m, 3H), 6.02 (dd, J = 10.8, 17.5 Hz, 1H), 5.47 (d, J = 10.8 Hz, 1H), 5.14 (d, J = 17.5 Hz, 1H), 2.63–2.52 (m, 2H), 2.27–2.24 (m, 2H), 1.47 (s, 3H); ^{13}C NMR (100 MHz, $CDCl_3$) δ 150.6, 141.0, 140.6, 134.6, 132.2, 128.6, 128.3, 126.4, 123.5, 121.7, 68.9, 34.4, 30.0, 16.4; HRMS (ESI-MS): Calcd. for $C_{18}H_{19}NO_4S$ (M + Na): 368.0927, Found: 368.0925.

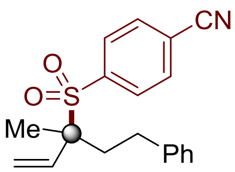

3ag

4-((3-methyl-5-phenylpent-1-en-3-yl)sulfonyl)benzonitrile (3ag) was prepared according to the general procedure from **1a** and **2g**. The crude product was purified by flash column chromatography (Petroleum ether/EtOAc = 50:1) on silica gel to provide the title compound as a white solid in 72% yield (46.8 mg); ^{1}H NMR (400 MHz, $CDCl_3$) δ 7.94–7.93 (m, 2H), 7.82–7.81 (m, 2H), 7.30–7.28 (m, 2H), 7.23–7.15 (m, 3H), 6.00 (dd, *J* = 10.8, 17.5 Hz, 1H), 5.46 (d, *J* = 10.8 Hz, 1H), 5.14 (d, *J* = 17.5 Hz, 1H), 2.62–2.51 (m, 2H), 2.25–2.22 (m, 2H), 1.45 (s, 3H); ^{13}C NMR (100 MHz, $CDCl_3$) δ 140.6, 139.5, 134.7, 132.1, 131.4, 128.6, 128.3, 126.4, 121.6, 117.4, 117.2, 68.8, 34.6, 30.0, 16.4; HRMS (ESI-MS): Calcd. for $C_{19}H_{19}NO_2S$ (M + Na): 348.1034, Found: 348.1023.

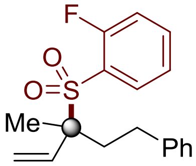

3ah

1-fluoro-2-((3-methyl-5-phenylpent-1-en-3-yl)sulfonyl)benzene (3ah) was prepared according to the general procedure from **1a** and **2h**. The crude product was purified by flash column chromatography (Petroleum ether/EtOAc = 50:1) on silica gel to provide the title compound as a white solid in 88% yield (56.0 mg); ^{1}H NMR (400 MHz, $CDCl_3$) δ 7.81–7.77 (m, 1H), 7.64–7.59 (m, 1H), 7.30–7.25 (m, 3H), 7.21–7.15 (m, 4H), 6.07 (dd, *J* = 10.8, 17.5 Hz, 1H), 5.43 (d, *J* = 10.8 Hz, 1H), 5.15 (d, *J* = 17.5 Hz, 1H), 2.65–2.51 (m, 2H), 2.34–2.20 (m, 2H), 1.50 (s, 3H); ^{13}C NMR (100 MHz, $CDCl_3$) δ 161.5, 159.0, 140.8, 136.3, 134.6, 133.8, 128.4, 128.2, 126.1, 124.1, 122.9, 122.8, 120.9, 117.4, 117.2, 69.4, 34.2, 30.0, 16.0; HRMS (ESI-MS): Calcd. for $C_{18}H_{19}FO_2S$ (M + Na): 341.0980, Found: 341.0982.

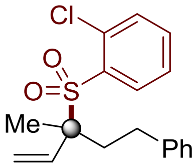

3ai

1-chloro-2-((3-methyl-5-phenylpent-1-en-3-yl)sulfonyl)benzene (3ai) was prepared according to the general procedure from **1a** and **2i**. The crude product was purified by flash col-

umn chromatography (Petroleum ether/EtOAc = 50:1) on silica gel to provide the title compound as a white solid in 87% yield (58.3 mg); ^{1}H NMR (400 MHz, CDCl$_3$) δ 7.96–7.94 (m, 1H), 7.54–7.47 (m, 2H), 7.41–7.37 (m, 1H), 7.30–7.25 (m, 2H), 7.21–7.15 (m, 3H), 6.09 (dd, *J* = 10.8, 17.5 Hz, 1H), 5.43 (d, *J* = 10.8 Hz, 1H), 5.15 (d, *J* = 17.5 Hz, 1H), 2.64–2.50 (m, 2H), 2.36–2.24 (m, 2H), 1.52 (s, 3H); ^{13}C NMR (100 MHz, CDCl$_3$) δ 140.8, 135.2, 134.9, 134.8, 134.6, 133.0, 132.5, 128.4, 128.2, 126.6, 126.1, 121.0, 70.5, 34.5, 29.9, 16.3; HRMS (ESI-MS): Calcd. for $C_{18}H_{19}ClO_2S$ (M + Na): 357.0686, Found: 357.0693.

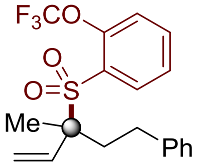

3aj

1-((3-methyl-5-phenylpent-1-en-3-yl)sulfonyl)-2-(trifluoromethoxy)benzene (3aj) was prepared according to the general procedure from **1a** and **2j**. The crude product was purified by flash column chromatography (Petroleum ether/EtOAc = 50:1) on silica gel to provide the title compound as a white solid in 72% yield (55.4 mg); ^{1}H NMR (400 MHz, CDCl$_3$) δ 7.95–7.92 (m, 1H), 7.68–7.64 (m, 1H), 7.42–7.37 (m, 2H),7.30–7.25 (m, 2H), 7.21–7.15 (m, 3H), 6.07 (dd, *J* = 10.8, 17.5 Hz, 1H), 5.40 (d, *J* = 10.8 Hz, 1H), 5.12 (d, *J* = 17.5 Hz, 1H), 2.64–2.51 (m, 2H), 2.34–2.20 (m, 2H), 1.48 (s, 3H); ^{13}C NMR (100 MHz, CDCl$_3$) δ 147.9, 147.8, 141.0, 135.7, 134.9, 134.9, 128.6, 128.4, 127.6, 126.3, 126.2, 120.8, 120.7, 120.6, 70.0, 34.4, 30.1, 16.2; HRMS (ESI-MS): Calcd. for $C_{19}H_{19}F_3O_3S$ (M + Na): 407.0899, Found: 407.0903.

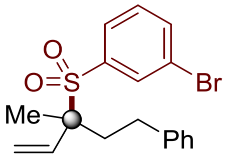

3ak

1-bromo-3-((3-methyl-5-phenylpent-1-en-3-yl)sulfonyl)benzene (3ak) was prepared according to the general procedure from **1a** and **2k**. The crude product was purified by flash column chromatography (Petroleum ether/EtOAc = 50:1) on silica gel to provide the title compound as a white solid in 82% yield (62.2 mg); ^{1}H NMR (400 MHz, CDCl$_3$) δ 7.96–7.95 (m, 1H), 7.76–7.63 (m, 2H), 7.41–7.37 (m, 1H),7.31–7.26 (m, 2H), 7.23–7.15 (m, 3H), 6.01 (dd, *J* = 10.8, 17.5 Hz, 1H), 5.46 (d, *J* = 10.8 Hz, 1H), 5.15 (d, *J* = 17.5 Hz, 1H), 2.64–2.50 (m, 2H), 2.28–2.19 (m, 2H), 1.45 (s, 3H); ^{13}C NMR (100 MHz, CDCl$_3$) δ 140.8, 137.0, 136.7, 134.9, 133.4, 129.9, 129.3, 128.5, 128.3, 126.3, 122.5, 121.2, 68.6, 34.5, 30.1, 16.5; HRMS (ESI-MS): Calcd. for $C_{18}H_{19}BrO_2S$ (M + Na): 401.0181, Found: 401.0190.

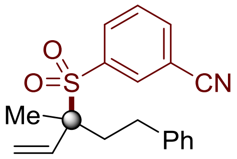

3al

3-((3-methyl-5-phenylpent-1-en-3-yl)sulfonyl)benzonitrile (3al) was prepared according to the general procedure from **1a** and **2l**. The crude product was purified by flash column chromatography (Petroleum ether/EtOAc = 50:1) on silica gel to provide the title compound as a white solid in 78% yield (50.8 mg); ^{1}H NMR (400 MHz, $CDCl_3$) δ 8.10–8.09 (m, 1H), 8.04–8.02 (m, 1H), 7.91–7.88 (m, 1H), 7.66 (t, J = 7.8 Hz, 1H), 7.31–7.26 (m, 2H), 7.23–7.15 (m, 3H), 6.01 (dd, J = 10.8, 17.5 Hz, 1H), 5.47 (d, J = 10.8 Hz, 1H), 5.12 (d, J = 17.5 Hz, 1H), 2.64–2.50 (m, 2H), 2.28–2.21 (m, 2H), 1.44 (s, 3H); ^{13}C NMR (100 MHz, $CDCl_3$) δ 140.6, 137.0, 136.8, 134.7, 134.6, 134.2, 129.5, 128.6, 128.3, 126.4, 121.6, 117.1, 113.2, 68.8, 34.4, 30.1, 16.5; HRMS (ESI-MS): Calcd. for $C_{19}H_{19}NO_2S$ (M + Na): 348.1029, Found: 348.1025.

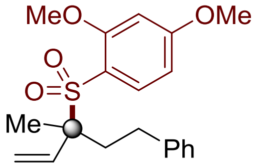

3am

2,4-dimethoxy-1-((3-methyl-5-phenylpent-1-en-3-yl)sulfonyl)benzene (3am) was prepared according to the general procedure from **1a** and **2m**. The crude product was purified by flash column chromatography (Petroleum ether/EtOAc = 50:1) on silica gel to provide the title compound as a white solid in 94% yield (67.7 mg); ^{1}H NMR (400 MHz, $CDCl_3$) δ 7.43 (dd, J = 1.4, 5.6 Hz, 1H), 7.29–7.27 (m, 2H), 7.24 (d, J = 1.4 Hz, 1H), 7.21–7.18 (m, 1H), 7.16–7.15 (m, 2H), 6.94 (d, J = 5.6 Hz, 1H), 6.01 (dd, J = 10.8, 17.5 Hz, 1H), 5.43 (d, J = 10.8 Hz, 1H), 5.15 (d, J = 17.5 Hz, 1H), 3.95 (s, 3H), 3.89 (s, 3H), 2.61–2.50 (m, 2H), 2.26–2.18 (m, 2H), 1.45 (s, 3H); ^{13}C NMR (100 MHz, $CDCl_3$) δ 153.3, 148. 4, 141.1, 135.4, 128.5, 128.3, 126.8, 126.1, 124.9, 120.6, 113.0, 110.0, 68.3, 56.2, 56.1, 34.8, 30.2, 16.4; HRMS (ESI-MS): Calcd. for $C_{20}H_{24}O_4S$ (M + Na): 383.1288, Found: 383.1297.

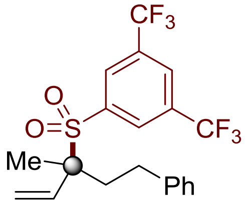

3an

1-((3-methyl-5-phenylpent-1-en-3-yl)sulfonyl)-3,5-bis(trifluoromethyl)benzene (3an) was prepared according to the general procedure from **1a** and **2n**. The crude product was purified by flash column chromatography (Petroleum ether/EtOAc = 50:1) on silica gel to provide the title compound as a white solid in 95% yield (82.8 mg); ^{1}H NMR (400 MHz, $CDCl_3$) δ 8.26 (s, 2H), 8.13 (s, 1H), 7.31–7.28 (m, 2H), 7.23–7.20 (m, 1H), 7.18–7.16 (m, 2H), 6.07 (dd, J = 10.8, 17.5 Hz, 1H), 5.47 (d, J = 10.8 Hz, 1H), 5.18 (d, J = 17.5 Hz, 1H), 2.67–2.55 (m, 2H), 2.34–2.26 (m, 2H), 1.42 (s, 3H); ^{13}C NMR (100 MHz, $CDCl_3$) δ 140.4, 137.9, 134.8, 132.6, 132.3, 132.1, 131.9, 131.0, 130.9, 128.6, 128.2, 127.2, 126.4, 123.3, 121.7, 121.5, 67.0, 34.0, 30.0, 16.5; HRMS (ESI-MS): Calcd. for $C_{20}H_{18}F_6O_2S$ (M + Na): 459.0824, Found: 459.0836.

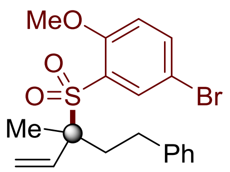

3ao

4-bromo-1-methoxy-2-((3-methyl-5-phenylpent-1-en-3-yl)sulfonyl)benzene (3ao) was prepared according to the general procedure from **1a** and **2o**. The crude product was purified by flash column chromatography (Petroleum ether/EtOAc = 50:1) on silica gel to provide the title compound as a white solid in 84% yield (68.8 mg); ^{1}H NMR (400 MHz, $CDCl_3$) δ 7.94 (d, J = 1.7 Hz, 1H), 7.65 (dd, J = 1.7, 5.8 Hz, 1H), 7.33–7.27 (m, 3H), 7.21–7.15 (m, 3H), 6.06 (dd, J = 10.8, 17.5 Hz, 1H), 5.39 (d, J = 10.8 Hz, 1H), 5.14 (d, J = 17.5 Hz, 1H), 3.83 (s, 3H), 2.63–2.55 (m, 2H), 2.31–2.21 (m, 2H), 1.48 (s, 3H); ^{13}C NMR (100 MHz, $CDCl_3$) δ 149.2, 141.1, 138.3, 136.9, 136.5, 135.5, 128.5, 128.3, 126.1, 123.7, 121.1, 119.7, 70.1, 56.1, 34.7, 30.1, 16.4; HRMS (ESI-MS): Calcd. for $C_{19}H_{21}BrO_3S$ (M + Na): 431.0287, Found: 431.0292.

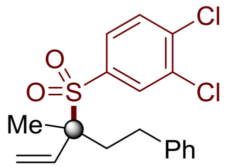

3ap

1,2-dichloro-4-((3-methyl-5-phenylpent-1-en-3-yl)sulfonyl)benzene (3ap) was prepared according to the general procedure from **1a** and **2p**. The crude product was purified by flash column chromatography (Petroleum ether/EtOAc = 50:1) on silica gel to provide the title compound as a white solid in 87% yield (64.2 mg); ^{1}H NMR (400 MHz, $CDCl_3$) δ 7.89 (s, 1H), 7.64–7.58 (m, 2H), 7.31–7.26 (m, 2H), 7.23–7.15 (m, 3H), 6.00 (dd, J = 10.8, 17.5 Hz, 1H), 5.48 (d, J = 10.8 Hz, 1H), 5.16 (d, J = 17.5 Hz, 1H), 2.64–2.50 (m, 2H), 2.25–2.21 (m, 2H), 1.45 (s, 3H); ^{13}C NMR (100 MHz, $CDCl_3$) δ 140.7, 138.8, 135.0, 134.8, 133.3, 132.4, 130.4,

129.7, 128.6, 128.3, 126.3, 121.5, 68.7, 34.4, 30.1, 16.5; HRMS (ESI-MS): Calcd. for $C_{18}H_{18}Cl_2O_2S$ (M + Na): 391.0297, Found: 391.0294.

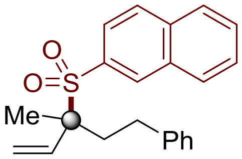

3aq

2-((3-methyl-5-phenylpent-1-en-3-yl)sulfonyl)naphthalene (3aq) was prepared according to the general procedure from **1a** and **2q**. The crude product was purified by flash column chromatography (Petroleum ether/EtOAc = 50:1) on silica gel to provide the title compound as a white solid in 82% yield (57.5 mg); ^{1}H NMR (400 MHz, $CDCl_3$) δ 8.39 (d, *J* = 1.6 Hz, 1H), 7.99–7.92 (m, 2H), 7.80 (dd, *J* = 1.7, 8.6 Hz, 1H), 7.70–7.60 (m, 2H), 7.30–7.26 (m, 3H), 7.21–7.15 (m, 3H), 6.07 (dd, *J* = 10.8, 17.5 Hz, 1H), 5.44 (d, *J* = 10.8 Hz, 1H), 5.13 (d, *J* = 17.5 Hz, 1H), 2.64–2.50 (m, 2H), 2.31–2.26 (m, 2H), 1.49 (s, 3H); ^{13}C NMR (100 MHz, $CDCl_3$) δ 141.0, 135.2, 135.1, 132.6, 132.4, 131.7, 129.5, 129.3, 128.5, 128.4, 128.3, 127.9, 127.4, 126.2, 125.5, 121.0, 68.5, 34.8, 30.2, 16.5; HRMS (ESI-MS): Calcd. for $C_{22}H_{22}O_2S$ (M + Na): 373.1233, Found: 373.1224.

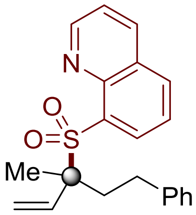

3ar

8-((3-methyl-5-phenylpent-1-en-3-yl)sulfonyl)quinoline (3ar) was prepared according to the general procedure from **1a** and **2r**. The crude product was purified by flash column chromatography (Petroleum ether/EtOAc = 50:1) on silica gel to provide the title compound as a white solid in 72% yield (54.8 mg); ^{1}H NMR (400 MHz, $CDCl_3$) δ 9.13 (d, *J* = 1.2, 2.8 Hz, 1H), 8.47 (d, *J* = 1.2, 4.8 Hz, 1H), 8.22 (d, *J* = 1.2, 4.8 Hz, 1H), 8.09 (d, *J* = 1.2, 4.8 Hz, 1H), 7.65 (t, *J*, 5.2 Hz, 1H), 7.50 (d, *J* = 2.8, 5.5 Hz, 1H), 7.27–7.24 (m, 2H), 7.19–7.14 (m, 3H), 6.18 (dd, *J* = 10.8, 17.5 Hz, 1H), 5.27 (d, *J* = 10.8 Hz, 1H), 5.08 (d, *J* = 17.5 Hz, 1H), 2.66–2.55 (m, 2H), 2.45–2.35 (m, 2H), 1.62 (s, 3H); ^{13}C NMR (100 MHz, $CDCl_3$) δ 151.5, 145.5, 141.3, 136.8, 136.4, 136.0, 135.0, 134.3, 129.0, 128.4, 128.3, 126.0, 125.2, 121.9, 119.8, 70.5, 35.5, 30.2, 17.0; HRMS (ESI-MS): Calcd. for $C_{21}H_{21}NO_2S$ (M + Na): 374.1185, Found: 374.1177.

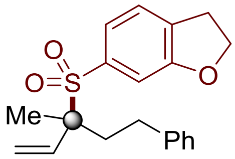

3as

6-((3-methyl-5-phenylpent-1-en-3-yl)sulfonyl)-2,3-dihydrobenzofuran (3as) was prepared according to the general procedure from **1a** and **2s**. The crude product was purified by flash column chromatography (Petroleum ether/EtOAc = 50:1) on silica gel to provide the title compound as a white solid in 92% yield (63.0 mg); ^{1}H NMR (400 MHz, $CDCl_3$) δ 7.59–7.57 (m, 2H), 7.30–7.26 (m, 2H), 7.21–7.15 (m, 3H), 6.82 (d, *J* = 8.2 Hz, 1H), 6.00 (dd, *J* = 10.8, 17.5 Hz, 1H), 5.43 (d, *J* = 10.8 Hz, 1H), 5.16 (d, *J* = 17.5 Hz, 1H), 4.68 (t, *J* = 8.8 Hz, 2H), 3.25 (t, *J* = 8.8 Hz, 2H), 2.61–2.48 (m, 2H), 2.26–2.15 (m, 2H), 1.44 (s, 3H); ^{13}C NMR (100 MHz, $CDCl_3$) δ 164.6, 141.1, 135.4, 132.4, 128.5, 128.3, 127.7, 127.6, 126.6, 126.1, 120.5, 109.0, 72.3, 68.1, 34.8, 30.2, 28.9, 16.4; HRMS (ESI-MS): Calcd. for $C_{20}H_{22}O_3S$ (M + Na): 365.1182, Found: 365.1184.

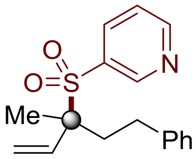

3at

3-((3-methyl-5-phenylpent-1-en-3-yl)sulfonyl)pyridine (3at) was prepared according to the general procedure from **1a** and **2t**. The crude product was purified by flash column chromatography (Petroleum ether/EtOAc = 50:1) on silica gel to provide the title compound as a white solid in 82% yield (49.4 mg); ^{1}H NMR (400 MHz, $CDCl_3$) δ 9.00 (d, *J* = 2.1 Hz, 1H), 8.83 (dd, *J* = 2.1, 4.8 Hz, 1H), 8.10–8.07 (m, 1H), 7.48–7.44 (m, 1H), 7.32–7.27 (m, 2H), 7.23–7.15 (m, 3H), 6.02 (dd, *J* = 10.8, 17.5 Hz, 1H), 5.47 (d, *J* = 10.8 Hz, 1H), 5.13 (d, *J* = 17.5 Hz, 1H), 2.65–2.50 (m, 2H), 2.29–2.22 (m, 2H), 1.46 (s, 3H); ^{13}C NMR (100 MHz, $CDCl_3$) δ 154.0, 151.0, 140.6, 138.3, 134.6, 131.6, 128.5, 128.2, 126.2, 123.1, 68.5, 34.2, 30.0, 16.3; HRMS (ESI-MS): Calcd. for $C_{17}H_{19}NO_2S$ (M + Na): 324.1029, Found: 324.1032.

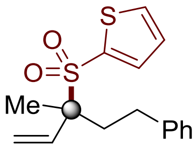

3au

2-((3-methyl-5-phenylpent-1-en-3-yl)sulfonyl)thiophene (3au) was prepared according to the general procedure from **1a** and **2u**. The crude product was purified by flash column chromatography (Petroleum ether/EtOAc = 50:1) on silica gel to provide the title compound as a white solid in 86% yield (52.7 mg); ^{1}H NMR (400 MHz, $CDCl_3$) δ 7.72 (d, *J* = 4.4 Hz, 1H),

7.59 (d, J = 4.4 Hz, 1H), 7.30–7.24 (m, 3H), 7.21–7.11 (m, 3H), 6.04 (dd, J = 10.8, 17.5 Hz, 1H), 5.50 (d, J = 10.8 Hz, 1H), 5.24 (d, J = 17.5 Hz, 1H), 2.65–2.51 (m, 2H), 2.32–2.19 (m, 2H), 1.52 (s, 3H); ^{13}C NMR (100 MHz, $CDCl_3$) δ 140.9, 136.3, 136.0, 134.8, 134.5, 128.5, 128.3, 127.4, 126.2, 121.3, 68.7, 34.8, 30.3, 16.5; HRMS (ESI-MS): Calcd. for $C_{16}H_{18}O_2S_2$ (M + Na): 329.0640, Found: 329.0645.

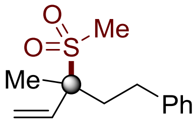

3av

(3-methyl-3-(methylsulfonyl)pent-4-en-1-yl)benzene (3av) was prepared according to the general procedure from **1a** and **2v**. The crude product was purified by flash column chromatography (Petroleum ether/EtOAc = 50:1) on silica gel to provide the title compound as a white solid in 72% yield (34.2 mg); ^{1}H NMR (400 MHz, $CDCl_3$) δ 7.30–7.27 (m, 2H), 7.22–7.17 (m, 3H), 6.11 (dd, J = 10.8, 17.5 Hz, 1H), 5.58 (d, J = 10.8 Hz, 1H), 5.47 (d, J = 17.5 Hz, 1H), 2.77 (s, 3H), 2.66–2.53 (m, 2H), 2.26–2.18 (m, 2H), 1.58 (s, 3H); ^{13}C NMR (100 MHz, $CDCl_3$) δ 140.8, 135.8, 128.5, 128.3, 126.3, 120.6, 67.1, 34.6, 33.8, 29.9, 16.2; HRMS (ESI-MS): Calcd. for $C_{13}H_{18}O_2S$ (M + Na): 261.0920, Found: 261.0924.

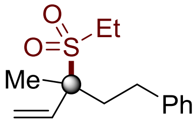

3aw

(3-(ethylsulfonyl)-3-methylpent-4-en-1-yl)benzene (3aw) was prepared according to the general procedure from **1a** and **2w**. The crude product was purified by flash column chromatography (Petroleum ether/EtOAc = 50:1) on silica gel to provide the title compound as a white solid in 78% yield (39.4 mg); ^{1}H NMR (400 MHz, $CDCl_3$) δ 7.31–7.26 (m, 2H), 7.22–7.17 (m, 3H), 6.09 (dd, J = 10.8, 17.5 Hz, 1H), 5.54 (d, J = 10.8 Hz, 1H), 5.44 (d, J = 17.5 Hz, 1H), 2.99–2.90 (m, 2H), 2.66–2.51 (m, 2H), 2.29–2.18 (m, 2H), 1.58 (s, 3H), 1.37 (t, J = 7.5 Hz, 3H); ^{13}C NMR (100 MHz, $CDCl_3$) δ 140.9, 135.8, 128.5, 128.3, 126.2, 120.3, 67.1, 40.7, 34.0, 29.8, 16.1, 5.1; HRMS (ESI-MS): Calcd. for $C_{14}H_{20}O_2S$ (M + Na): 275.1076, Found: 275.1079.

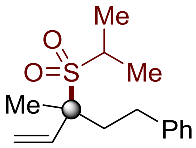

3ax

(3-(isopropylsulfonyl)-3-methylpent-4-en-1-yl)benzene (3ax) was prepared according to the general procedure from **1a** and **2x**. The crude product was purified by flash column chro-

matography (Petroleum ether/EtOAc = 50:1) on silica gel to provide the title compound as a white solid in 82% yield (43.7 mg); ^{1}H NMR (400 MHz, $CDCl_3$) δ 7.29–7.26 (m, 2H), 7.21–7.17 (m, 3H), 6.15 (dd, *J* = 10.8, 17.5 Hz, 1H), 5.50 (d, *J* = 10.8 Hz, 1H), 5.42 (d, *J* = 17.5 Hz, 1H), 3.44–3.37 (m, 1H), 2.63–2.52 (m, 2H), 2.26–2.21 (m, 2H), 1.59 (s, 3H), 1.38–1.35 (m, 6H); ^{13}C NMR (100 MHz, $CDCl_3$) δ 141.1, 136.6, 128.5, 128.4, 126.2, 119.3, 68.6, 50.2, 34.92, 29.8, 17.6, 17.3, 16.7; HRMS (ESI-MS): Calcd. for $C_{15}H_{22}O_2S$ (M + Na): 289.1238, Found: 289.1244.

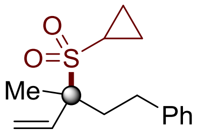

3ay

(3-(cyclopropylsulfonyl)-3-methylpent-4-en-1-yl)benzene (3ay) was prepared according to the general procedure from **1a** and **2y**. The crude product was purified by flash column chromatography (Petroleum ether/EtOAc = 50:1) on silica gel to provide the title compound as a white solid in 78% yield (41.2 mg); ^{1}H NMR (400 MHz, $CDCl_3$) δ 7.30–7.25 (m, 2H), 7.21–7.17 (m, 3H), 6.11 (dd, *J* = 10.8, 17.5 Hz, 1H), 5.54 (d, *J* = 10.8 Hz, 1H), 5.45 (d, *J* = 17.5 Hz, 1H), 2.65–2.51 (m, 2H), 2.39–2.30 (m, 1H), 2.28–2.19 (m, 2H), 1.60 (s, 3H), 1.20–1.17 (m, 2H), 1.03–0.93 (m, 2H); ^{13}C NMR (100 MHz, $CDCl_3$) δ 141.0, 135.7, 128.4, 128.2, 126.1, 120.2, 67.6, 34.5, 29.7, 24.6, 16.2, 5.2, 4.1; HRMS (ESI-MS): Calcd. for $C_{15}H_{20}O_2S$ (M + Na): 287.1076, Found: 287.1082.

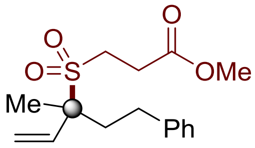

3az

methyl 3-((3-methyl-5-phenylpent-1-en-3-yl)sulfonyl)propanoate (3az) was prepared according to the general procedure from **1a** and **2z**. The crude product was purified by flash column chromatography (Petroleum ether/EtOAc = 50:1) on silica gel to provide the title compound as a white solid in 72% yield (44.7 mg); ^{1}H NMR (400 MHz, $CDCl_3$) δ 7.30–7.27 (m, 2H), 7.22–7.17 (m, 3H), 6.08 (dd, *J* = 10.8, 17.5 Hz, 1H), 5.59 (d, *J* = 10.8 Hz, 1H), 5.48 (d, *J* = 17.5 Hz, 1H), 3.72 (s, 3H), 3.27–3.22 (m, 2H), 2.85 (t, *J* = 5.4 Hz, 2H), 2.65–2.53 (m, 2H), 2.27–2.19 (m, 2H), 1.59 (s, 3H); ^{13}C NMR (100 MHz, $CDCl_3$) δ 171.2, 140.8, 135.3, 128.6, 128.3, 126.3, 121.0, 67.6, 52.3, 41.9, 33.9, 29.8, 25.6, 16.1; HRMS (ESI-MS): Calcd. for $C_{16}H_{22}O_4S$ (M + Na): 333.1131, Found: 333.1134.

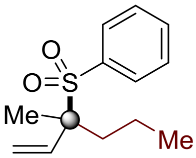

3ba

((3-methylhex-1-en-3-yl)sulfonyl)benzene (3ba) was prepared according to the general procedure from **1b** and **2a**. The crude product was purified by flash column chromatography (Petroleum ether/EtOAc = 60:1) on silica gel to provide the title compound as a colorless oil in 87% yield (41.5 mg); ^{1}H NMR (400 MHz, $CDCl_3$) δ 7.83–7.80 (m, 2H), 7.64–7.61 (m, 1H), 7.53–7.50 (m, 2H), 5.92 (dd, J = 10.7, 17.4 Hz, 1H), 5.34 (d, J = 10.7 Hz, 1H), 5.03 (d, J = 17.4 Hz, 1H), 1.93–1.85 (m, 2H), 1.34 (s, 3H), 1.31–1.21 (m, 2H), 0.94 (t, J = 7.3 Hz, 3H); ^{13}C NMR (100 MHz, $CDCl_3$) δ 136.9, 135.4, 133.5, 130.8, 128.3, 120.3, 68.4, 34.7, 17.1, 16.2, 14.4; HRMS (ESI-MS): Calcd. for $C_{13}H_{18}O_2S$ (M + Na): 261.0920, Found: 261.0926.

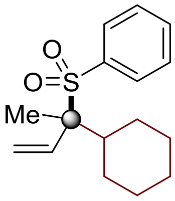

3ca

((2-cyclohexylbut-3-en-2-yl)sulfonyl)benzene (3ca) was prepared according to the general procedure from **1c** and **2a**. The crude product was purified by flash column chromatography (Petroleum ether/EtOAc = 60:1) on silica gel to provide the title compound as a colorless oil in 24% yield (13.4 mg); ^{1}H NMR (400 MHz, $CDCl_3$) δ 7.80–7.78 (m, 2H), 7.63–7.58 (m, 1H), 7.51–7.47 (m, 2H), 6.04 (dd, J = 10.8, 17.5 Hz, 1H), 5.21 (d, J = 10.8 Hz, 1H), 4.85 (d, J = 17.5 Hz, 1H), 2.34–2.18 (m, 2H), 1.92–1.67 (m, 4H), 1.39–1.04 (m, 8H); ^{13}C NMR (100 MHz, $CDCl_3$) δ 136.7, 134.8, 133.2, 130.5, 128.1, 119.2, 72.0, 41.5, 28.8, 28.3, 26.8, 26.5, 26.3, 13.9; HRMS (ESI-MS): Calcd. for $C_{16}H_{22}O_2S$ (M + Na): 301.1238, Found: 301.1246.

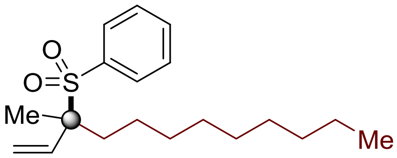

3da

((3-methyldodec-1-en-3-yl)sulfonyl)benzenei (3da) was prepared according to the general procedure from **1d** and **2a**. The crude product was purified by flash column chromatography (Petroleum ether/EtOAc = 60:1) on silica gel to provide the title compound as a colorless oil in 91% yield (58.7 mg); ^{1}H NMR (400 MHz, $CDCl_3$) δ 7.82–7.80 (m, 2H), 7.63–7.61 (m, 1H),

7.53–7.49 (m, 2H), 5.91 (dd, *J* = 10.7, 17.4 Hz, 1H), 5.34 (d, *J* = 10.7 Hz, 1H), 5.02 (d, *J* = 17.4 Hz, 1H), 1.96–1.86 (m, 2H), 1.34 (s, 3H), 1.30–1.25 (m, 14H), 0.88 (t, *J* = 7.3 Hz, 3H); ^{13}C NMR (100 MHz, $CDCl_3$) δ 135.4, 135.3, 133.4, 130.7, 128.2, 120.3, 68.4, 32.5, 31.8, 29.9, 29.4, 29.3, 29.2, 23.7, 22.6, 16.2, 14.1; HRMS (ESI-MS): Calcd. for $C_{19}H_{30}O_2S$ (M + Na): 345.1859, Found: 345.1866.

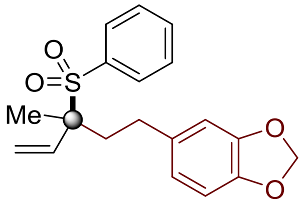

3ea

5-(3-methyl-3-(phenylsulfonyl)pent-4-en-1-yl)benzo[d][1,3]dioxole (3ea) was prepared according to the general procedure from **1e** and **2a**. The crude product was purified by flash column chromatography (Petroleum ether/EtOAc = 60:1) on silica gel to provide the title compound as a white solid in 96% yield (66.1 mg); ^{1}H NMR (400 MHz, $CDCl_3$) δ 7.82–7.79 (m, 2H), 7.65–7.60 (m, 1H), 7.53–7.49 (m, 2H), 6.73–6.58 (m, 3H), 5.98 (dd, *J* = 10.7, 17.4 Hz, 1H), 5.91 (s, 2H), 5.42 (d, *J* = 10.7 Hz, 1H), 5.11 (d, *J* = 17.4 Hz, 1H), 2.54–2.40 (m, 2H), 2.23–2.13 (m, 2H), 1.42 (s, 3H); ^{13}C NMR (100 MHz, $CDCl_3$) δ 147.5, 145.8, 135.0, 134.9, 134.7, 133.6, 130.6, 128.3, 121.0, 120.8, 108.7, 108.2, 100.8, 68.0, 34.9, 29.8, 16.3; HRMS (ESI-MS): Calcd. for $C_{19}H_{20}O_4S$ (M + Na): 367.0980, Found: 367.0984.

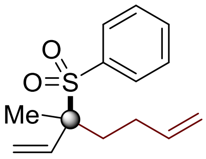

3fa

((3-methylhepta-1,6-dien-3-yl)sulfonyl)benzene (3fa) was prepared according to the general procedure from **1f** and **2a**. The crude product was purified by flash column chromatography (Petroleum ether/EtOAc = 60:1) on silica gel to provide the title compound as a white solid in 85% yield (42.5 mg); ^{1}H NMR (400 MHz, $CDCl_3$) δ 7.82–7.81 (m, 2H), 7.64–7.61 (m, 1H), 7.53–7.50 (m, 2H), 5.93 (dd, *J* = 10.7, 17.4 Hz, 1H), 5.81–5.75 (m, 1H), 5.37 (d, *J* = 10.7 Hz, 1H), 5.07 (d, *J* = 17.4 Hz, 1H), 5.03 (d, *J* = 17.4 Hz, 1H), 4.98 (d, *J* = 10.5 Hz, 1H), 2.06–1.96 (m, 4H), 1.37 (s, 3H); ^{13}C NMR (100 MHz, $CDCl_3$) δ 137.3, 135.2, 135.0, 133.6, 130.7, 128.3, 120.7, 115.3, 68.1, 31.9, 28.0, 16.2; HRMS (ESI-MS): Calcd. for $C_{14}H_{18}O_2S$ (M + Na): 273.0920, Found: 273.0924.

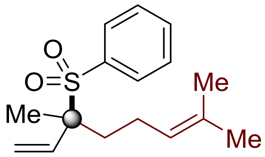

3ga

((3,7-dimethylocta-1,6-dien-3-yl)sulfonyl)benzene (3ga) was prepared according to the general procedure from **1g** and **2a**. The crude product was purified by flash column chromatography (Petroleum ether/EtOAc = 60:1) on silica gel to provide the title compound as a white solid in 92% yield (51.2 mg); ^{1}H NMR (400 MHz, $CDCl_3$) δ 7.82–7.80 (m, 2H), 7.64–7.61 (m, 1H), 7.53–7.50 (m, 2H), 5.92 (dd, *J* = 10.7, 17.4 Hz, 1H), 5.37 (d, *J* = 10.7 Hz, 1H), 5.08–5.05 (m, 2H), 1.96–1.86 (m, 4H), 1.67 (s, 3H), 1.56 (s, 3H), 1.37 (s, 3H); ^{13}C NMR (100 MHz, $CDCl_3$) δ 135.3, 135.2, 133.5, 132.6, 130.8, 128.3, 123.0, 120.5, 68.2, 32.7, 25.6, 22.4, 17.6, 16.2; HRMS (ESI-MS): Calcd. for $C_{16}H_{22}O_2S$ (M + Na): 301.1238, Found: 301.1245.

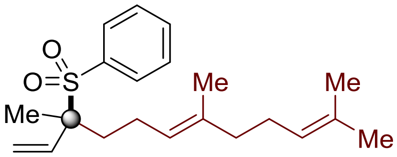

3ha

(*E*)-((3,7,11-trimethyldodeca-1,6,10-trien-3-yl)sulfonyl)benzene (3ha) was prepared according to the general procedure from **1h** and **2a**. The crude product was purified by flash column chromatography (Petroleum ether/EtOAc = 60:1) on silica gel to provide the title compound as a white solid in 86% yield (59.6 mg); ^{1}H NMR (400 MHz, $CDCl_3$) δ 7.83–7.80 (m, 2H), 7.64–7.61 (m, 1H), 7.52–7.50 (m, 2H), 5.93 (dd, *J* = 10.7, 17.4 Hz, 1H), 5.37 (d, *J* = 10.7 Hz, 1H), 5.09–5.05 (m, 3H), 2.07–1.87 (m, 8H), 1.67 (s, 3H), 1.60 (s, 3H),1.56 (s, 3H), 1.37 (s, 3H); ^{13}C NMR (100 MHz, $CDCl_3$) δ 136.9, 136.3, 135.3, 135.2, 133.5, 130.8, 128.3, 124.1, 123.7, 120.5, 68.3, 39.6, 31.9, 26.5, 25.7, 23.3, 22.3, 17.7, 16.2; HRMS (ESI-MS): Calcd. for $C_{21}H_{30}O_2S$ (M + Na): 369.1859, Found: 369.1864.

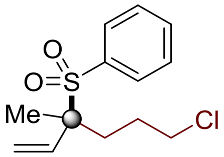

3ia

((6-chloro-3-methylhex-1-en-3-yl)sulfonyl)benzene (3ia) was prepared according to the general procedure from **1i** and **2a**. The crude product was purified by flash column chromatography (Petroleum ether/EtOAc = 60:1) on silica gel to provide the title compound as a white solid in 82% yield (44.7 mg); ^{1}H NMR (400 MHz, $CDCl_3$) δ 7.83–7.81 (m, 2H), 7.65–7.62 (m, 1H), 7.54–7.51 (m, 2H), 5.93 (dd, *J* = 10.7, 17.4 Hz, 1H), 5.37 (d, *J* = 10.7 Hz, 1H), 5.07 (d, *J* = 17.4 Hz, 1H), 3.53 (t, *J* = 6.4 Hz, 2H), 2.11–2.02 (m, 2H), 1.82–1.96 (m, 2H), 1.36 (s, 3H); ^{13}C

NMR (100 MHz, $CDCl_3$) δ 136.9, 134.9, 133.9, 130.7, 128.4, 120.9, 67.7, 44.6, 30.4, 27.2, 16.4; HRMS (ESI-MS): Calcd. for $C_{13}H_{17}ClO_2S$ (M + Na): 295.0535, Found: 295.0530.

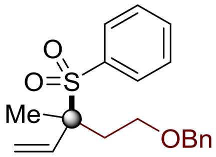

3ja

((5-(benzyloxy)-3-methylpent-1-en-3-yl)sulfonyl)benzene (3ja) was prepared according to the general procedure from **1j** and **2a**. The crude product was purified by flash column chromatography (Petroleum ether/EtOAc = 60:1) on silica gel to provide the title compound as a white solid in 92% yield (60.8 mg); ^{1}H NMR (400 MHz, $CDCl_3$) δ 7.81–7.79 (m, 2H), 7.64–7.60 (m, 1H), 7.52–7.48 (m, 2H), 7.35–7.25 (m, 5H), 5.94 (dd, *J* = 10.7, 17.4 Hz, 1H), 5.36 (d, *J* = 10.7 Hz, 1H), 5.09 (d, *J* = 17.4 Hz, 1H), 4.46 (d, *J* = 12.4 Hz, 1H), 4.42 (d, *J* = 12.4 Hz, 1H), 3.52 (t, *J* = 6.8 Hz, 2H), 2.29–2.22 (m, 2H), 1.42 (s, 3H); ^{13}C NMR (100 MHz, $CDCl_3$) δ 137.9, 134.8, 134.7, 133.6, 130.7, 128.4, 128.3, 127.6, 127.5, 120.6, 72.9, 67.2, 65.9, 32.7, 16.8; HRMS (ESI-MS): Calcd. for $C_{19}H_{22}O_3S$ (M + Na): 353.1182, Found: 353.1177.

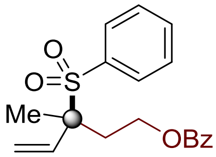

3ka

3-methyl-3-(phenylsulfonyl)pent-4-en-1-yl benzoate (3ka) was prepared according to the general procedure from **1k** and **2a**. The crude product was purified by flash column chromatography (Petroleum ether/EtOAc = 60:1) on silica gel to provide the title compound as a white solid in 94% yield (64.7 mg); ^{1}H NMR (400 MHz, $CDCl_3$) δ 8.01–7.98 (m, 2H), 7.84–7.82 (m, 2H), 7.67–7.63 (m, 1H), 7.59–7.51 (m, 3H), 7.46–7.42 (m, 2H), 6.02 (dd, *J* = 10.7, 17.4 Hz, 1H), 5.39 (d, *J* = 10.7 Hz, 1H), 5.12 (d, *J* = 17.4 Hz, 1H), 4.45–4.31 (m, 2H), 2.52–2.38 (m, 2H), 1.49 (s, 3H); ^{13}C NMR (100 MHz, $CDCl_3$) δ 166.2, 134.6, 134.5, 133.8, 133.1, 130.8, 129.8, 129.5, 128.4, 128.3, 121.0, 67.0, 60.6, 31.8, 16.7; HRMS (ESI-MS): Calcd. for $C_{19}H_{20}O_4S$ (M + Na): 367.0975, Found: 367.0977.

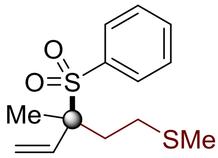

3la

methyl(3-methyl-3-(phenylsulfonyl)pent-4-en-1-yl)sulfane (3la) was prepared according to the general procedure from **1l** and **2a**. The crude product was purified by flash column chromatography (Petroleum ether/EtOAc = 60:1) on silica gel to provide the title compound as a

white solid in 88% yield (47.6 mg); ^{1}H NMR (400 MHz, $CDCl_3$) δ 7.83–7.81 (m, 2H), 7.65–7.63 (m, 1H), 7.54–7.51 (m, 2H), 5.93 (dd, *J* = 10.7, 17.4 Hz, 1H), 5.39 (d, *J* = 10.7 Hz, 1H), 5.09 (d, *J* = 17.4 Hz, 1H), 2.46–2.38 (m, 2H), 2.26–2.18 (m, 2H), 2.11 (s, 3H), 1.38 (s, 3H); ^{13}C NMR (100 MHz, $CDCl_3$) δ 136.9, 134.6, 133.7, 130.7, 128.4, 121.0, 67.8, 33.1, 28.5, 16.4, 15.5; HRMS (ESI-MS): Calcd. for $C_{13}H_{18}O_2S_2$ (M + Na): 293.0640, Found: 293.0539.

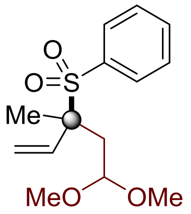

3ma

((5,5-dimethoxy-3-methylpent-1-en-3-yl)sulfonyl)benzene (3ma) was prepared according to the general procedure from **1m** and **2a**. The crude product was purified by flash column chromatography (Petroleum ether/EtOAc = 60:1) on silica gel to provide the title compound as a white solid in 86% yield (48.9 mg); ^{1}H NMR (400 MHz, $CDCl_3$) δ 7.82–7.80 (m, 2H), 7.66–7.62 (m, 1H), 7.54–7.50 (m, 2H), 6.00 (dd, *J* = 10.7, 17.4 Hz, 1H), 5.38 (d, *J* = 10.7 Hz, 1H), 5.10 (d, *J* = 17.4 Hz, 1H), 4.40–4.38 (m, 1H), 3.27 (s, 6H), 2.31–2.18 (m, 2H), 1.43 (s, 3H); ^{13}C NMR (100 MHz, $CDCl_3$) δ 134.9, 134.7, 133.7, 130.8, 128.4, 120.4, 101.5, 66.8, 52.7, 52.4, 35.7, 16.7; HRMS (ESI-MS): Calcd. for $C_{14}H_{20}O_4S$ (M + Na): 307.0975, Found: 307.0973.

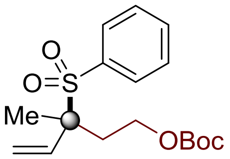

3na

tert-butyl (3-methyl-3-(phenylsulfonyl)pent-4-en-1-yl) carbonate (3na) was prepared according to the general procedure from **1n** and **2a**. The crude product was purified by flash column chromatography (Petroleum ether/EtOAc = 60:1) on silica gel to provide the title compound as a white solid in 87% yield (59.2 mg); ^{1}H NMR (400 MHz, $CDCl_3$) δ 7.83–7.80 (m, 2H), 7.67–7.63 (m, 1H), 7.55–7.51 (m, 2H), 5.94 (dd, *J* = 10.8, 17.5 Hz, 1H), 5.40 (d, *J* = 10.8 Hz, 1H), 5.13 (d, *J* = 17.5 Hz, 1H), 4.15–4.04 (m, 2H), 2.36–2.22 (m, 2H), 1.46 (s, 9H), 1.43 (s, 3H); ^{13}C NMR (100 MHz, $CDCl_3$) δ 153.2, 134.7, 134.3, 133.8, 130.8, 128.5, 121.1, 82.4, 66.7, 62.6, 31.9, 27.7, 16.6; HRMS (ESI-MS): Calcd. for $C_{17}H_{24}O_5S$ (M + Na): 363.1242, Found: 363.1239.

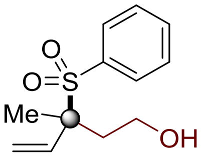

3oa

3-methyl-3-(phenylsulfonyl)pent-4-en-1-ol (3oa) was prepared according to the general procedure from **1o** and **2a**. The crude product was purified by flash column chromatography (Petroleum ether/EtOAc = 60:1) on silica gel to provide the title compound as a white solid in 78% yield (37.5 mg); ^{1}H NMR (400 MHz, $CDCl_3$) δ 7.84–7.80 (m, 2H), 7.67–7.62 (m, 1H), 7.56–7.50 (m, 2H), 6.01 (dd, *J* = 10.7, 17.4 Hz, 1H), 5.37 (d, *J* = 10.7 Hz, 1H), 5.10 (d, *J* = 17.4 Hz, 1H), 3.74 (t, *J* = 6.4 Hz, 2H), 2.30–2.15 (m, 2H), 1.43 (s, 3H); ^{13}C NMR (100 MHz, $CDCl_3$) δ 135.1, 134.1, 132.8, 130.4, 128.7, 121.1, 68.2, 58.3, 34.4, 17.4; HRMS (ESI-MS): Calcd. for $C_{12}H_{16}O_3S$ (M + Na): 263.0718, Found: 263.0724.

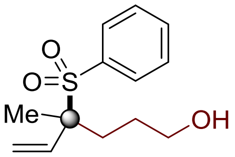

3pa

4-methyl-4-(phenylsulfonyl)hex-5-en-1-ol (3pa) was prepared according to the general procedure from **1p** and **2a**. The crude product was purified by flash column chromatography (Petroleum ether/EtOAc = 60:1) on silica gel to provide the title compound as a white solid in 78% yield (39.7 mg); ^{1}H NMR (400 MHz, $CDCl_3$) δ 7.83–7.80 (m, 2H), 7.64–7.61 (m, 1H), 7.53–7.49 (m, 2H), 5.90 (dd, *J* = 10.7, 17.4 Hz, 1H), 5.37 (d, *J* = 10.7 Hz, 1H), 5.07 (d, *J* = 17.4 Hz, 1H), 3.91–3.78 (m, 1H), 3.65 (brs, 1H), 2.01–1.95 (m, 2H), 1.49–1.43 (m, 2H), 1.42 (s, 3H); ^{13}C NMR (100 MHz, $CDCl_3$) δ 136.9, 135.1, 133.5, 130.7, 128.3, 121.0, 67.5, 60.4, 26.1, 23.3, 14.1; HRMS (ESI-MS): Calcd. for $C_{13}H_{18}O_3S$ (M + Na): 277.0869, Found: 277.0871.

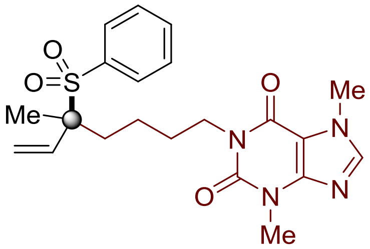

3qa

3,7-dimethyl-1-(5-methyl-5-(phenylsulfonyl)hept-6-en-1-yl)-3,4,5,7-tetrahydro-1H-purine-2,6-dione (3qa) was prepared according to the general procedure from **1q** and **2a**. The crude product was purified by flash column chromatography (Petroleum ether/EtOAc = 60:1) on silica gel to provide the title compound as a white solid in 92% yield (79.0 mg); ^{1}H NMR (400 MHz,

CDCl$_3$) δ 7.81–7.79 (m, 2H), 7.64–7.61 (m, 1H), 7.54–7.49 (m, 3H), 5.90 (dd, *J* = 10.7, 17.4 Hz, 1H), 5.36 (d, *J* = 10.7 Hz, 1H), 5.07 (d, *J* = 17.4 Hz, 1H), 4.00–3.95 (m, 5H), 3.56 (s, 3H), 1.99–1.89 (m, 2H), 1.68–1.63 (m, 2H), 1.36 (s, 3H), 1.36–1.26 (m, 2H); ^{13}C NMR (100 MHz, CDCl$_3$) δ 155.1, 151.3, 148.6, 141.4, 135.2, 135.0, 133.5, 130.7, 128.3, 120.6, 107.5, 68.2, 41.0, 33.5, 32.5, 29.6, 28.3, 21.3, 16.1; HRMS (ESI-MS): Calcd. for $C_{21}H_{26}N_4O_4S$ (M + Na): 453.1567, Found: 453.1570.

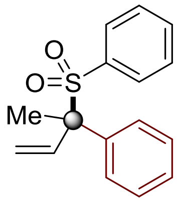

3ra

((2-phenylbut-3-en-2-yl)sulfonyl)benzene (3ra) was prepared according to the general procedure from **1r** and **2a**. The crude product was purified by flash column chromatography (Petroleum ether/EtOAc = 60:1) on silica gel to provide the title compound as a yellow solid in 15% yield (8.2 mg); ^{1}H NMR (400 MHz, CDCl$_3$) δ 7.57–7.26 (m, 10H), 6.70 (dd, *J* = 10.7, 17.4 Hz, 1H), 5.53 (d, *J* = 10.7 Hz, 1H), 5.38 (d, *J* = 17.4 Hz, 1H), 1.86 (s, 3H); ^{13}C NMR (100 MHz, CDCl$_3$) δ 136.1, 135.6, 135.5, 133.9, 130.2, 129.9, 128.9, 128.4, 128.3, 120.2, 71.6, 19.4; Other spectroscopic data for this compound matches with that reported in the literature.[1b]

Formal synthesis of (±)-agelasidine A

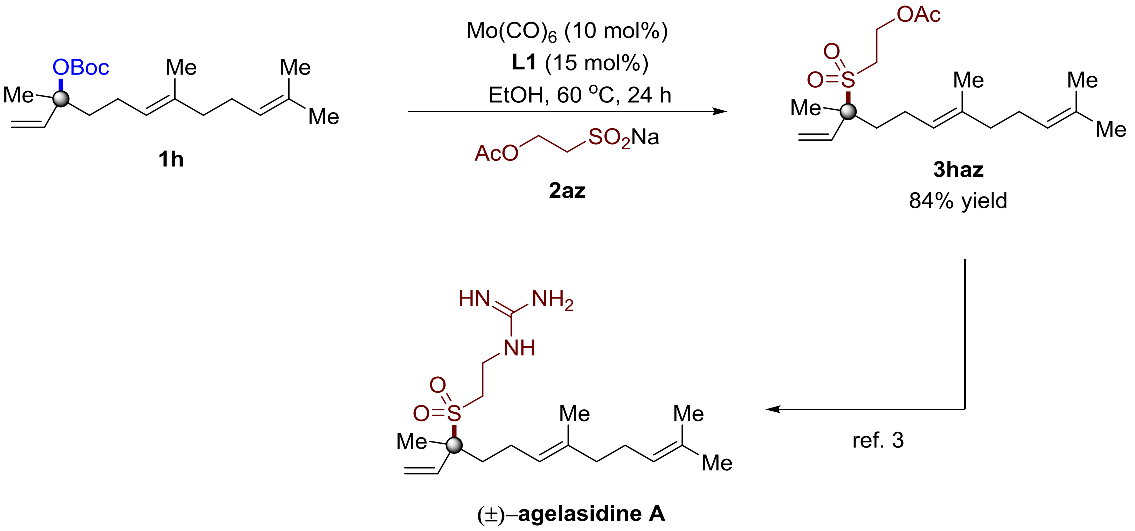

Compound **3haz** was prepared according to the general procedure from **1h** and **2az**. The crude product was purified by flash column chromatography (Petroleum ether/EtOAc = 20:1) on silica gel to provide the title compound as a colorless oil in 84% yield (58.2 mg). ^{1}H NMR (400 MHz, CDCl$_3$) δ 6.01 (dd, *J* = 10.8, 17.6 Hz, 1H), 5.52 (d, *J* = 10.8 Hz, 1H), 5.39 (d, *J* = 17.6 Hz, 1H), 5.11–5.04

(m, 2H), 4.50 (t, J = 6.6 Hz, 1H), 3.25 (t, J = 6.6 Hz, 2H), 2.07–1.87 (m, 11H), 1.68 (s, 3H), 1.60 (s, 3H), 1.58 (s, 3H), 1.51 (s, 3H); ^{13}C NMR (100 MHz, CDCl3) δ 170.7, 136.6, 135.6, 131.6, 124.2, 122.7, 120.8, 68.3, 57.0, 45.7, 31.7, 26.7, 25.8, 22.2, 20.9, 17.8, 16.2, 16.0; The overall spectroscopic data are in complete agreement with assigned structures and consistent with literature.[1b,3] This compound could be directly converted to (±)-**agelasidine A** by following the previously reported literature procedures.[3]

Synthesis of (±)-Sporochnol methyl ether and (±)-Bakuchiol methyl ether

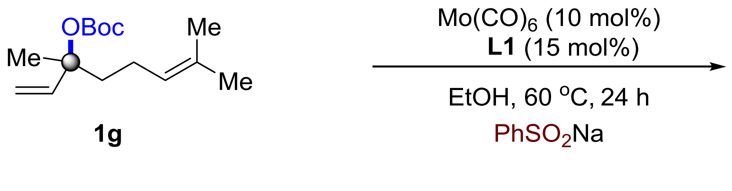

((3,7-dimethylocta-1,6-dien-3-yl)sulfonyl)benzene (3ga): To an oven dried screw-cap reaction tube equipped with a magnetic stir bar, $Mo(CO)_6$ (26.4 mg, 10 mol%), 2,2'-bipyridyne ligand (**L1**) (23.5 mg, 15 mol%), allylic carbonate **1g** (254.4 mg, 1.0 mmol), and sodium benzenesulfinate **2a** (246.23 mg, 0.3 mmol) were added. The reaction tube was sealed with rubber-septum, then evacuated and backfilled with nitrogen. Anhydrous ethanol (0.2 M, 5 mL) were added via syringe. The resulting mixture was stirred at 60 ˚C for 24 hours. The reaction mixture was warm to room temperature and then quenched with water (5 mL). The organic portion was extracted with CH_2Cl_2 and the solvent was removed *in vacuo* with the aid of a rotary evaporator. The obtained residue was purified by flash column chromatography on silica gel to afford the pure tertiary allylic sulfone **3ga** in 85% of isolated yield (236.6 mg) as a colorless oil.

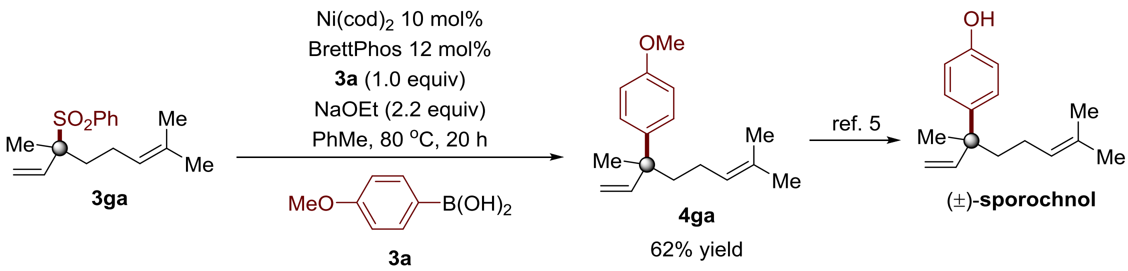

Following the literature procedure[4] with few modifications, describe by C. M. Crudden et. al. as follows: under nitrogen atmosphere, to an oven dried screw-cap reaction tube equipped with a magnetic stir bar, $Ni(cod)_2$ (10 mol %), BrettPhos (12 mol %), and NaOEt (2.2 equiv) were added. Then, ter-

tiary allylic sulfone (**3ga**) (0.2 mmol), boronic acid **3a** (1.0 equiv) and toluene (0.2 M) were added. The reaction tube was capped and sealed, and was stirred at 80 °C for 20 h. The reaction mixture was cooled to room temperature and then diluted with EtOAc and saturated aqueous NH_4Cl (0.2 mL) was added. The mixture was filtered and washed with EtOAc. The obtained residue was concentrated and purified by flash column chromatography on silica gel to afford the pure **4ga** as a light-yellow oil (30.3 mg, 62%). This compound can be converted to (±)-**bakuchiol** by following the reported literature procedures.[5] ^{1}H NMR (400 MHz, $CDCl_3$) δ 7.32 (d, *J* = 8.6 Hz, 2H), 6.85 (d, *J* = 8.6 Hz, 2H), 6.27 (d, *J* = 16.2 Hz, 1H), 6.05 (d, *J* = 16.2 Hz, 1H), 5.87 (dd, *J* = 10.6, 17.4 Hz, 1H), 5.11 (t, *J* = 7.2 Hz, 1H), 5.04 (d, *J* = 10.6 Hz, 1H), 5.01 (d, *J* = 17.4 Hz, 1H), 3.80 (s, 3H), 1.95–1.88 (m, 2H), 1.67 (s, 3H), 1.56 (s, 3H), 1.51–1.45 (m, 2H), 1.22 (s, 3H); ^{13}C NMR (100 MHz, CDC13) δ 158.9, 146.2, 135.9, 131.5, 130.9, 127.3, 126.7, 125.0, 114.0, 112.1, 55.5, 42.7, 41.5, 25.9, 23.5, 23.4, 17.8. The overall spectroscopic data are in complete agreement with assigned structures and consistent with literature.[5]

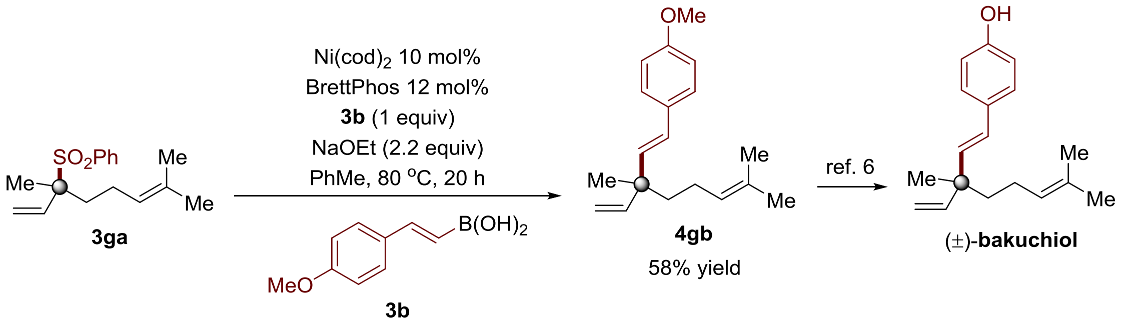

Following the literature procedure[4] with some modifications, describe by C. M. Crudden et. al. as follows: Under nitrogen atmosphere, to an oven dried screw-cap reaction tube equipped with a magnetic stir bar, $Ni(cod)_2$ (10 mol %), BrettPhos (12 mol %), NaOEt (2.2 equiv) were added. Then, tertiary allylic sulfone (**3ga**) (0.2 mmol), boronic acid **3b** (1.0 equiv) and toluene (0.2 M) were added. The reaction tube was capped and sealed, and was stirred at 80 °C for 20 h. The reaction mixture was cooled to room temperature and then diluted with EtOAc and saturated aqueous NH_4Cl (0.2 mL) was added. The mixture was filtered and washed with EtOAc. The obtained residue was concentrated and purified by flash column chromatography on silica gel to afford **4gb** as a colorless oil (31.4 mg, 58%). This compound can be converted to (±)-**sporochnol** by following the reported literature.[6] ^{1}H NMR (400 MHz, $CDCl_3$) δ 7.24 (d, *J* = 8.4 Hz, 2H), 6.84 (d, *J* = 8.4 Hz, 2H), 6.03 (dd, *J* = 10.6, 17.4 Hz, 1H), 5.08–5.00 (m, 3H), 3.79 (s, 3H), 1.90–1.69 (m, 4H), 1.67 (s, 3H), 1.54 (s, 3H), 1.37 (s, 3H); ^{13}C NMR (100 MHz, CDC13) δ 157.5, 147.2, 139.5, 131.3, 127.6, 124.7, 113.4, 111.4,

55.2, 43.7, 41.1, 25.6, 25.1, 23.3, 17.6. The overall spectroscopic data are in complete agreement with assigned structures and consistent with literature.[6]

Synthesis of Mo-complex [Mo(bpy)$(CO)_4$]

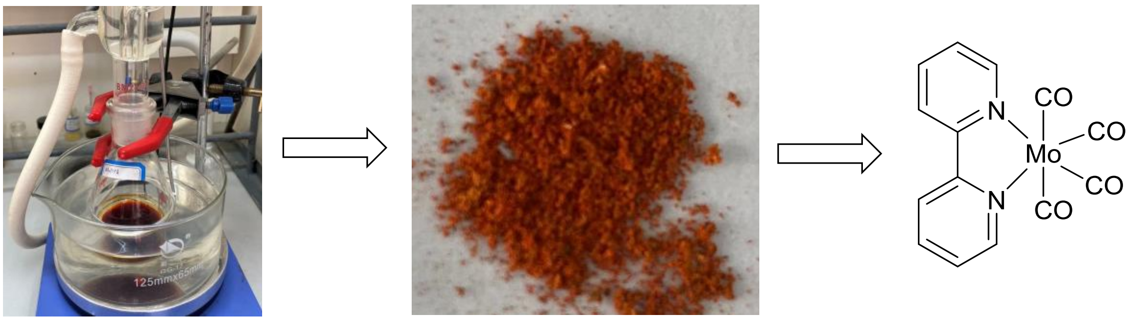

during stiring (A) **after filtration (B)** **Mo(bpy)$(CO)_4$**

In a 100 mL round-bottom flask equipped with a magnetic stir bar, a mixture of $Mo(CO)_6$ (1.0 mmol, 264.0 mg) and 2,2'-bipyridyne (**L1**) (1.0 mmol, 156.2 mg) in anhydrous THF were stirred at 60 °C for 12 hours under nitrogen atmosphere. After few minutes, the solution turned orange and slowly becoming dark red (picture A, left). After 12 h, the reaction mixture was cool to room temperature, filtered and washed with anhydrous hexane. The upper solid portion was scratched with spatula and collected as a dark red crystalline solid (picture B, middle) affording Mo(bpy)$(CO)_4$ complex (262.2 mg, 72% yield). **^{1}H NMR** (400 MHz, $CDCl_3$) δ 9.17–9.15 (m, 1H), 8.12 (d, *J* = 8.0 Hz, 1H), 7.94 (ddd, *J* = 1.6, 7.8, 15.7 Hz, 1H), 7.42–7.38 (m, 1H); **^{13}C NMR** (100 MHz, CDCl3) δ 204.7, 154.5, 153.1, 137.2, 125.2, 121.9; Other spectroscopic data for this compound matches with that reported in the literature.[7]

Preliminary Mechanistic Experiments

(1) Mechanistic experiments with Mo(bpy)$(CO)_4$

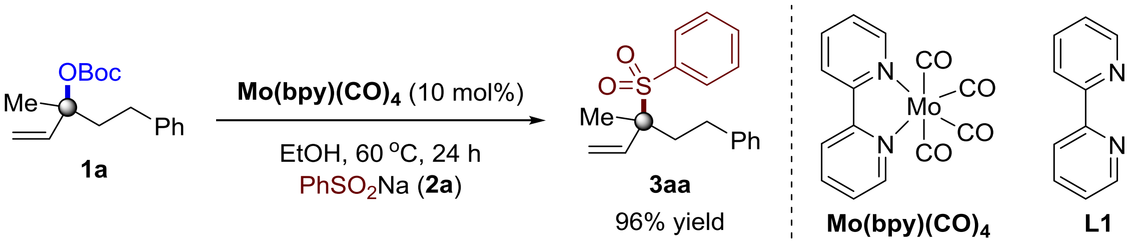

- **Without L1.** Following the standard procedure, to an oven dried screw-cap reaction tube equipped with a magnetic stir bar, Mo(bpy)$(CO)_4$ (7.3 mg, 10 mol%), allylic carbonate **1a** (55.28 mg, 0.2 mmol), and sodium benzenesulfinate **2a** (49.25 mg, 0.3 mmol) were added. The reaction tube was

sealed with rubber-septum, then evacuated and backfilled with nitrogen. Anhydrous ethanol (0.2 M, 1 mL) were added via syringe. The resulting mixture was stirred at 60 ˚C for 24 hours. Interestingly, the reaction proceeded smoothly to provide the title compound **3aa** in 96% of isolated yield.

- **With L1.** Following the standard procedure, to an oven dried screw-cap reaction tube equipped with a magnetic stir bar, Mo(bpy)$(CO)_4$ (7.3 mg, 10 mol%), **L1** (4.7 mg, 15 mmol), allylic carbonate **1a** (55.28 mg, 0.2 mmol), and sodium benzenesulfinate **2a** (49.25 mg, 0.3 mmol) were added. The reaction tube was sealed with rubber-septum, then evacuated and backfilled with nitrogen. Anhydrous ethanol (0.2 M, 1 mL) were added via syringe. The resulting mixture was stirred at 60 ˚C for 24 hours afforded **3aa** in 89% of isolated yield.

(2) Mechanistic experiments with $Mo(CO)_6$

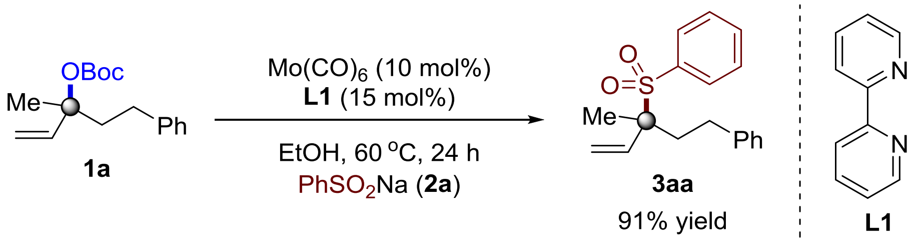

- **Without L1.** Following the standard procedure, using allylic carbonate **1a** (55.28 mg, 0.2 mmol), and sodium benzenesulfinate **2a** (49.25 mg, 0.3 mmol), and $Mo(CO)_6$ (5.3 mg, 10 mol%), did not provide any conversion to **3aa** as judged by ^{1}H NMR analysis.

- **With L1.** Following the standard procedure, using allylic carbonate **1a** (55.28 mg, 0.2 mmol), and sodium benzenesulfinate **2a** (49.25 mg, 0.3 mmol), and $Mo(CO)_6$ (5.3 mg, 10 mol%), **L1** (4.7 mg, 15 mmol) afforded **3aa** in 91% of isolated yield. A small decline in yield of **3aa** under [$Mo(CO)_6$]/**L1** catalyst system, thus providing evidence and implicit that a [Mo(bpy)$(CO)_4$] complex is likely the active catalyst species in this allylic sulfonylation reaction.

References:

1. (a) Zhang, P.; Le, H.; Kyne, R. E.; Morken, J. P. *J. Am. Chem. Soc.* **2011**, *133*, 9716; (b) Cai, A.; Kleij, A. W. *Angew. Chem. Int. Ed*. **2019**, *58*, 14944; (c) Guo, W.; Cai, A.; Xie, J.; Kleij. A. W. *Angew. Chem. Int. Ed*. **2017**, *56*, 11797; (d) Trost, B. M.; Malhotra, S.; Chan, W. H. *J. Am. Chem. Soc*. **2011**, *133*, 7328.

2. Du, B.; Qian, P.; Wang, Y.; Mei, H.; Han, J.; Pan, Y. *Org. Lett.* **2016**, *18*, 4144.

3. Yang, X.-H.; Davison, R. T.; Nie, S.-Z.; Cruz, F. A.; McGinnis, T. M.; Dong, V. M. *J. Am. Chem. Soc*. **2019**, *141*, 3006.

4. Arika, Z. T.; Maekawa, Y.; Nambo, M.; Crudden, C. M. *J. Am. Chem. Soc*., **2018**, *140*, 78.

5. (a) Li, Y.; Han, J.; Luo, H.; An, Q.; Cao, X.-P.; Li, B. *Org. Lett*. **2019**, *21*, 6050; (b) Sonawane, R. P.; Jheengut, V.; Rabalakos, C.; Larouche-Gauthier, R.; Scott, H. K.; Aggarwal, V. K. *Angew. Chem. Int. Ed*. **2011**, *50*, 3760.

6. (a) Majeed, R.; Reddy, M. V.; Chinthakindi, P. K.; Sangwan, P. L.; Hamid, A.; Chashoo, G.; Saxena, A. K.; Koul, S. *Eur. J. Med. Chem*. **2012**, *49*, 55; (b) Chakrabarty, S.; Takacs, J. M. *J. Am. Chem. Soc*. **2017**, *139*, 6066; (c) Gao, F.; McGrath, K. P.; Lee, Y.; Hoveyda, A. H. *J. Am. Chem. Soc*. **2010**, *132*, 14315.

7. (a) Birdwhistell, K. R.; Schulz, B. E.; Dizon, P. M. *Inorg. Chem. Commun*. **2012**, *26*, 69; (b) Neri, G.; Donaldson, P. M.; Cowan, A. J. *J. Am. Chem. Soc*. **2017**, *139*, 13791.

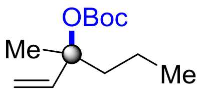

1b

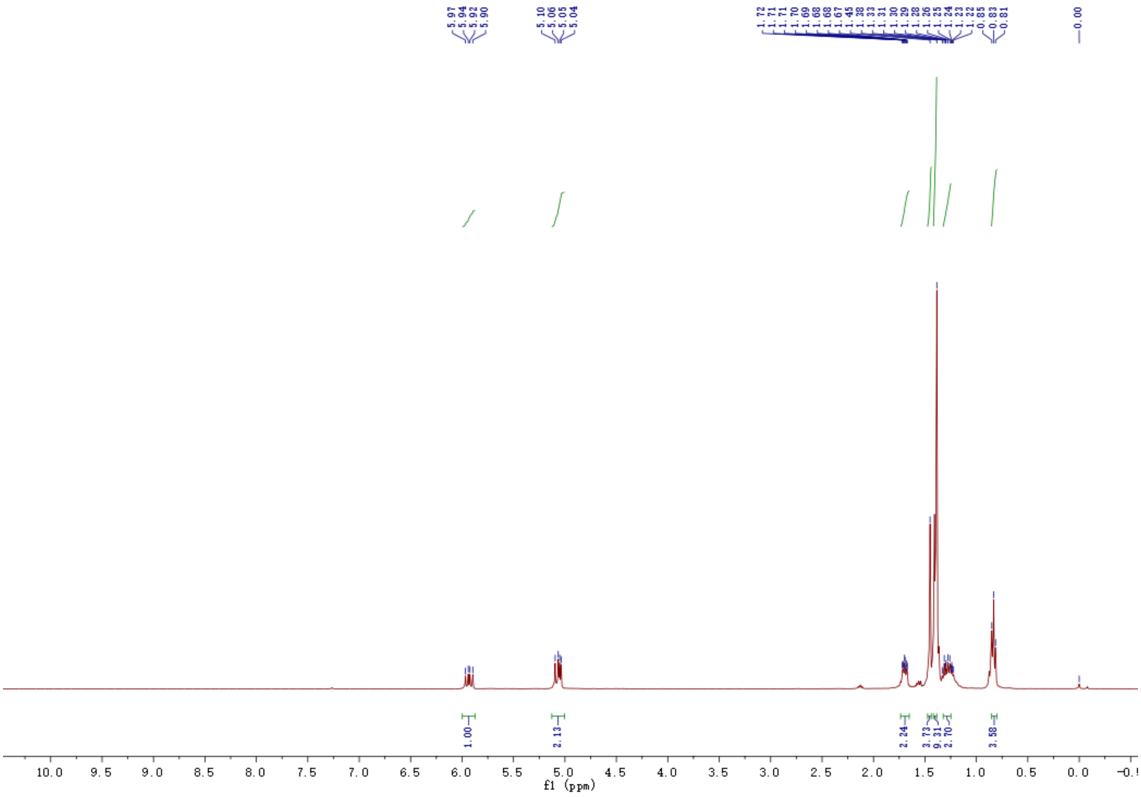

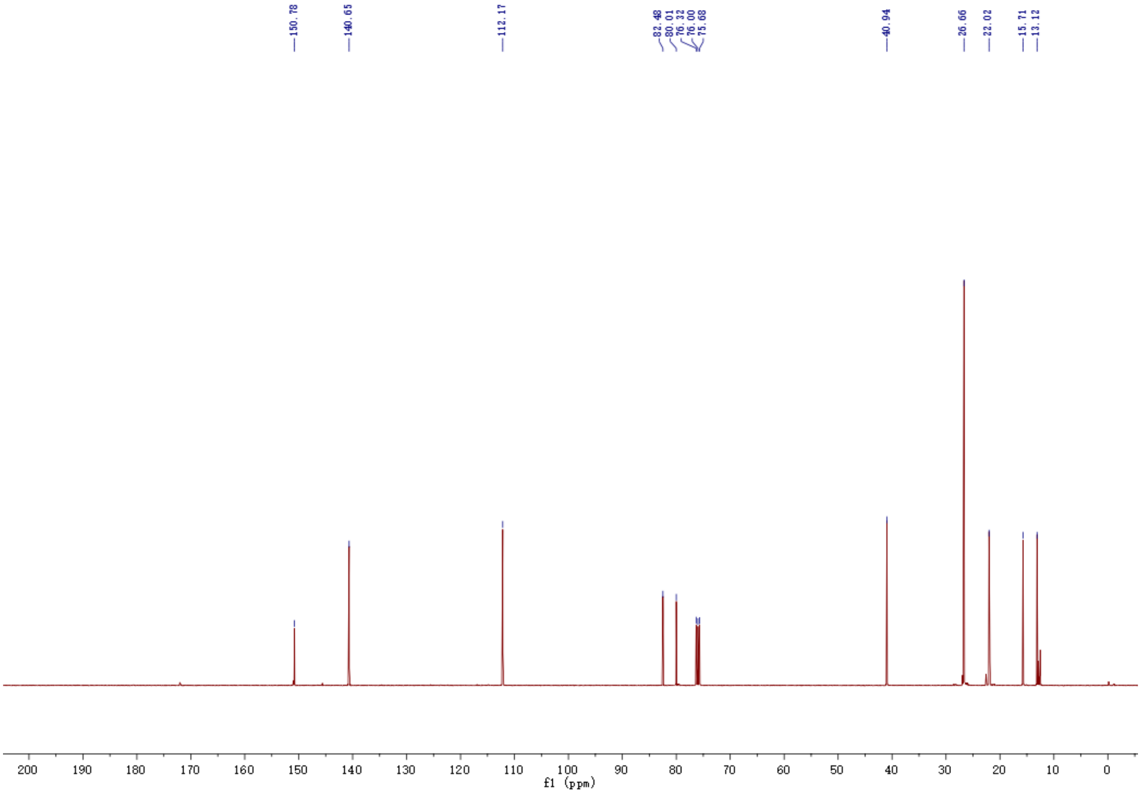

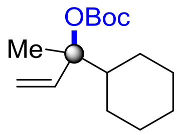

1c

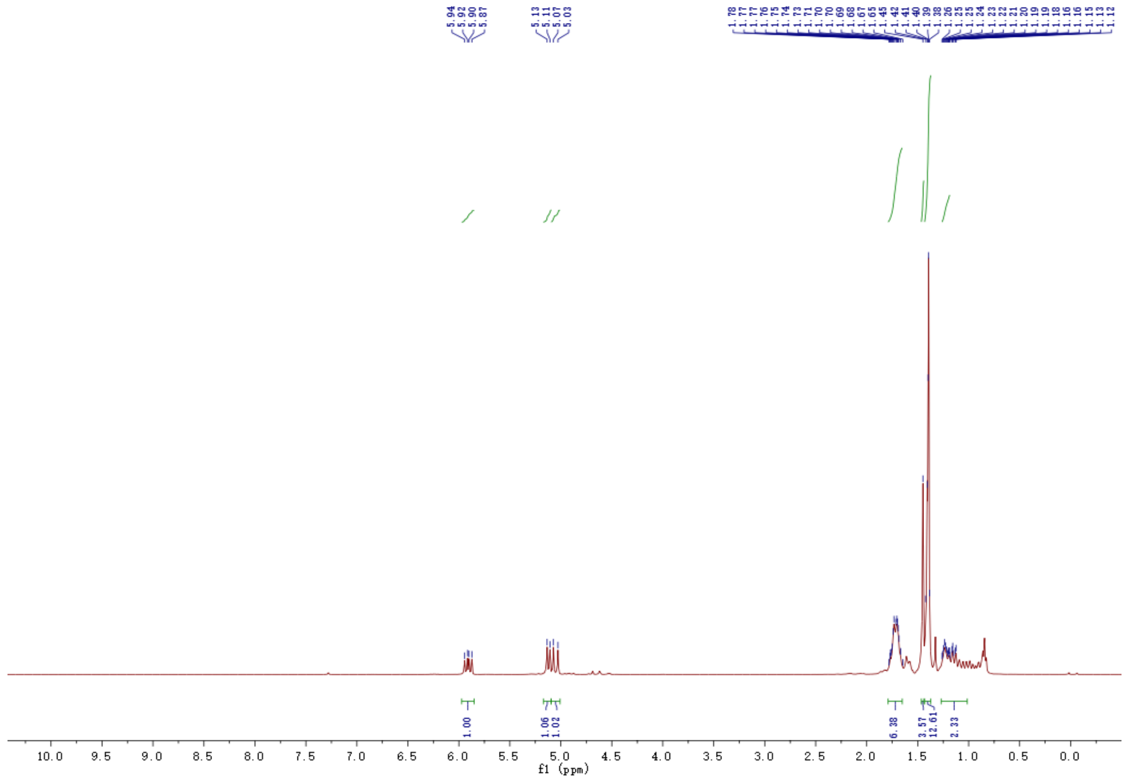

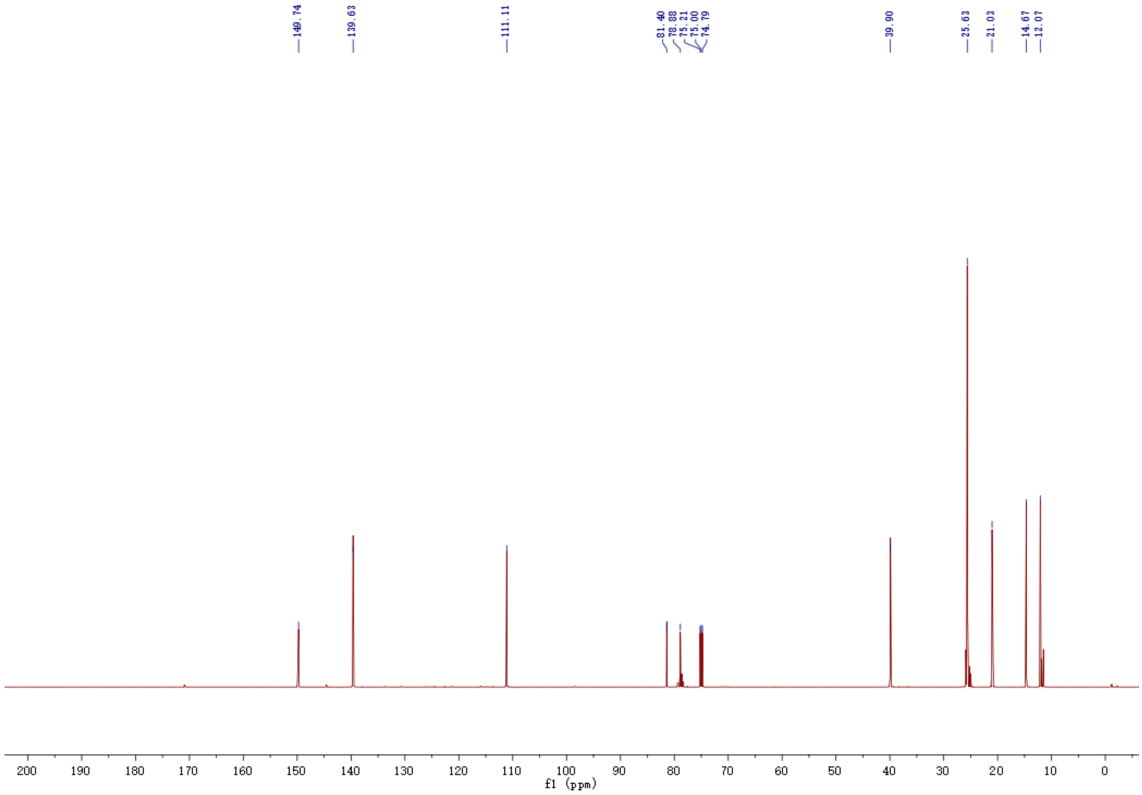

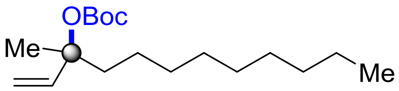
OBoc
Me
Me

1d

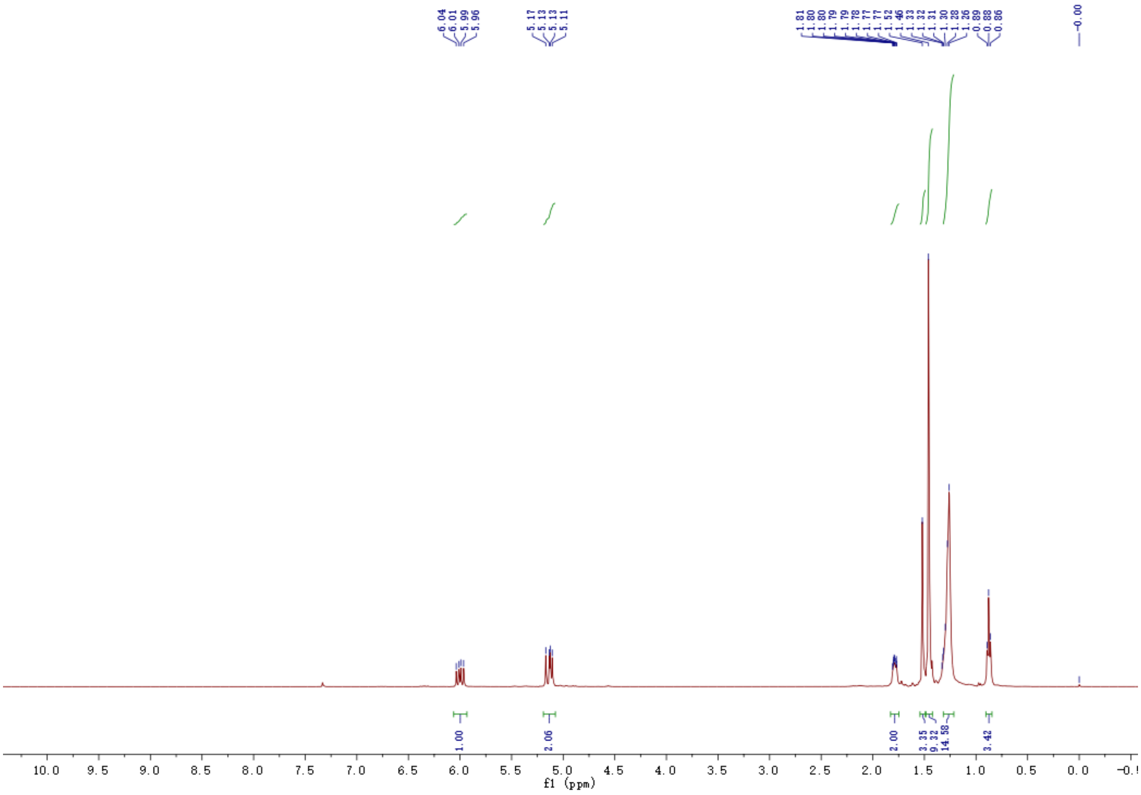
6.04
6.01
5.99
5.96
5.17
5.13
5.13
5.11
1.81
1.80
1.80
1.79
1.78
1.77
1.77
1.52
1.46
1.33
1.32
1.31
1.30
1.28
1.26
0.89
0.88
0.86
-0.00
1.00
2.06
2.00
3.35
9.32
14.58
3.42
f1 (ppm)

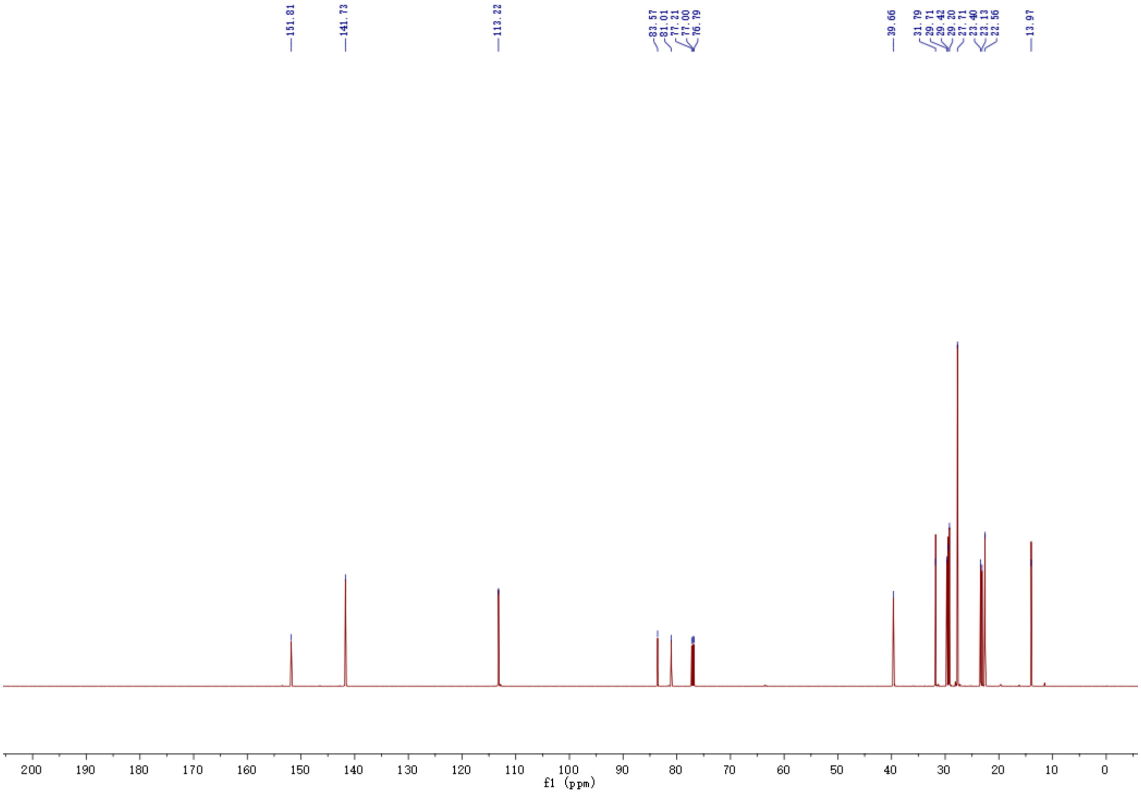
151.81
141.73
113.22
83.57
81.01
77.21
77.00
76.79
39.66
31.79
29.71
29.42
29.20
27.71
23.40
23.13
22.56
13.97
f1 (ppm)

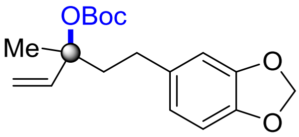

1e

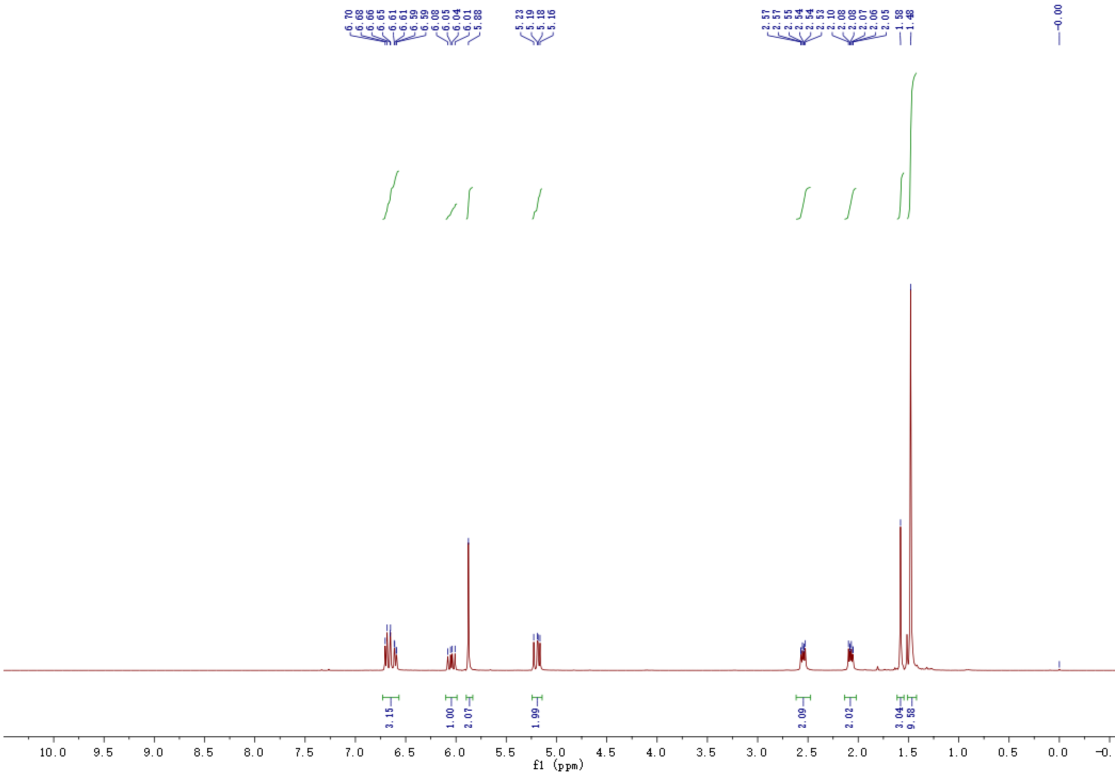

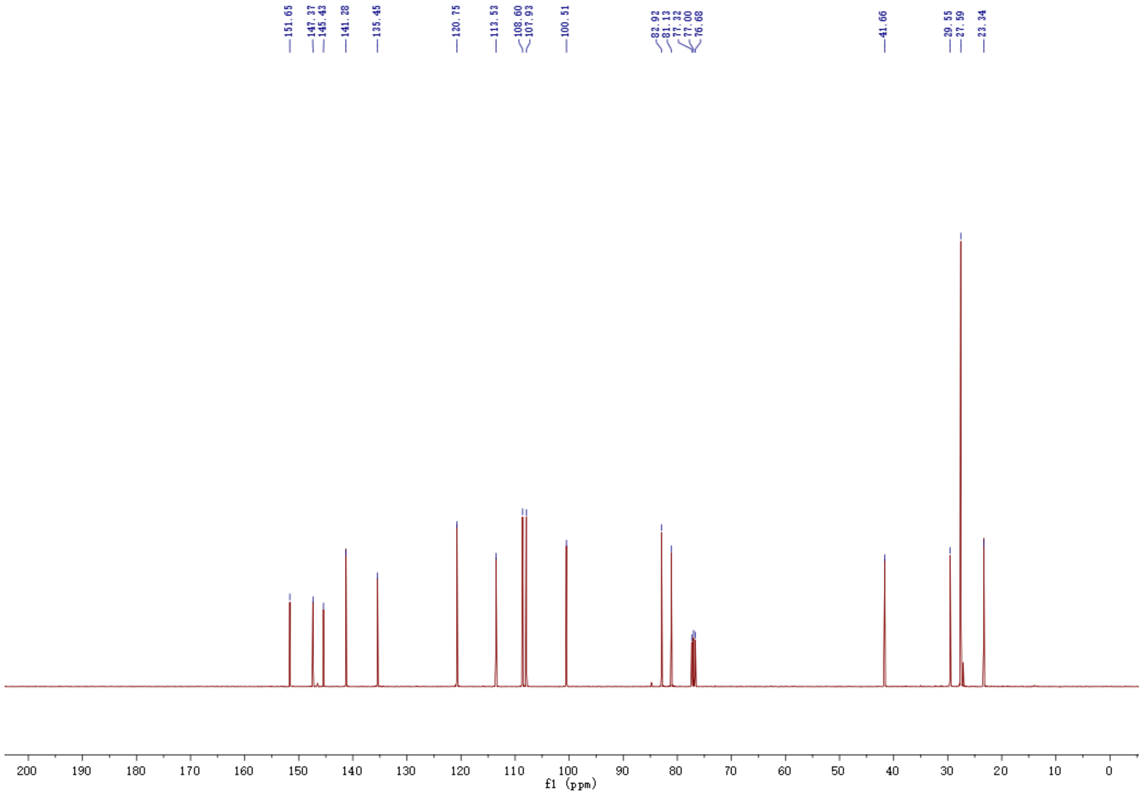

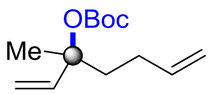
OBoc
Me

1f

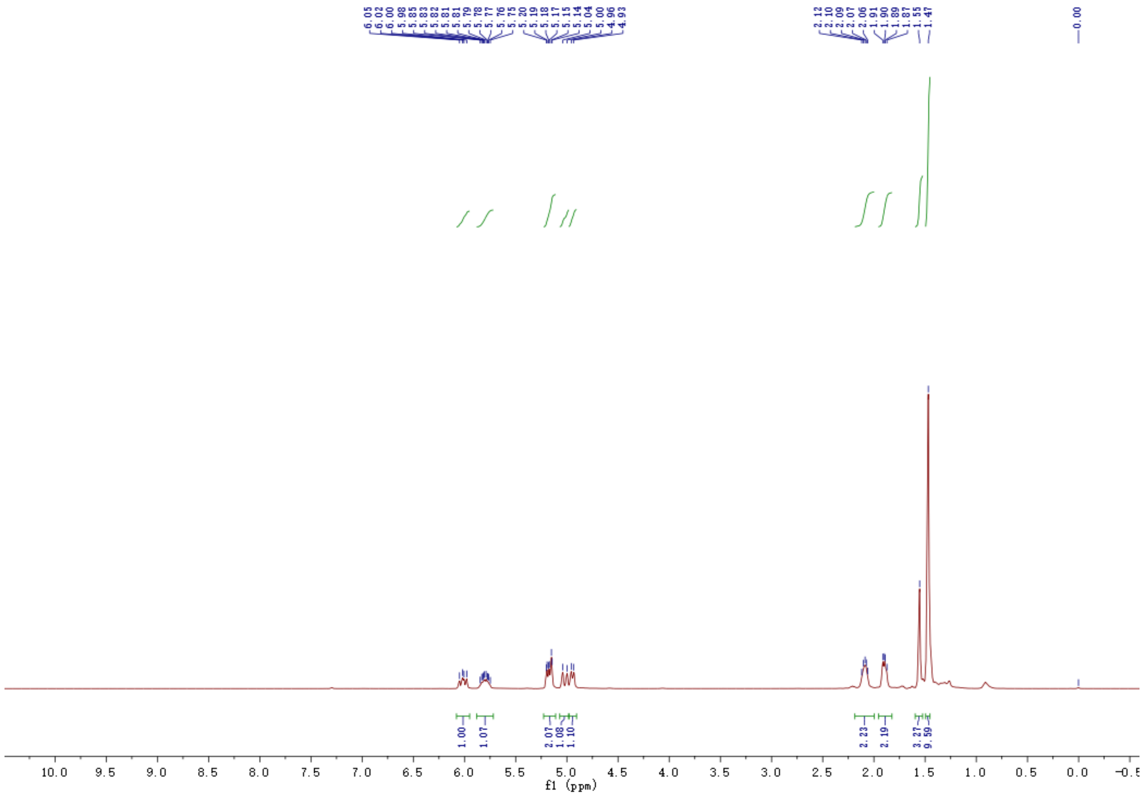
6.05
6.02
6.00
5.98
5.85
5.83
5.82
5.81
5.81
5.79
5.78
5.77
5.76
5.75
5.20
5.19
5.18
5.17
5.15
5.14
5.04
5.00
4.96
4.93
2.12
2.10
2.09
2.07
2.06
1.91
1.90
1.89
1.87
1.55
1.47
0.00
1.00
1.07
2.07
1.08
1.10
2.23
2.19
3.27
9.59
f1 (ppm)

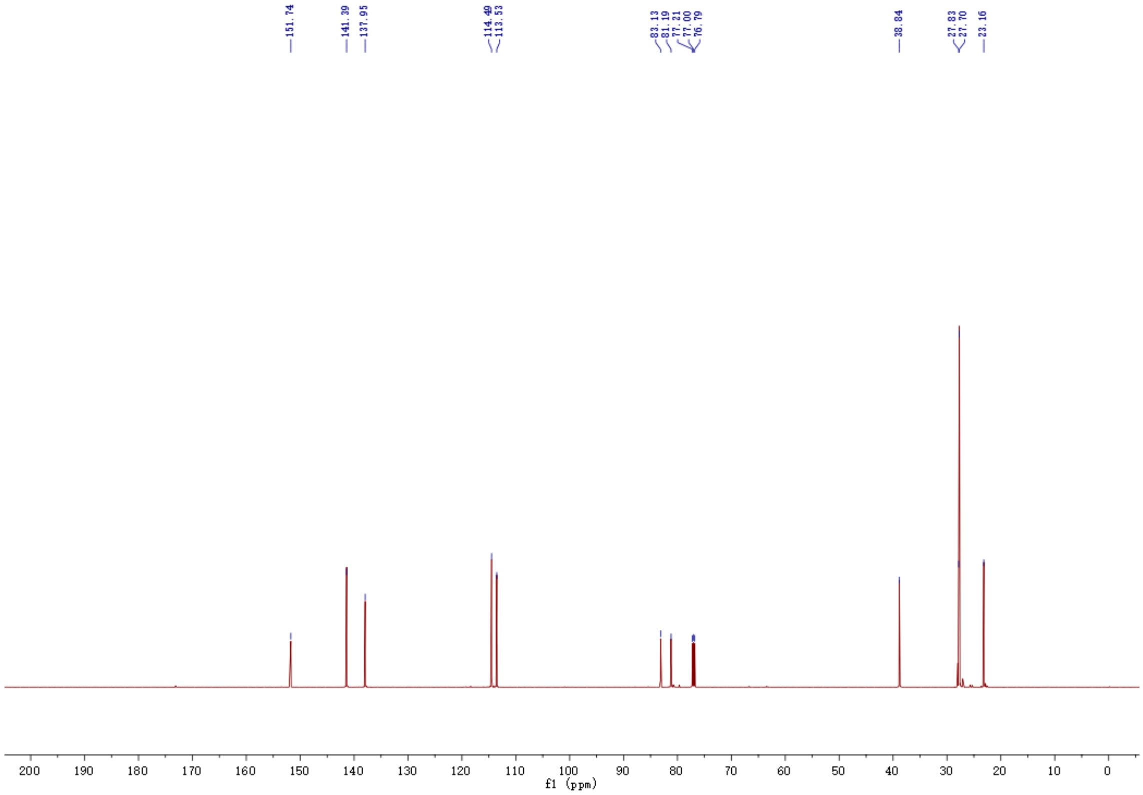
151.74
141.39
137.95
114.49
113.53
83.13
81.19
77.21
77.00
76.79
38.84
27.83
27.70
23.16
f1 (ppm)

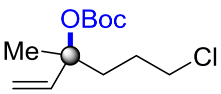

1i

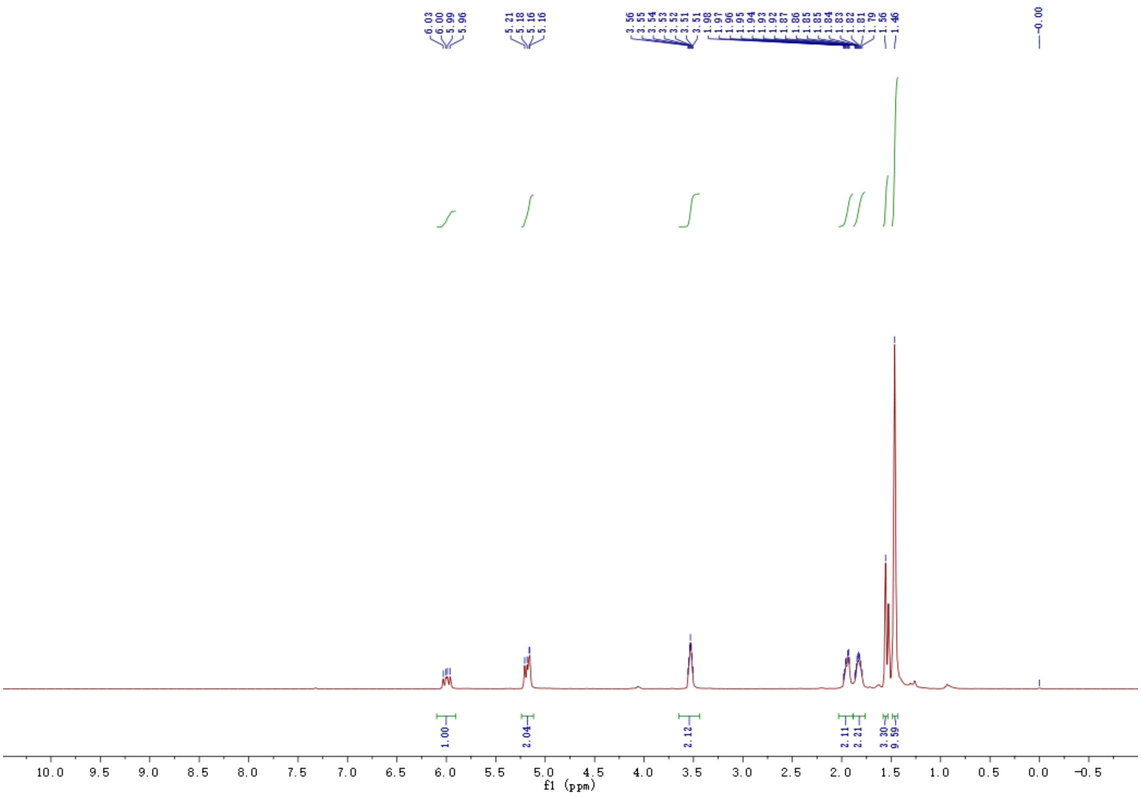

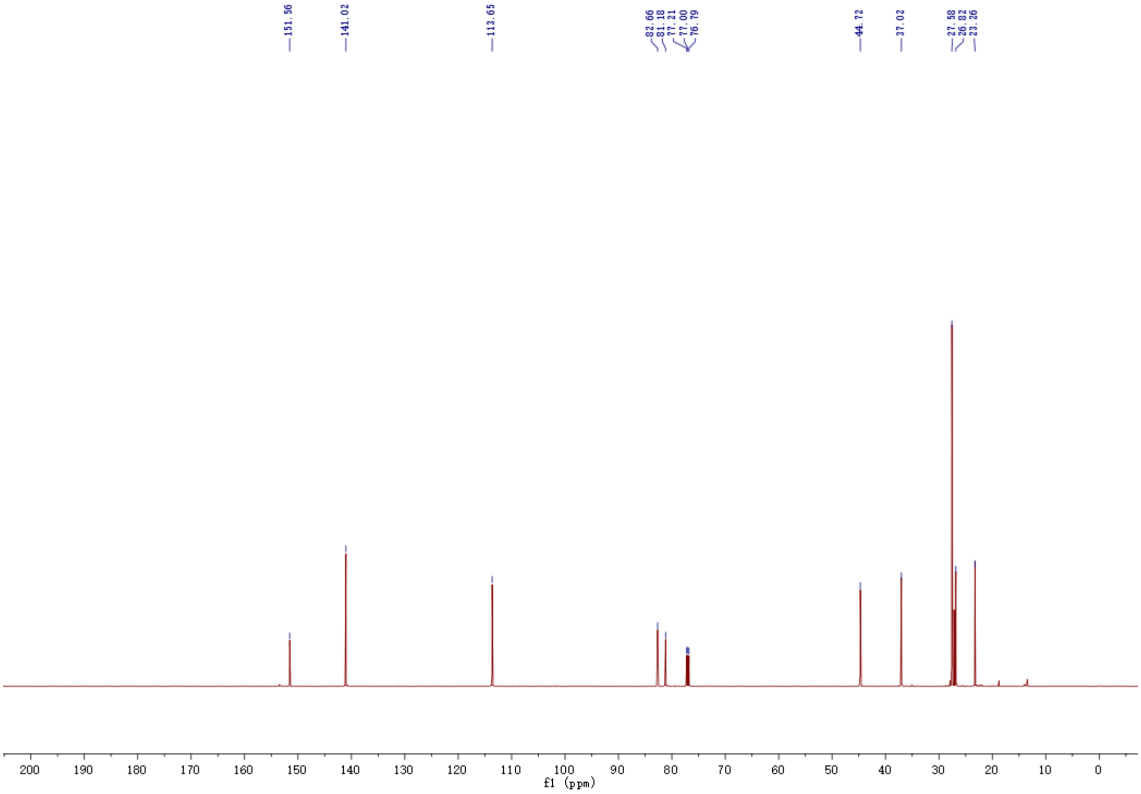

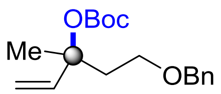

1j

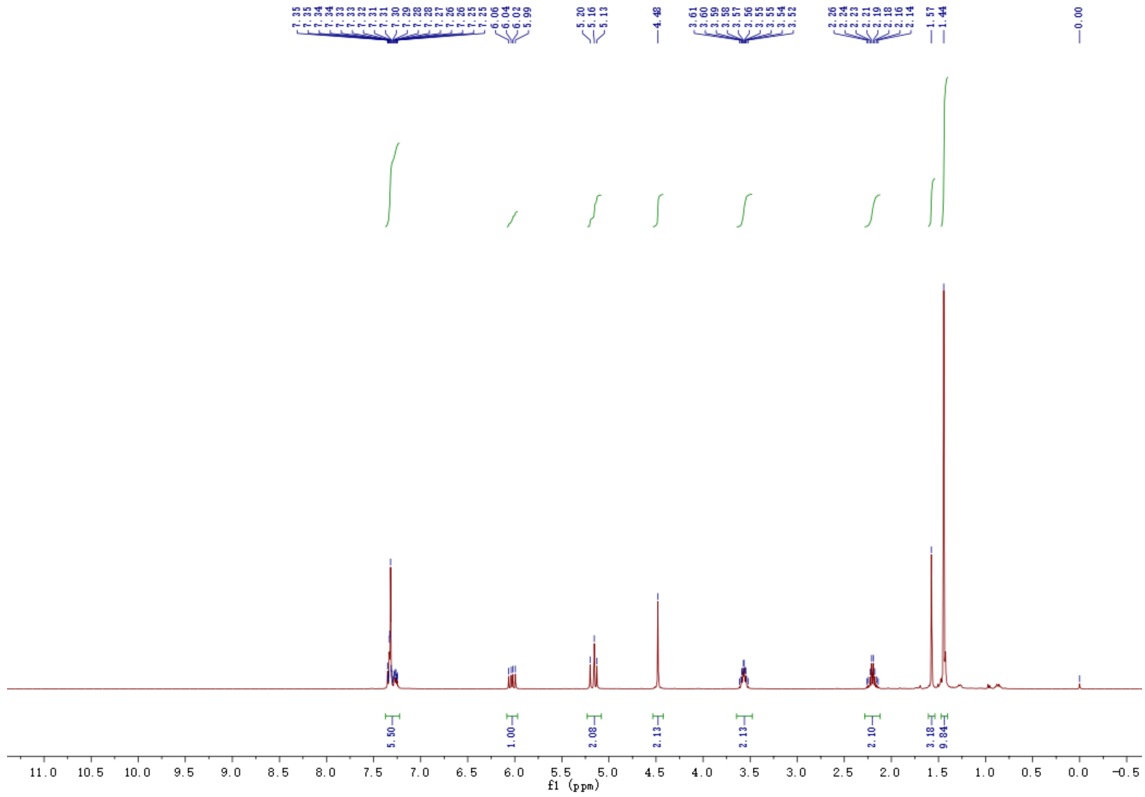

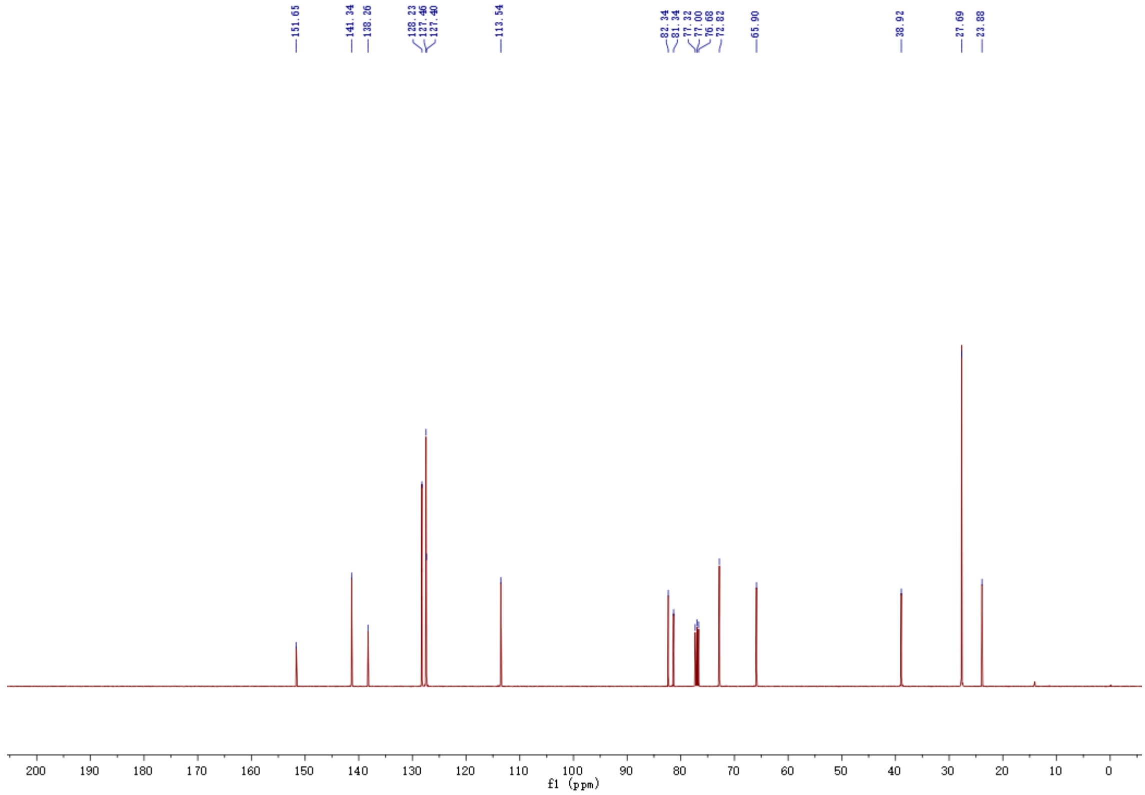

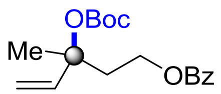
OBoc
Me
OBz

1k

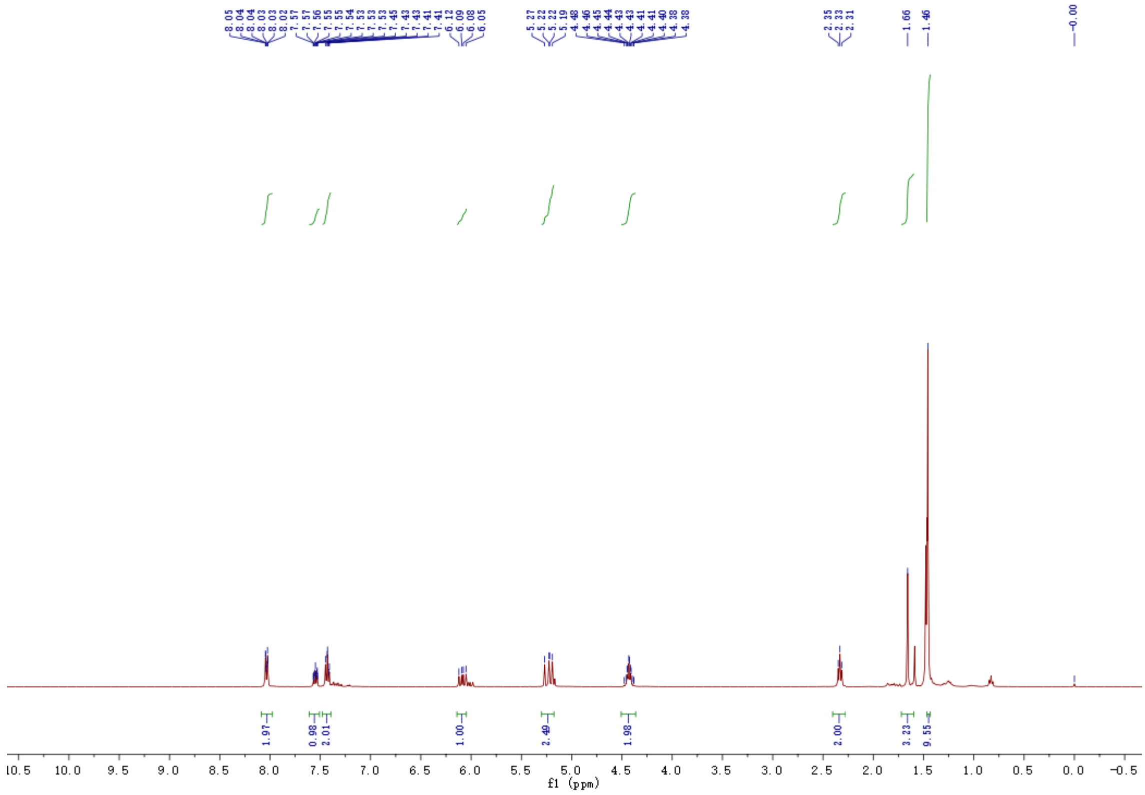
8.05 8.04 8.04 8.03 8.03 8.02 7.57 7.56 7.55 7.55 7.54 7.53 7.53 7.53 7.45 7.43 7.43 7.41 7.41 6.12 6.09 6.08 6.05
5.27 5.22 5.22 5.19 4.48 4.46 4.45 4.44 4.43 4.43 4.41 4.41 4.40 4.38 4.38
2.35 2.33 2.31
1.66
1.46
0.00
1.97
0.98
2.01
1.00
2.49
1.98
2.00
3.23
9.55
f1 (ppm)

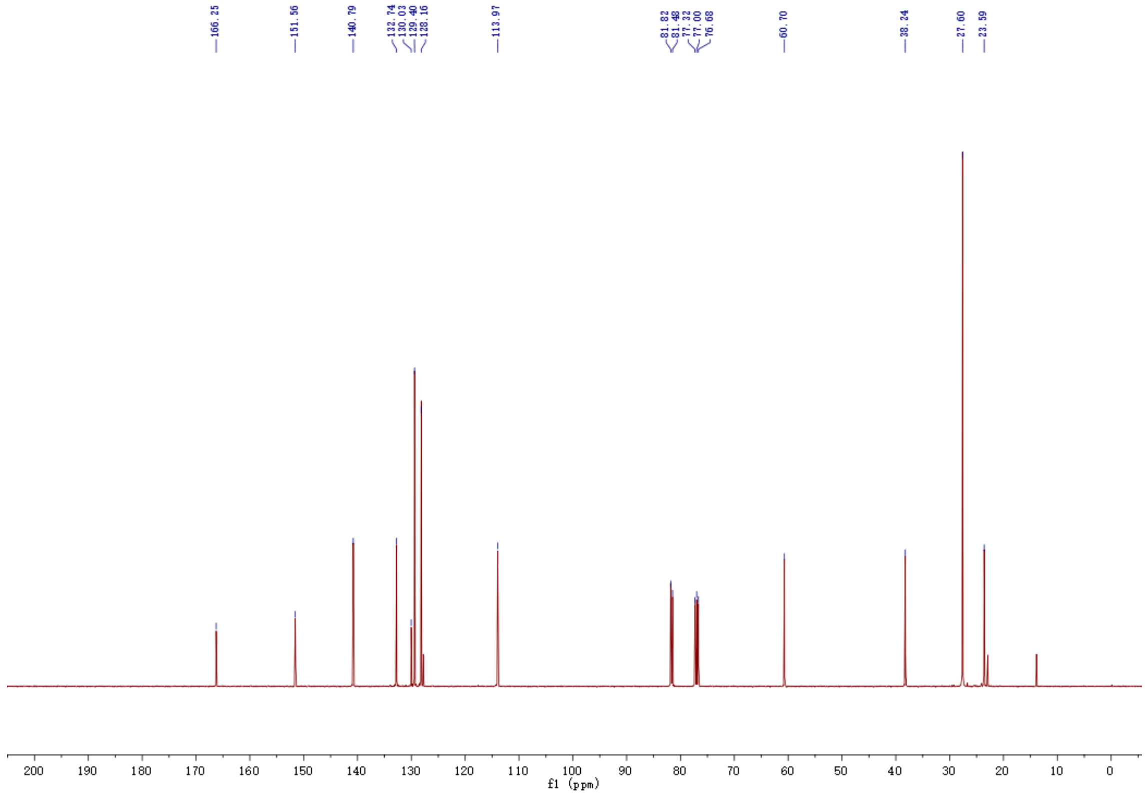
166.25
151.56
140.79
132.74 130.03 129.40 128.16
113.97
81.82 81.48 77.32 77.00 76.68
60.70
38.24
27.60
23.59
f1 (ppm)

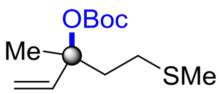

1l

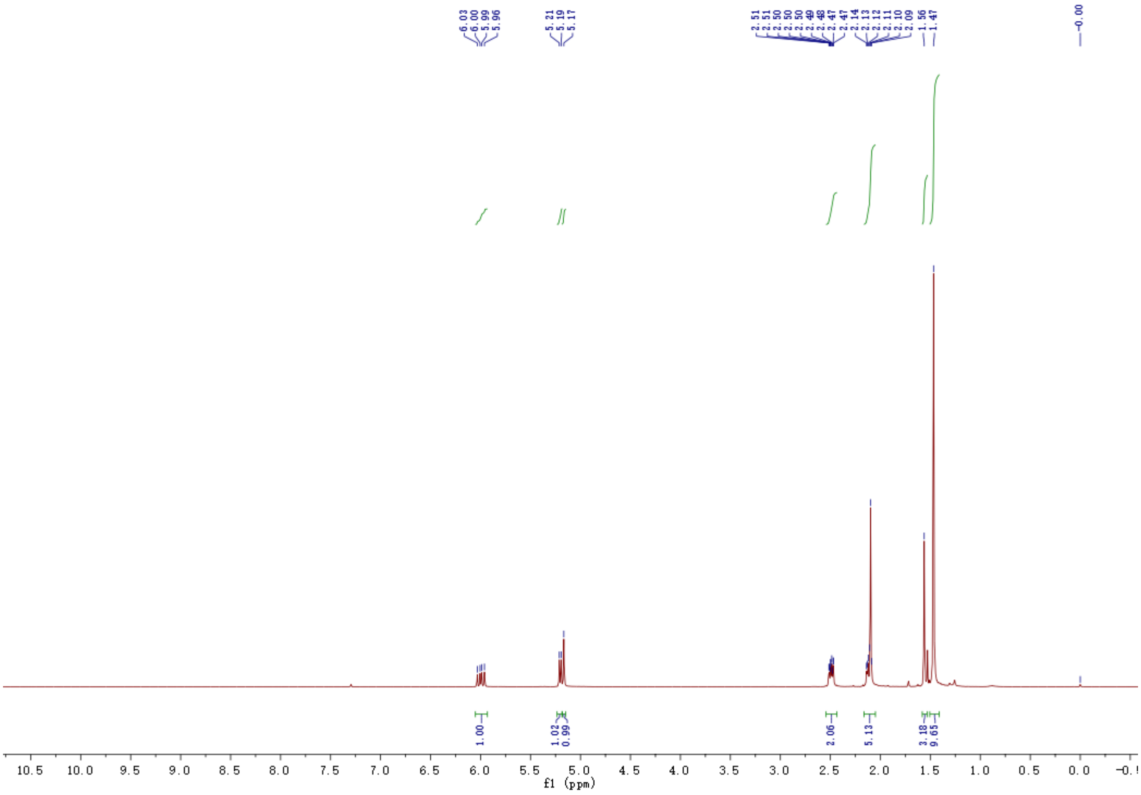
6.03
6.00
5.99
5.96
5.21
5.19
5.17
2.51
2.51
2.50
2.50
2.49
2.48
2.47
2.47
2.14
2.13
2.12
2.11
2.10
2.09
1.56
1.47
0.00
1.00
1.02
0.99
2.06
5.13
3.18
9.65
f1 (ppm)

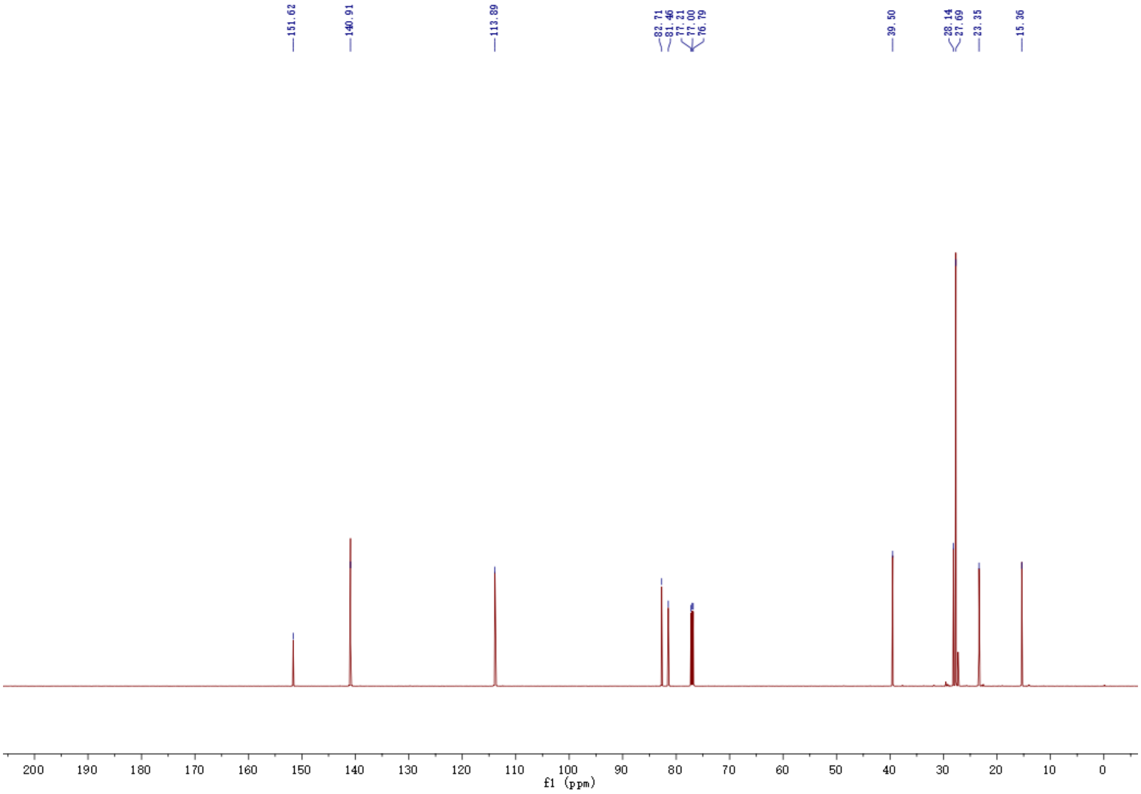
151.62
140.91
113.89
82.71
81.46
77.21
77.00
76.79
39.50
28.14
27.69
23.35
15.36
f1 (ppm)

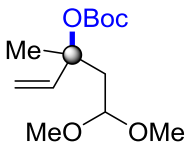

1m

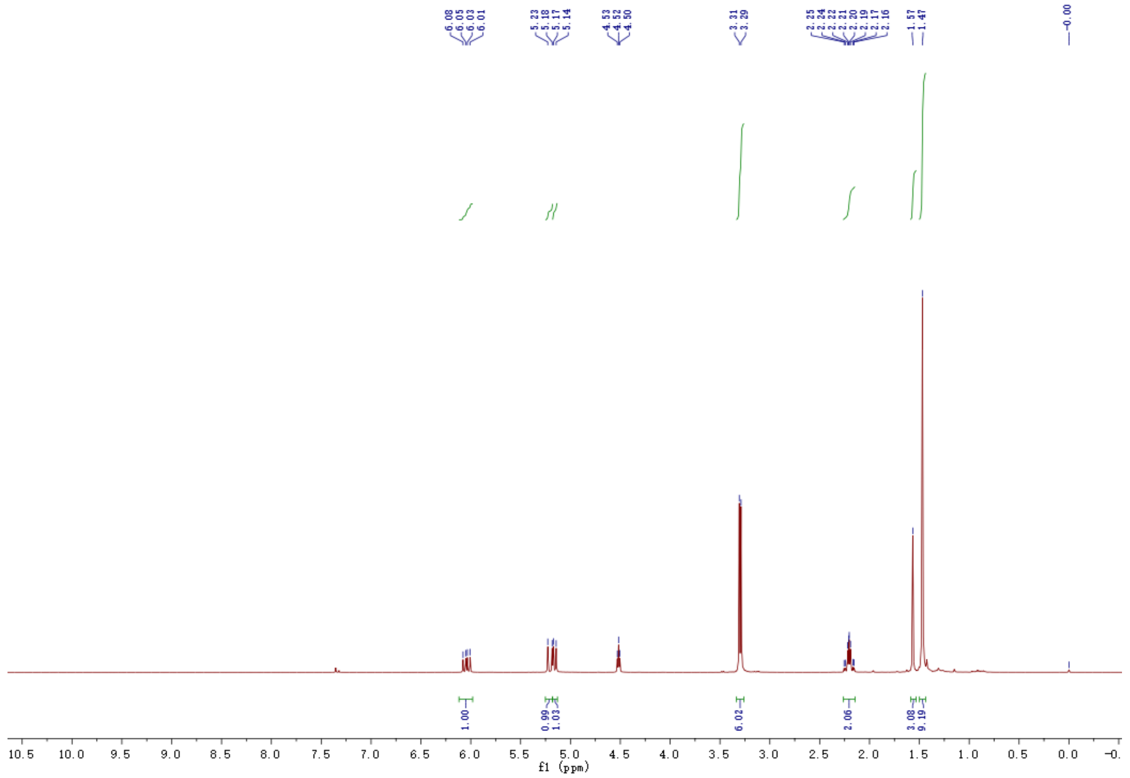

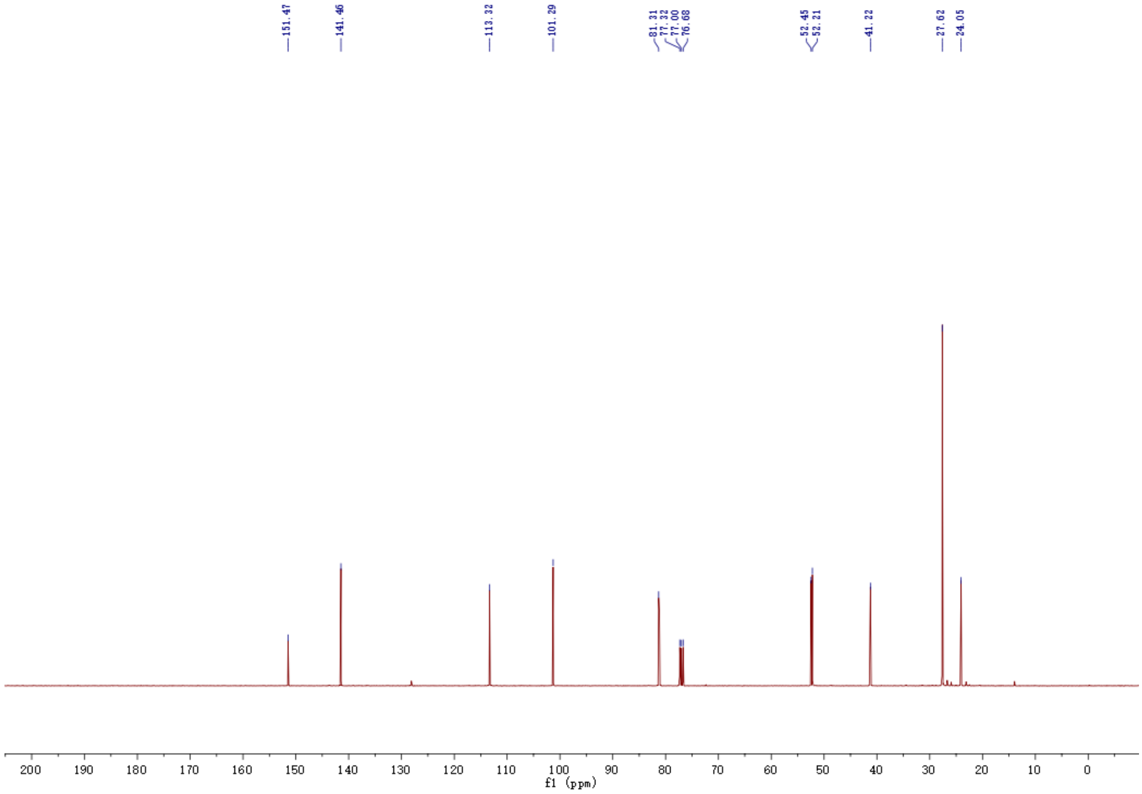

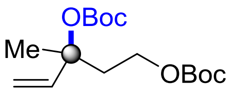

OBoc
Me
OBoc

1n

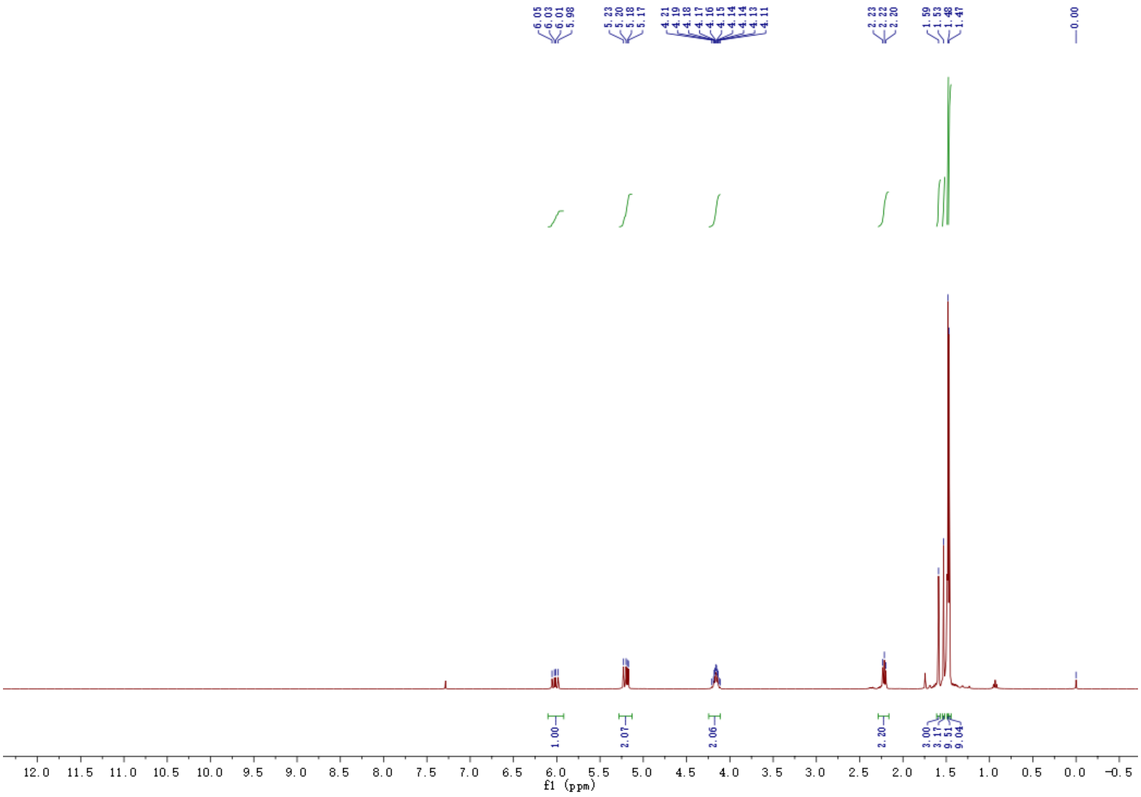

6.05
6.03
6.01
5.98
5.23
5.20
5.18
5.17
4.21
4.19
4.18
4.17
4.16
4.15
4.14
4.14
4.13
4.11
2.23
2.22
2.20
1.59
1.53
1.48
1.47
0.00
1.00
2.07
2.06
2.20
3.00
3.17
9.51
9.04
f1 (ppm)

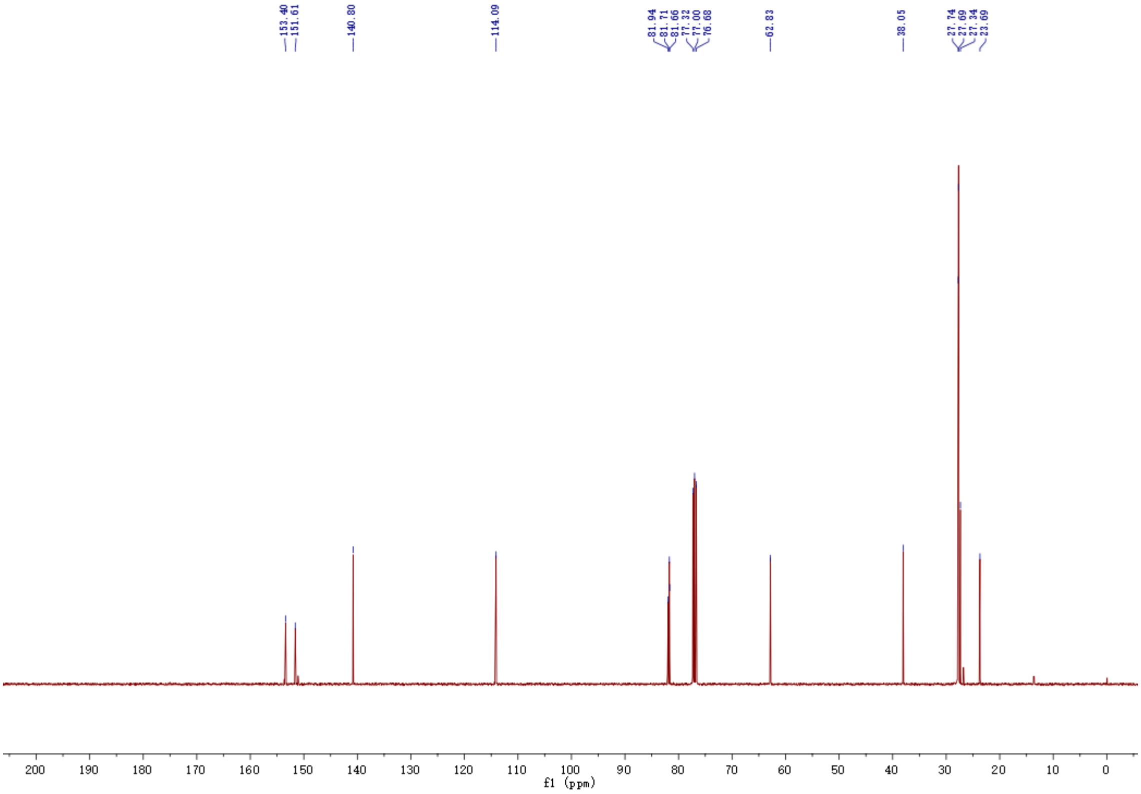

153.40
151.61
140.80
114.09
81.94
81.71
81.66
77.32
77.00
76.68
62.83
38.05
27.74
27.69
27.34
23.69
f1 (ppm)

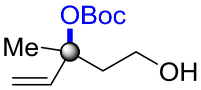
OBoc
Me
OH

1o

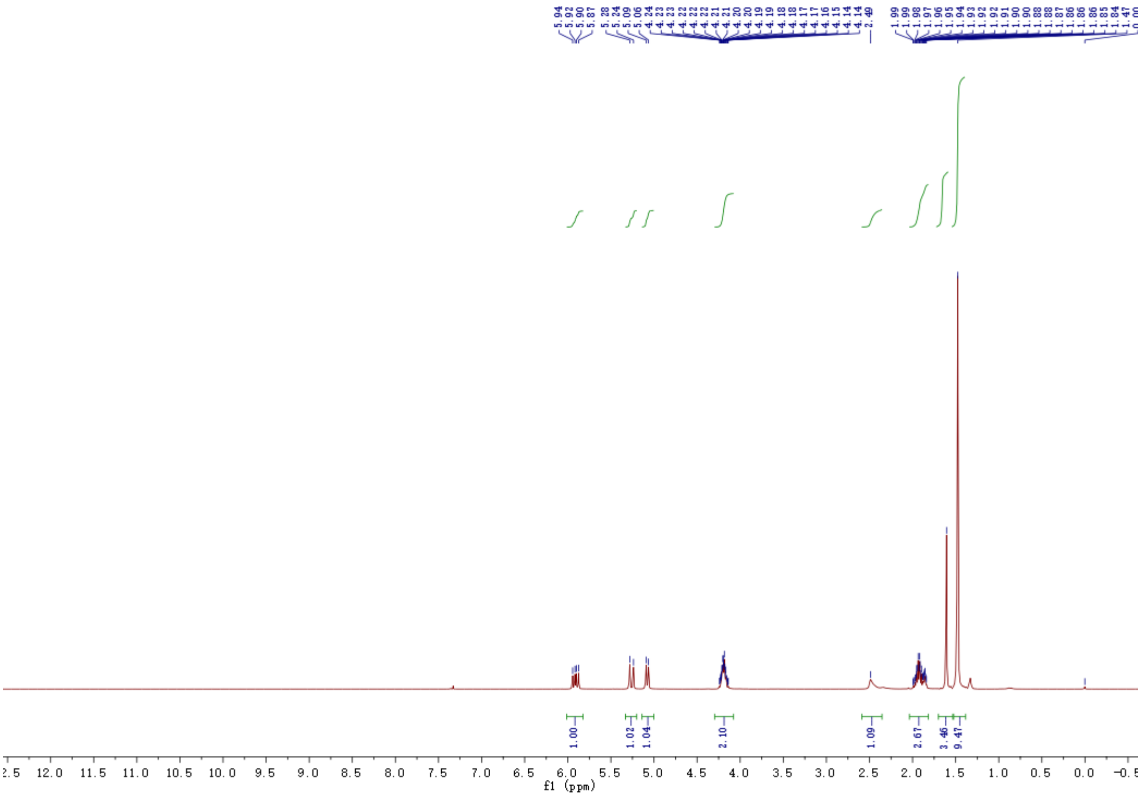
f1 (ppm)

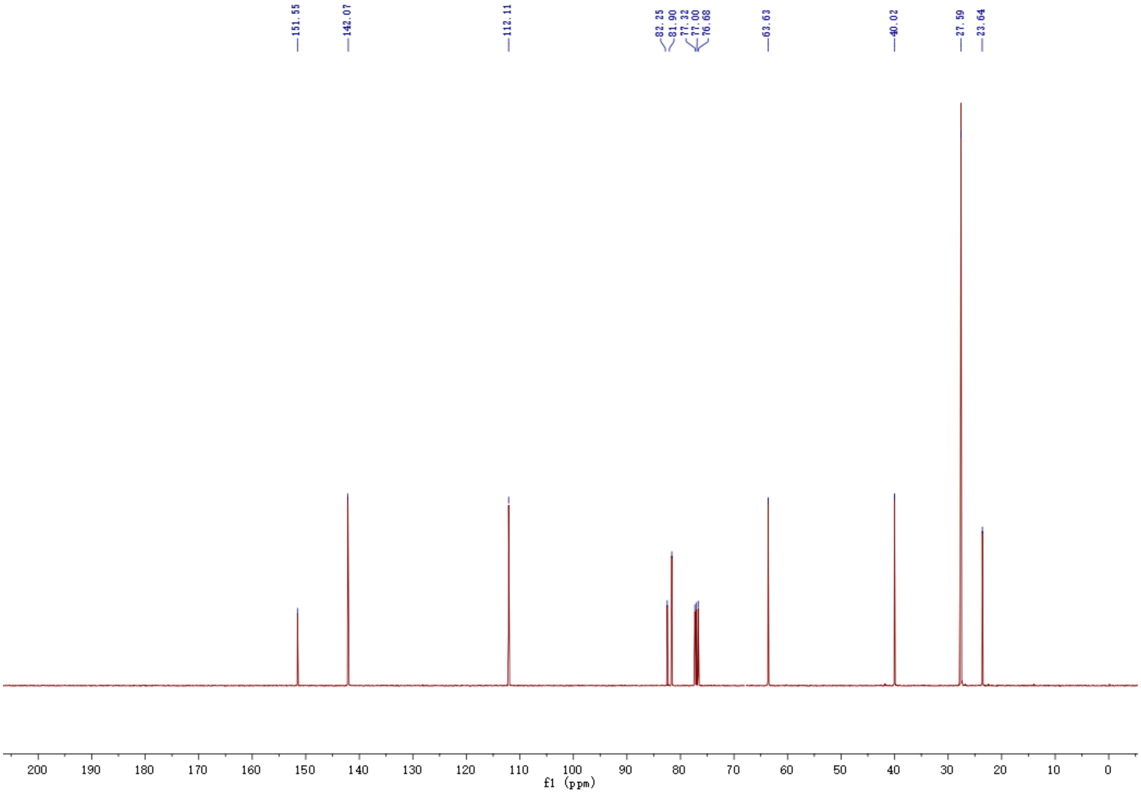
151.55
142.07
112.11
82.25
81.90
77.32
77.00
76.68
63.63
40.02
27.59
23.64
f1 (ppm)

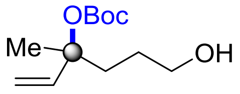

1p

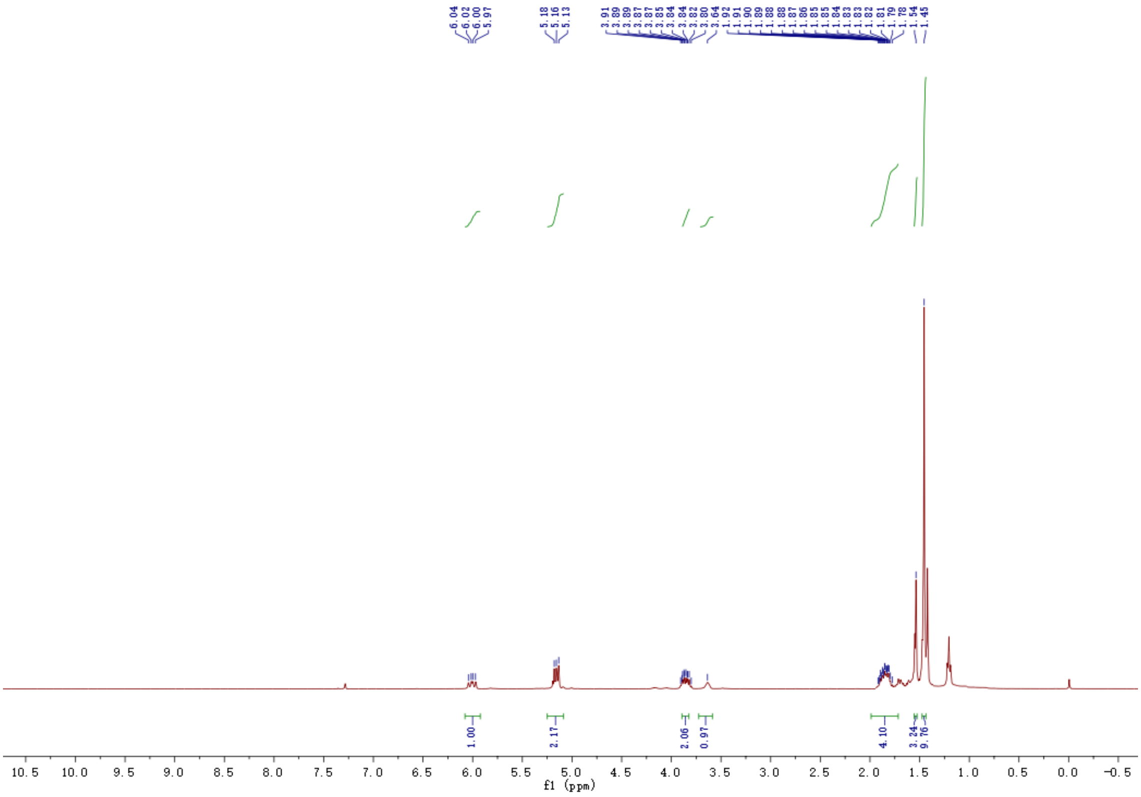
6.04
6.02
6.00
5.97
5.18
5.16
5.13
3.91
3.89
3.89
3.87
3.87
3.85
3.84
3.84
3.82
3.80
3.64
1.92
1.91
1.90
1.89
1.88
1.88
1.87
1.86
1.85
1.85
1.84
1.83
1.83
1.82
1.81
1.79
1.78
1.54
1.45
1.00
2.17
2.06
0.97
4.10
3.24
9.76
f1 (ppm)

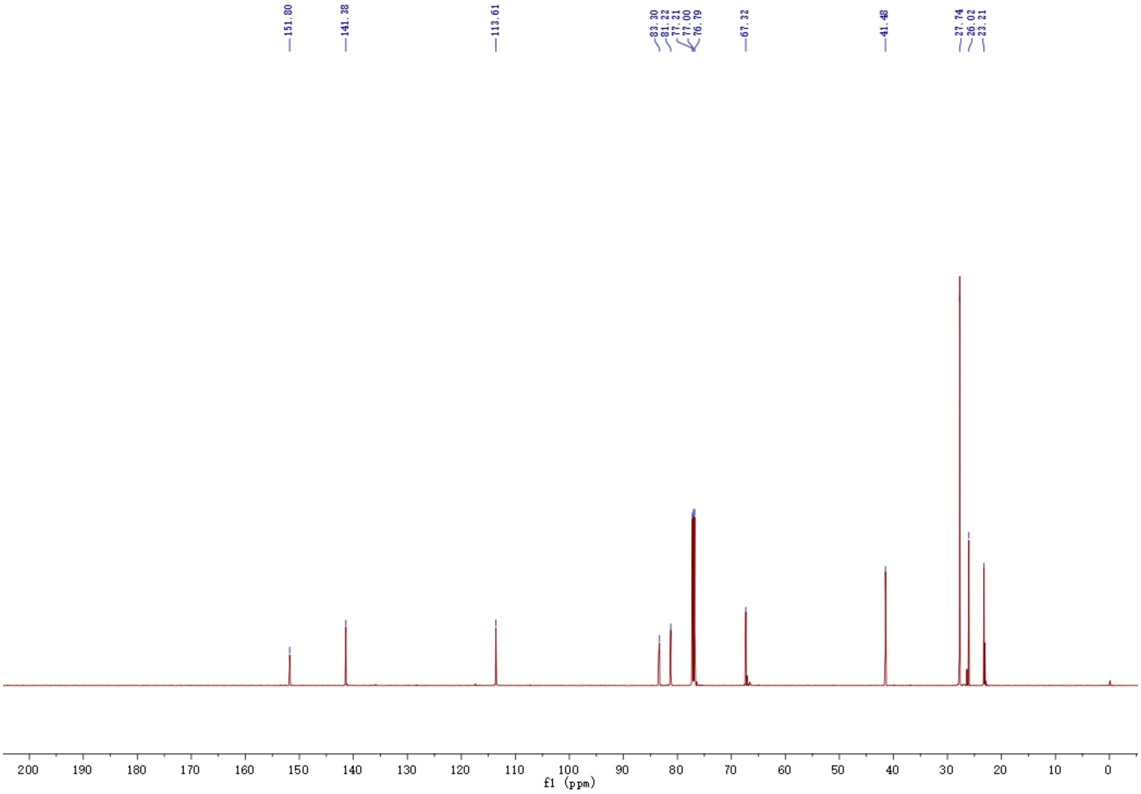
151.80
141.38
113.61
83.30
81.22
77.21
77.00
76.79
67.32
41.48
27.74
26.02
23.21
f1 (ppm)

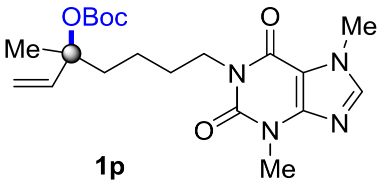

1p

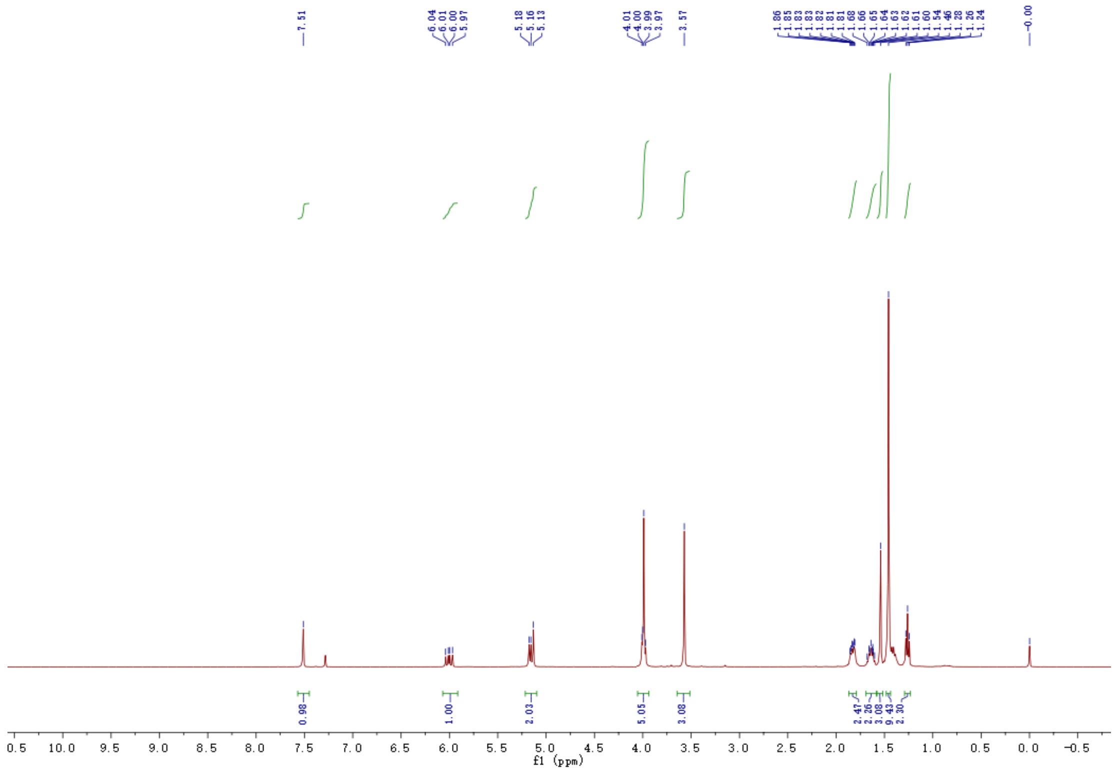

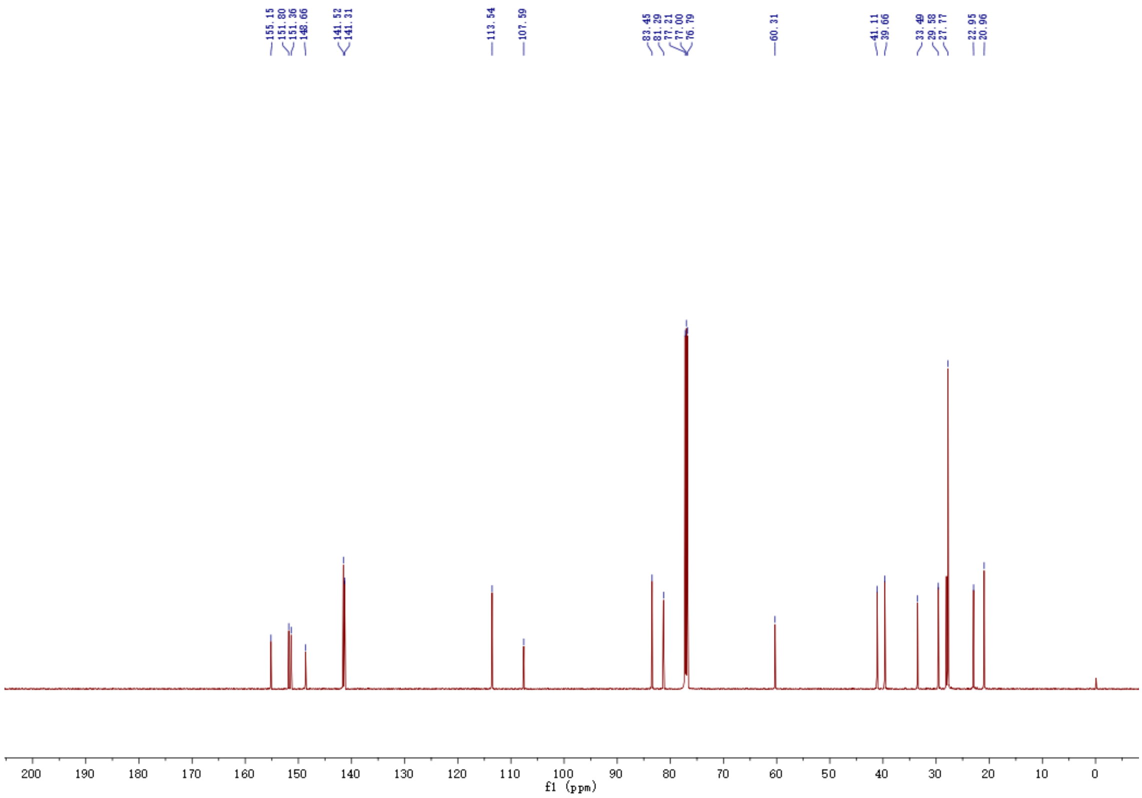

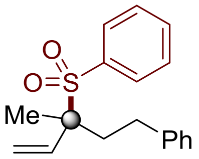
O
O=S
Me
Ph

3aa

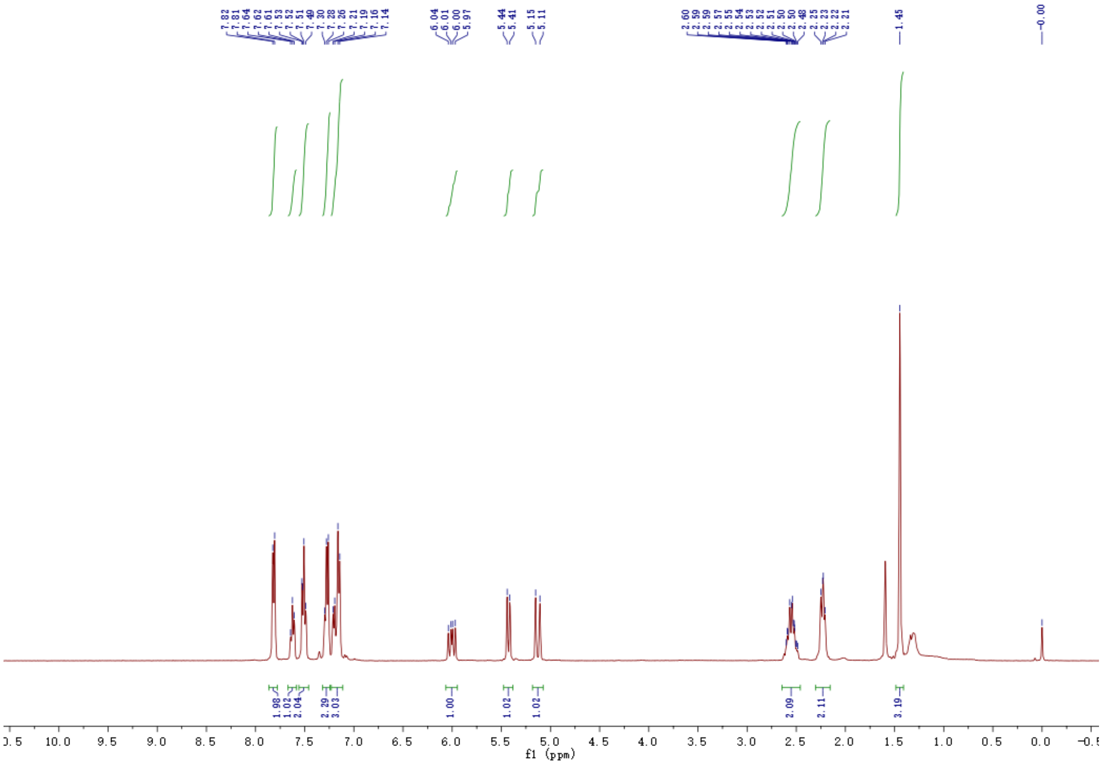
7.82 7.81 7.64 7.62 7.61 7.53 7.52 7.51 7.49 7.30 7.28 7.26 7.21 7.19 7.16 7.14
6.04 6.01 6.00 5.97
5.44 5.41 5.15 5.11
2.60 2.59 2.59 2.57 2.55 2.54 2.53 2.52 2.51 2.50 2.50 2.48 2.25 2.23 2.22 2.21
1.45
-0.00
1.98 1.02 2.04 2.29 3.03 1.00 1.02 1.02 2.09 2.11 3.19
f1 (ppm)

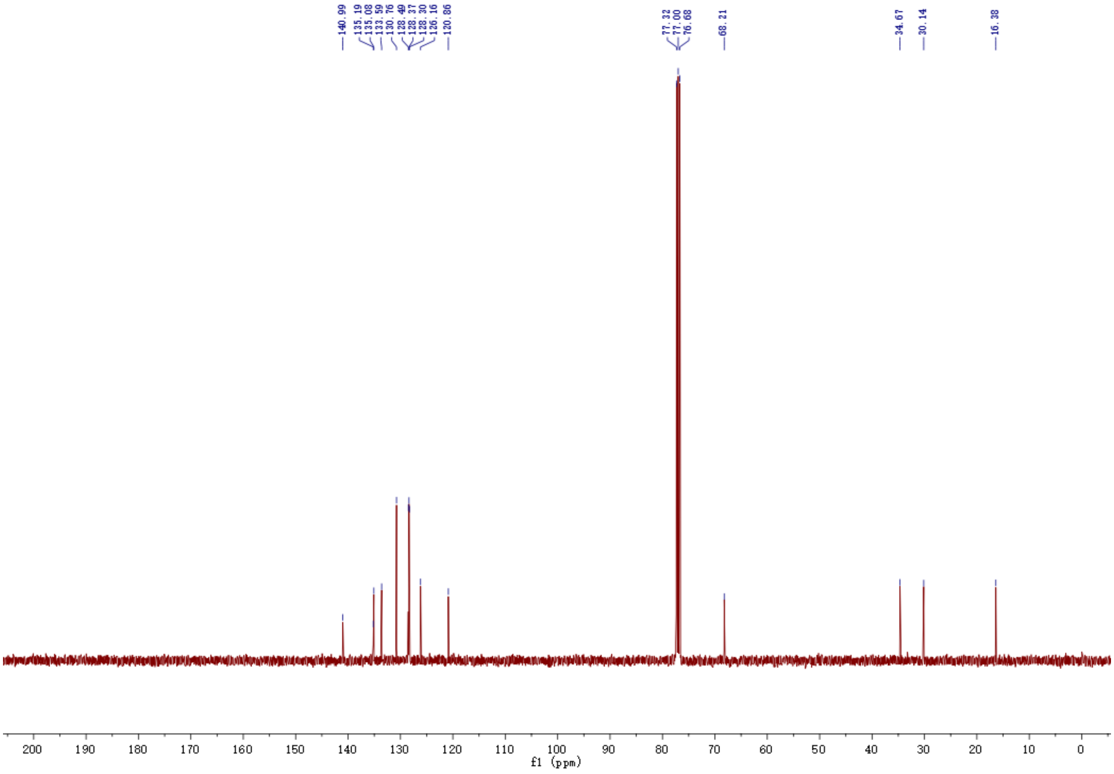
140.99 135.19 135.08 133.59 130.76 128.49 128.37 128.30 126.16 120.86
77.32 77.00 76.68
68.21
34.67 30.14
16.38
f1 (ppm)

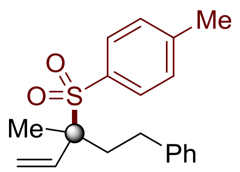

3ab

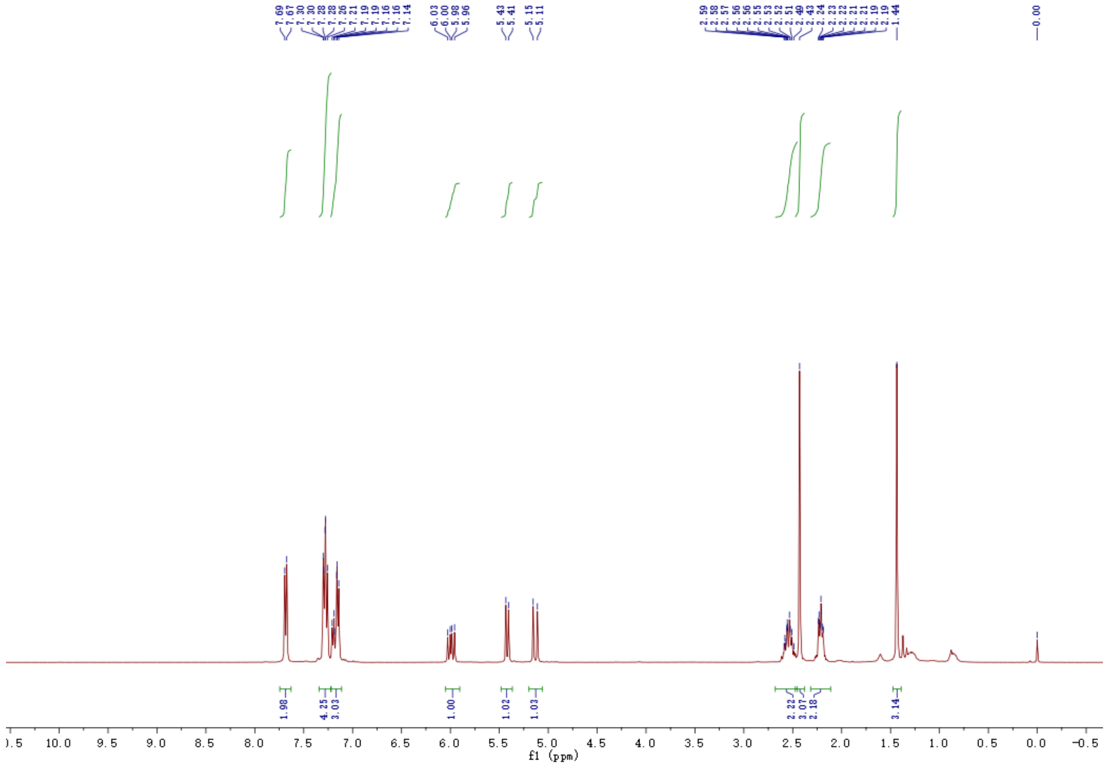

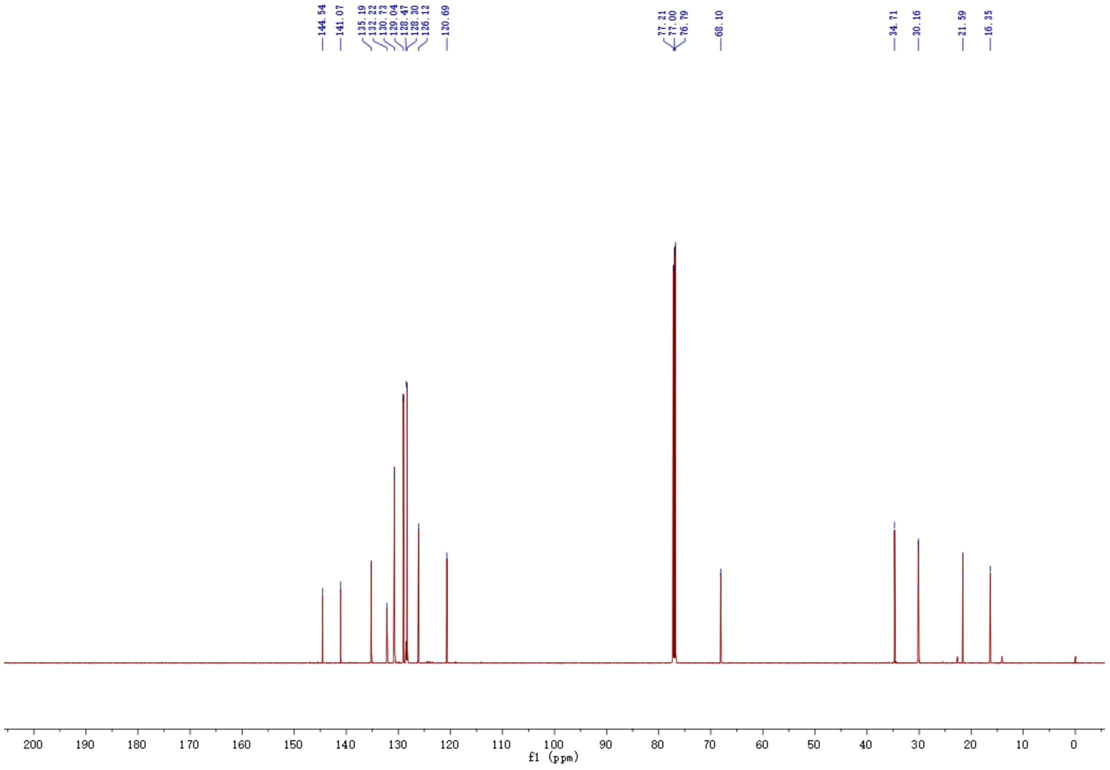

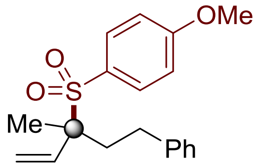

3ac

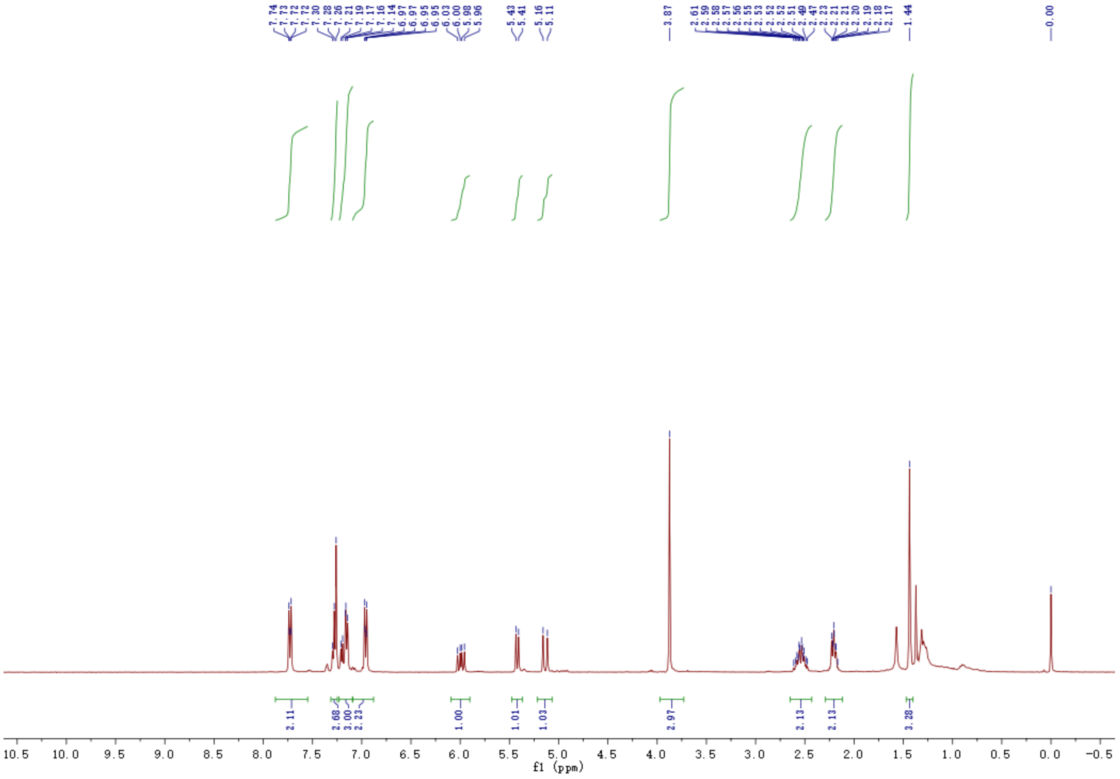

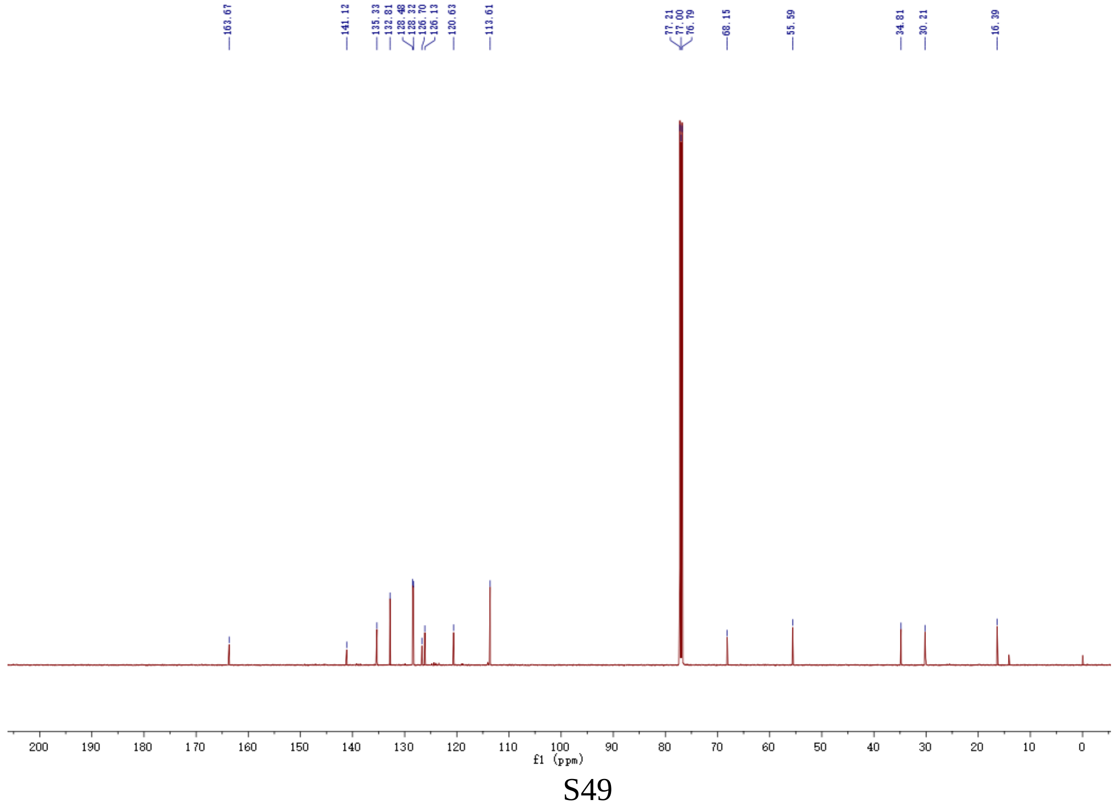

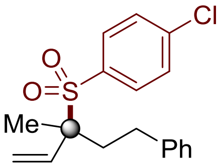

3ad

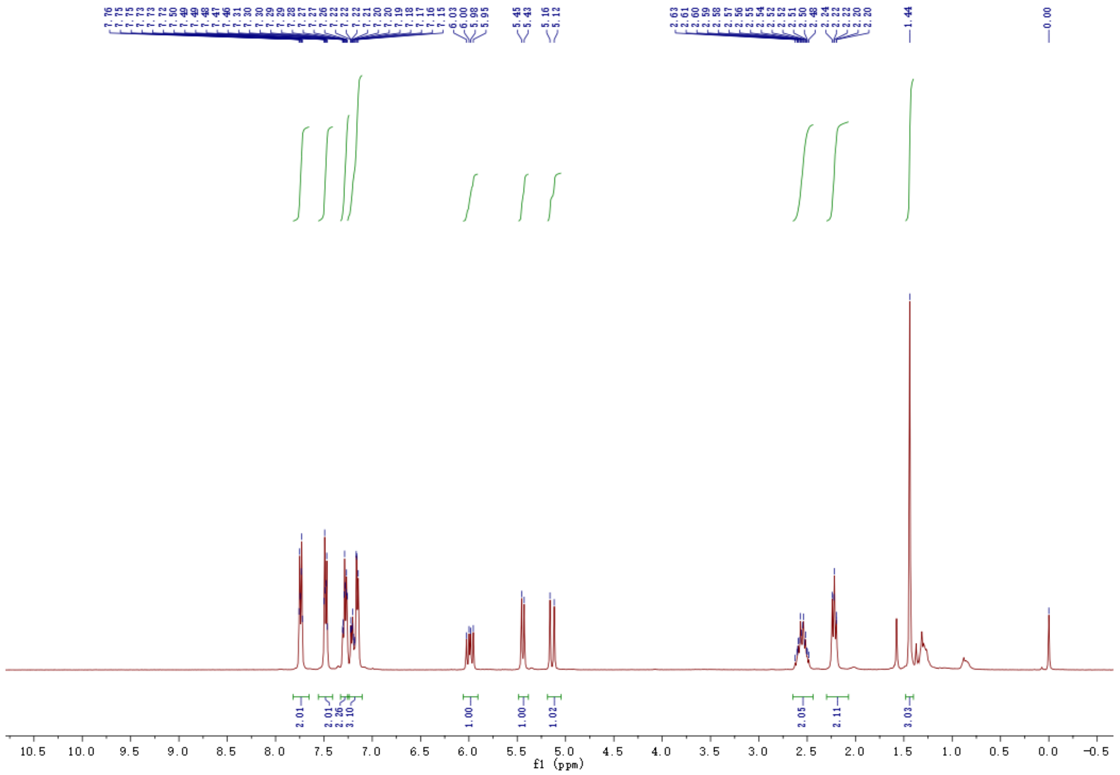

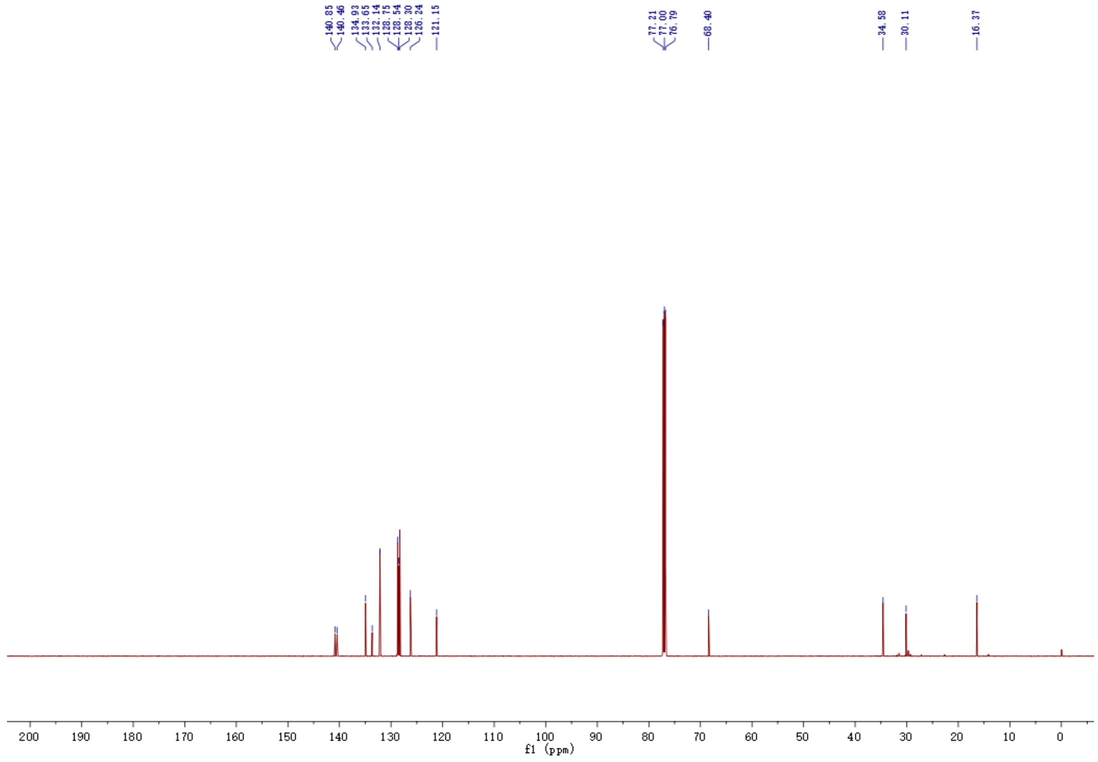

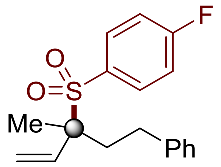
F
O
O=S
Me
Ph

3ae

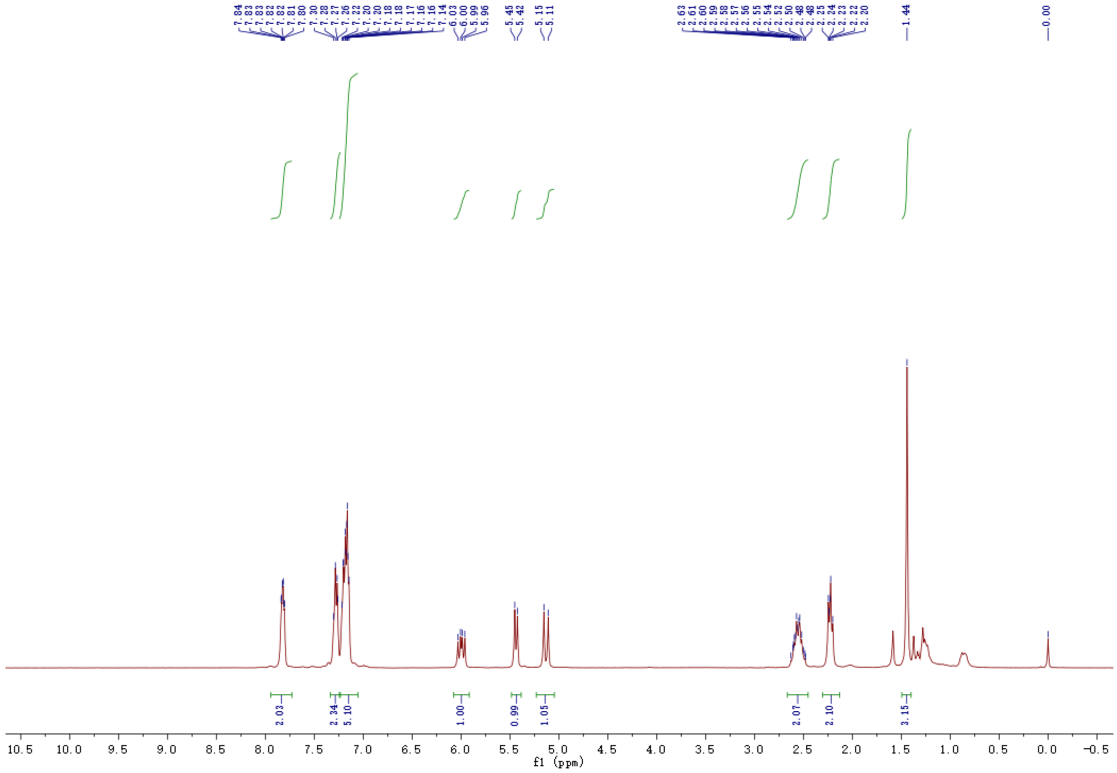
7.84 7.83 7.83 7.82 7.82 7.81 7.80 7.30 7.28 7.27 7.26 7.22 7.20 7.20 7.18 7.18 7.17 7.16 7.16 7.14 6.03 6.00 5.99 5.96 5.45 5.42 5.15 5.11
2.63 2.61 2.60 2.59 2.58 2.57 2.56 2.55 2.54 2.52 2.50 2.48 2.48 2.25 2.24 2.23 2.22 2.20
1.44
0.00
2.03 2.34 5.10 1.00 0.99 1.05 2.07 2.10 3.15
10.5 10.0 9.5 9.0 8.5 8.0 7.5 7.0 6.5 6.0 5.5 5.0 4.5 4.0 3.5 3.0 2.5 2.0 1.5 1.0 0.5 0.0 -0.5
f1 (ppm)

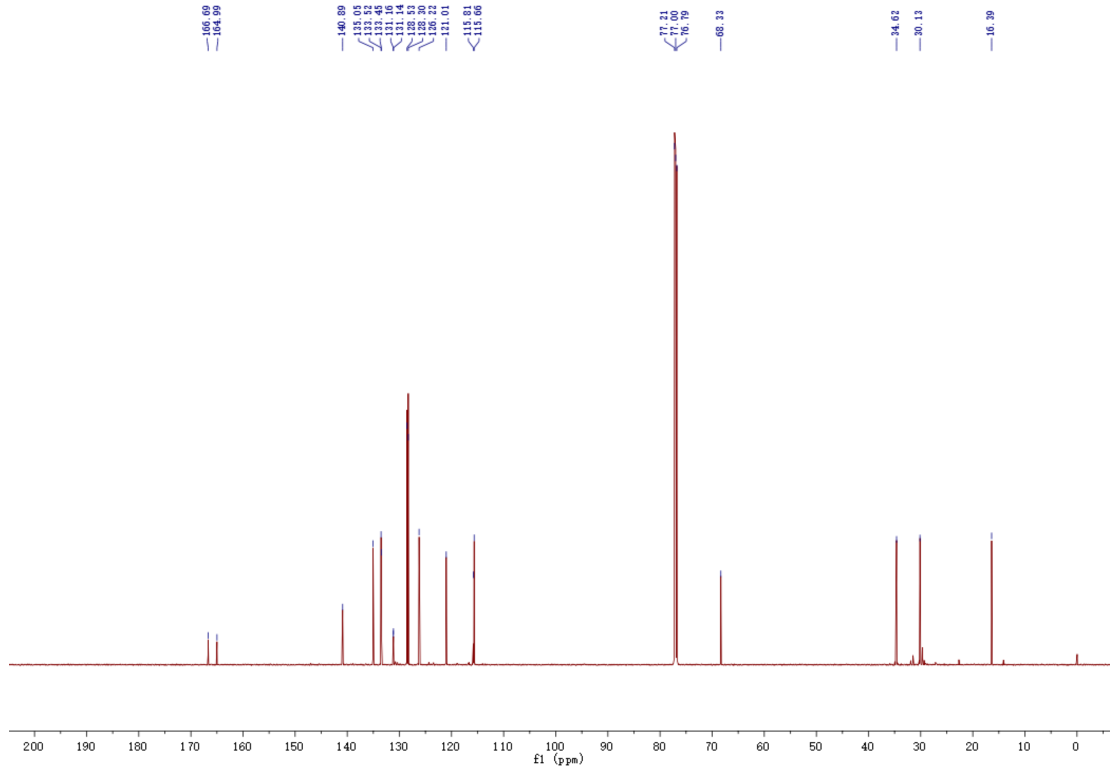
166.69 164.99
140.89 135.05 133.52 133.45 131.16 131.14 128.53 128.30 126.22 121.01 115.81 115.66
77.21 77.00 76.79
68.33
34.62
30.13
16.39
200 190 180 170 160 150 140 130 120 110 100 90 80 70 60 50 40 30 20 10 0
f1 (ppm)

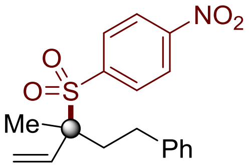

3af

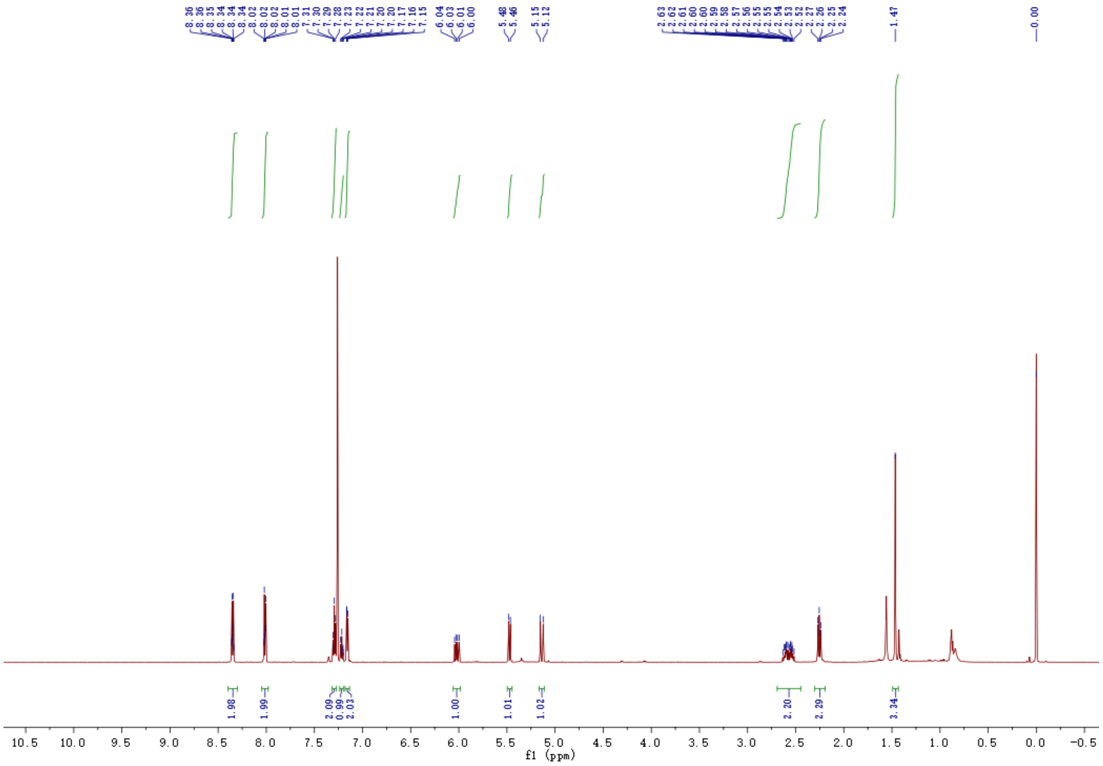

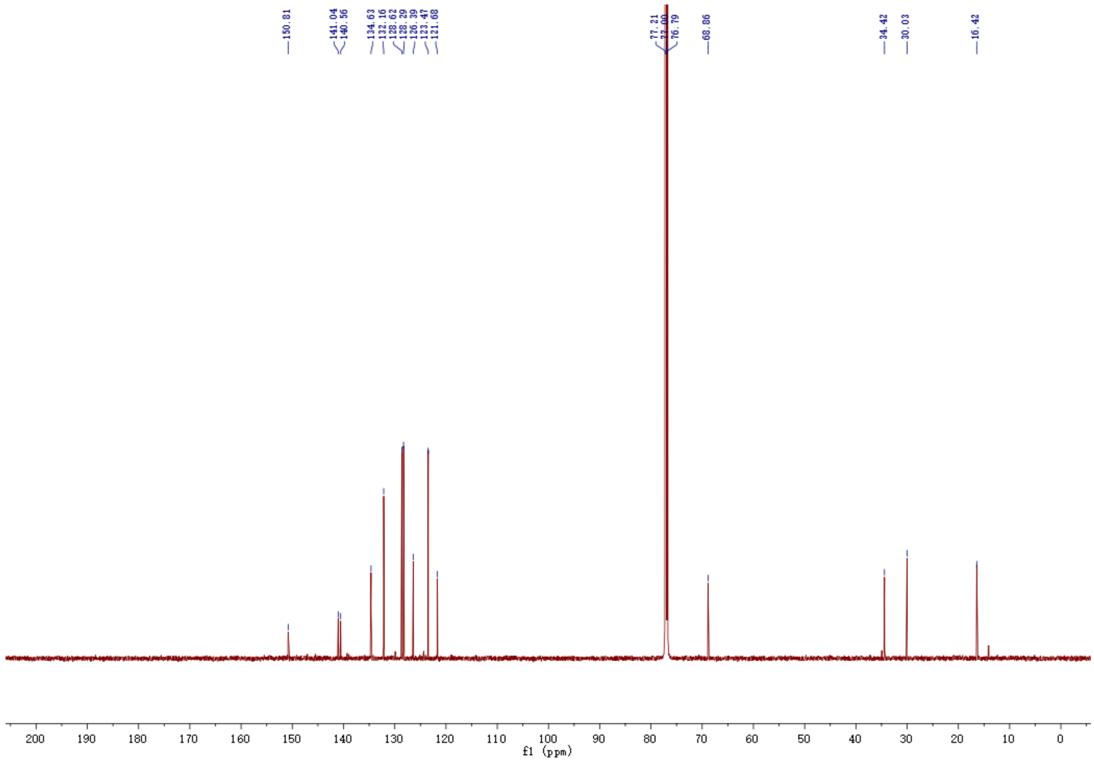

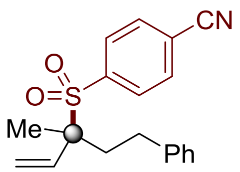

3ag

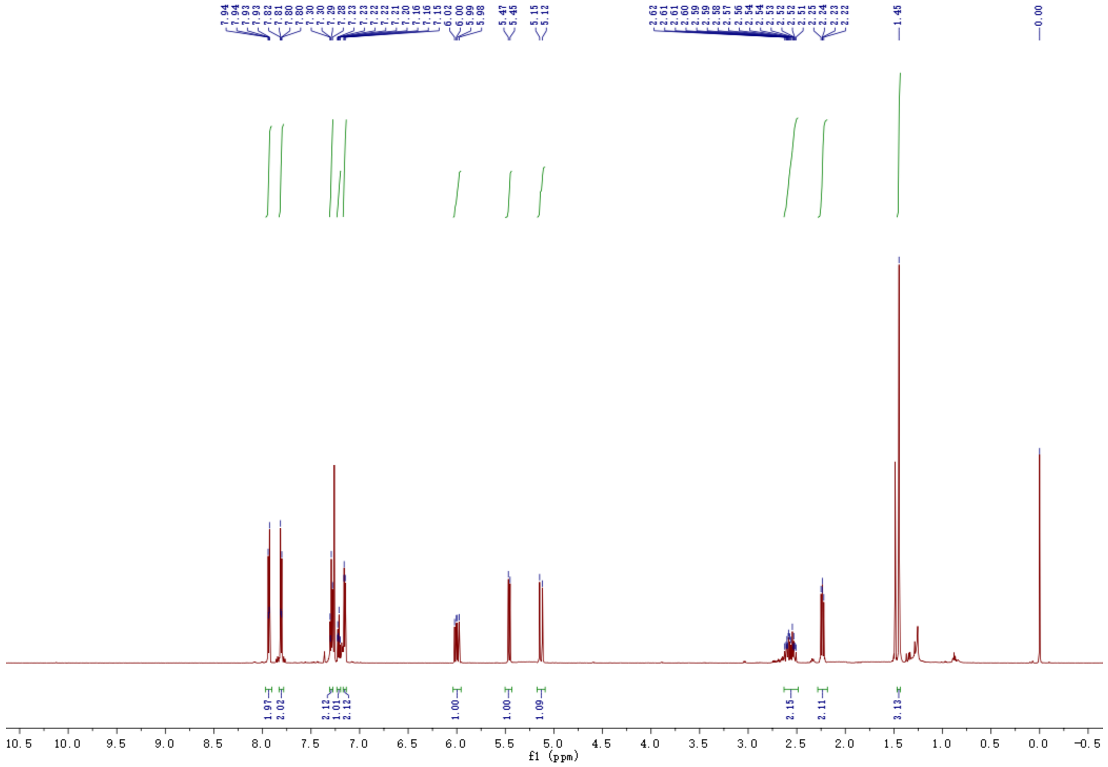

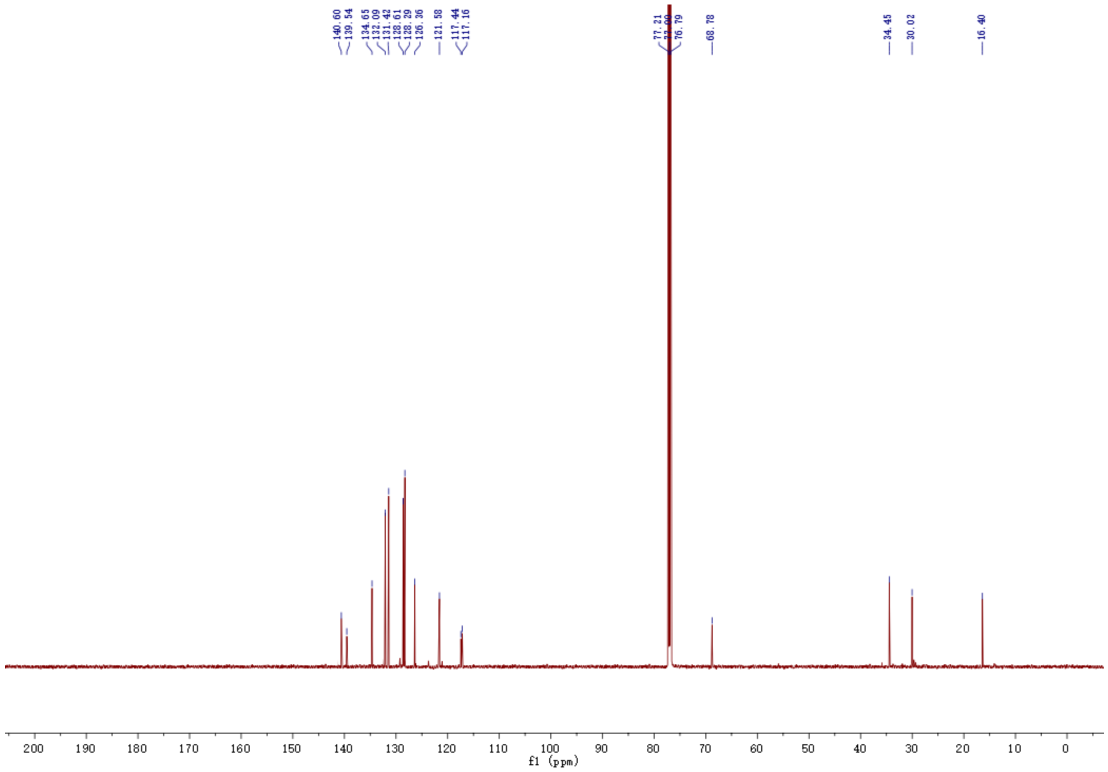

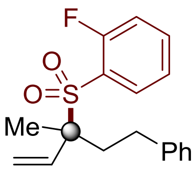

3ah

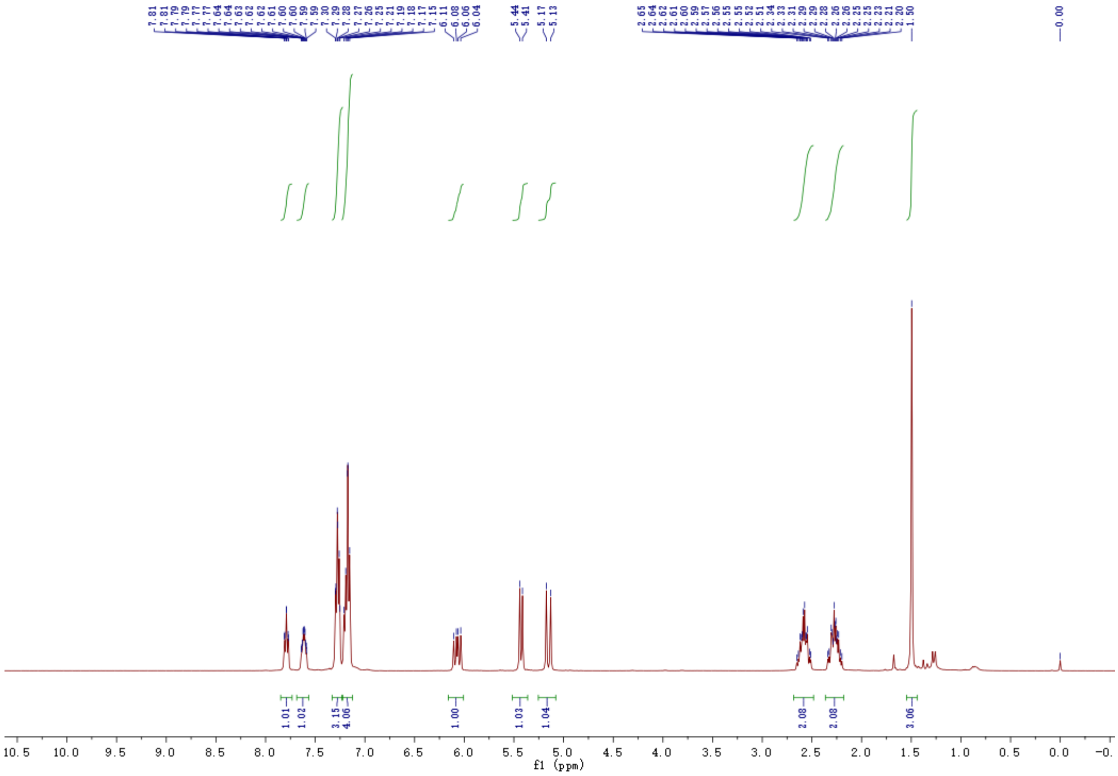

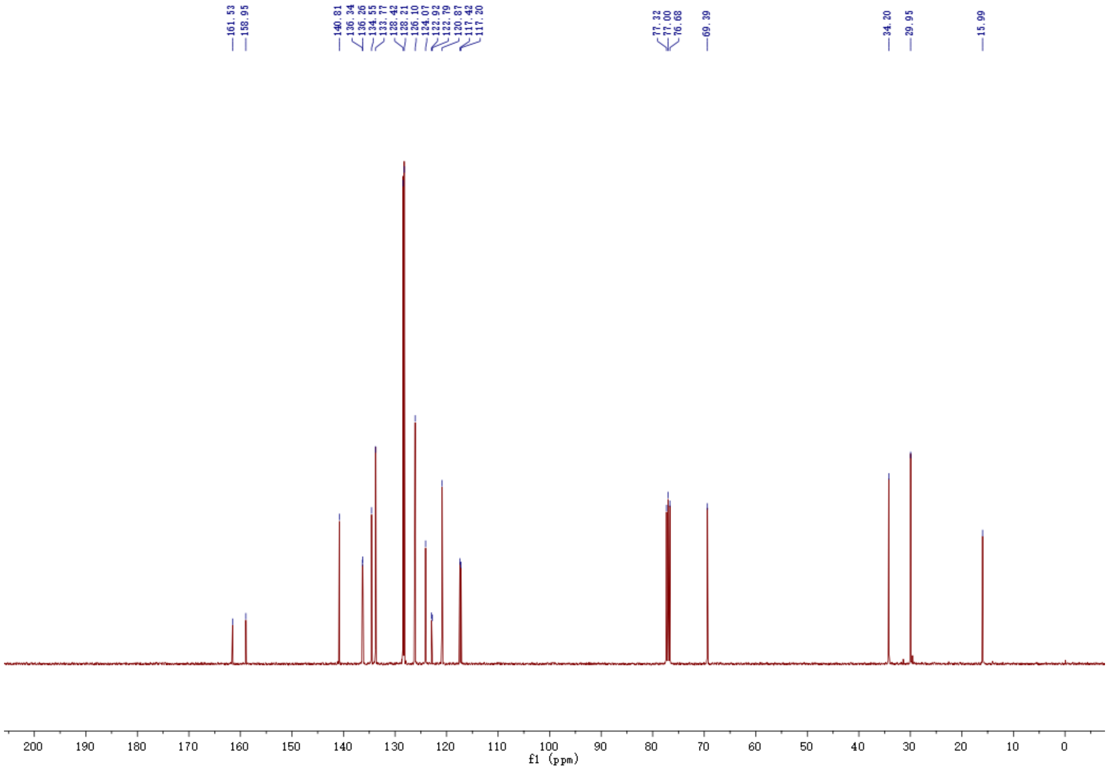

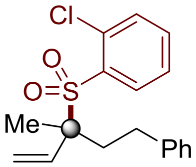

3ai

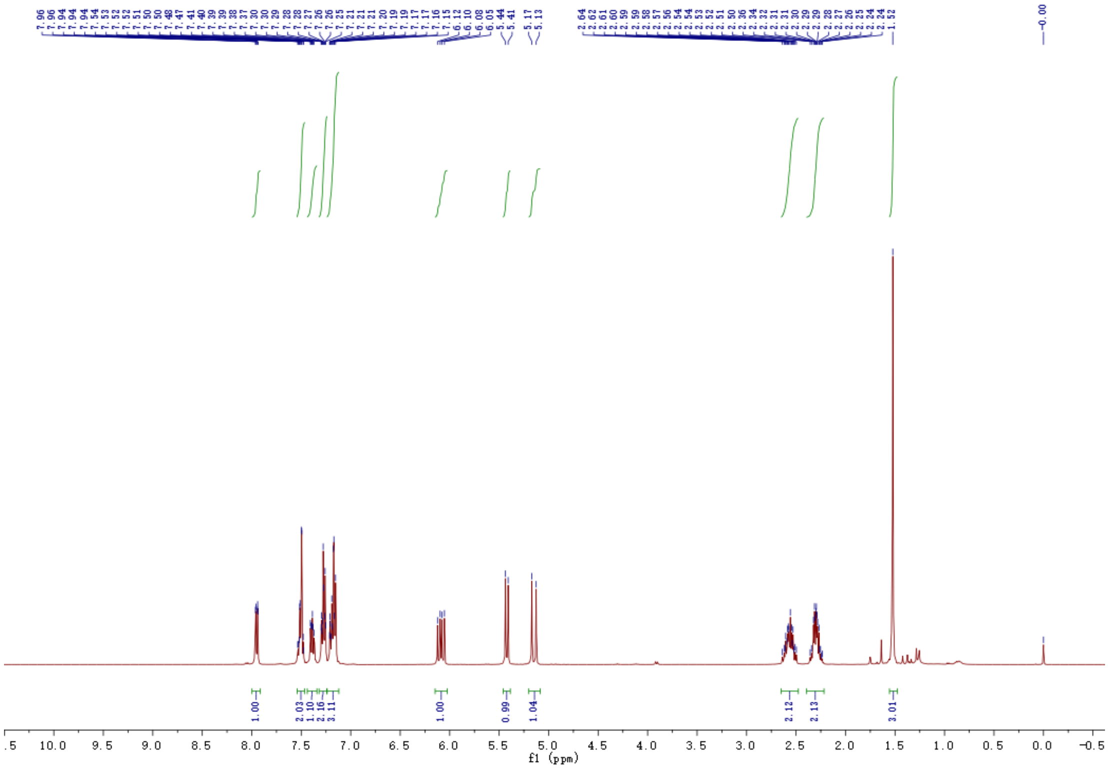

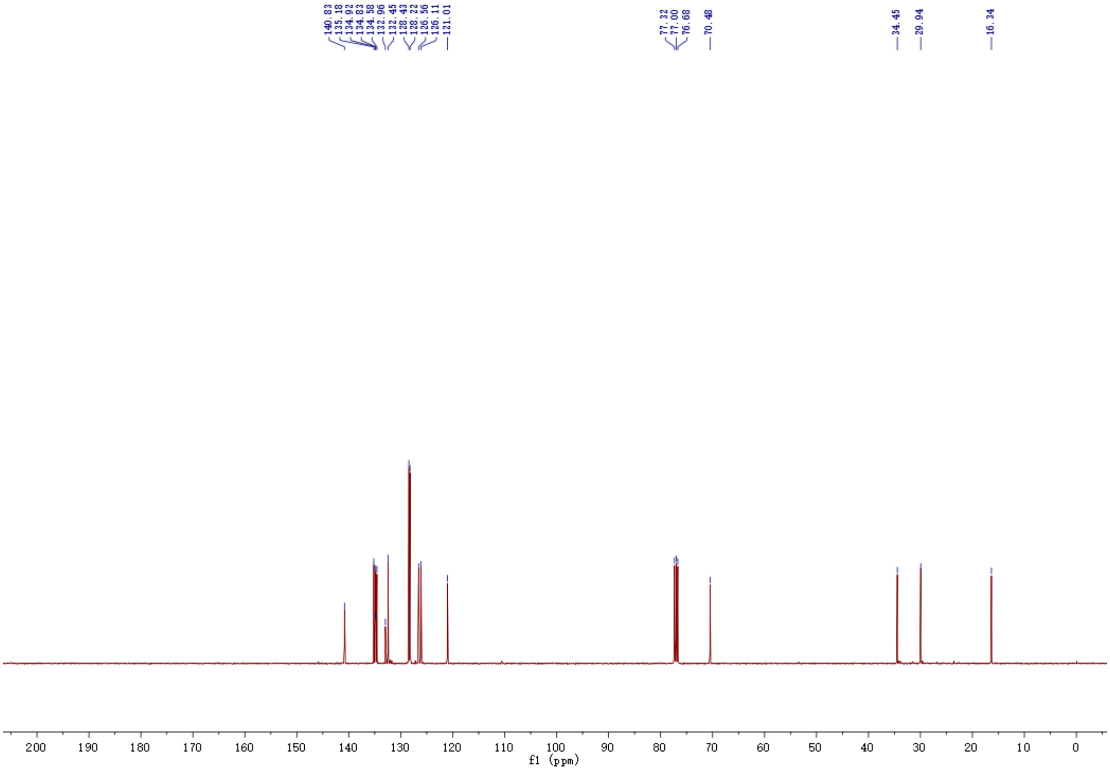

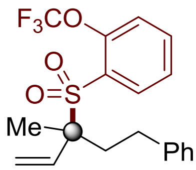
F3CO
O
O=S
Me
Ph

3aj

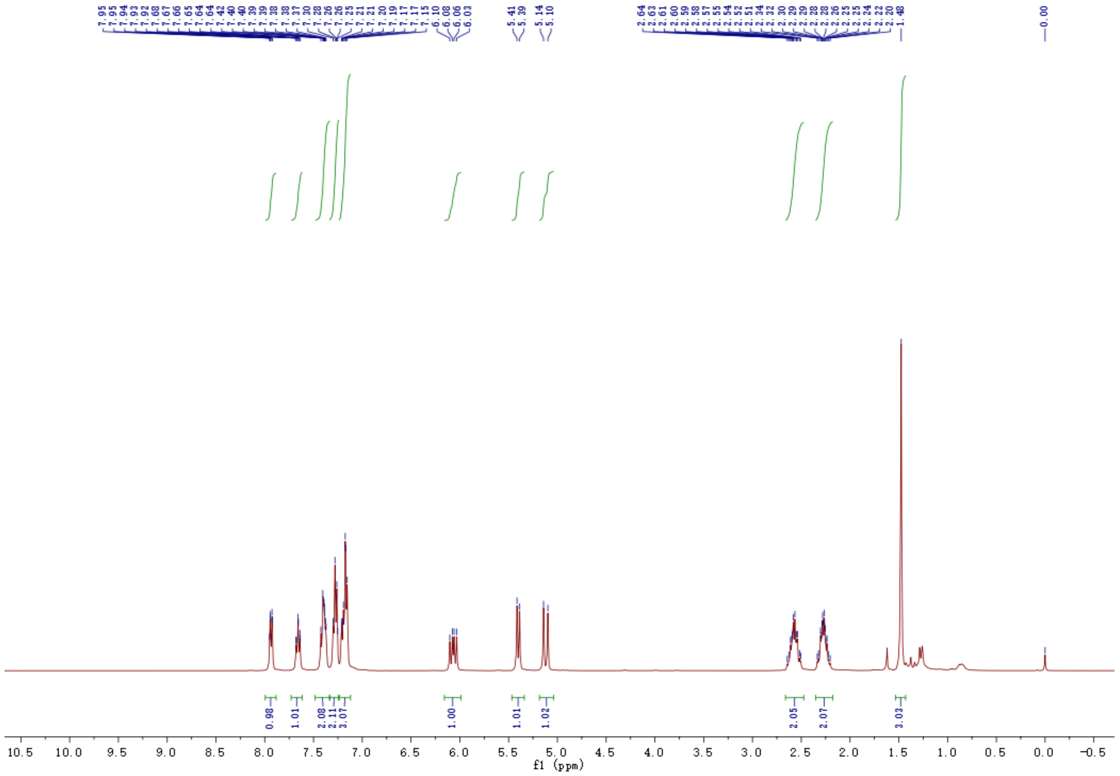
0.98
1.01
2.08
2.11
3.07
1.00
1.01
1.02
2.05
2.07
3.03
f1 (ppm)

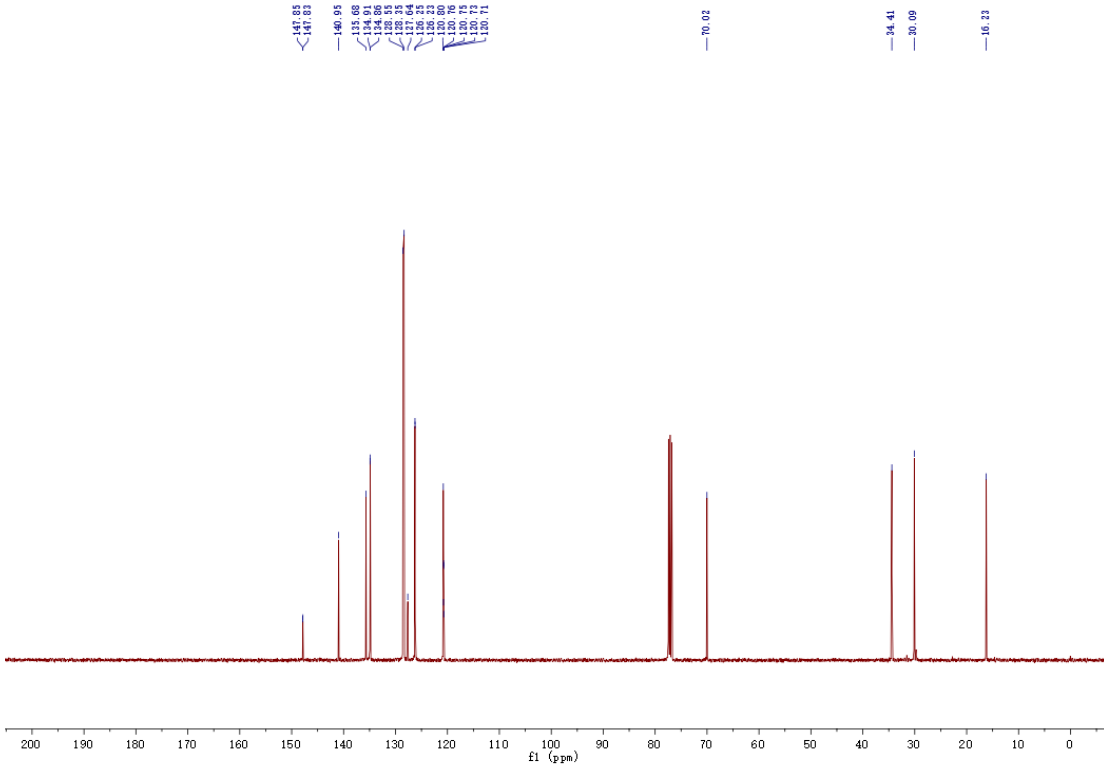
147.85
147.83
140.95
135.68
134.91
134.86
128.55
128.35
127.64
126.25
126.23
120.80
120.76
120.75
120.73
120.71
70.02
34.41
30.09
16.23
f1 (ppm)

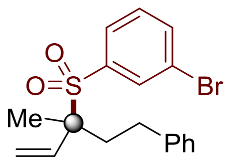

3ak

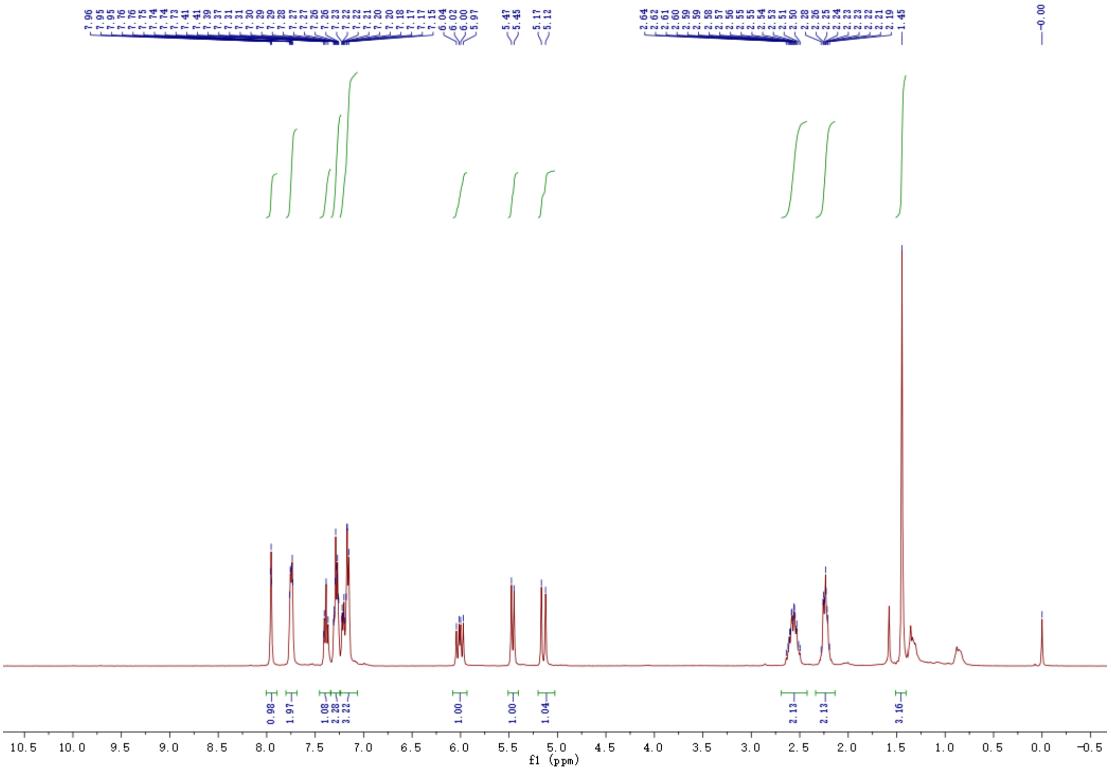
f1 (ppm)

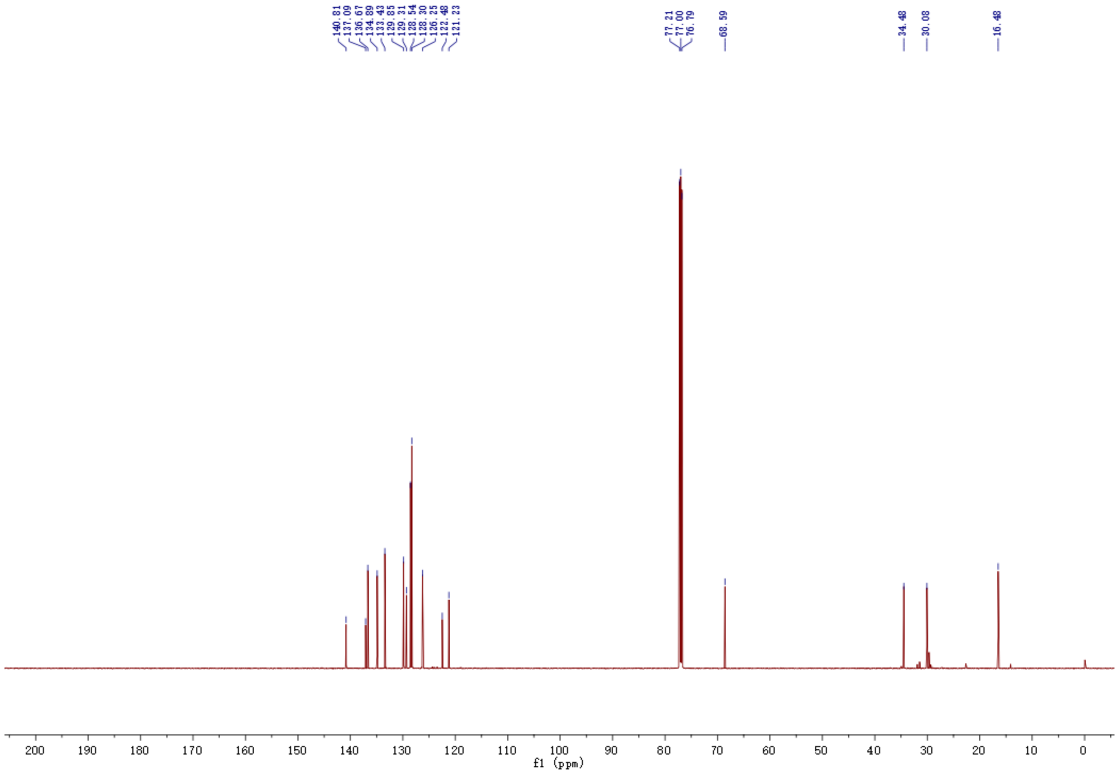
140.81
137.09
136.67
134.89
133.43
129.85
129.31
128.54
128.30
126.25
122.48
121.23
77.21
77.00
76.79
68.59
34.48
30.08
16.48
f1 (ppm)

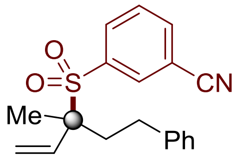

3al

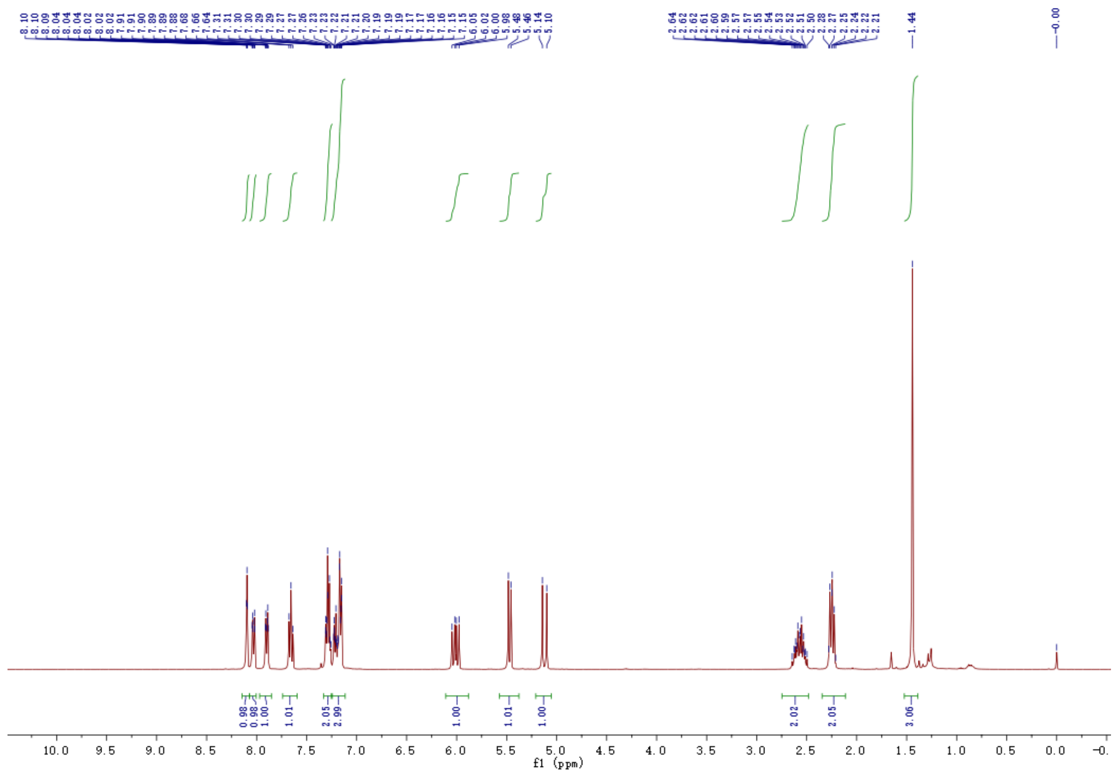

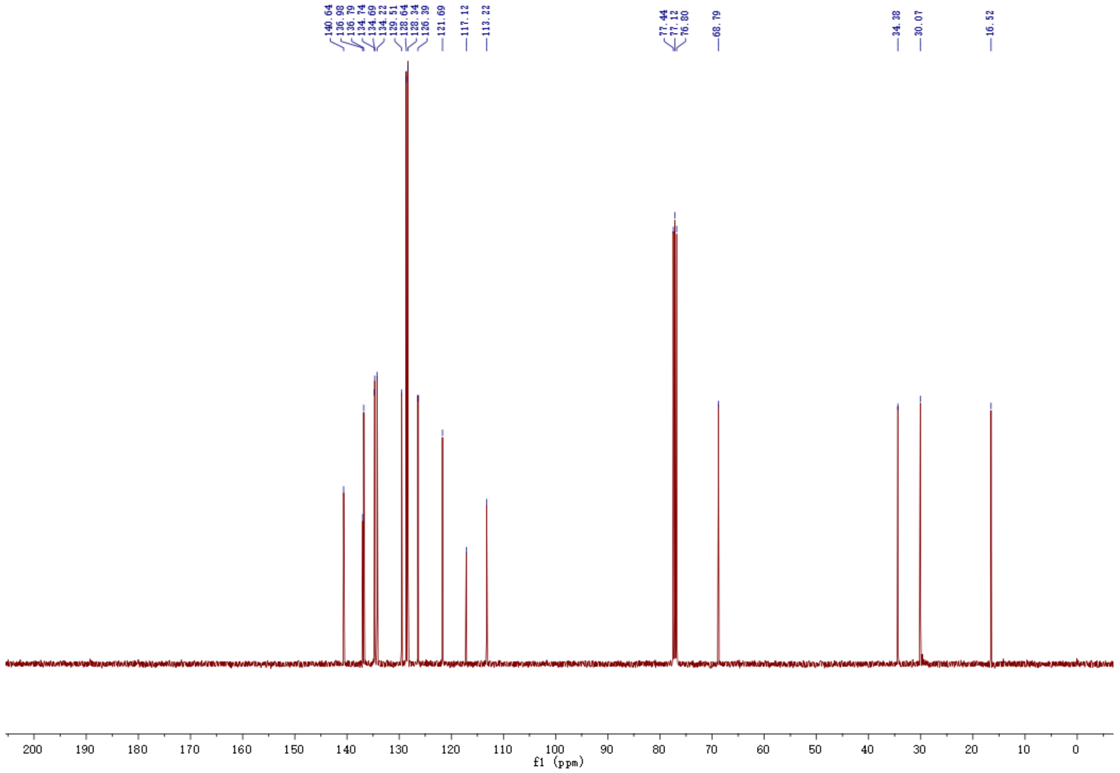

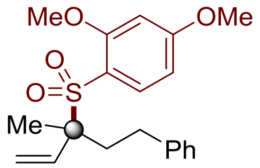

3am

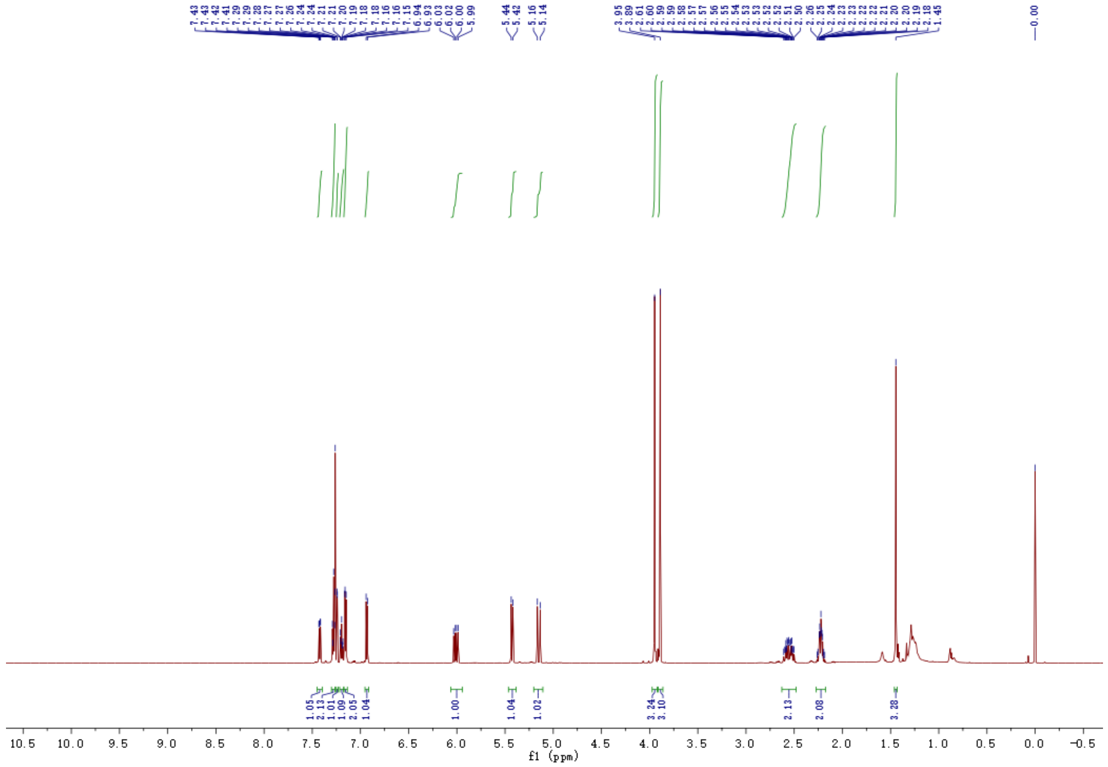

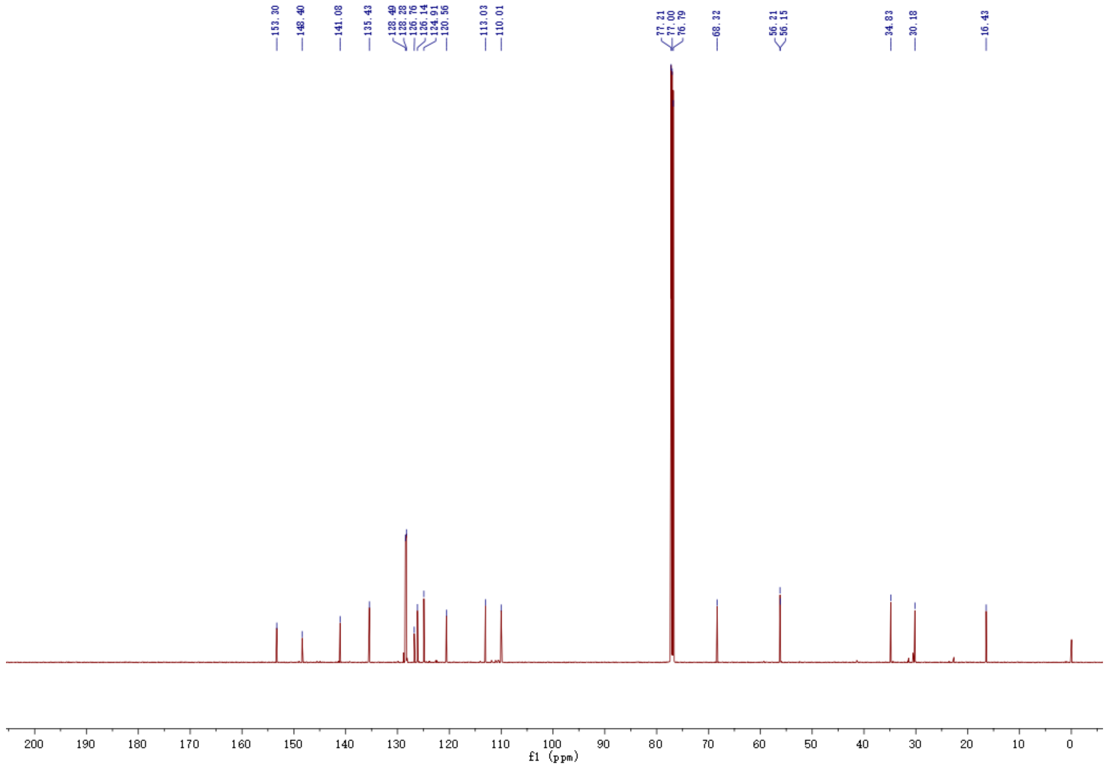

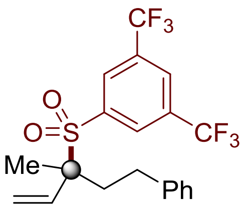

3an

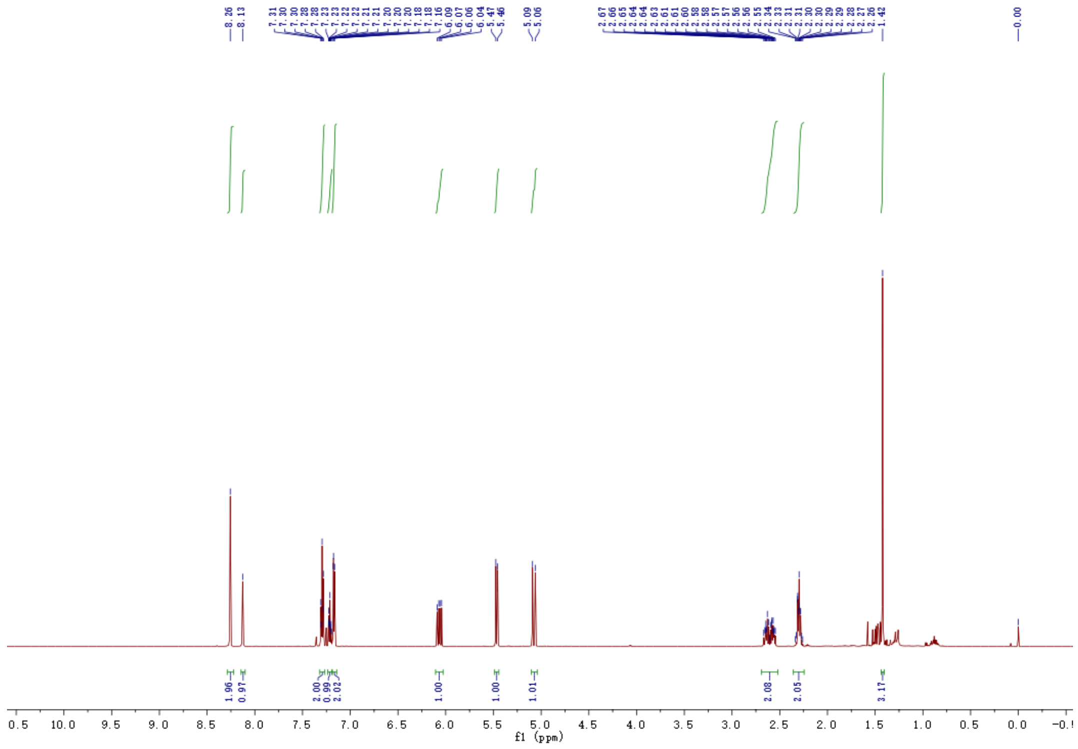

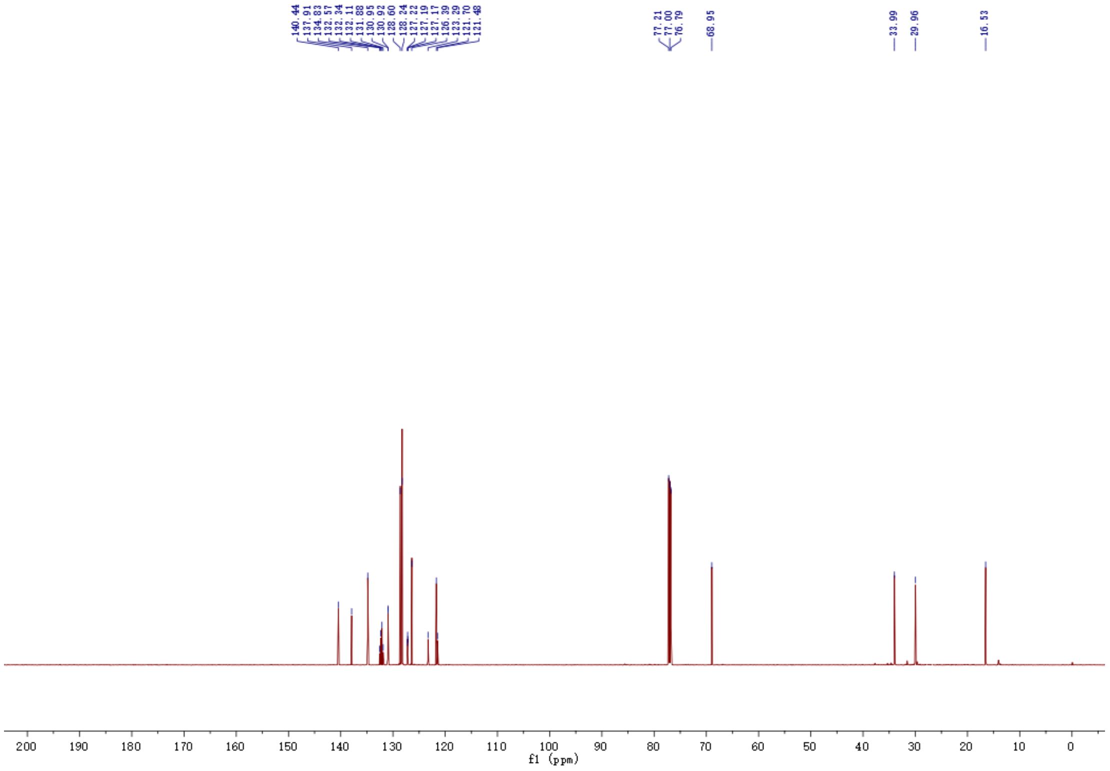

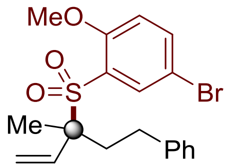

3ao

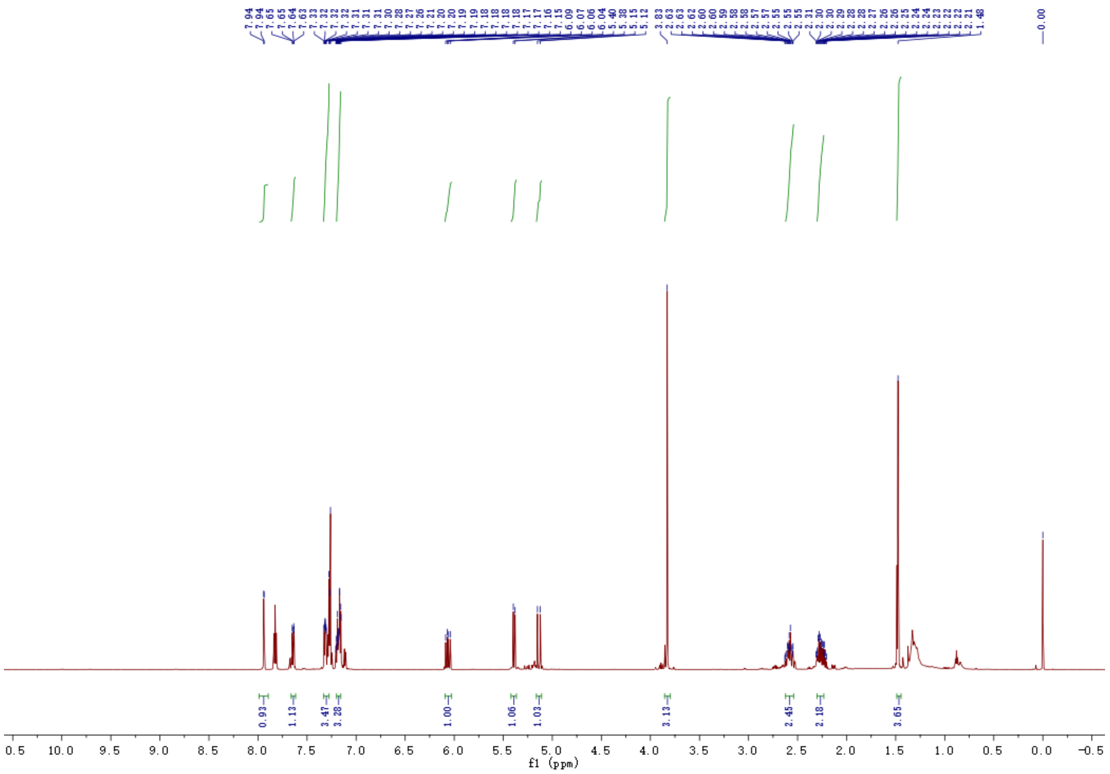

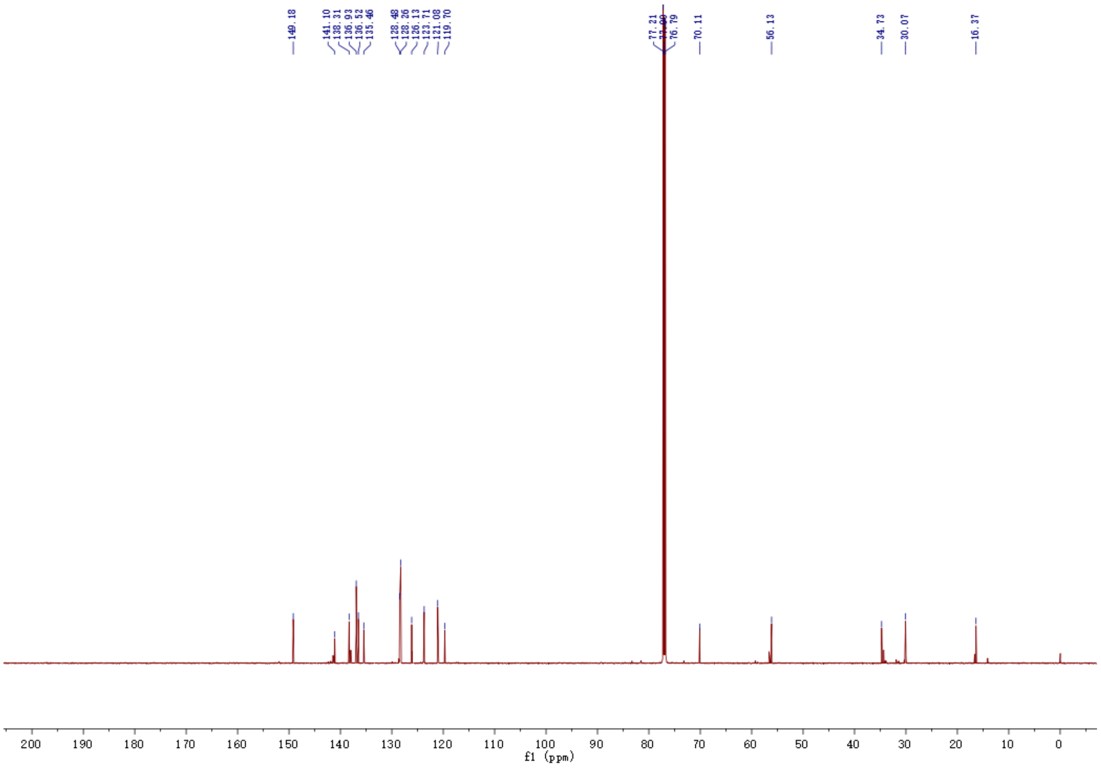

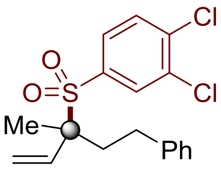

3ap

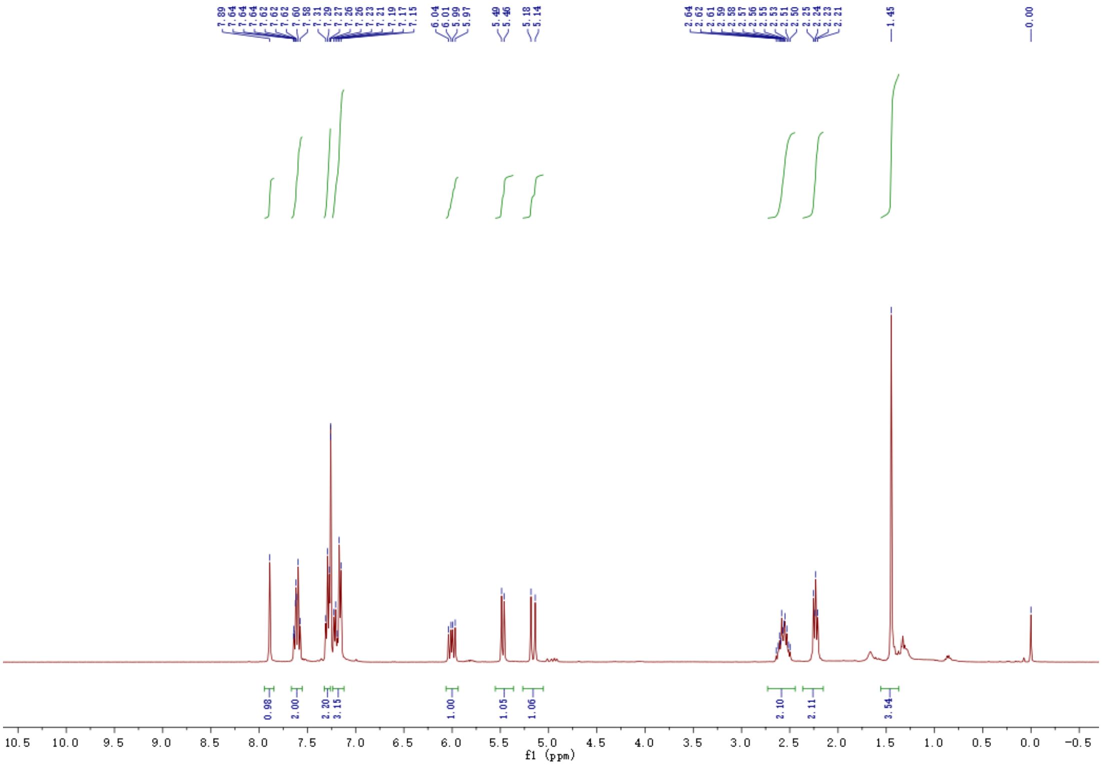

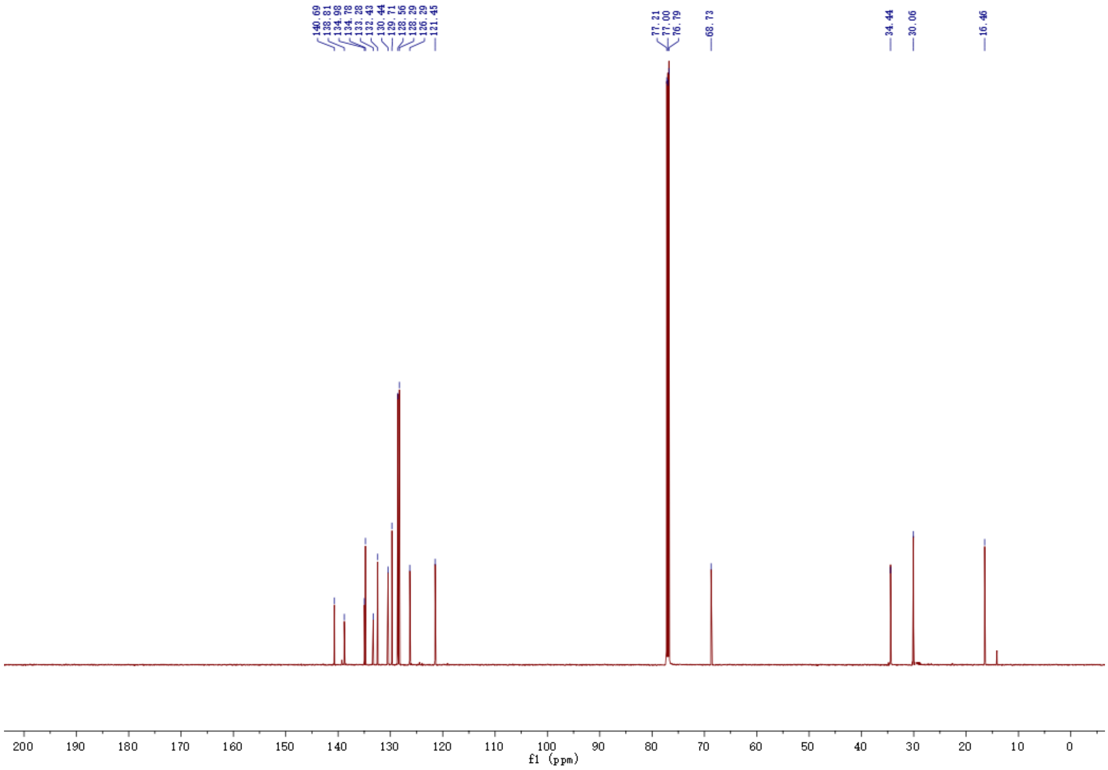

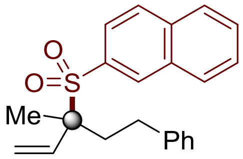

3aq

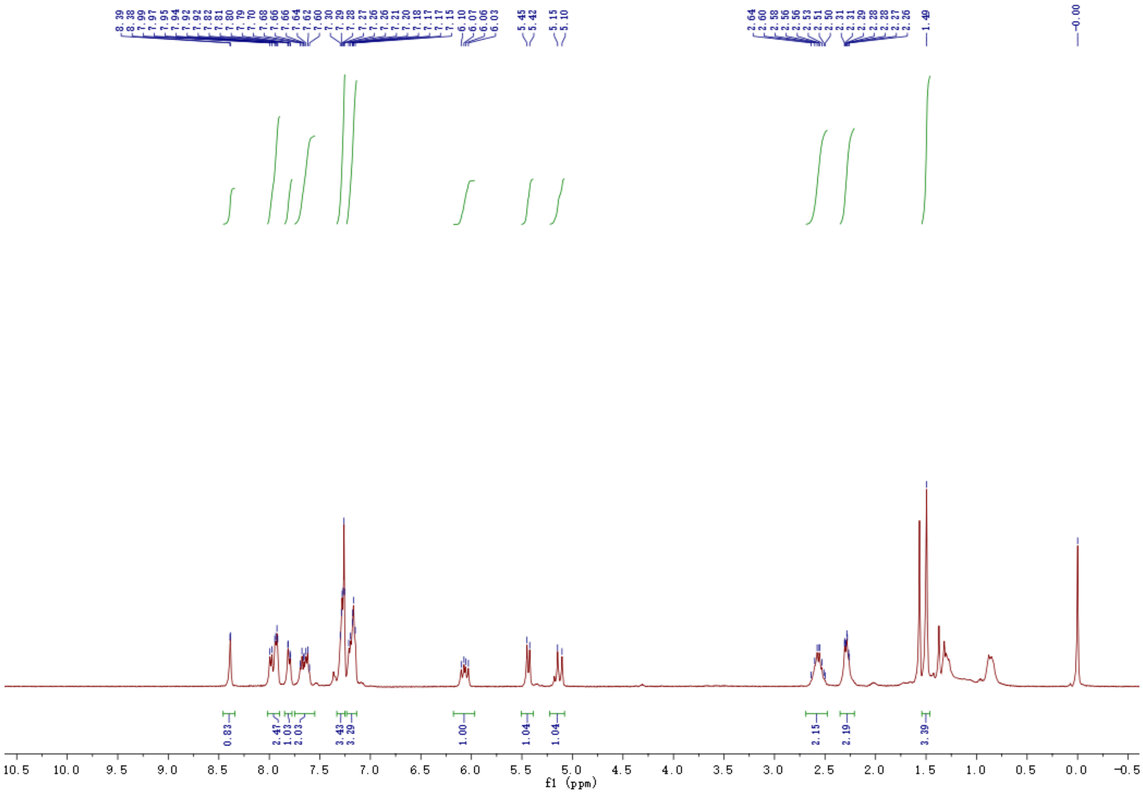

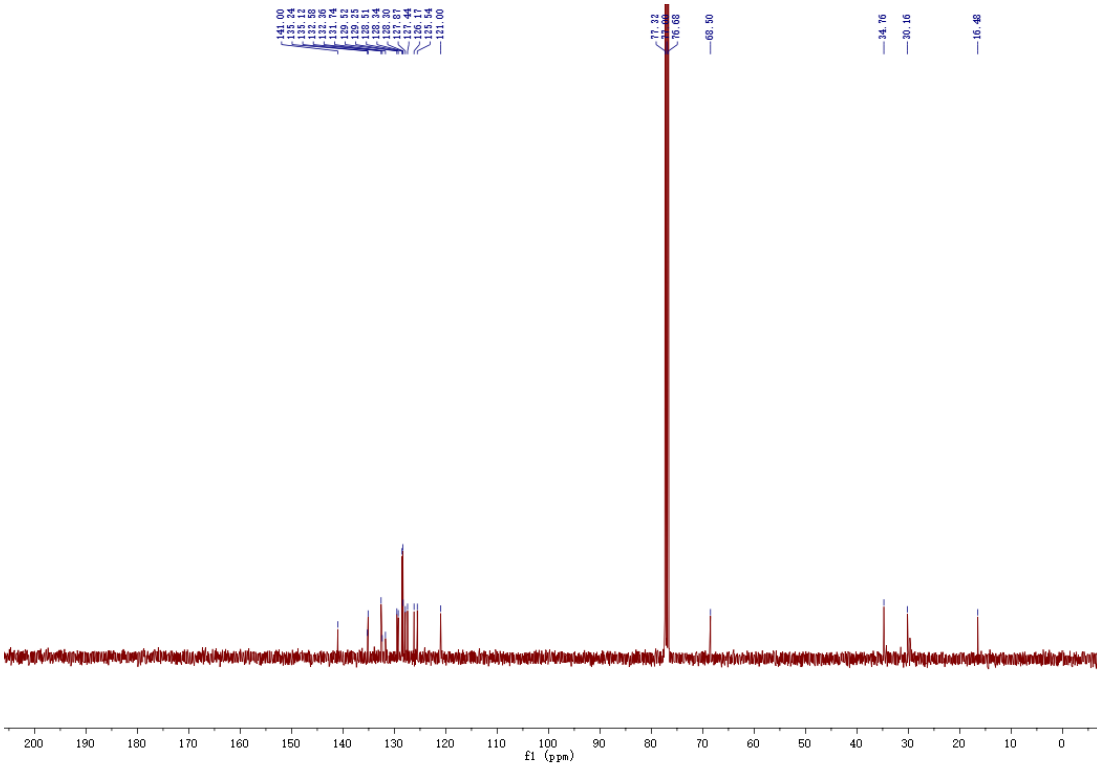

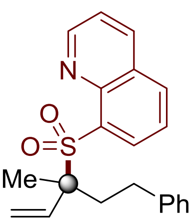
N
O
O=S
Me
Ph

3ar

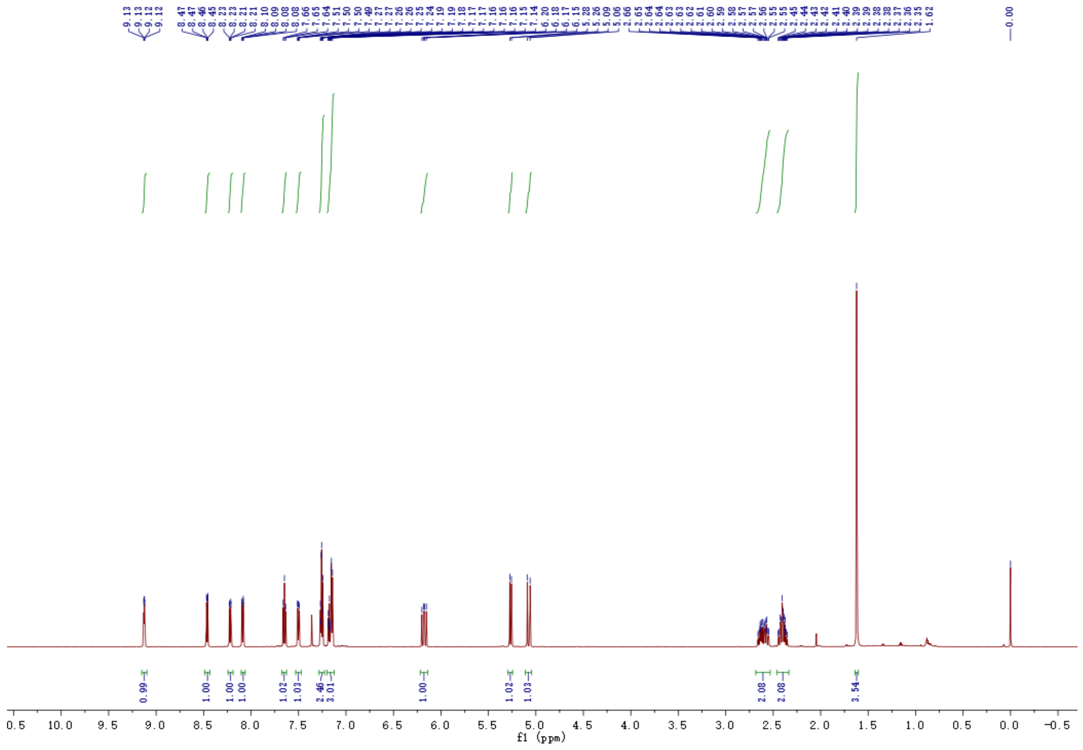
f1 (ppm)

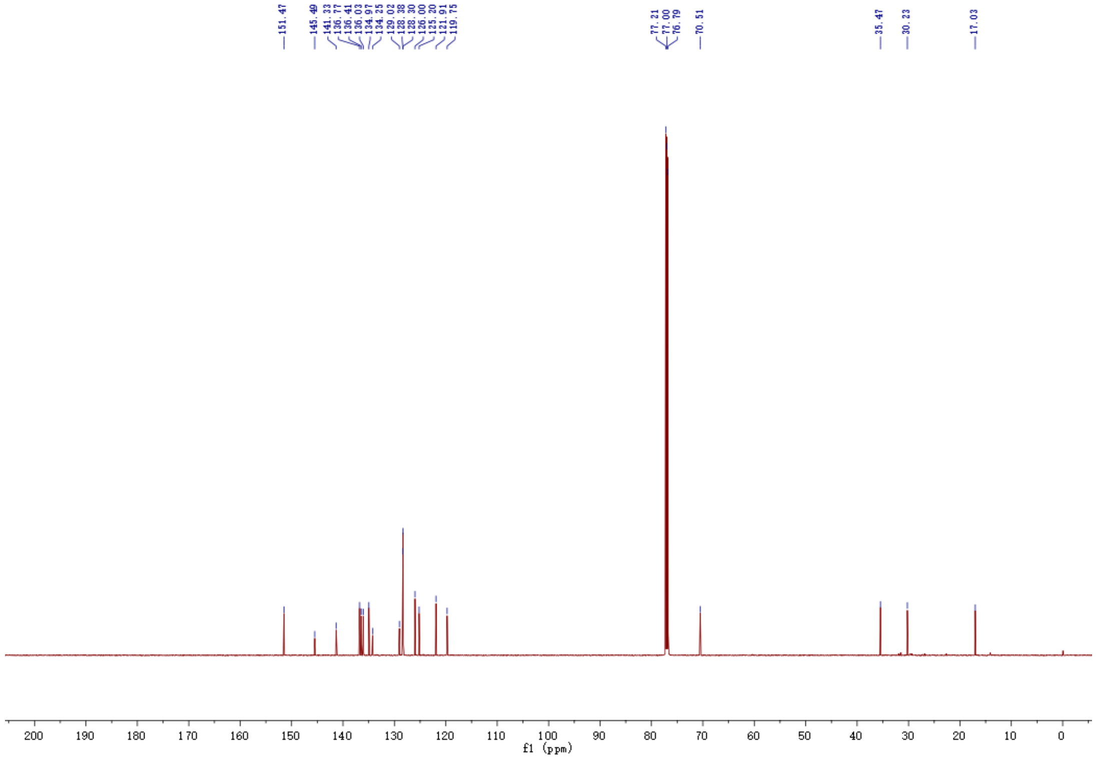
151.47
145.49
141.33
136.77
136.41
136.03
134.97
134.25
129.02
128.38
128.30
126.00
125.20
121.91
119.75
77.21
77.00
76.79
70.51
35.47
30.23
17.03
f1 (ppm)

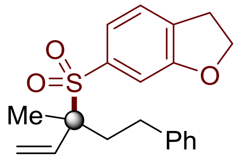
O
O=S
Me
Ph
O

3as

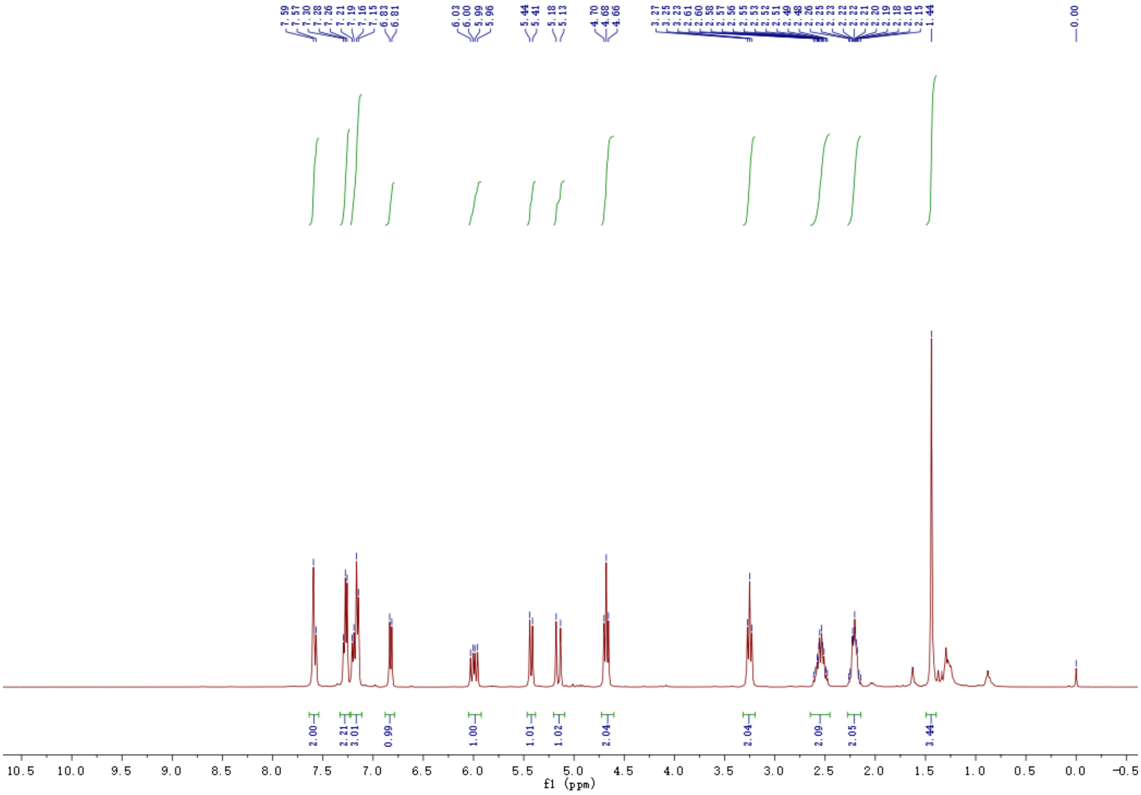
7.59
7.57
7.30
7.28
7.26
7.21
7.19
7.16
7.15
6.83
6.81
6.03
6.00
5.99
5.96
5.44
5.41
5.18
5.13
4.70
4.68
4.66
3.27
3.25
3.23
2.61
2.60
2.58
2.57
2.56
2.55
2.53
2.52
2.51
2.49
2.48
2.26
2.25
2.23
2.22
2.22
2.21
2.20
2.19
2.18
2.16
2.15
1.44
0.00
2.00
2.21
3.01
0.99
1.00
1.01
1.02
2.04
2.04
2.09
2.05
3.44
f1 (ppm)

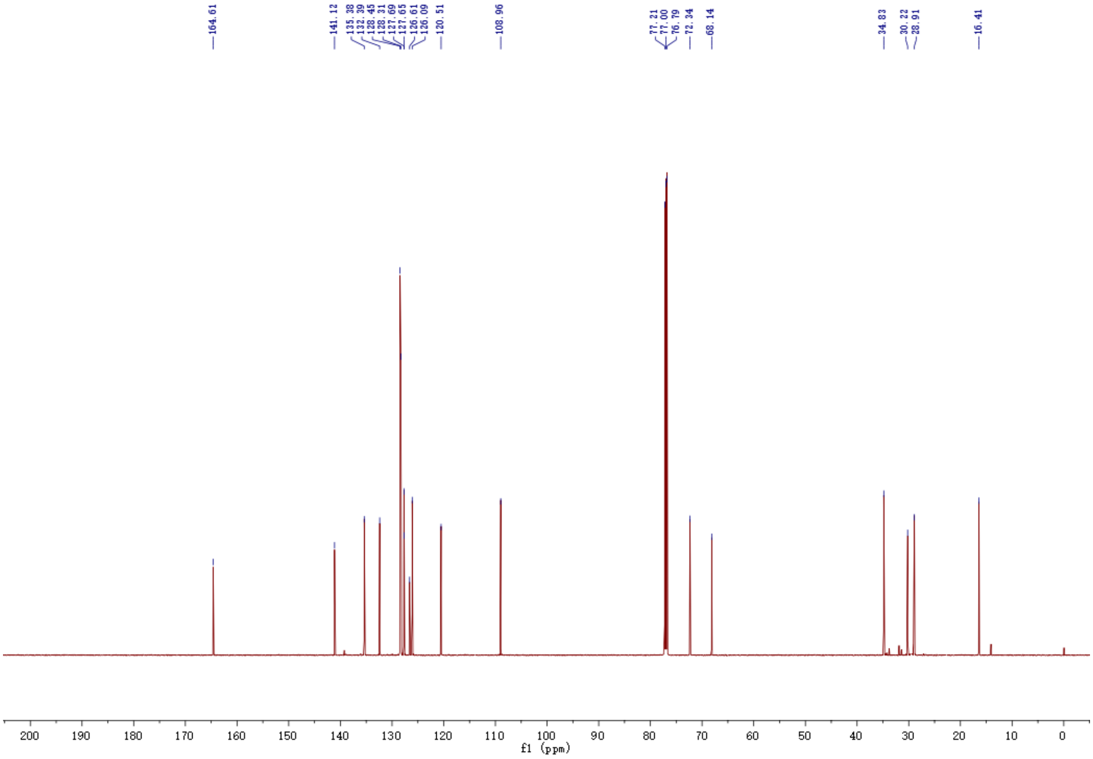
164.61
141.12
135.38
132.39
128.45
128.31
127.69
127.65
126.61
126.09
120.51
108.96
77.21
77.00
76.79
72.34
68.14
34.83
30.22
28.91
16.41
f1 (ppm)

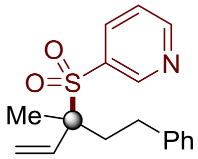

3at

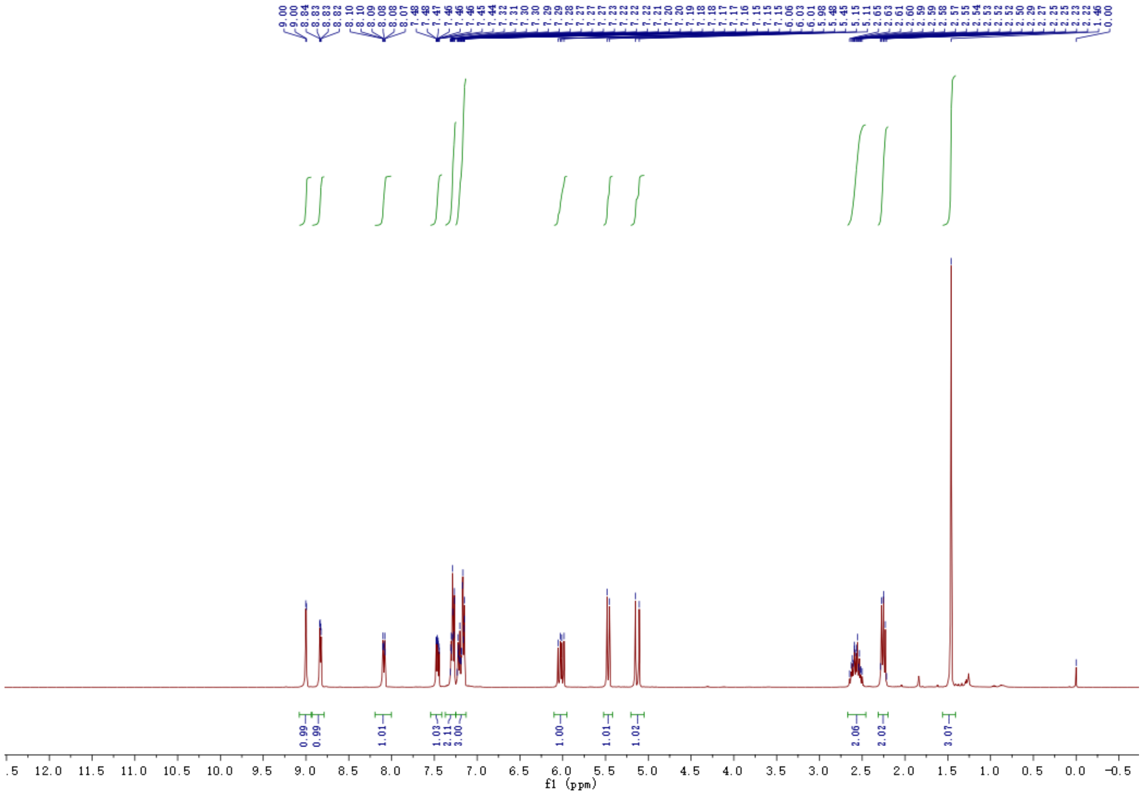

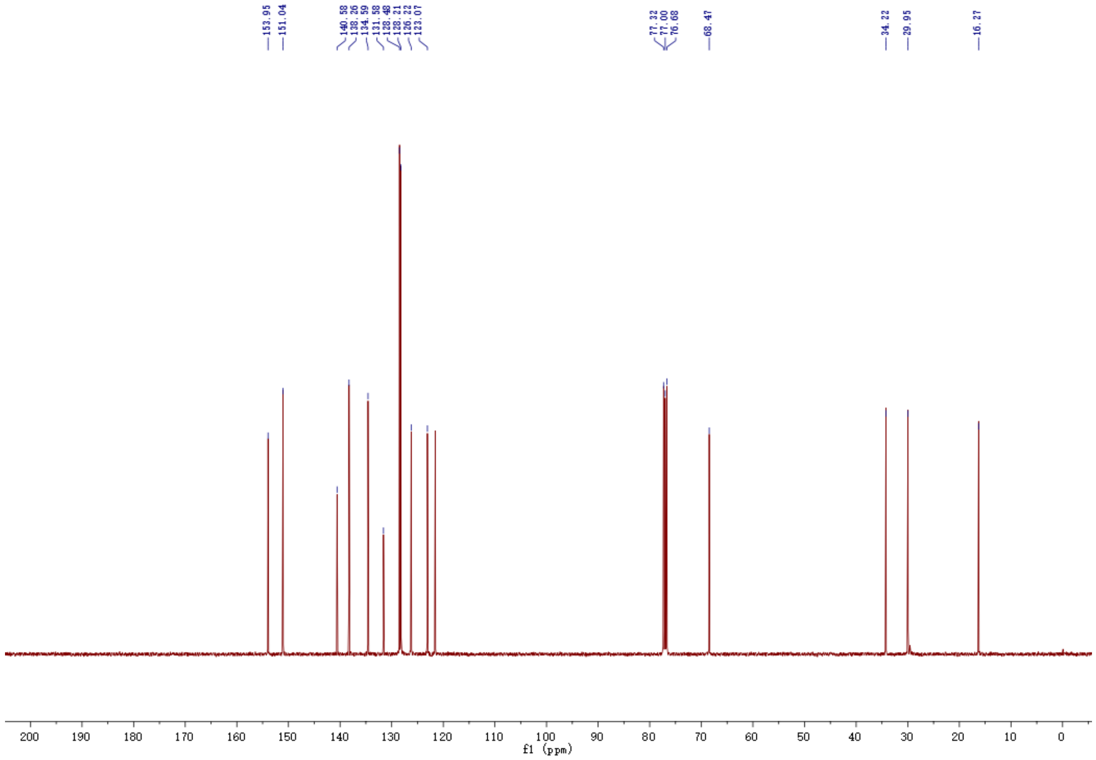

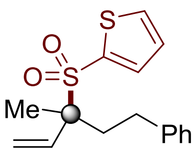

3au

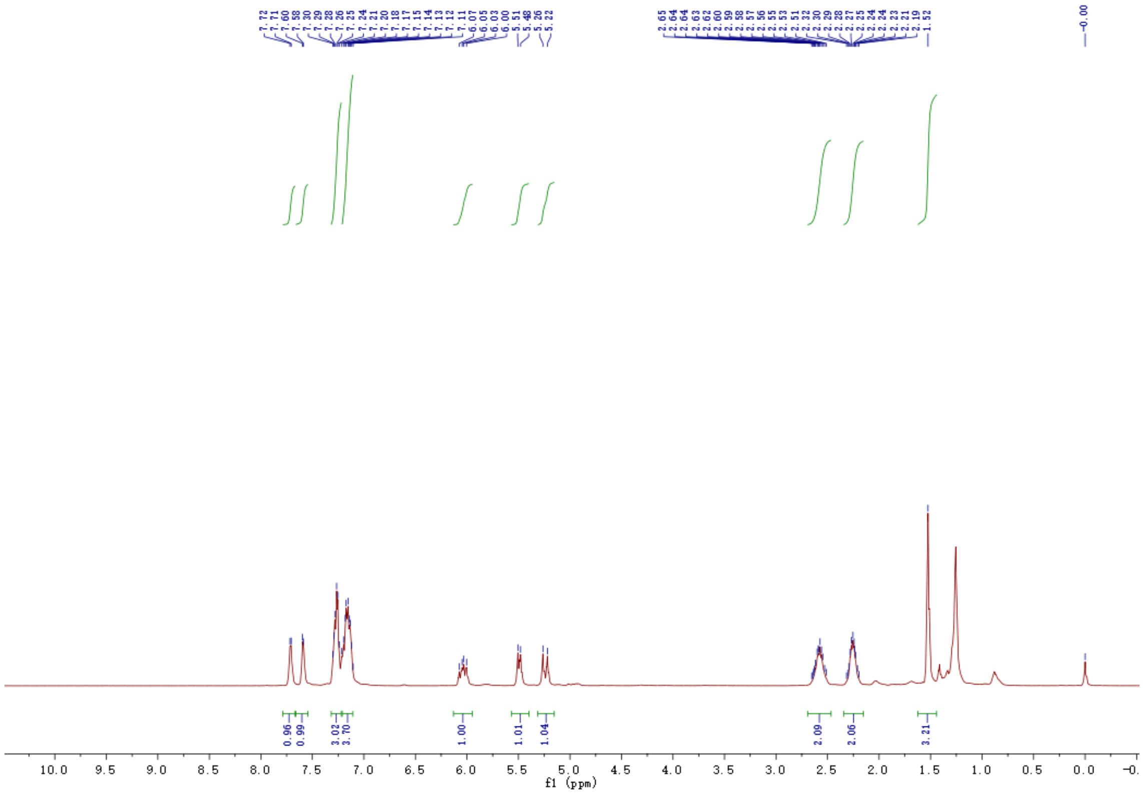

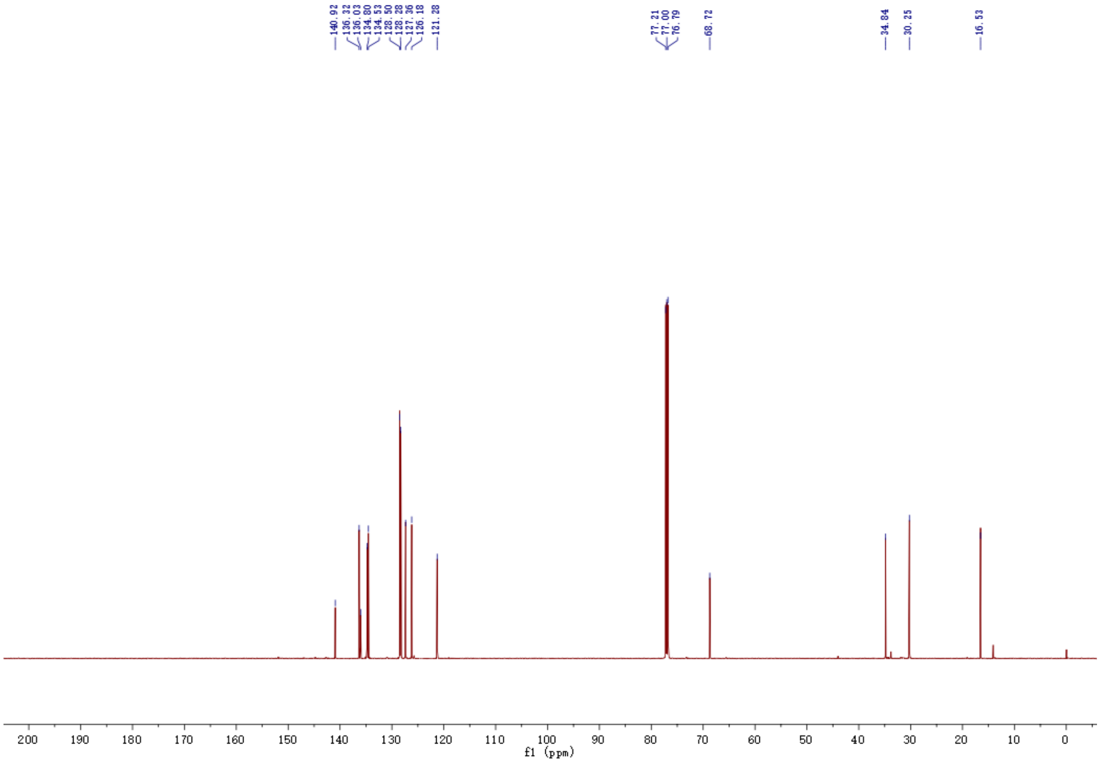

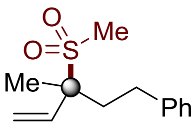
O
O=S
Me
Me
Ph

3av

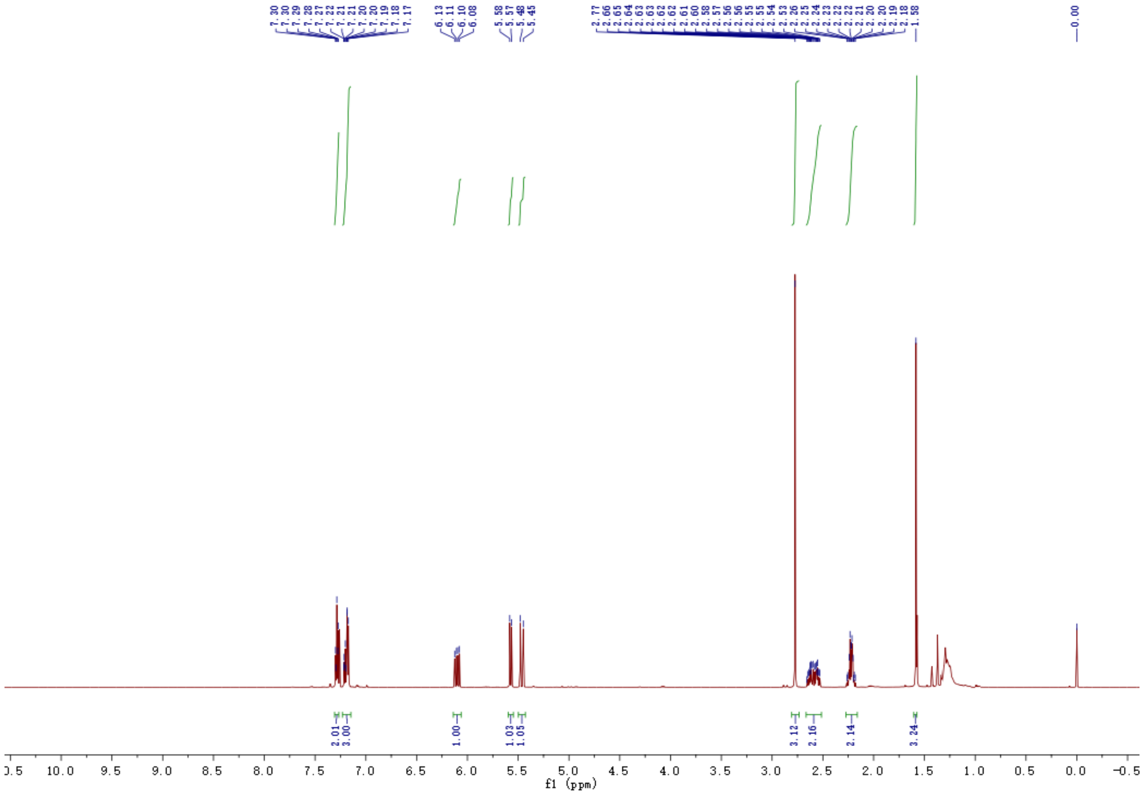
f1 (ppm)

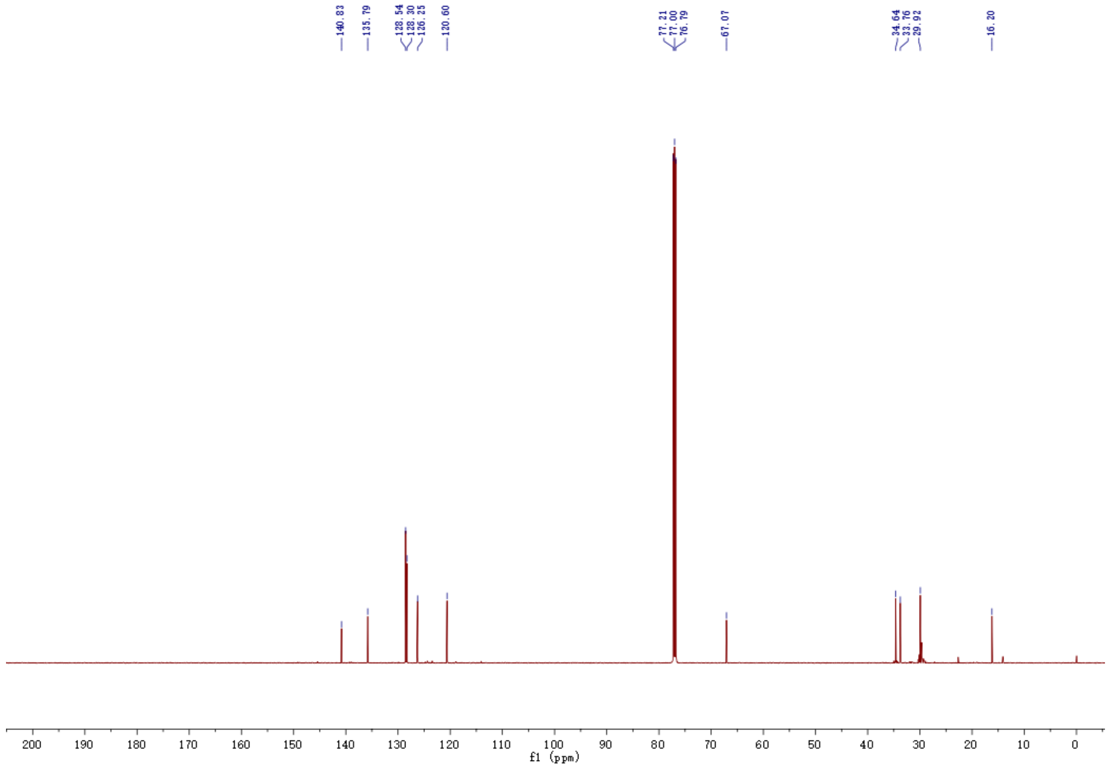
140.83
135.79
128.54
128.30
126.25
120.60
77.21
77.00
76.79
67.07
34.64
33.76
29.92
16.20
f1 (ppm)

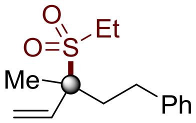

3aw

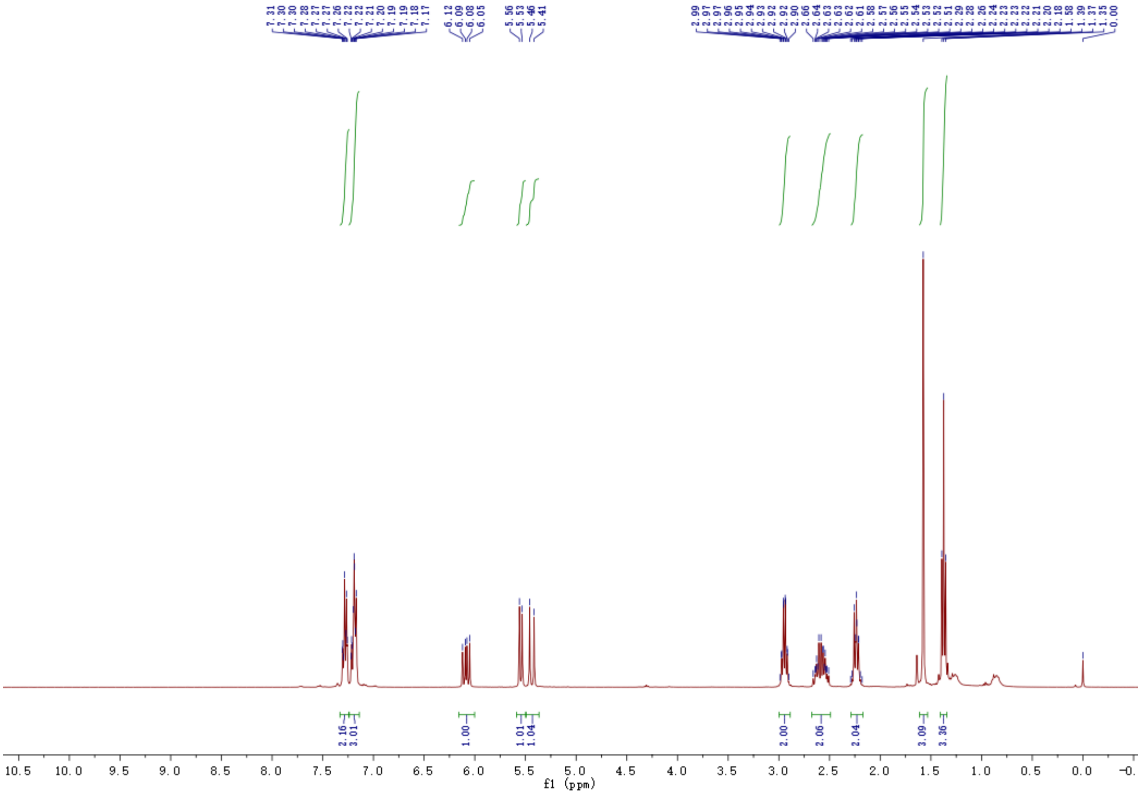

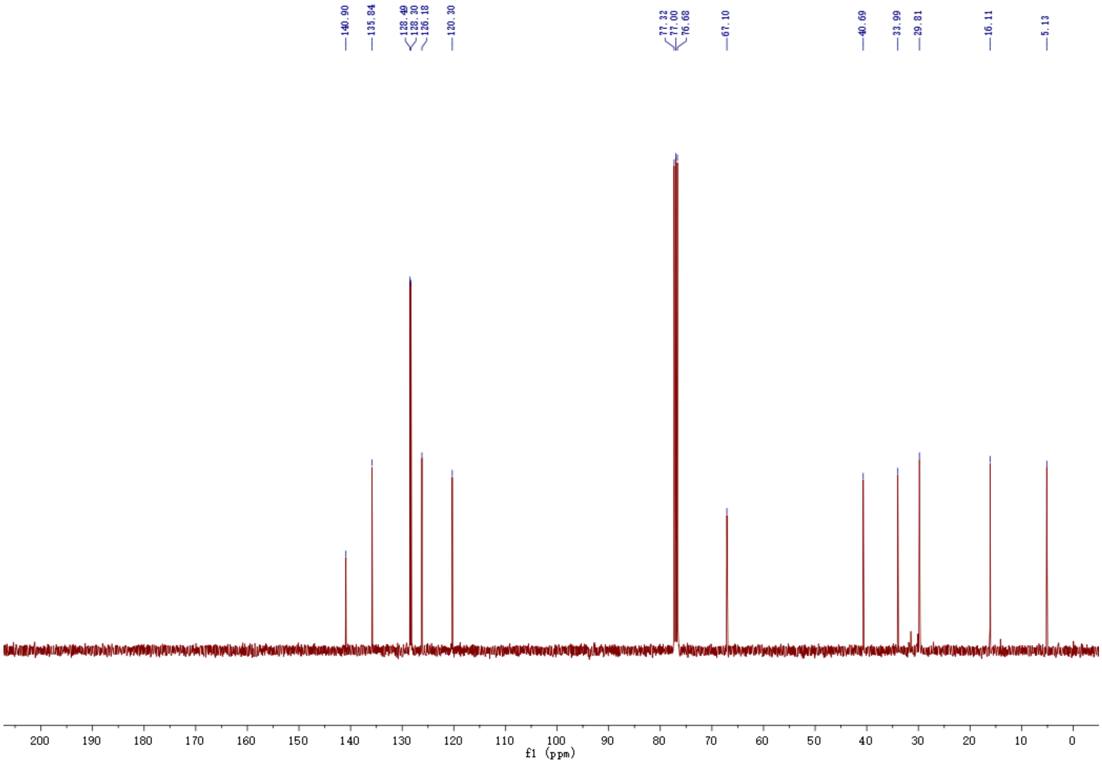

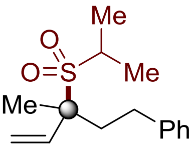
Me
O
O=S
Me
Me
Ph

3ax

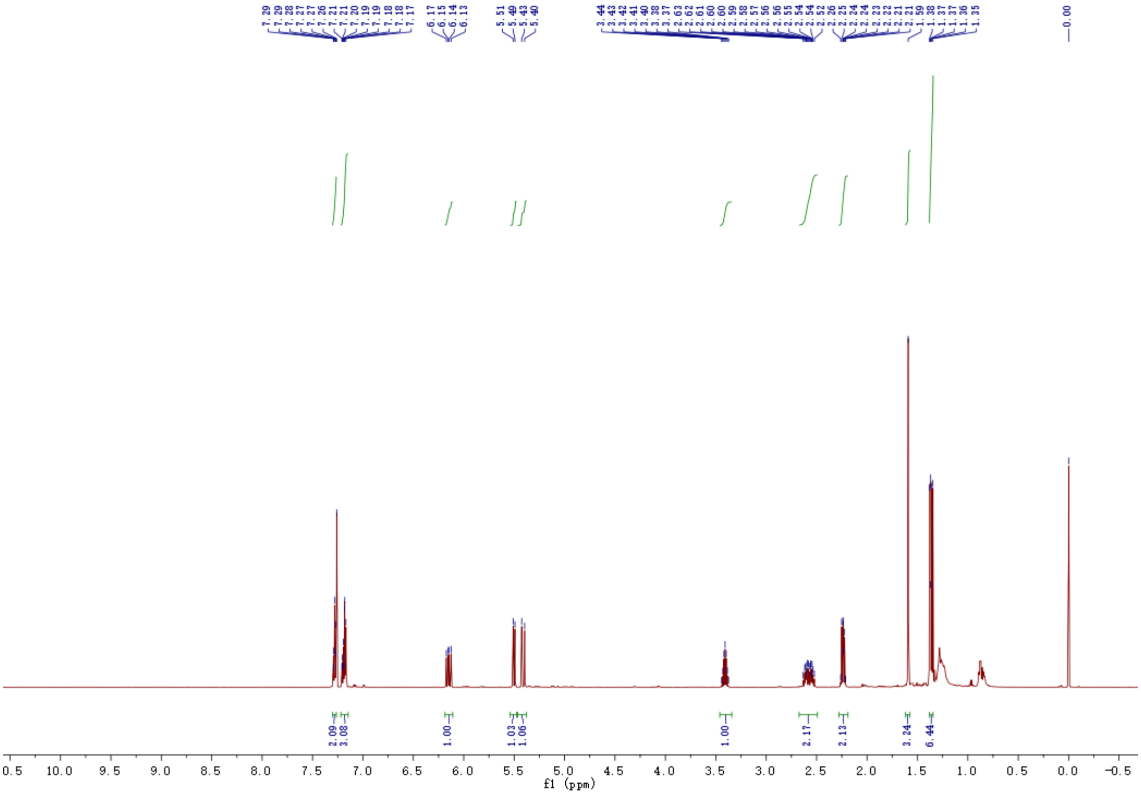
2.09
3.08
1.00
1.03
1.06
1.00
2.17
2.13
3.24
6.44
f1 (ppm)

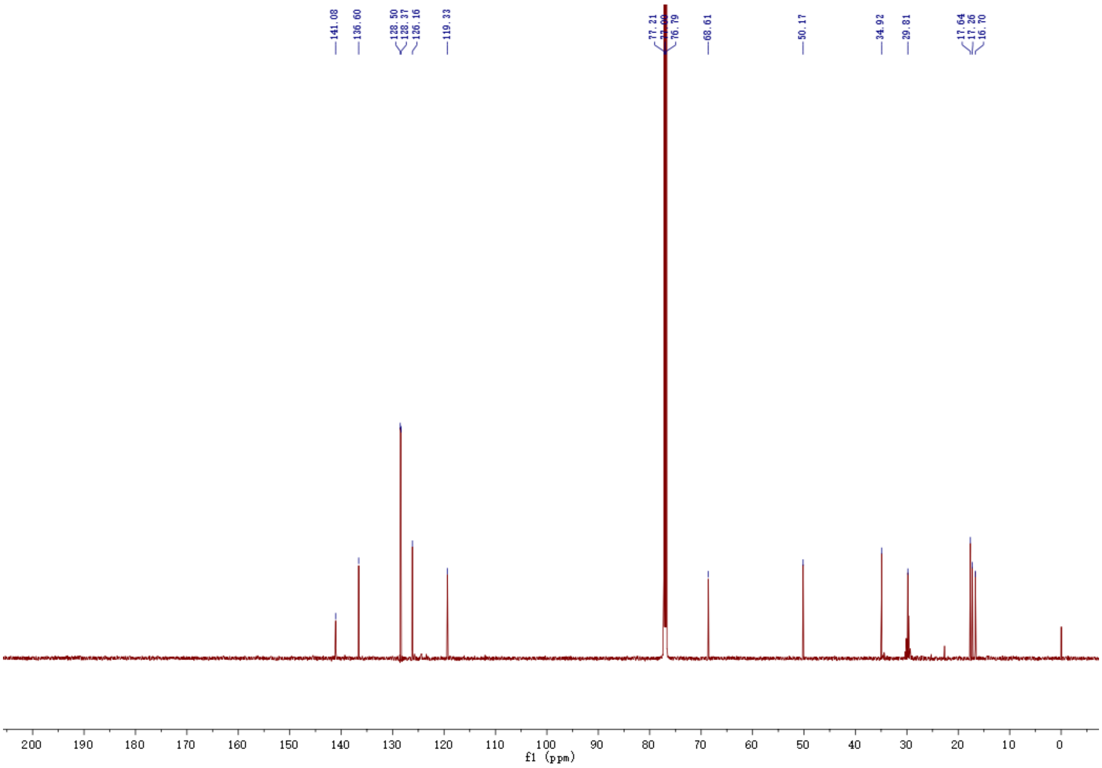
141.08
136.60
128.50
128.37
126.16
119.33
77.21
76.79
68.61
50.17
34.92
29.81
17.64
17.26
16.70
f1 (ppm)

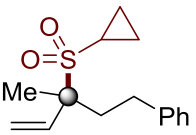

3ay

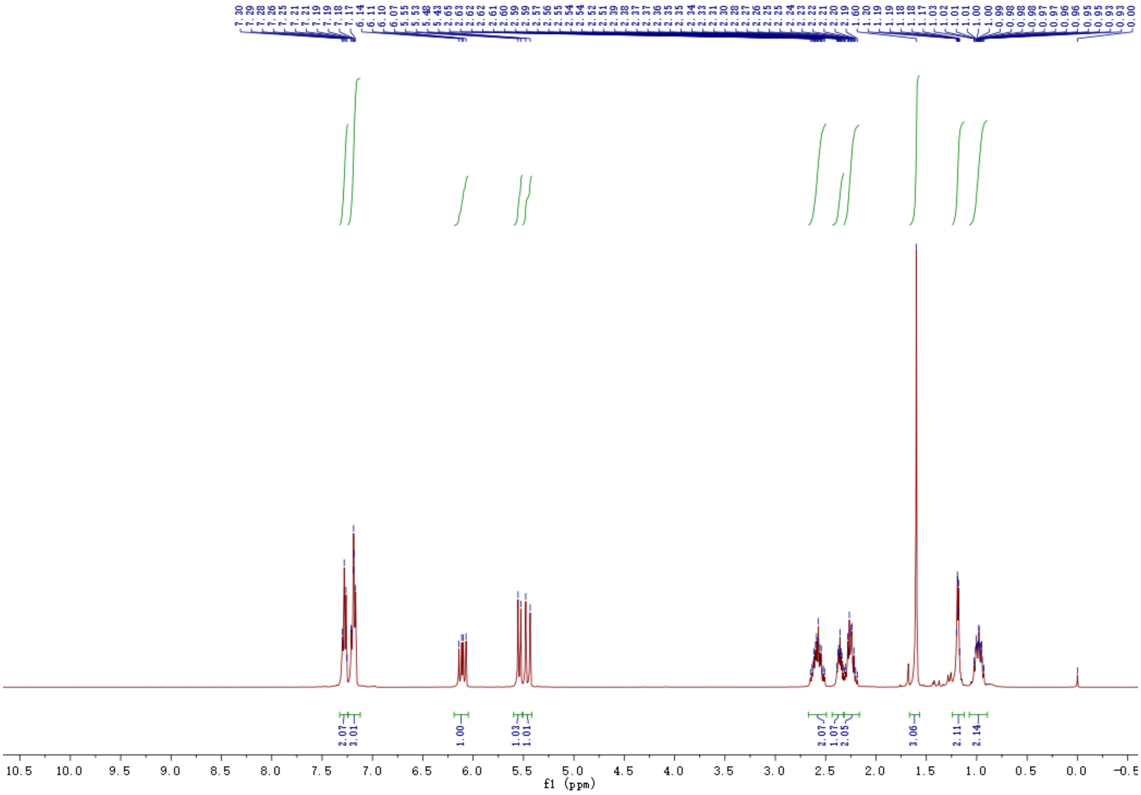

2.07
3.01
1.00
1.03
1.01
2.07
1.07
2.05
3.06
2.11
2.14
10.5 10.0 9.5 9.0 8.5 8.0 7.5 7.0 6.5 6.0 5.5 5.0 4.5 4.0 3.5 3.0 2.5 2.0 1.5 1.0 0.5 0.0 -0.5
f1 (ppm)

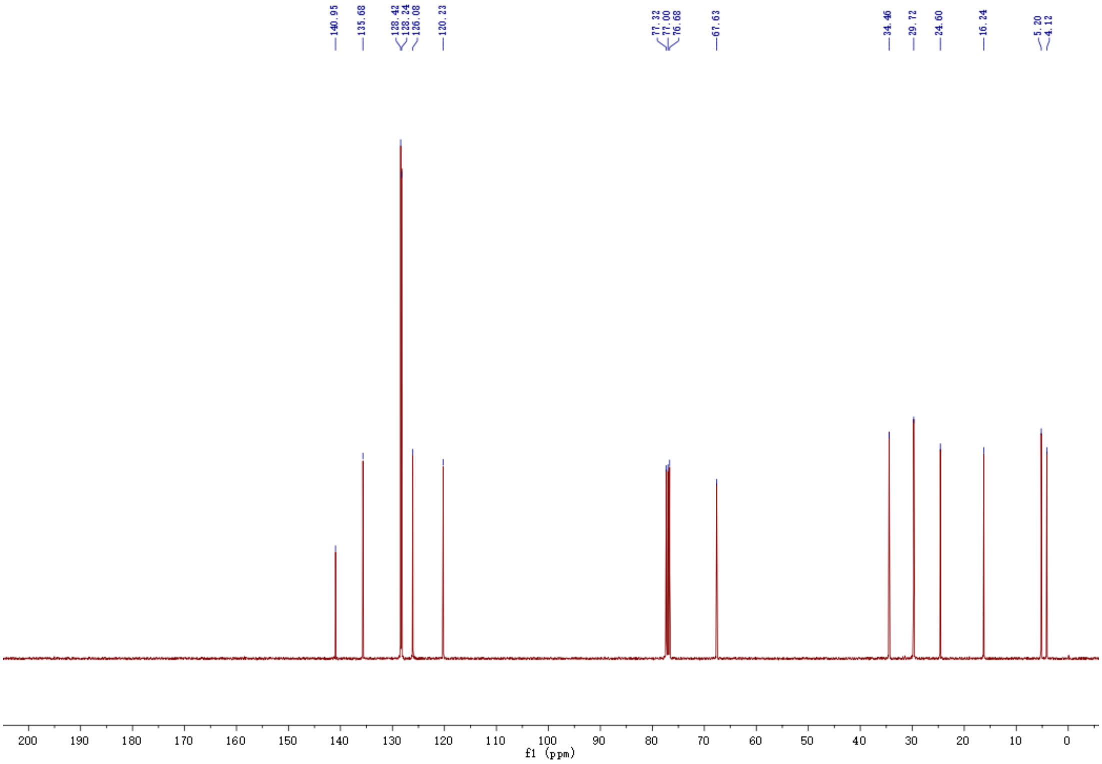

140.95
135.68
128.42
128.24
126.08
120.23
77.32
77.00
76.68
67.63
34.46
29.72
24.60
16.24
5.20
4.12
200 190 180 170 160 150 140 130 120 110 100 90 80 70 60 50 40 30 20 10 0
f1 (ppm)

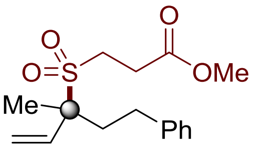

O
O=S
OMe
Me
Ph

3az

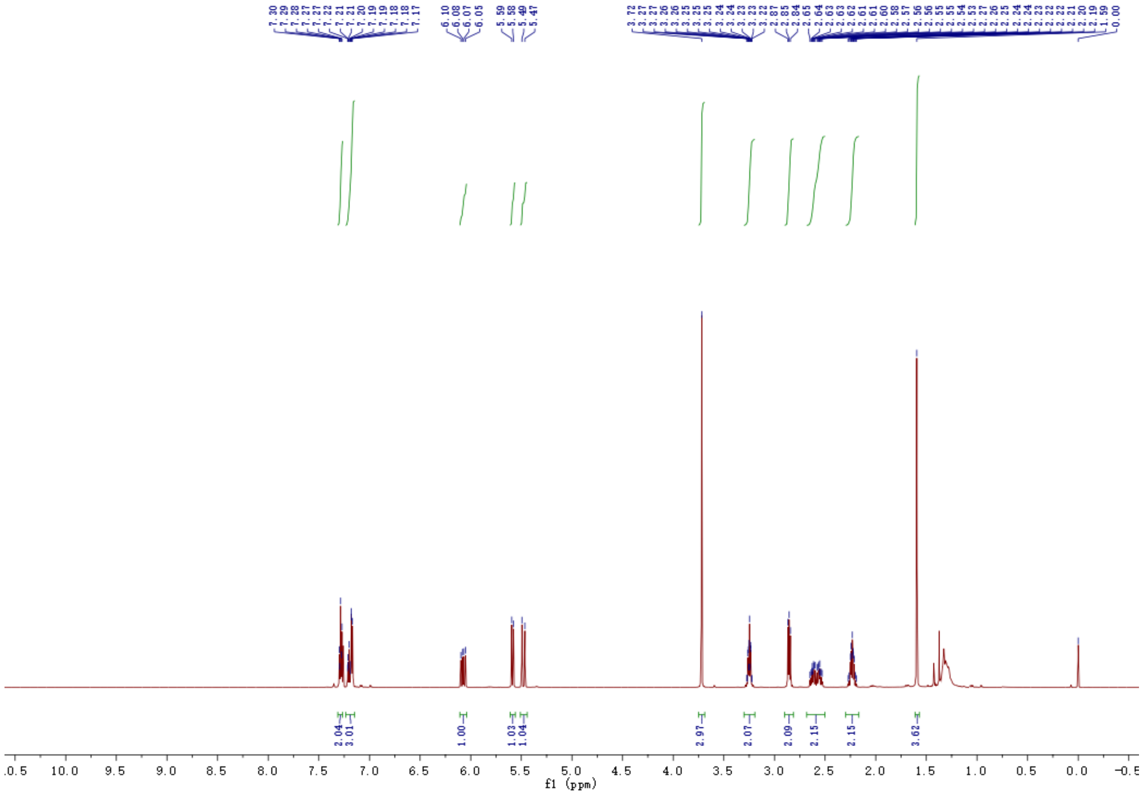

2.04
3.01
1.00
1.03
1.04
2.97
2.07
2.09
2.15
2.15
3.62
f1 (ppm)

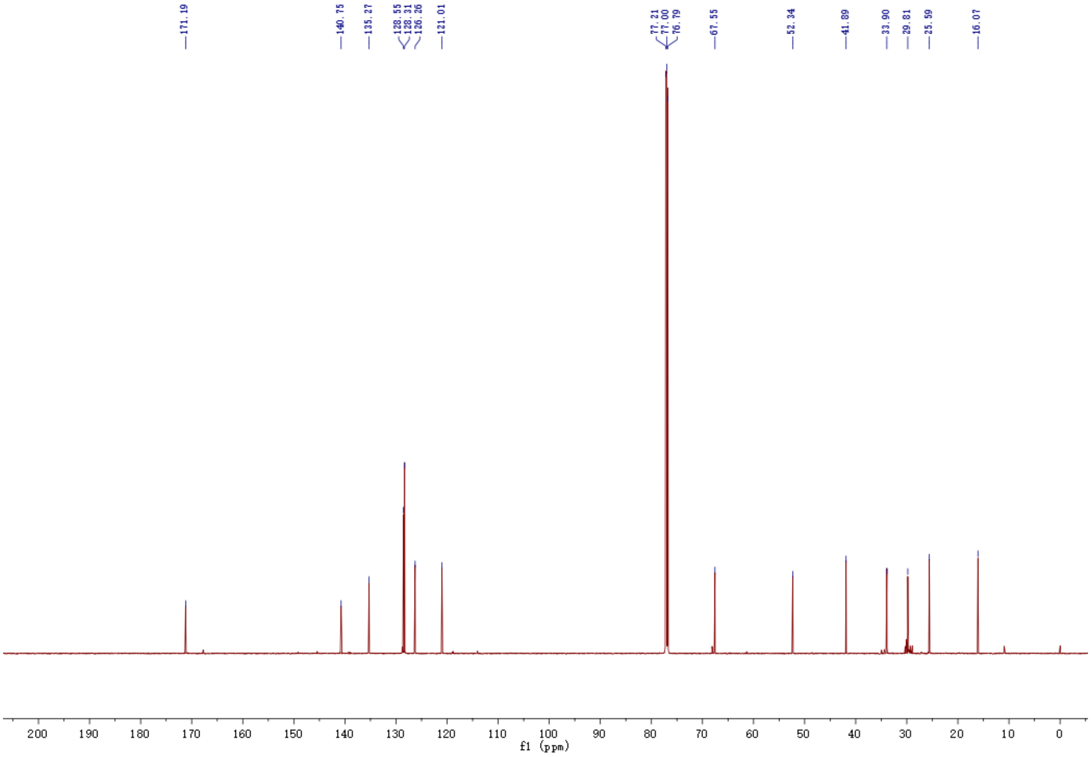

171.19
140.75
135.27
128.55
128.31
126.26
121.01
77.21
77.00
76.79
67.55
52.34
41.89
33.90
29.81
25.59
16.07
f1 (ppm)

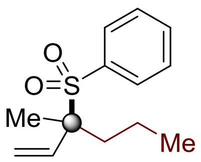
O
O=S
Me
Me

3ba

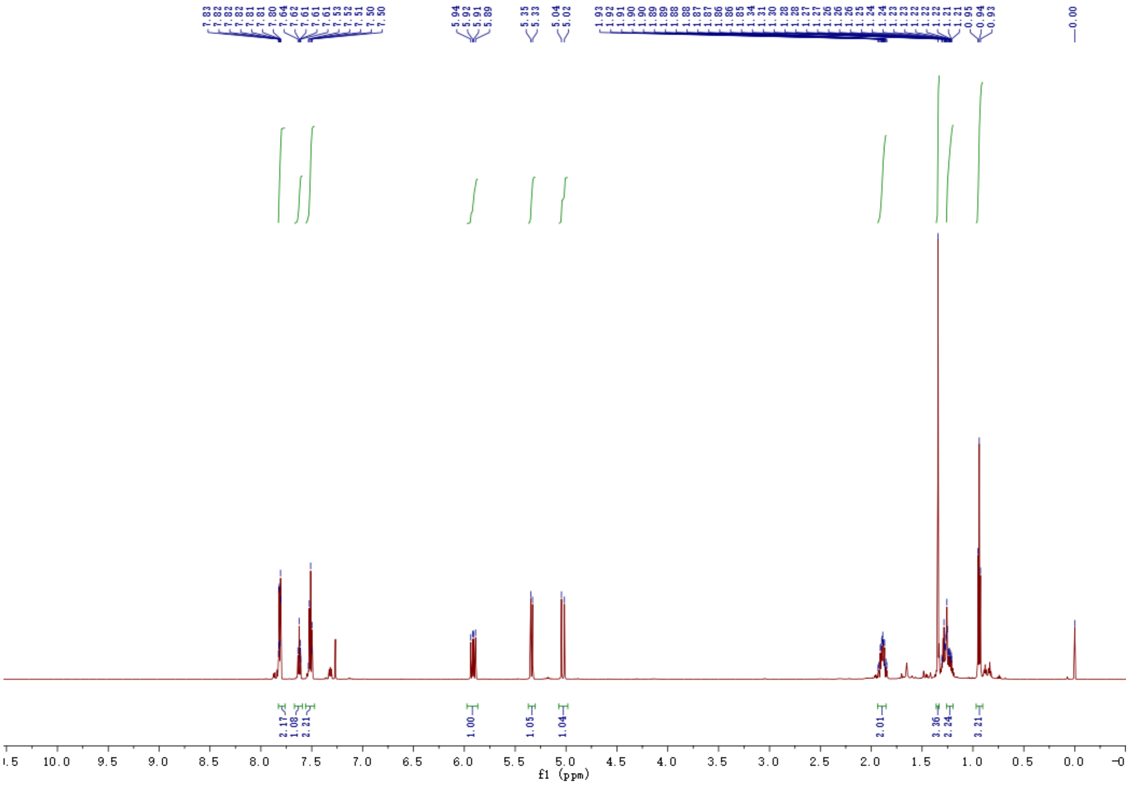
2.17
1.08
2.21
1.00
1.05
1.04
2.01
3.36
2.24
3.21
f1 (ppm)

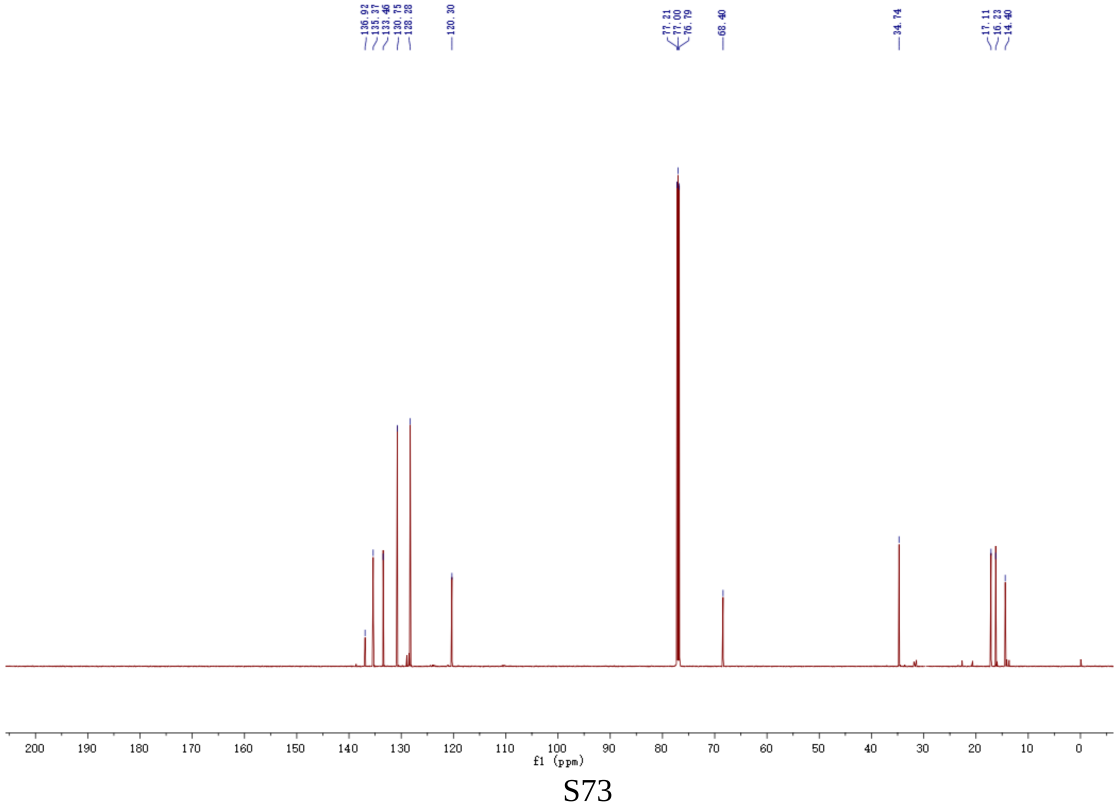
136.92
135.37
133.46
130.75
128.28
120.30
77.21
77.00
76.79
68.40
34.74
17.11
16.23
14.40
f1 (ppm)

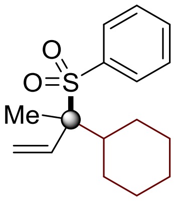

3ca

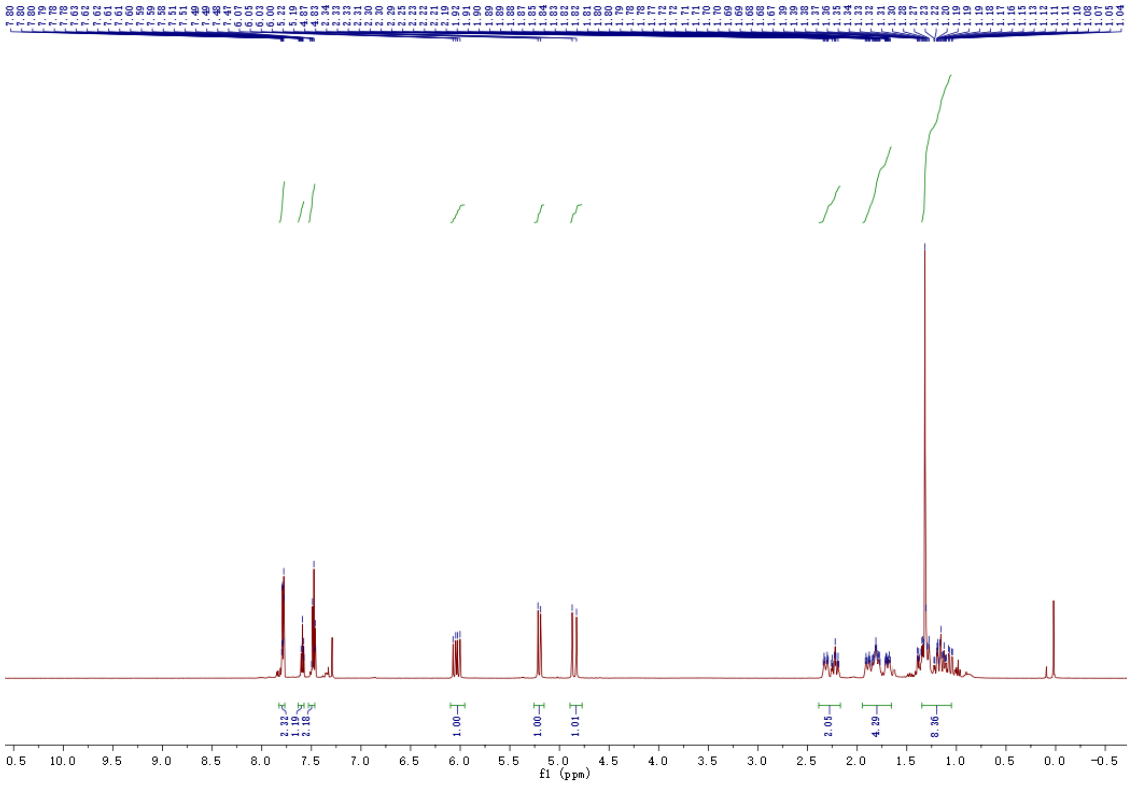

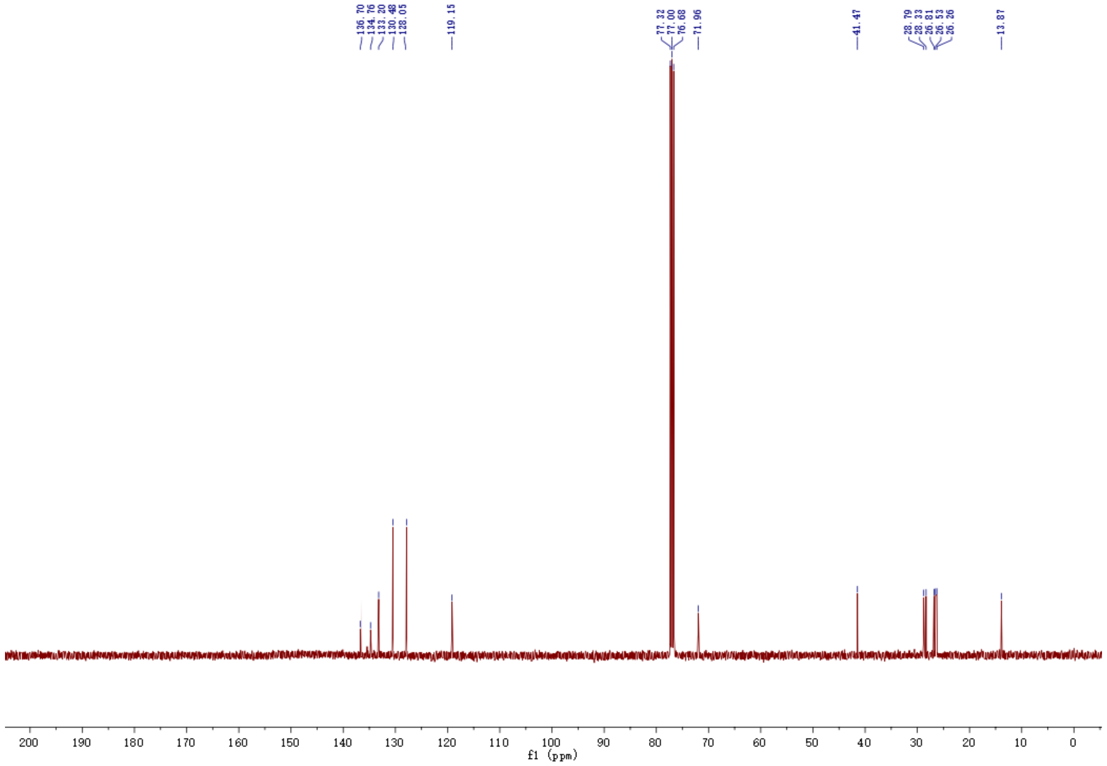

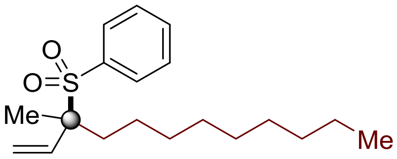
O
O=S
Me
Me

3da

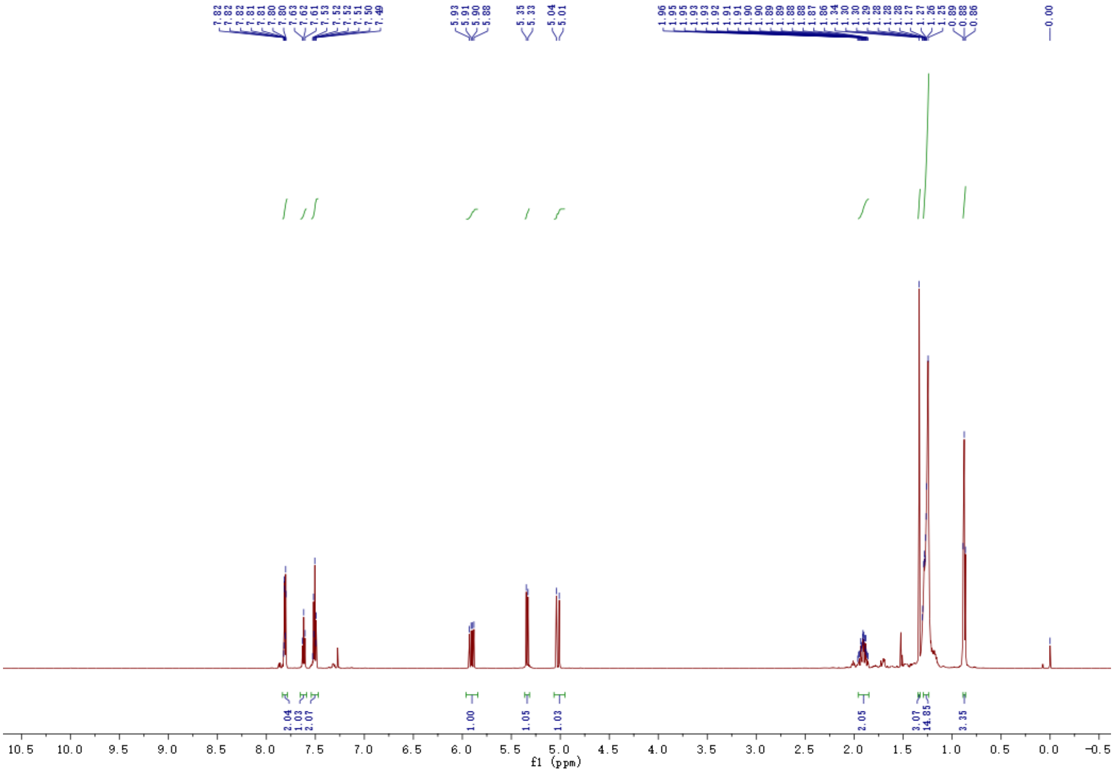
7.82 7.82 7.81 7.81 7.80 7.80 7.63 7.62 7.61 7.53 7.52 7.52 7.51 7.50 7.49
5.93 5.91 5.90 5.88
5.35 5.33
5.04 5.01
1.96 1.95 1.95 1.93 1.92 1.91 1.91 1.90 1.90 1.89 1.89 1.88 1.88 1.87 1.86 1.34 1.30 1.30 1.29 1.28 1.28 1.28 1.27 1.27 1.26 1.25 0.89 0.88 0.86
0.00
2.04 1.03 2.07 1.00 1.05 1.03 2.05 3.07 14.85 3.35
10.5 10.0 9.5 9.0 8.5 8.0 7.5 7.0 6.5 6.0 5.5 5.0 4.5 4.0 3.5 3.0 2.5 2.0 1.5 1.0 0.5 0.0 -0.5
f1 (ppm)

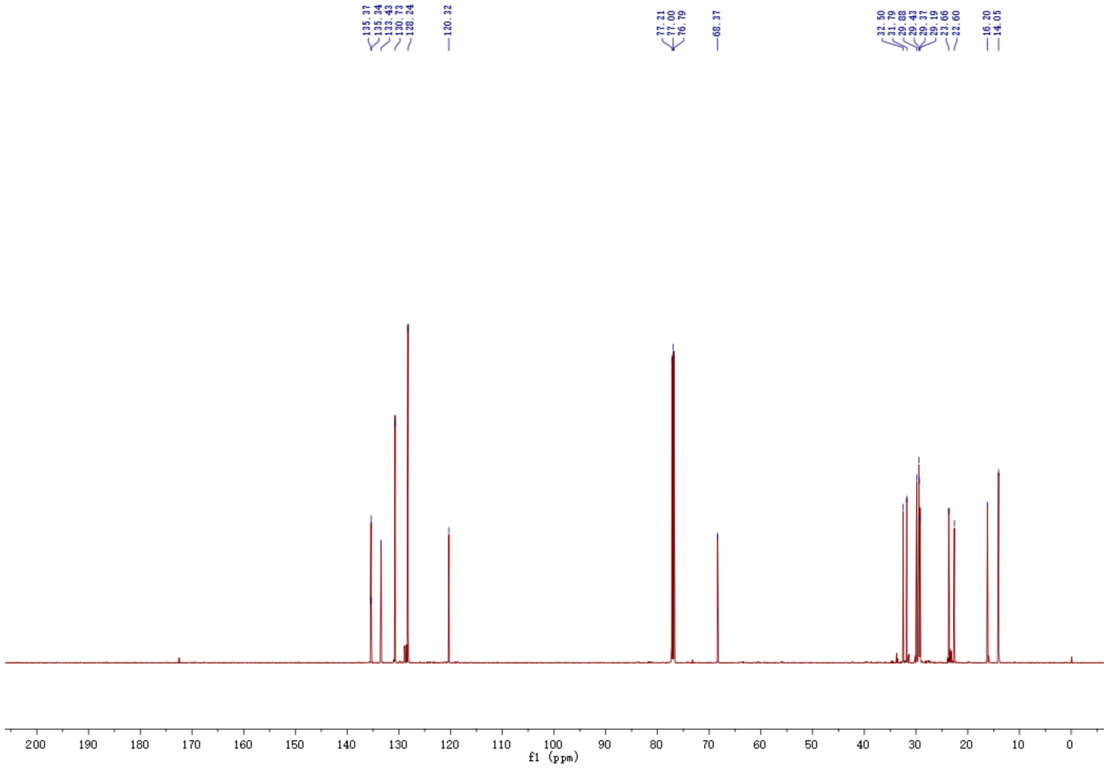
135.37 135.34 133.43 130.73 128.24
120.32
77.21 77.00 76.79
68.37
32.50 31.79 29.88 29.43 29.37 29.19 23.66 22.60
16.20 14.05
200 190 180 170 160 150 140 130 120 110 100 90 80 70 60 50 40 30 20 10 0
f1 (ppm)

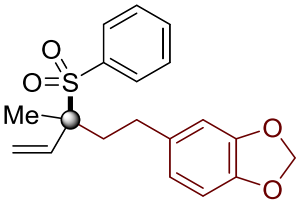

O
O=S
Me
O
O

3ea

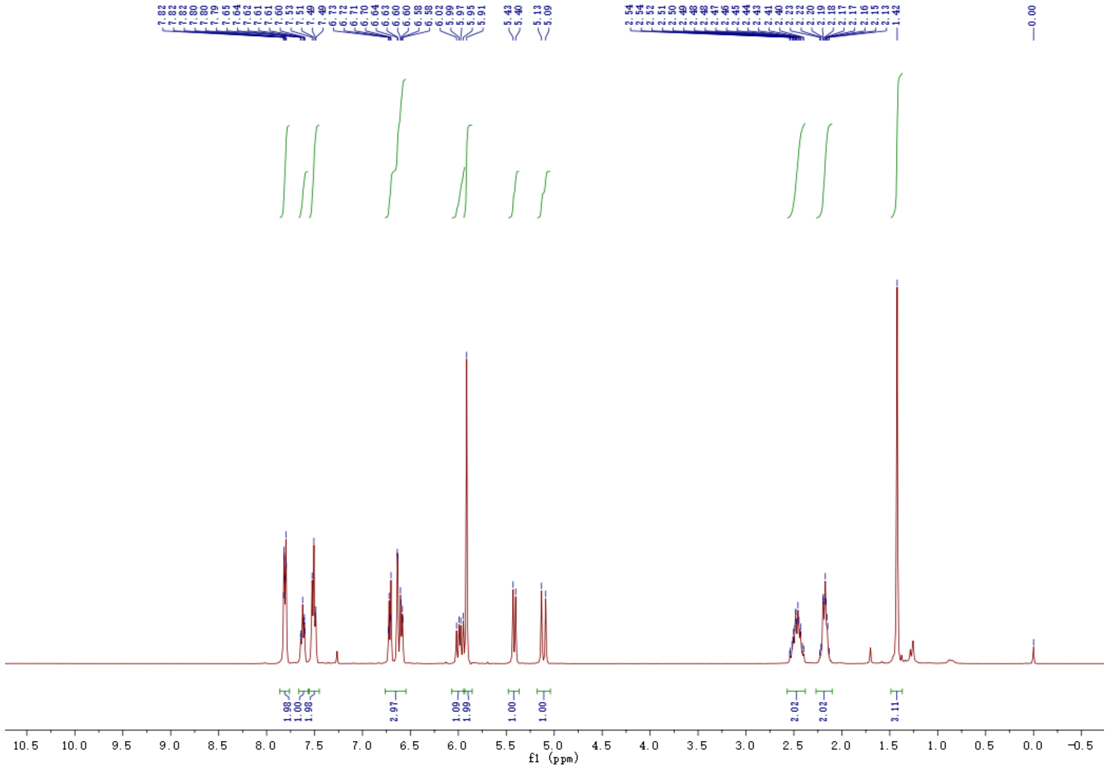

7.82 7.82 7.80 7.80 7.79 7.65 7.64 7.62 7.61 7.61 7.60 7.60 7.53 7.51 7.49 7.49 6.73 6.72 6.71 6.70 6.64 6.63 6.60 6.60 6.58 6.58 6.02 5.99 5.97 5.95 5.91 5.43 5.40 5.13 5.09
2.54 2.54 2.52 2.51 2.50 2.49 2.48 2.48 2.47 2.46 2.45 2.44 2.43 2.41 2.40 2.23 2.22 2.20 2.19 2.18 2.17 2.16 2.15 2.13 1.42
0.00
1.98 1.00 1.98 2.97 1.09 1.99 1.00 1.00 2.02 2.02 3.11
f1 (ppm)

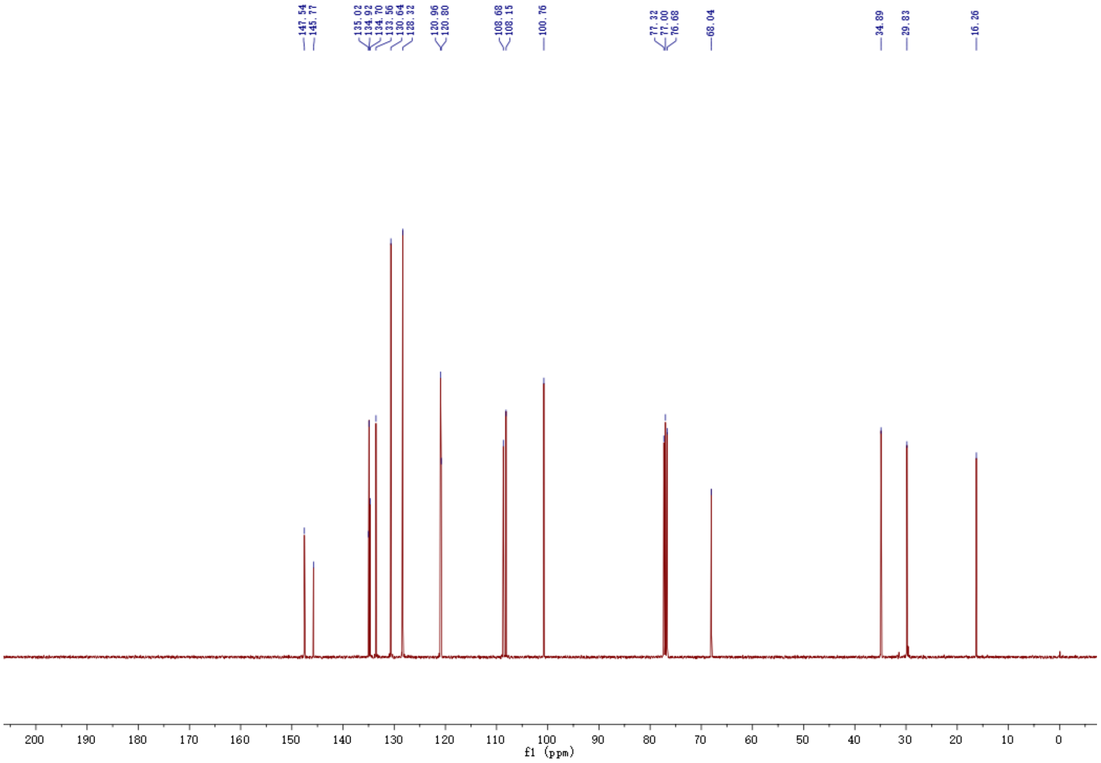

147.54 145.77
135.02 134.92 134.70 133.56 130.64 128.32
120.96 120.80
108.68 108.15
100.76
77.32 77.00 76.68
68.04
34.89
29.83
16.26
f1 (ppm)

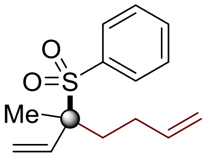

3fa

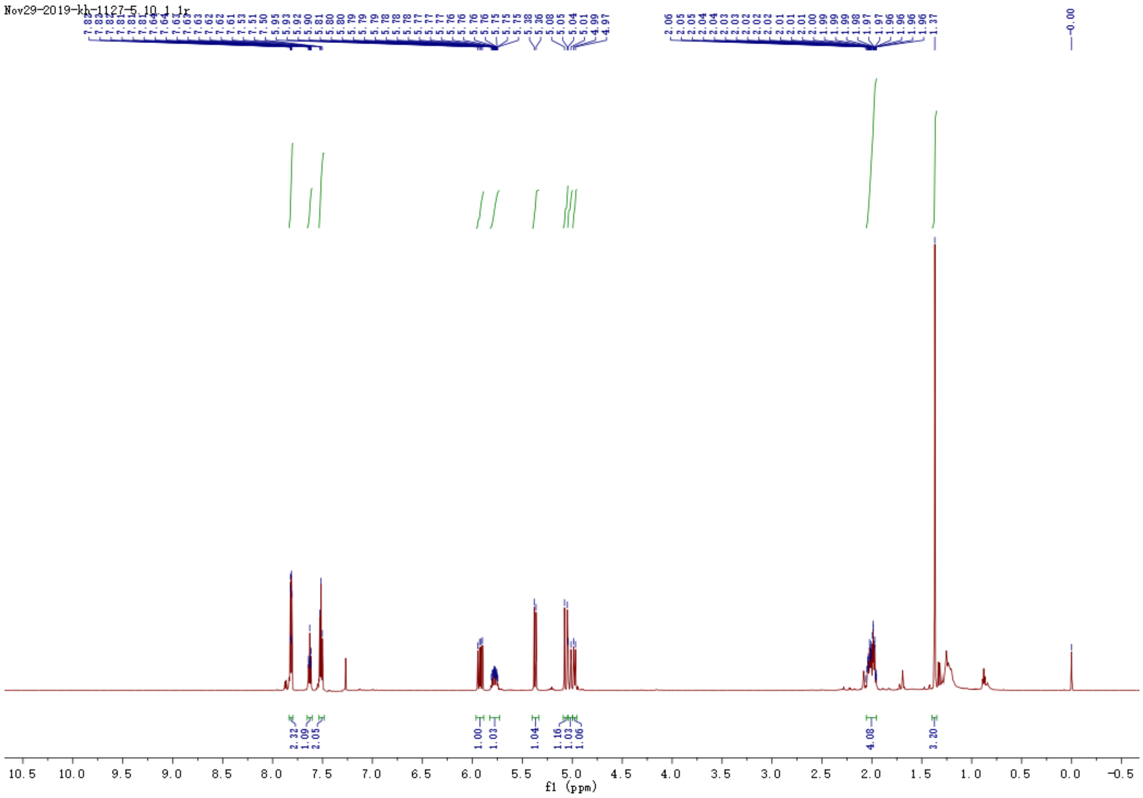

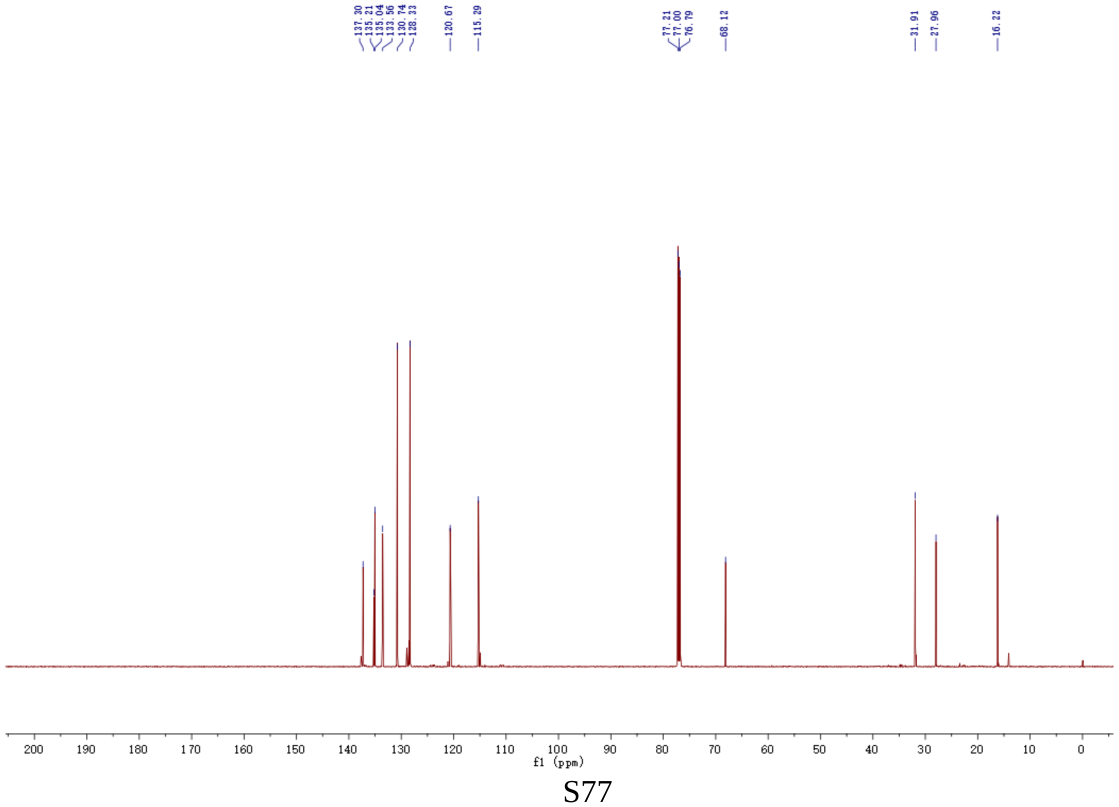

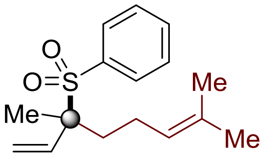

3ga

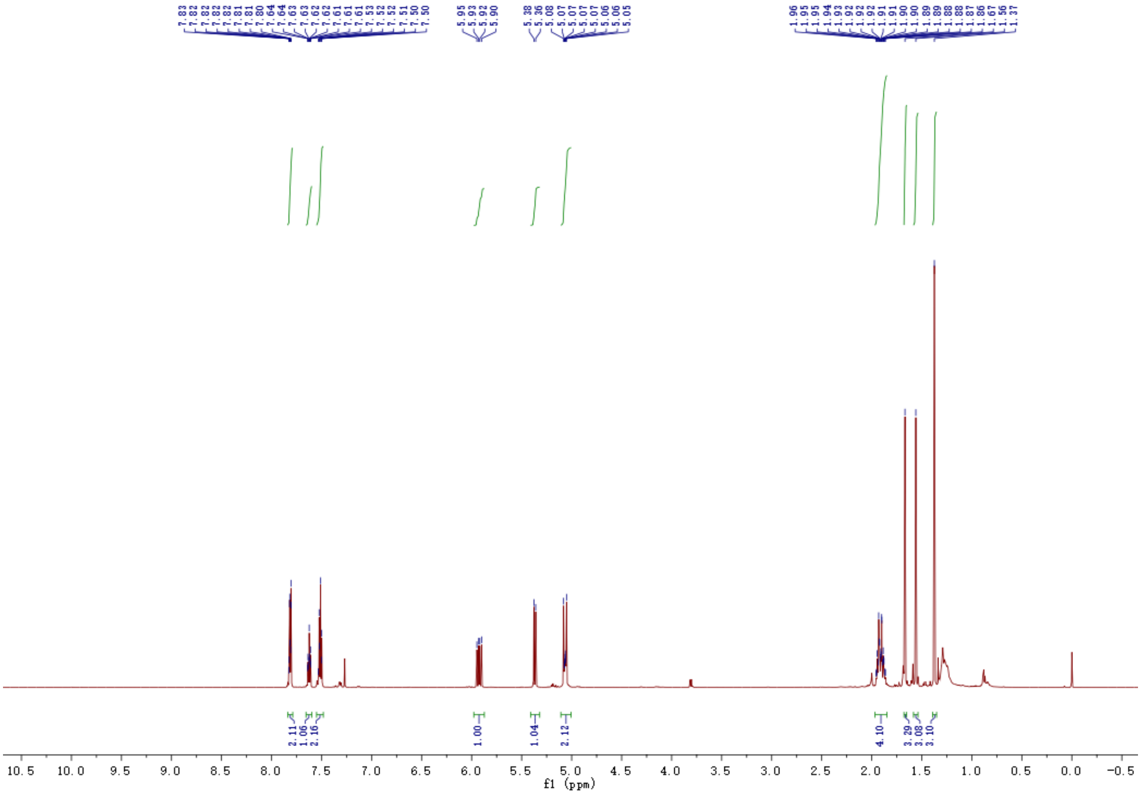

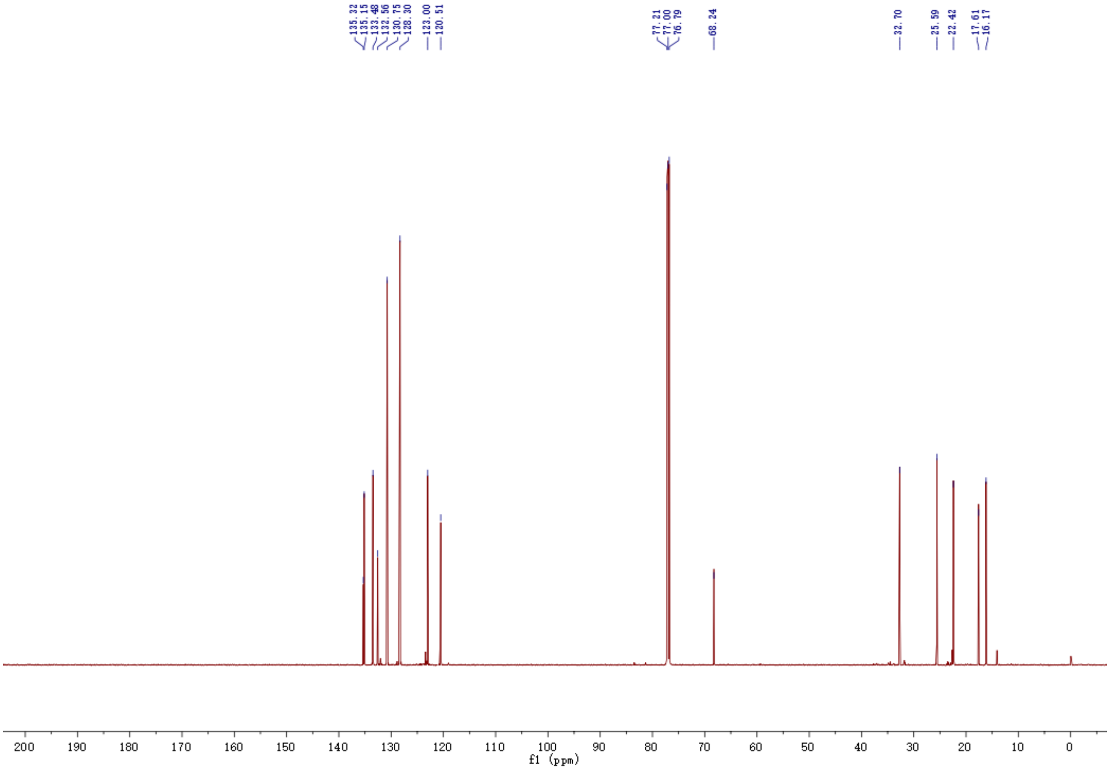

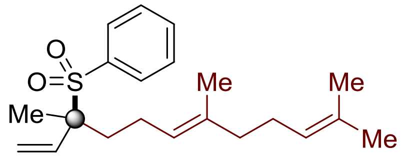

O
O=S
Me
Me
Me
Me

3ha

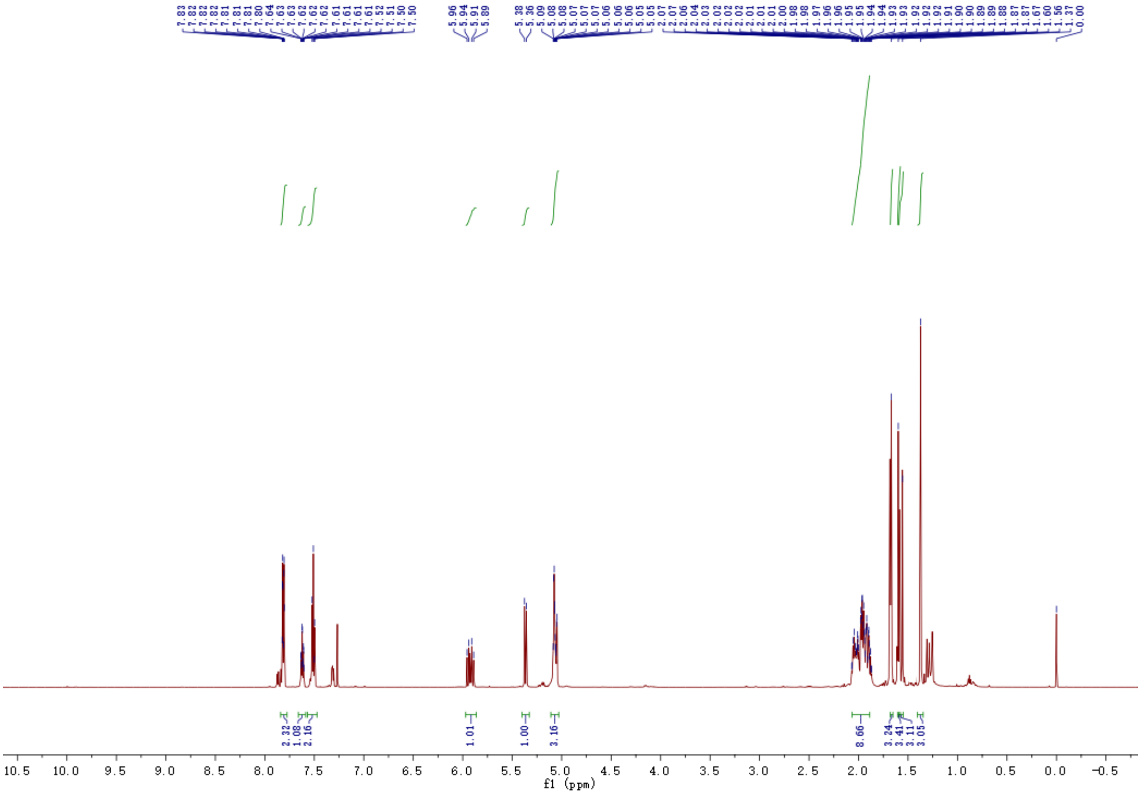

f1 (ppm)

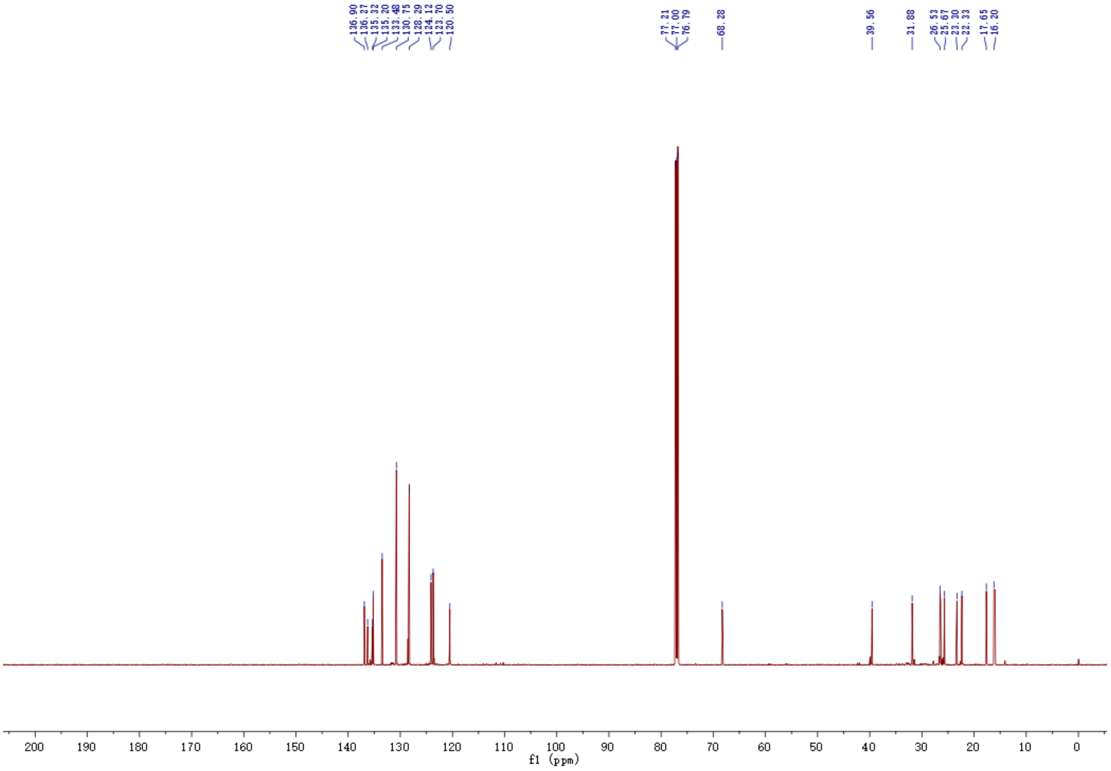

136.90
136.27
135.32
135.20
133.48
130.75
128.29
124.12
123.70
120.50
77.21
77.00
76.79
68.28
39.56
31.88
26.53
25.67
23.30
22.33
17.65
16.20
f1 (ppm)

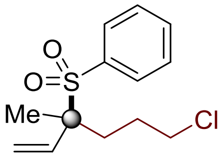

3ia

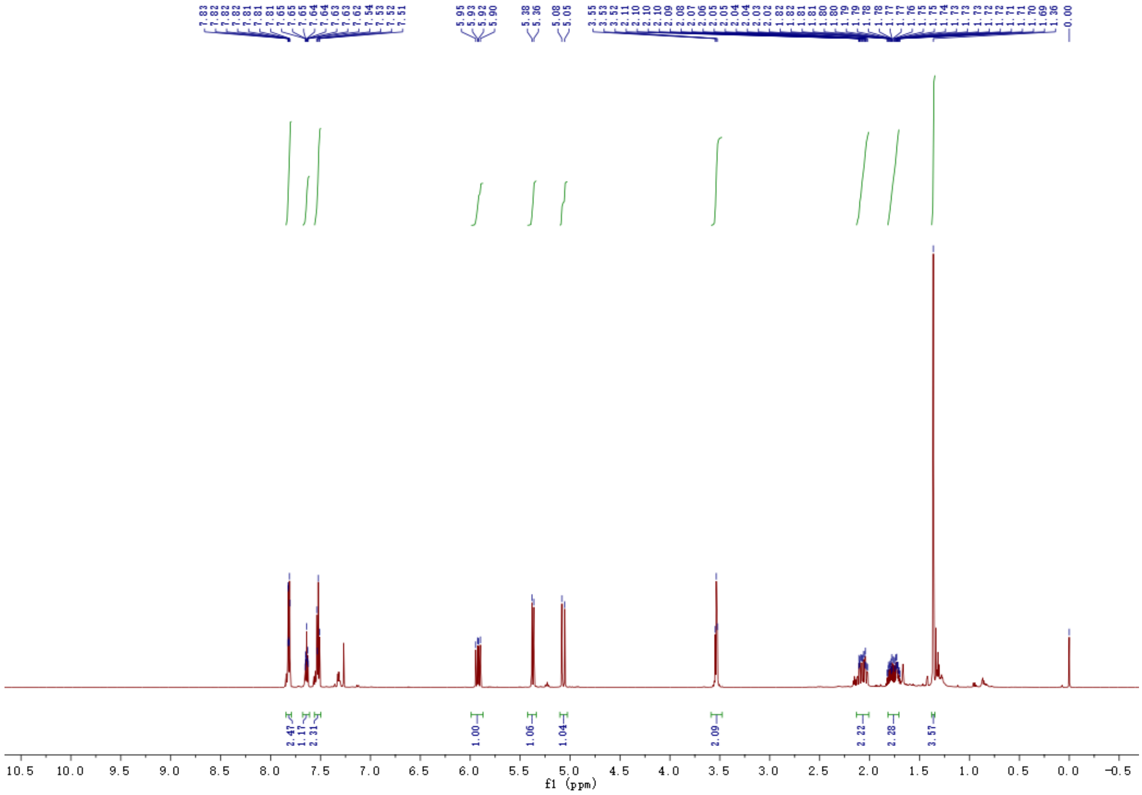

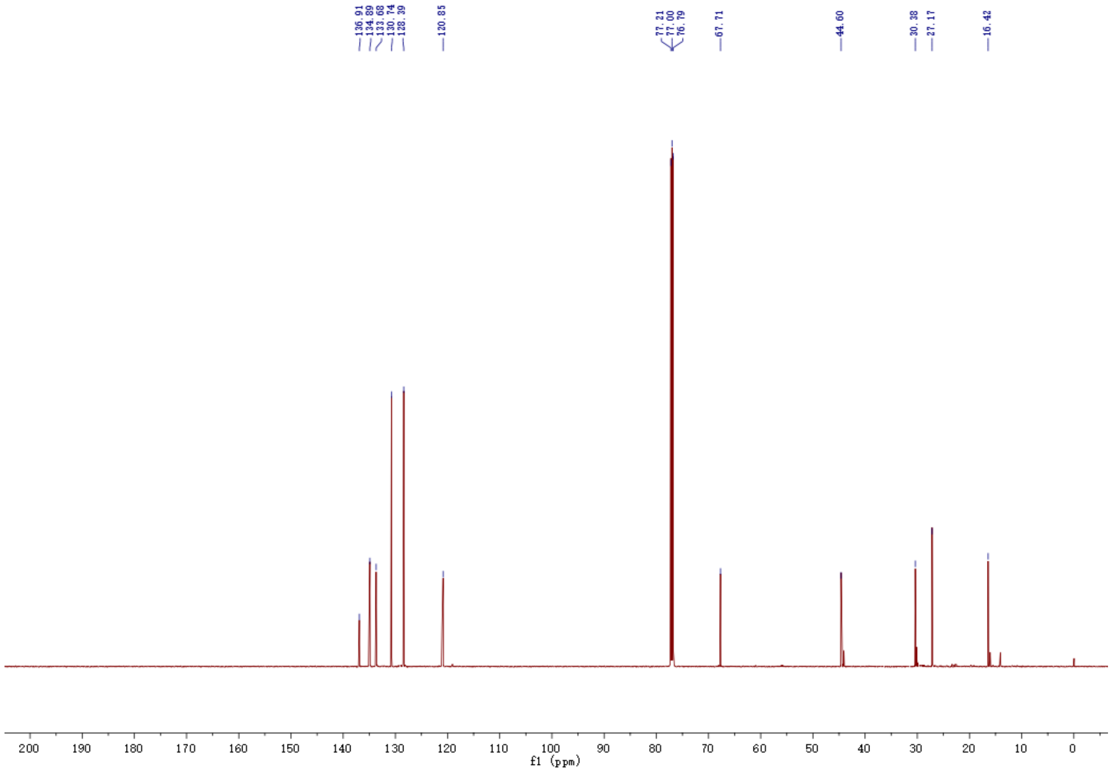

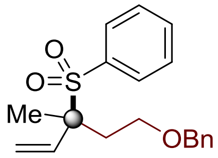
O
O=S
Me
OBn

3ja

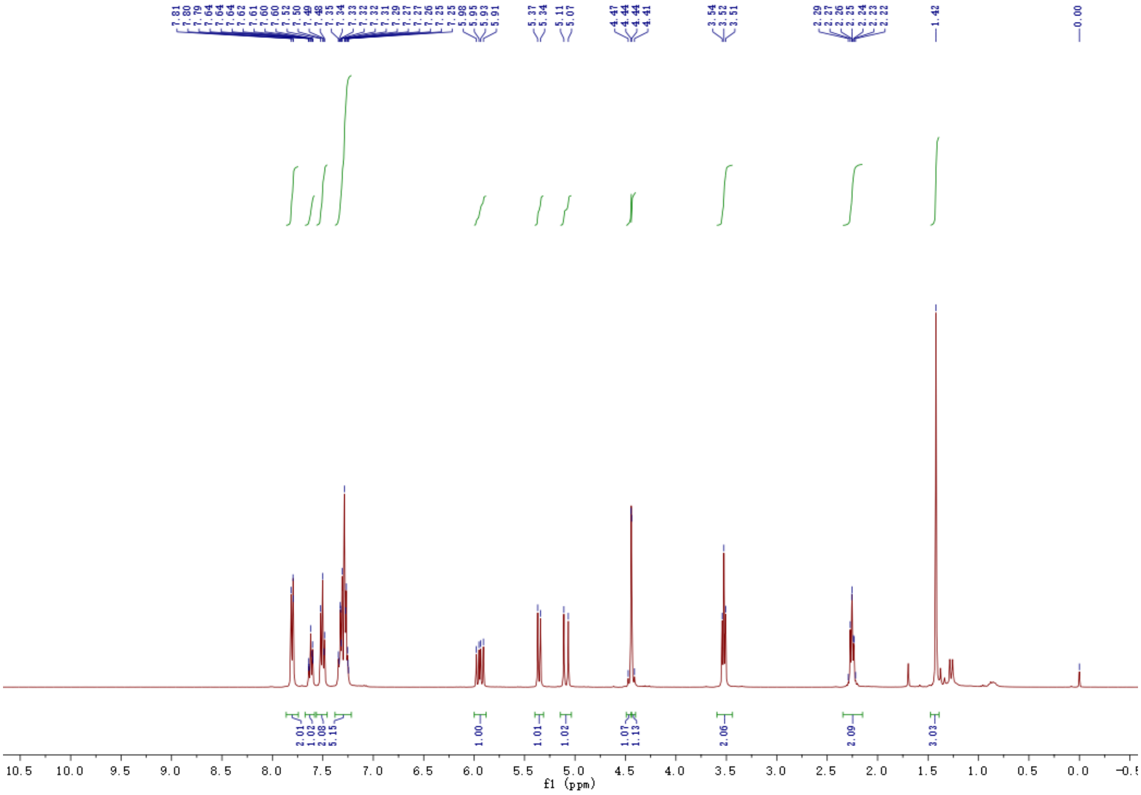
7.81
7.80
7.79
7.64
7.64
7.62
7.61
7.60
7.60
7.52
7.50
7.49
7.48
7.35
7.34
7.33
7.32
7.31
7.29
7.27
7.27
7.26
7.25
7.25
5.98
5.95
5.93
5.91
5.37
5.34
5.11
5.07
4.47
4.44
4.44
4.41
3.54
3.52
3.51
2.29
2.27
2.26
2.25
2.24
2.23
2.22
1.42
0.00
2.01
1.02
2.08
5.15
1.00
1.01
1.02
1.07
1.13
2.06
2.09
3.03
f1 (ppm)

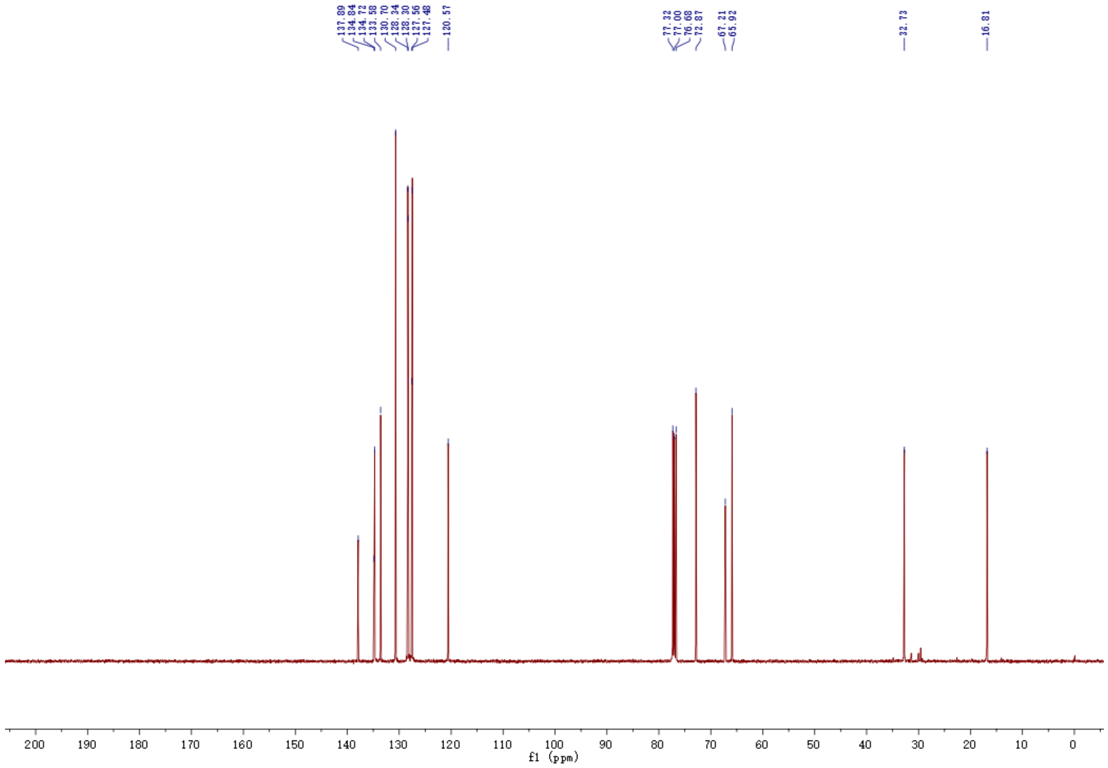
137.89
134.84
134.72
133.58
130.70
128.34
128.30
127.56
127.48
120.57
77.32
77.00
76.68
72.87
67.21
65.92
32.73
16.81
f1 (ppm)

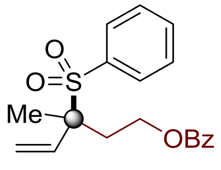

3ka

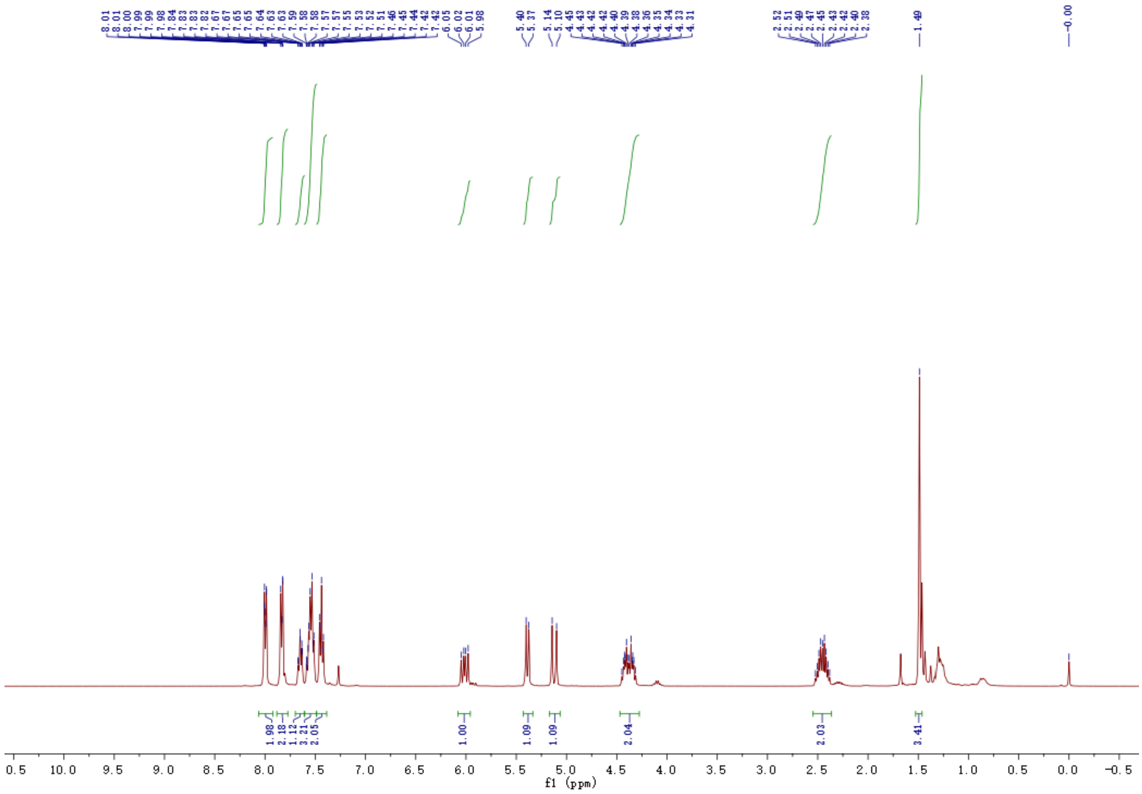

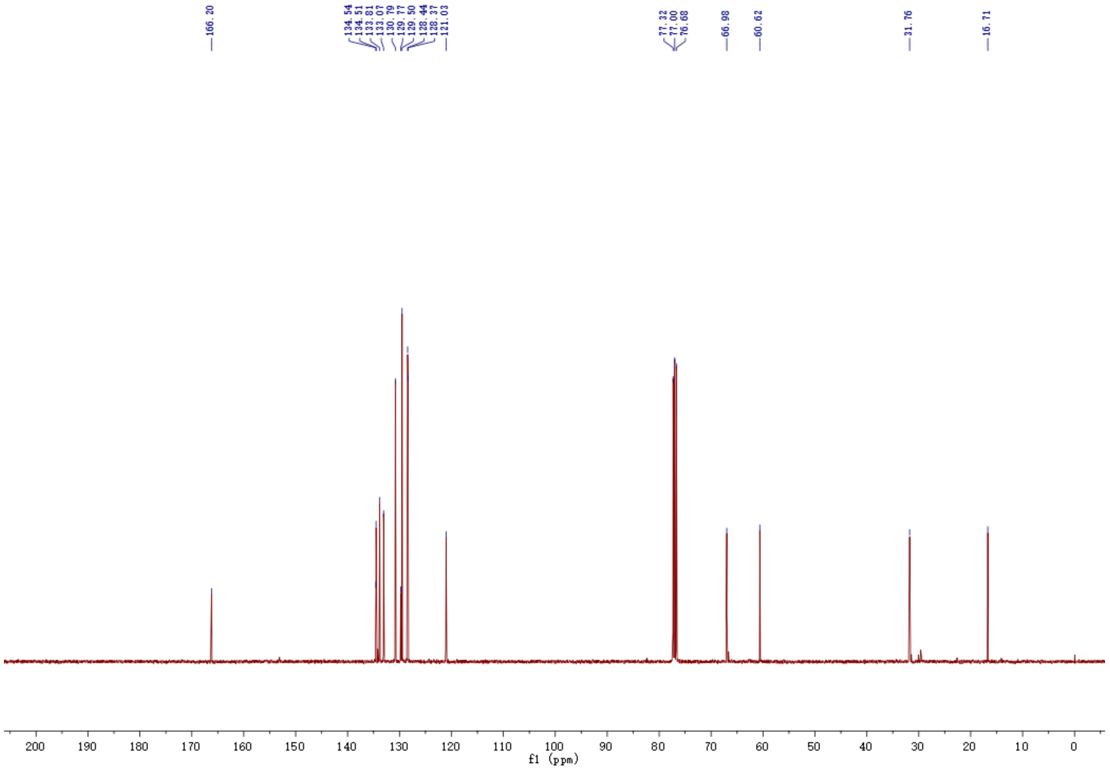

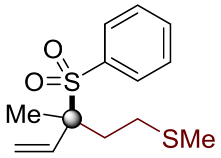

3la

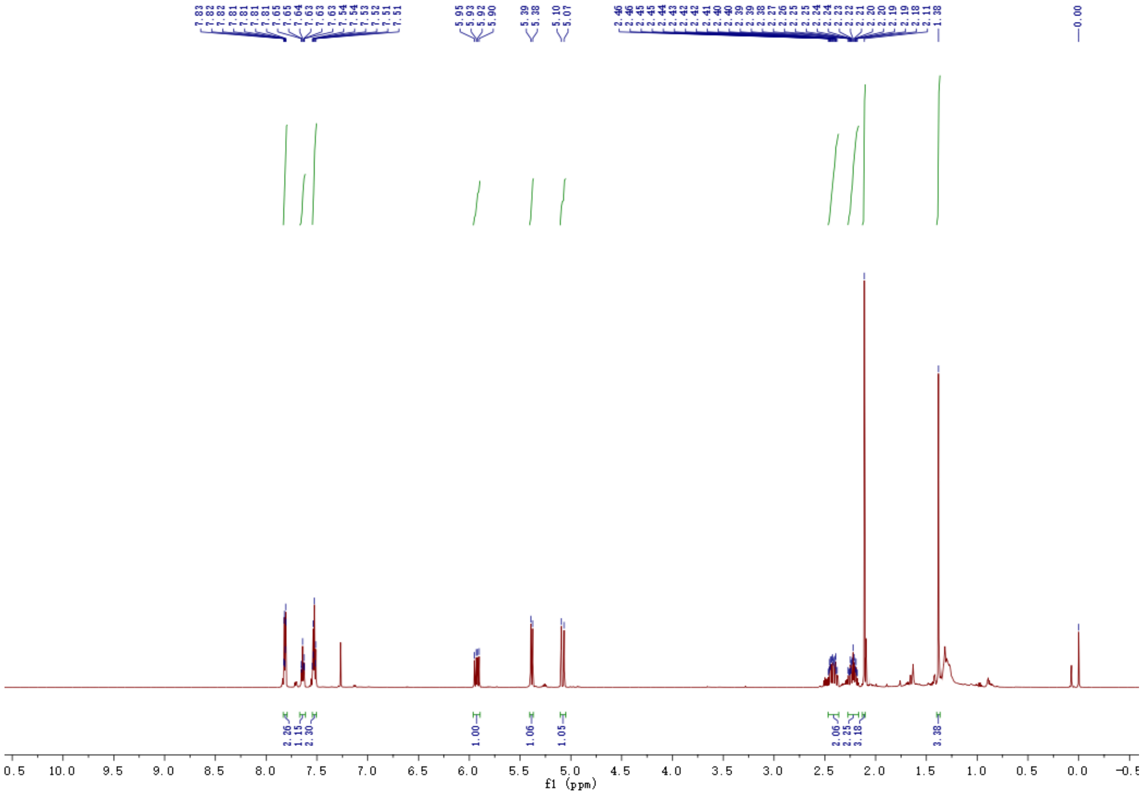

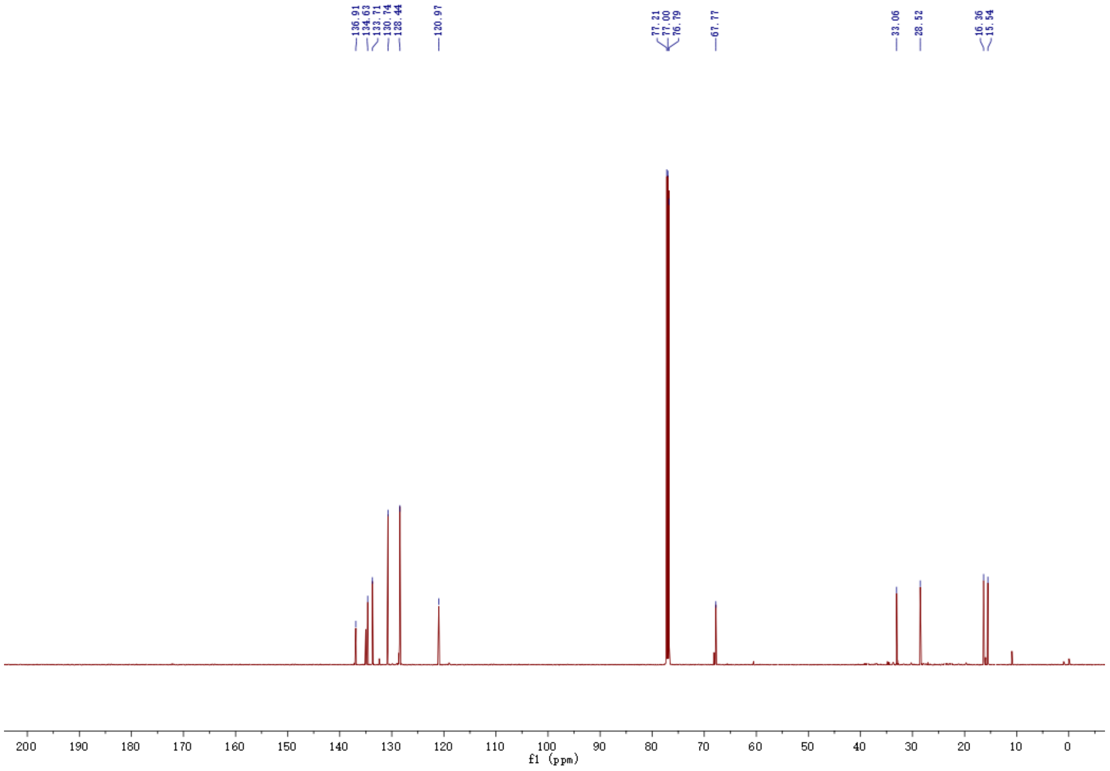

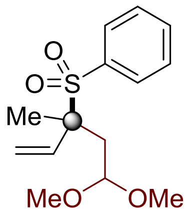

3ma

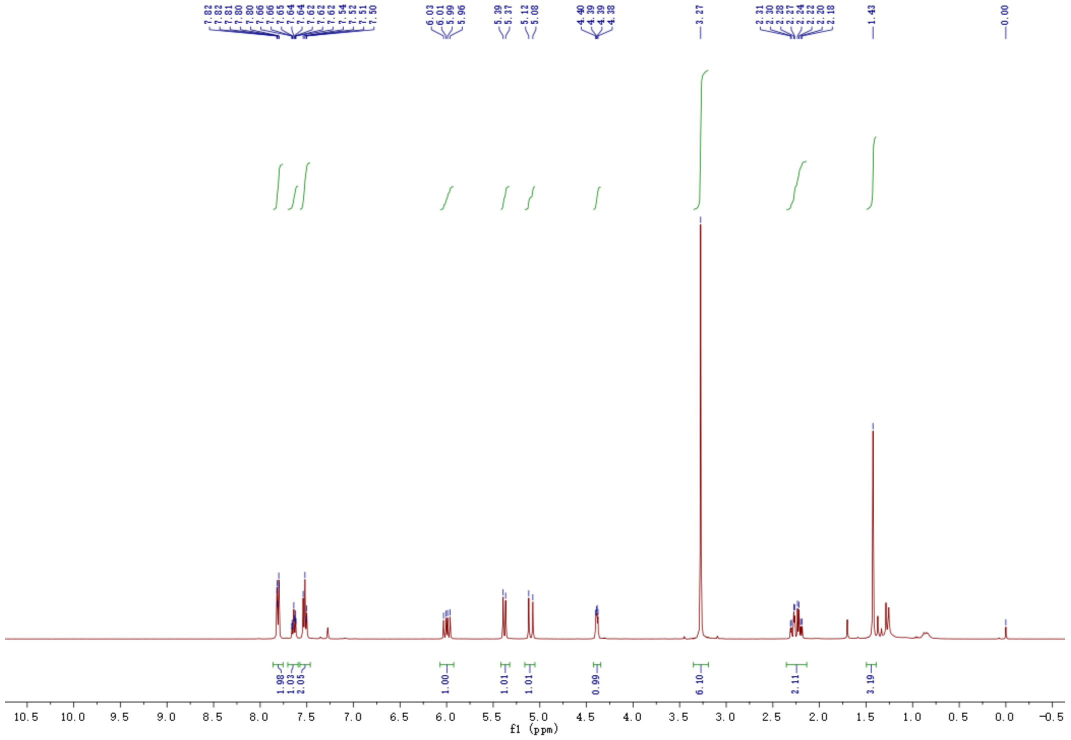

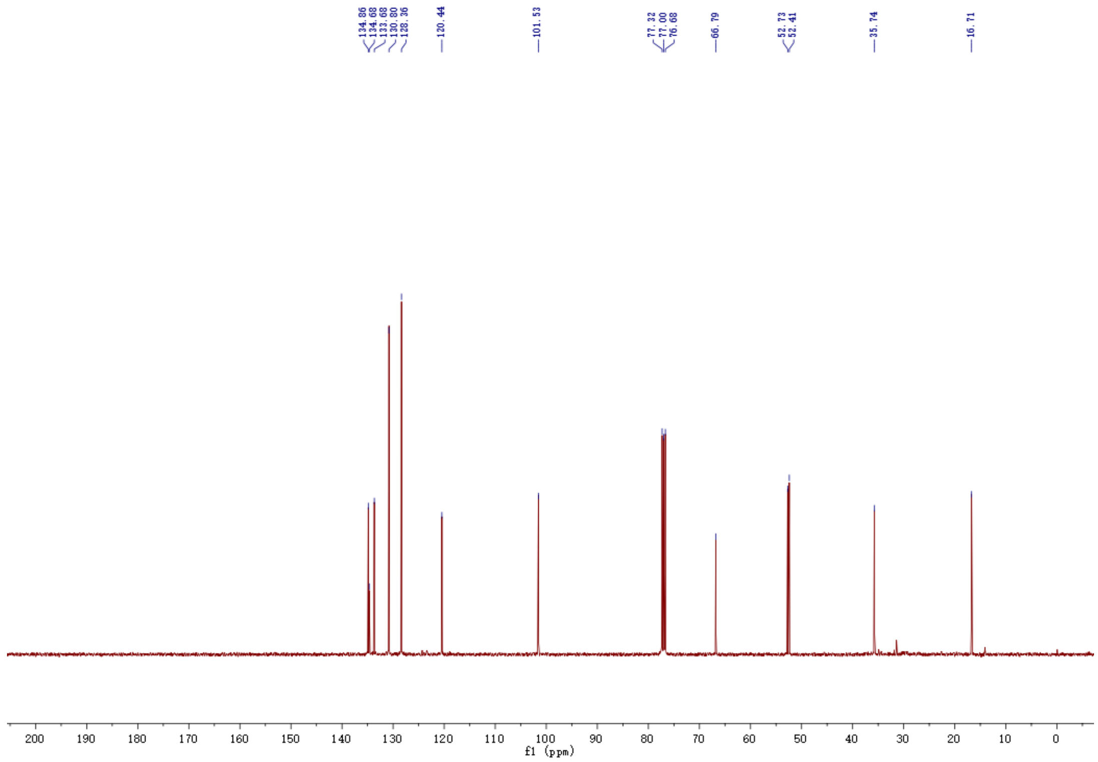

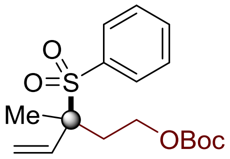

3na

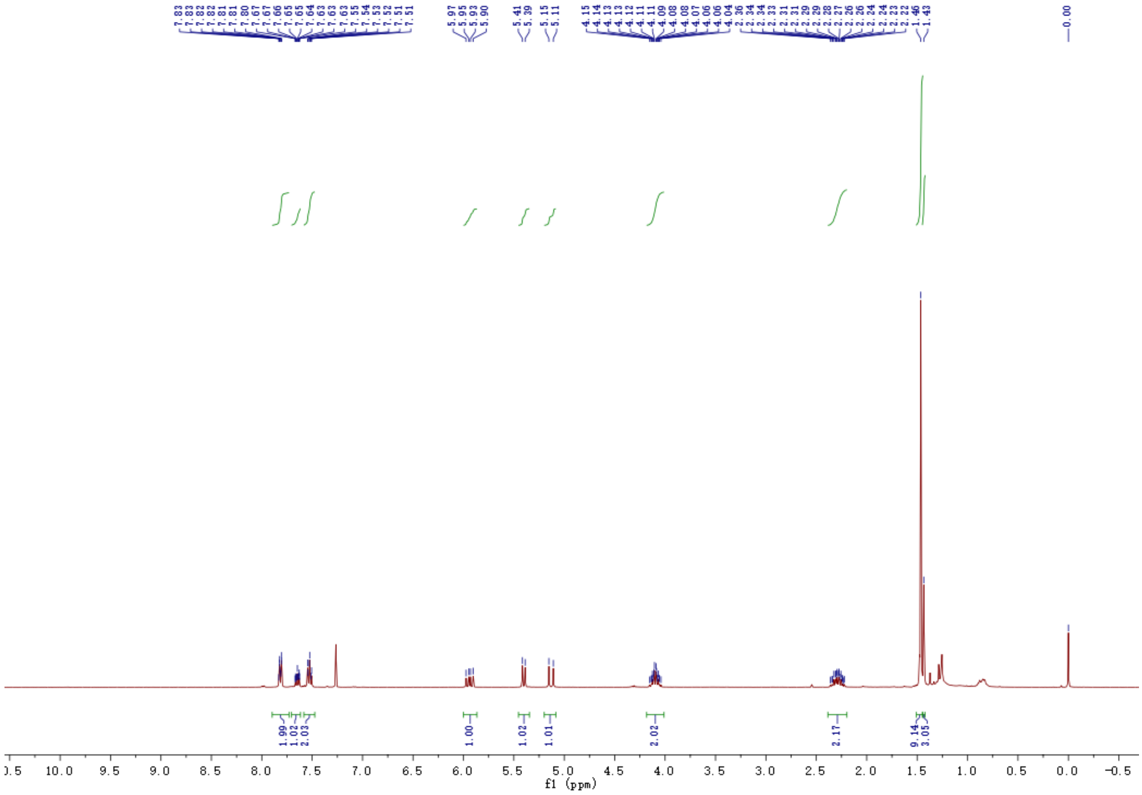

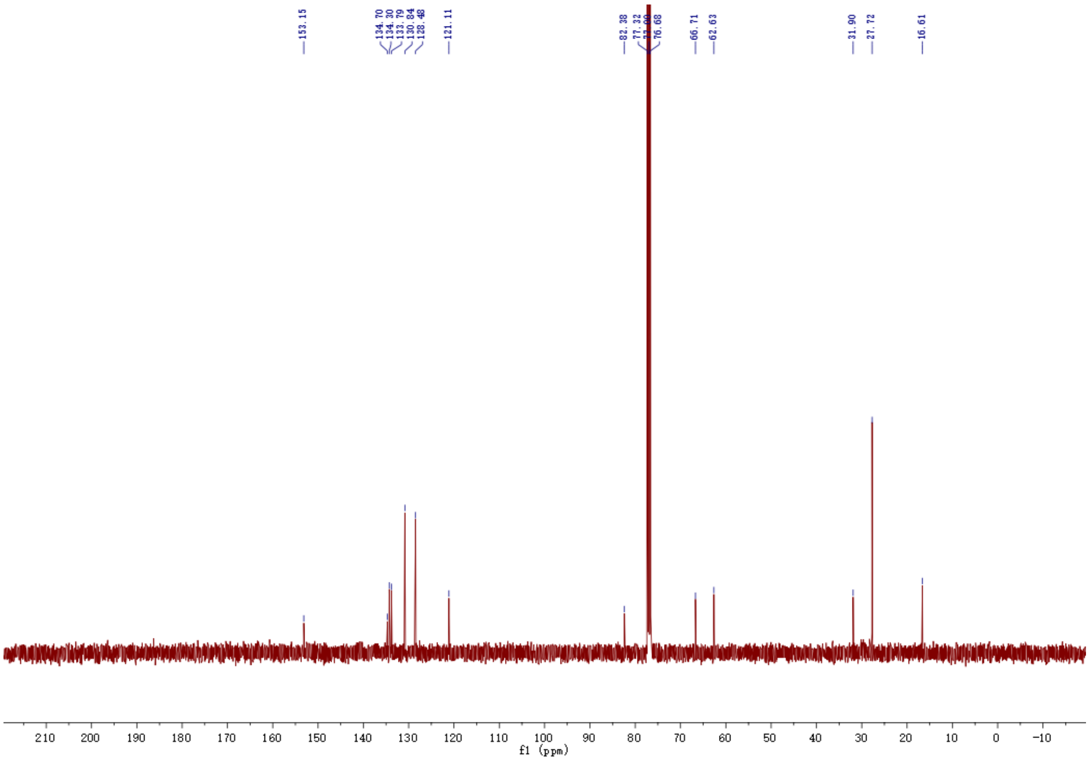

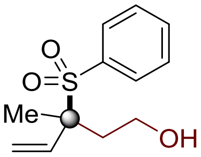

3oa

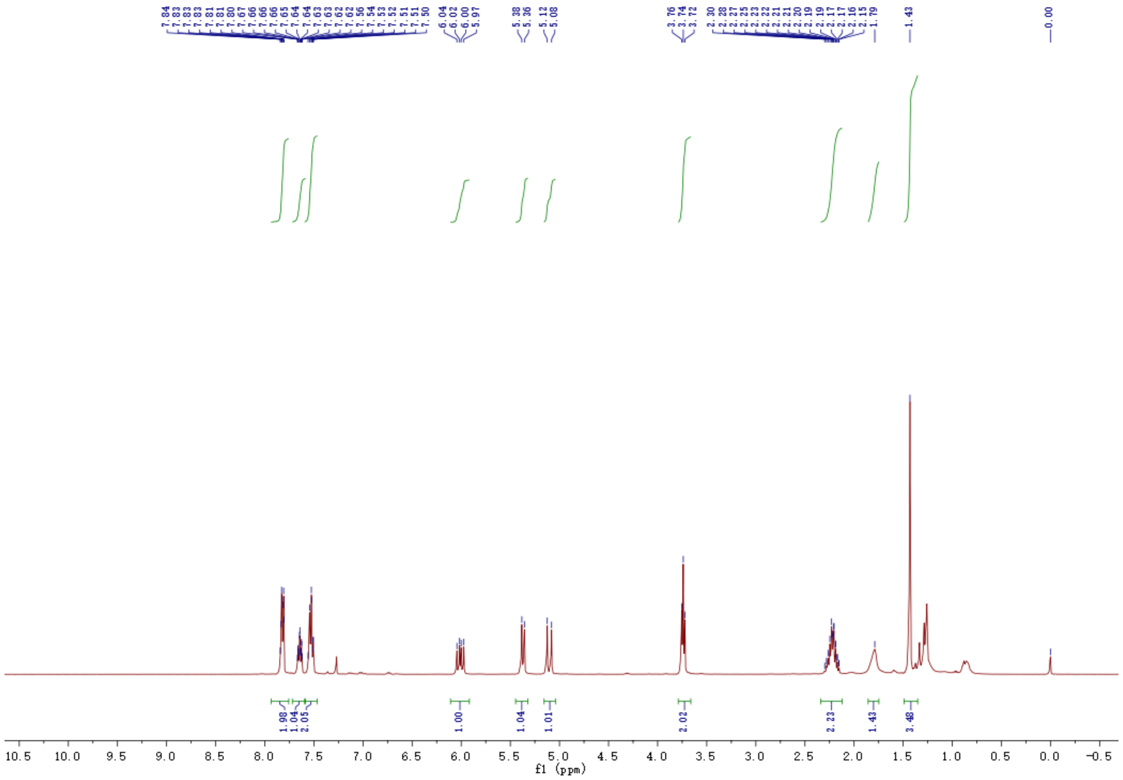

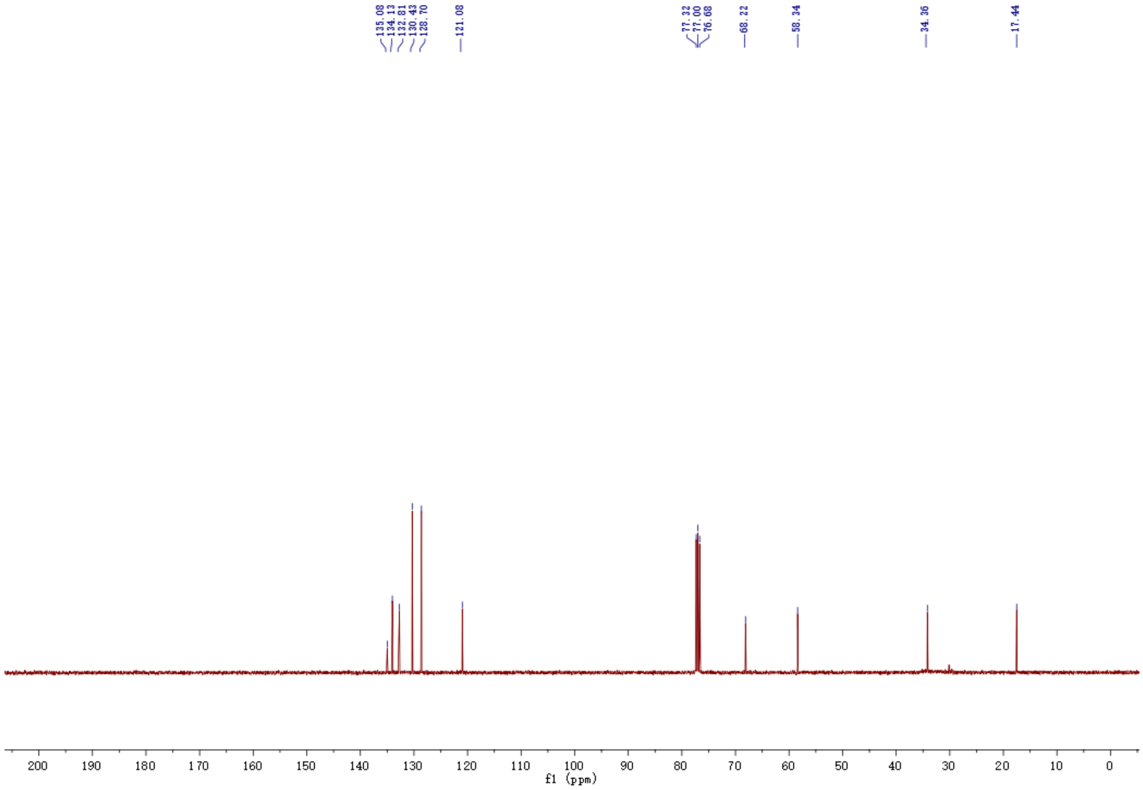

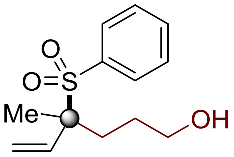

3pa

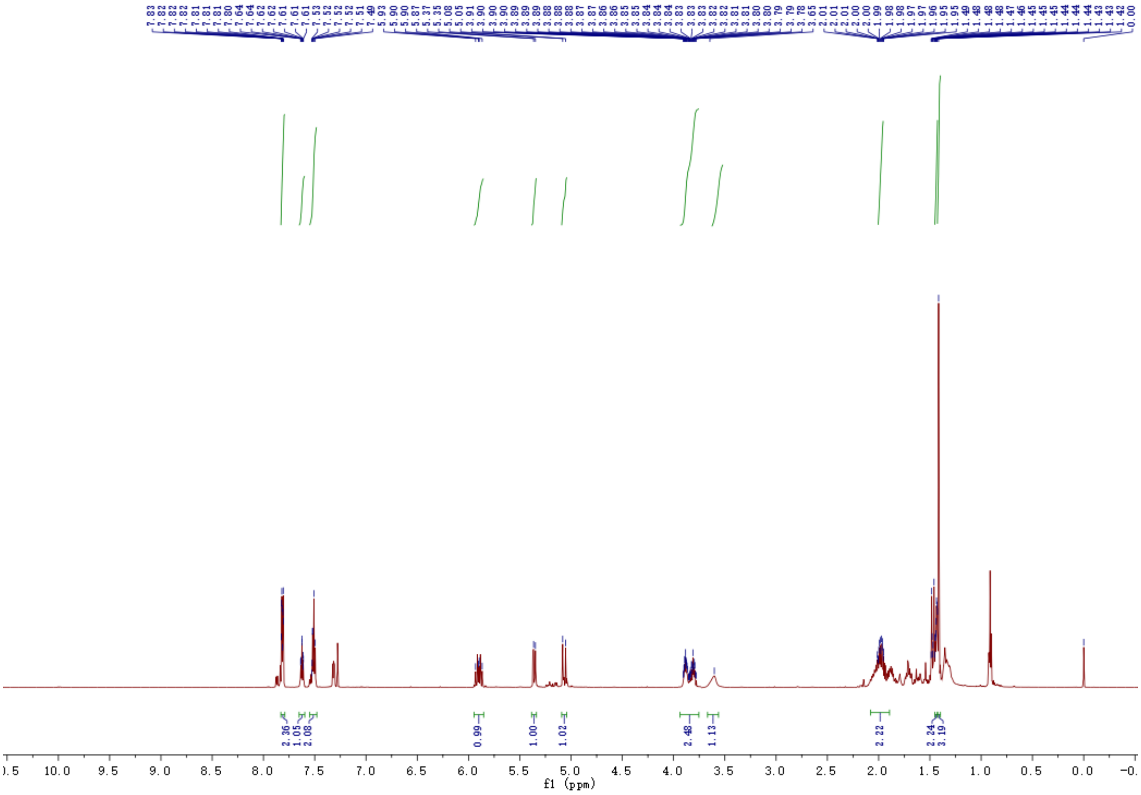

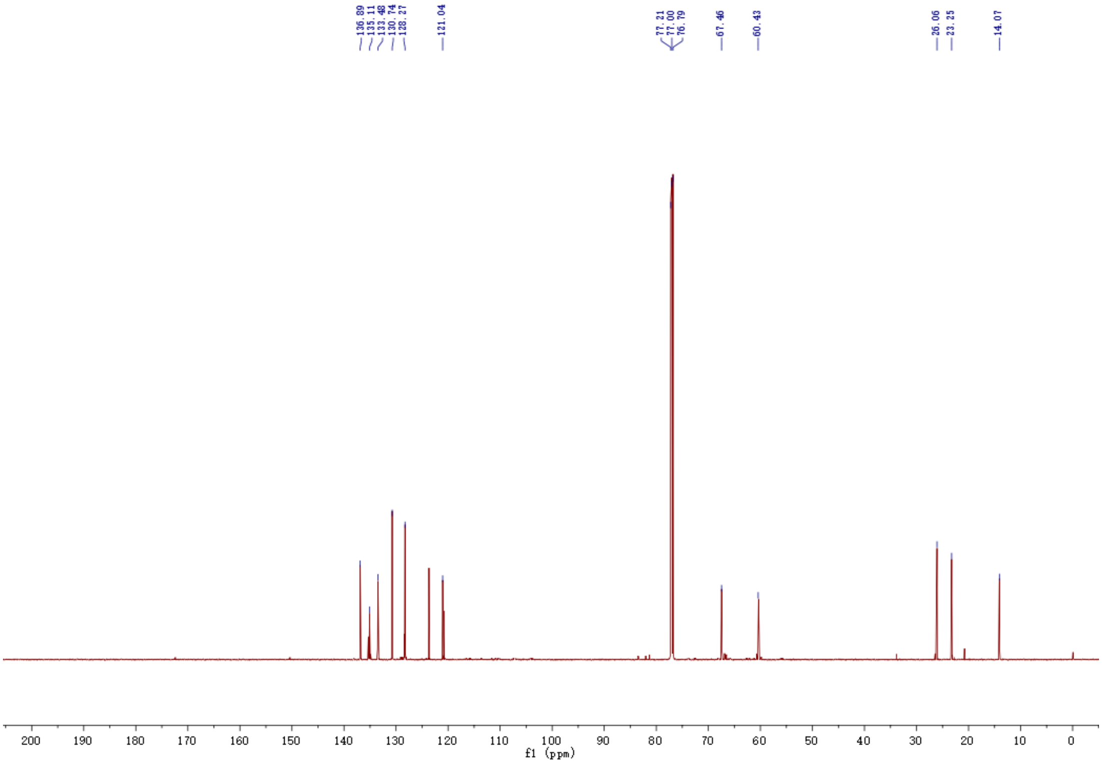

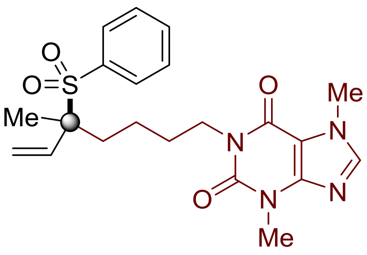

3qa

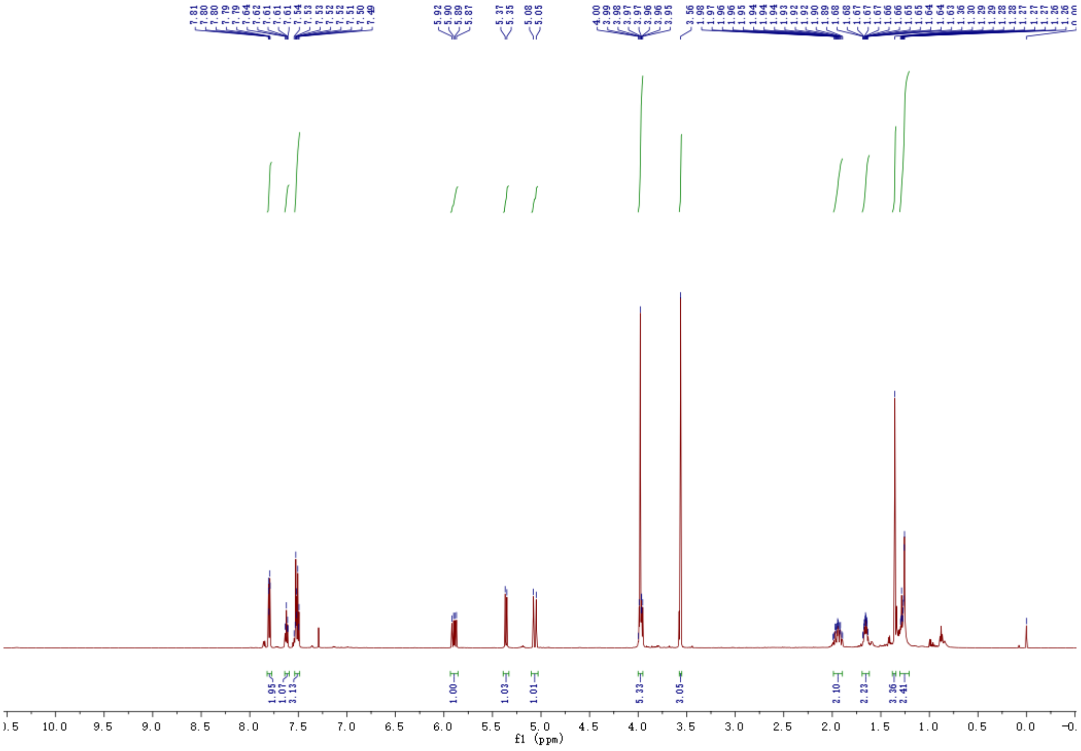

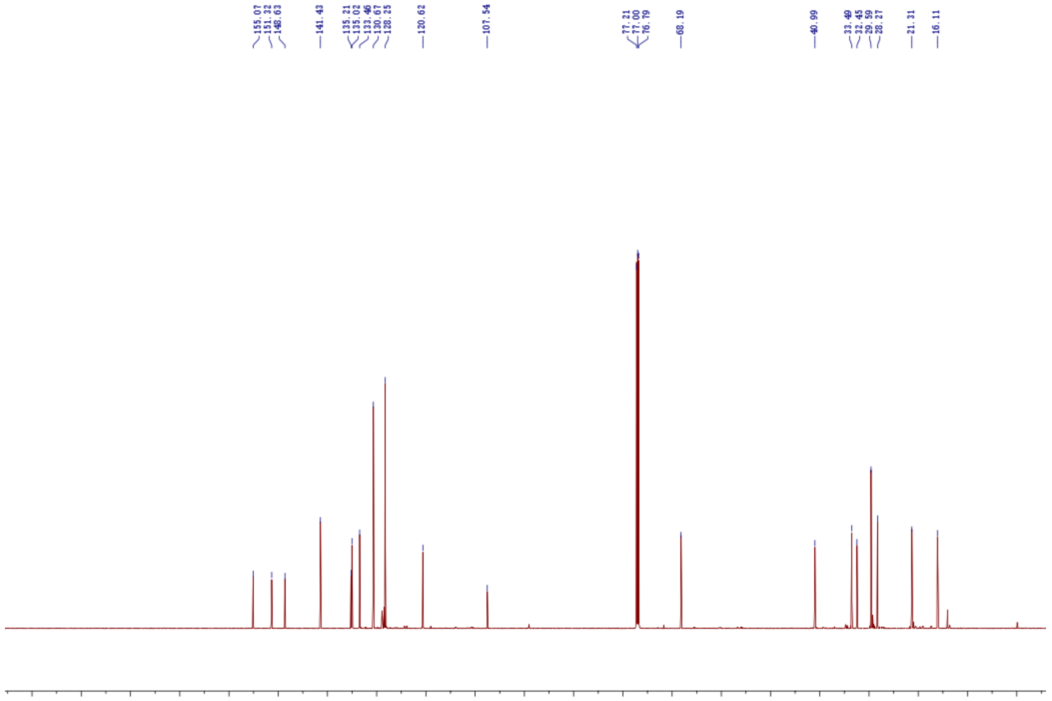

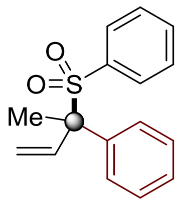
O
O=S
Me

3ra

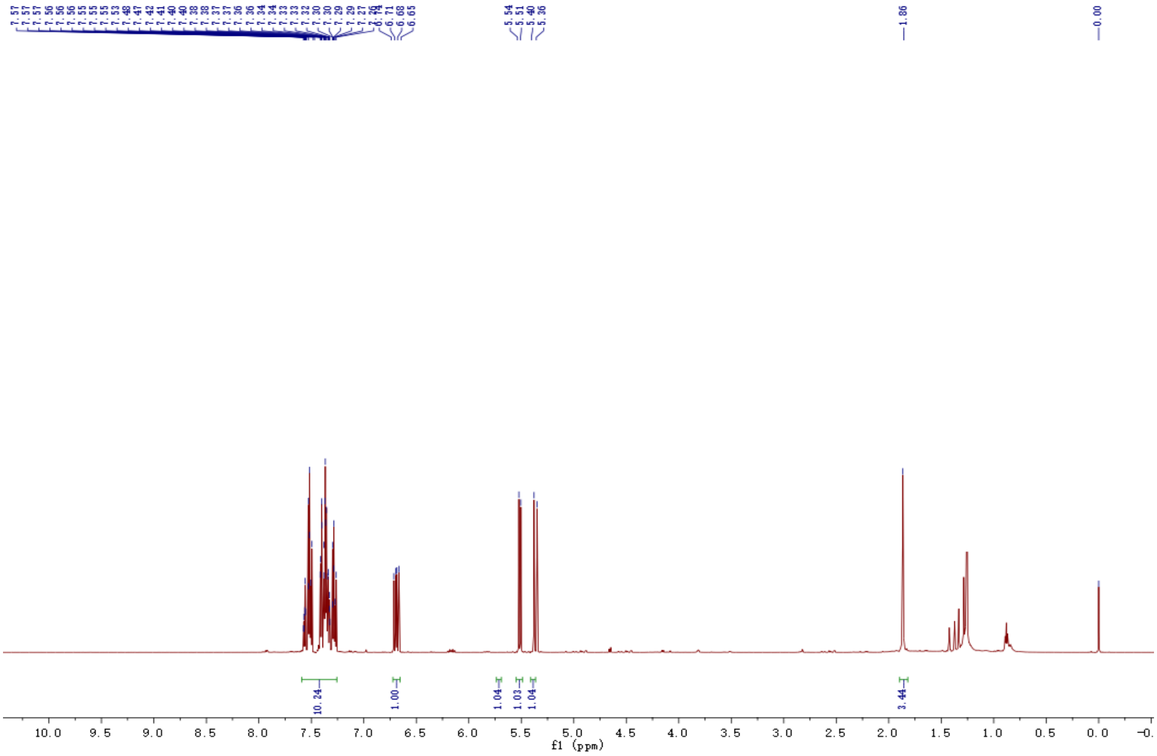
6.71
6.68
6.65
5.54
5.51
5.40
5.36
1.86
0.00
10.24
1.00
1.04
1.03
1.04
3.44
f1 (ppm)

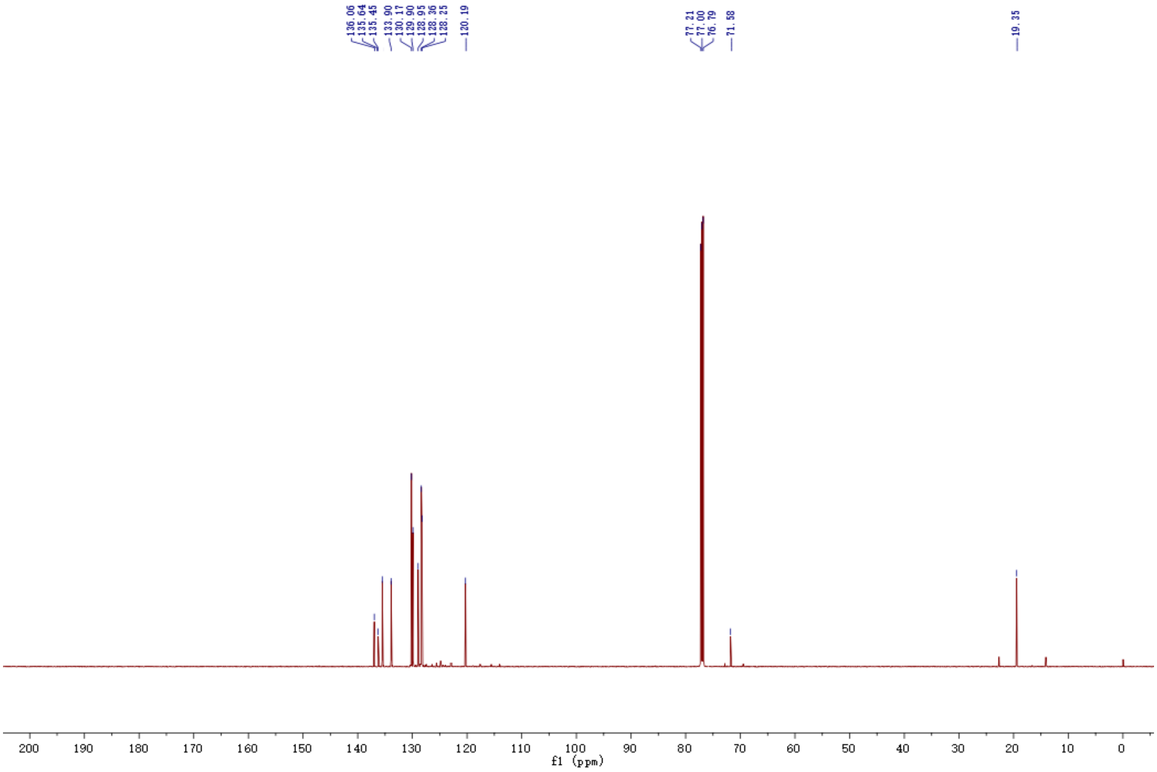
136.06
135.64
135.45
133.90
130.17
129.90
128.95
128.36
128.25
120.19
77.21
77.00
76.79
71.58
19.35
f1 (ppm)

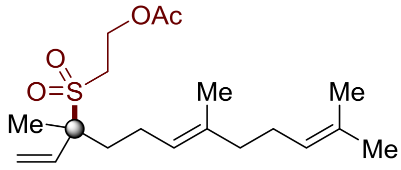
OAc
O
O=S
Me
Me
Me
Me

3haz

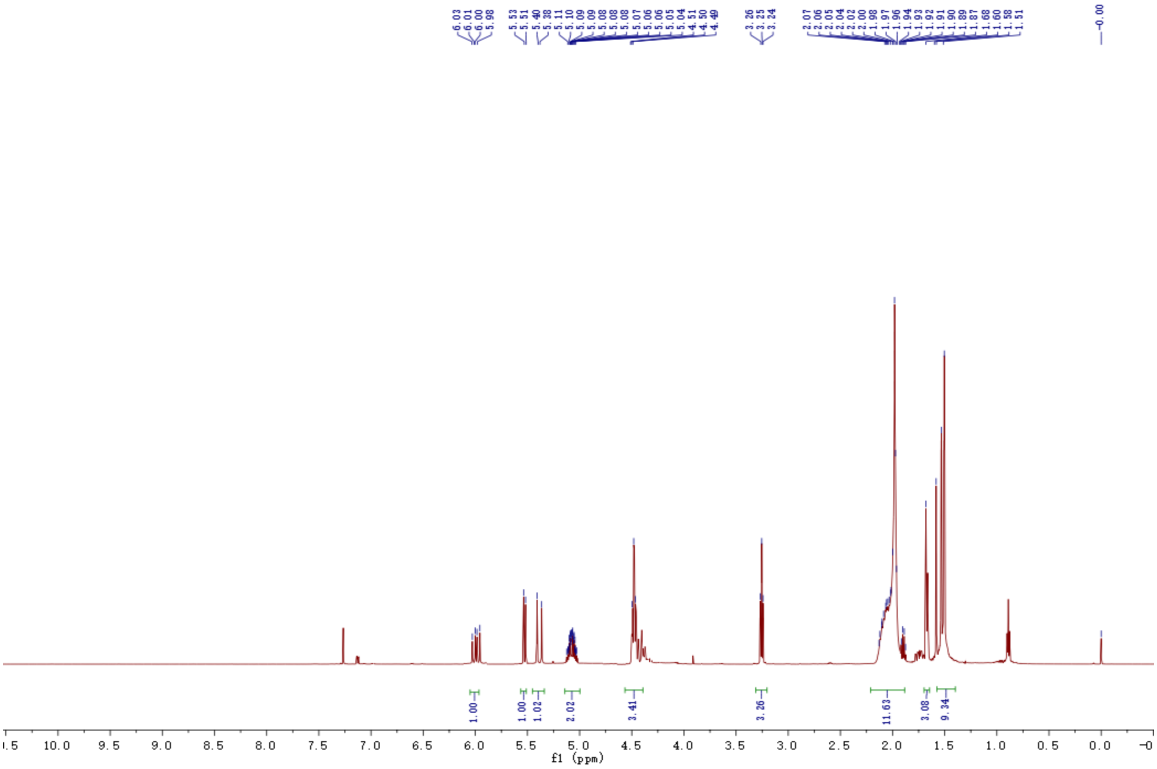
6.03 6.01 6.00 5.98 5.53 5.51 5.40 5.38 5.11 5.10 5.09 5.09 5.08 5.08 5.08 5.07 5.06 5.05 5.04 4.51 4.50 4.49 3.26 3.25 3.24 2.07 2.06 2.05 2.04 2.02 2.00 1.98 1.97 1.96 1.94 1.93 1.92 1.91 1.90 1.89 1.87 1.68 1.60 1.58 1.51 0.00
1.00 1.00 1.02 2.02 3.41 3.26 11.63 3.08 9.34
10.0 9.5 9.0 8.5 8.0 7.5 7.0 6.5 6.0 5.5 5.0 4.5 4.0 3.5 3.0 2.5 2.0 1.5 1.0 0.5 0.0
f1 (ppm)

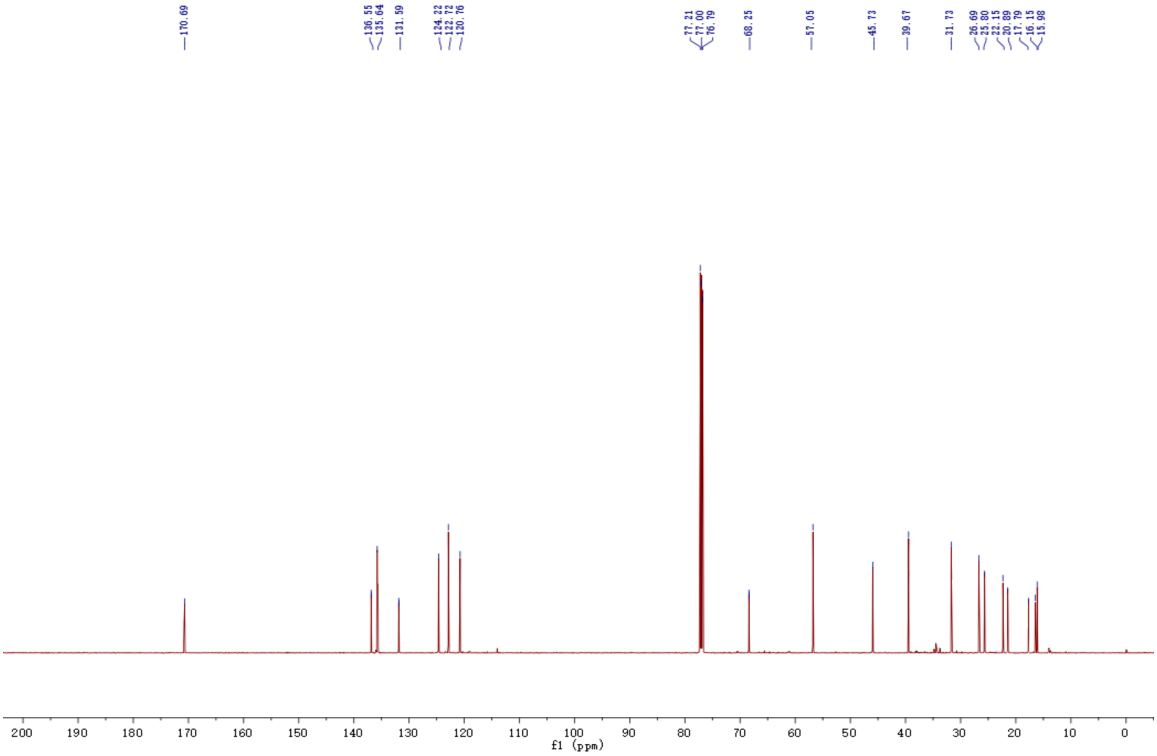
170.69 136.55 135.64 131.59 124.22 122.72 120.76 77.21 77.00 76.79 68.25 57.05 45.73 39.67 31.73 26.69 25.80 22.15 20.89 17.79 16.15 15.98
200 190 180 170 160 150 140 130 120 110 100 90 80 70 60 50 40 30 20 10 0
f1 (ppm)

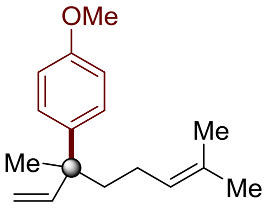

4ga

(±)-**sporochnol-methyl ether**

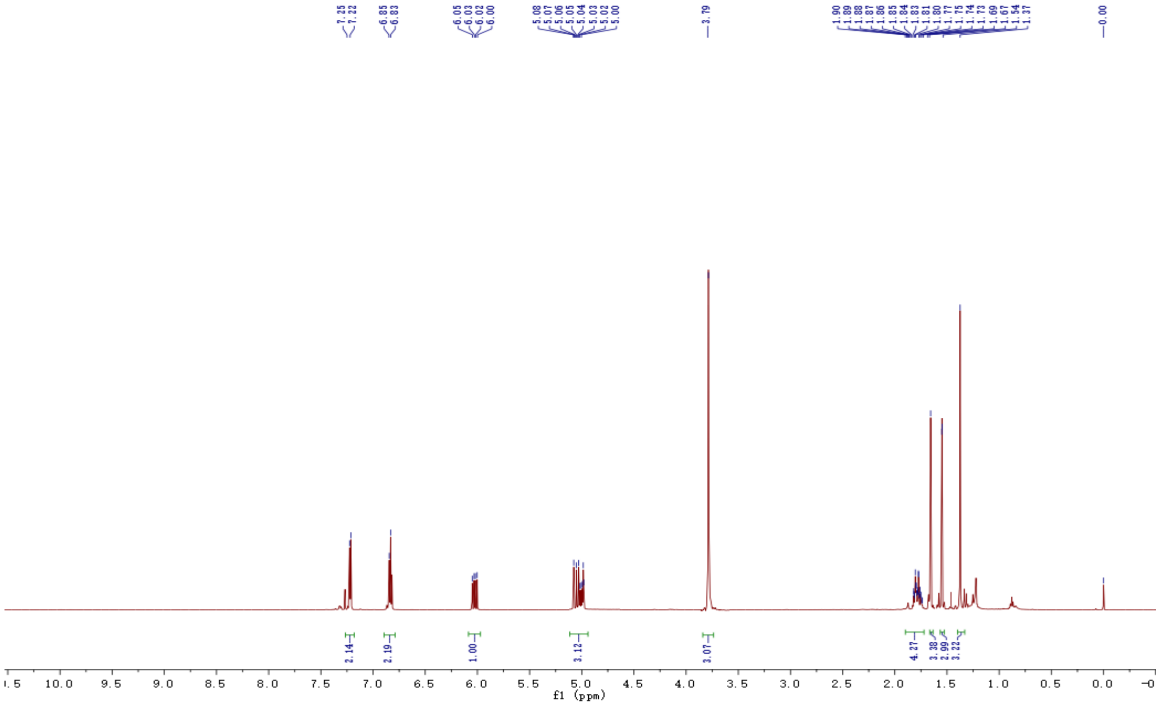

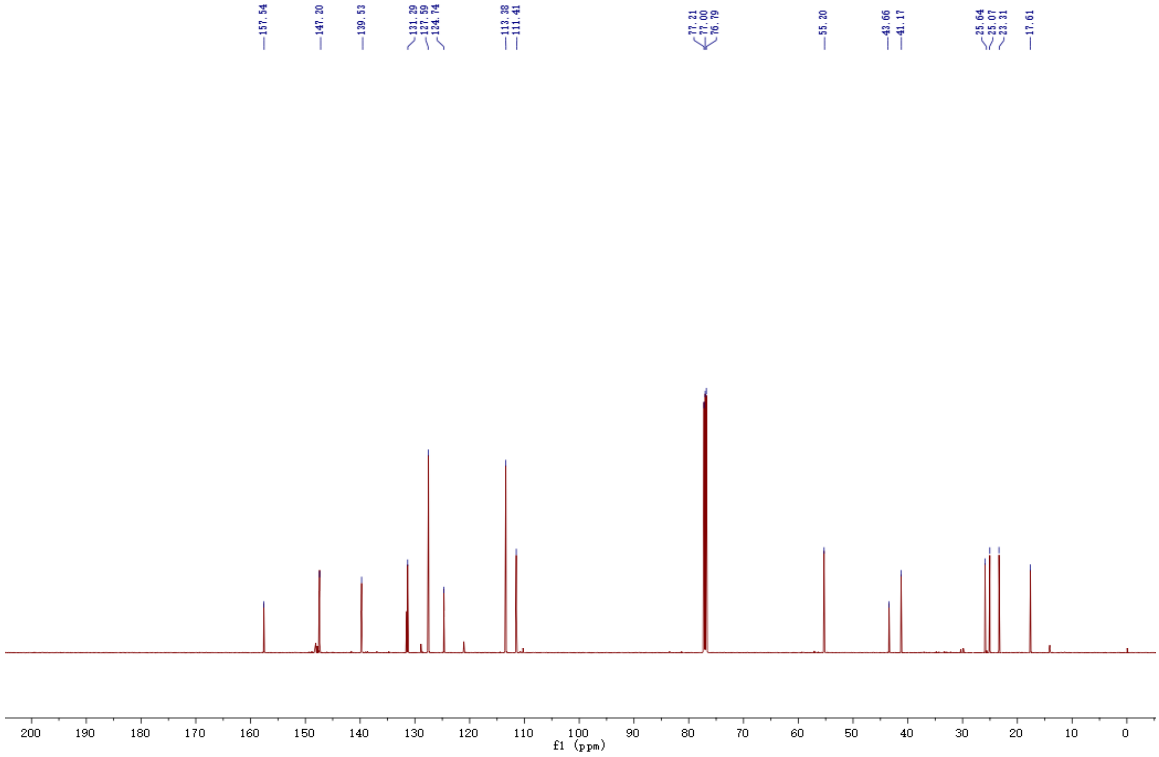

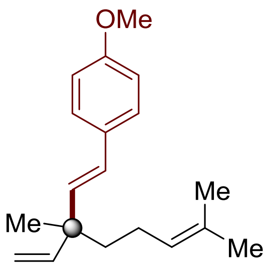

4gb

(±)-**bakuchiol-methyl ether**

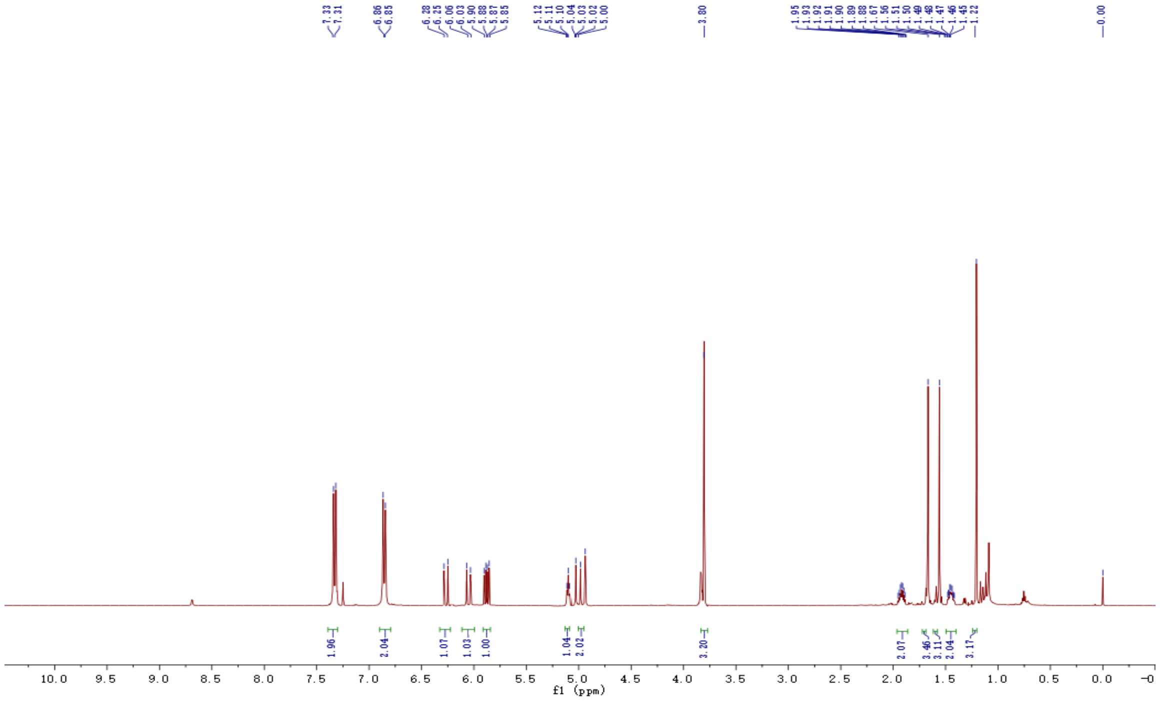

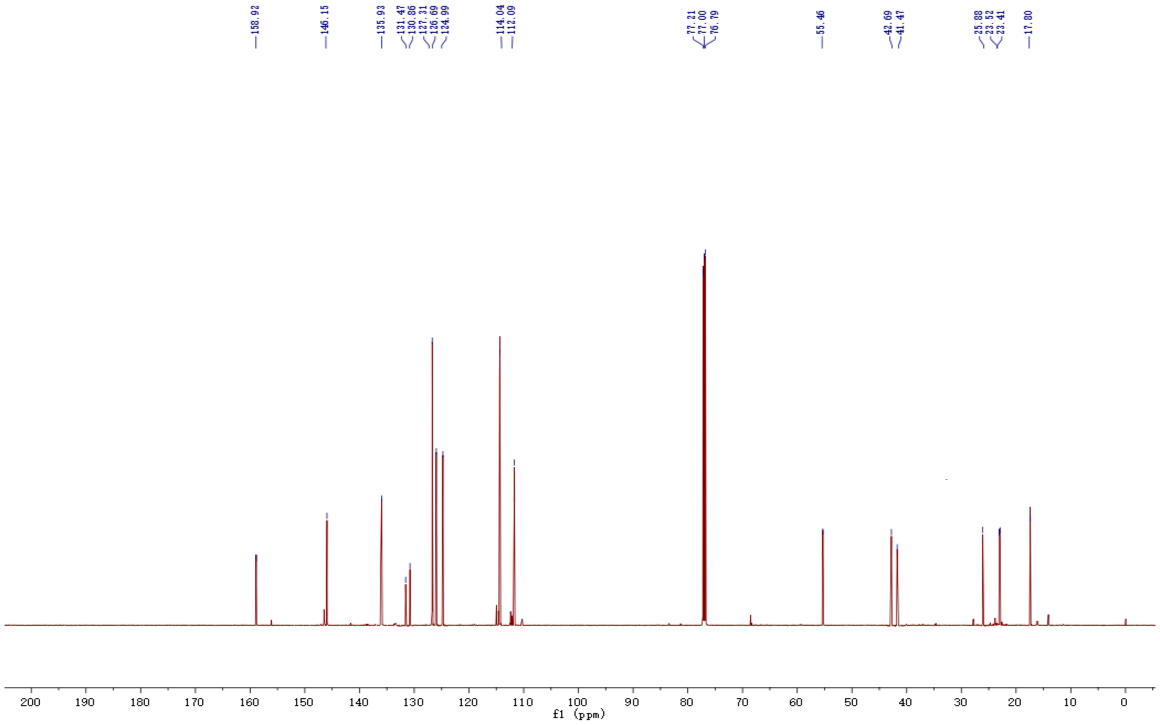

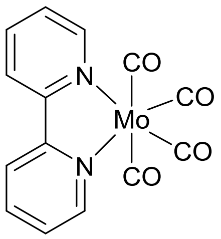

Mo(bpy)(CO)$_4$

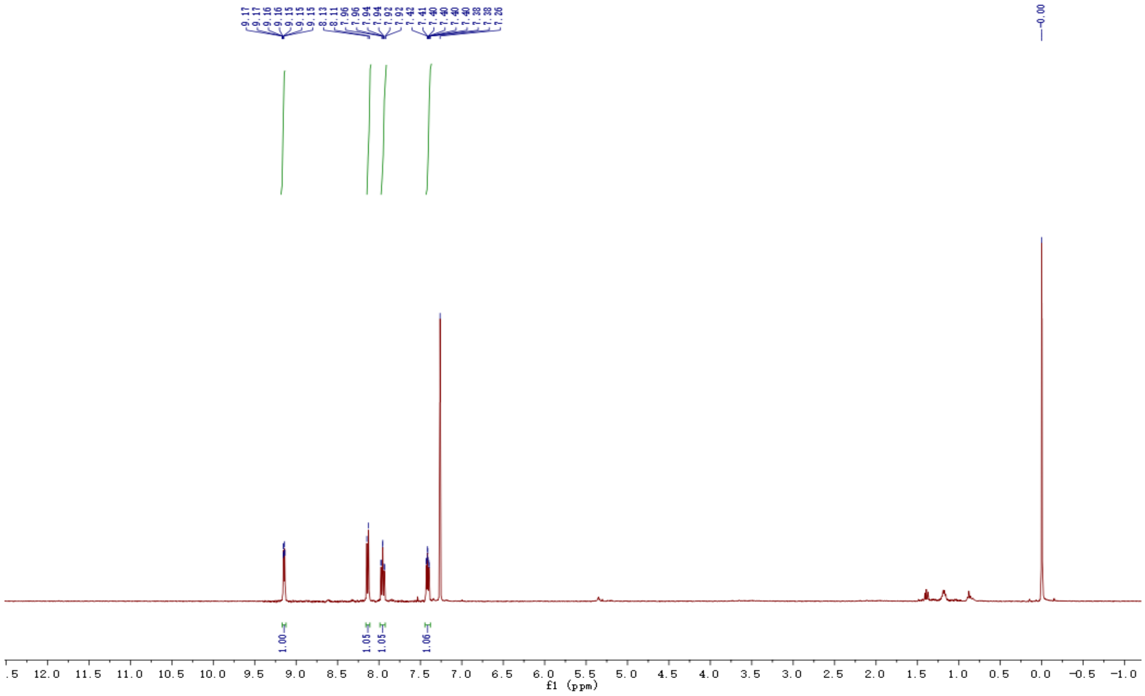

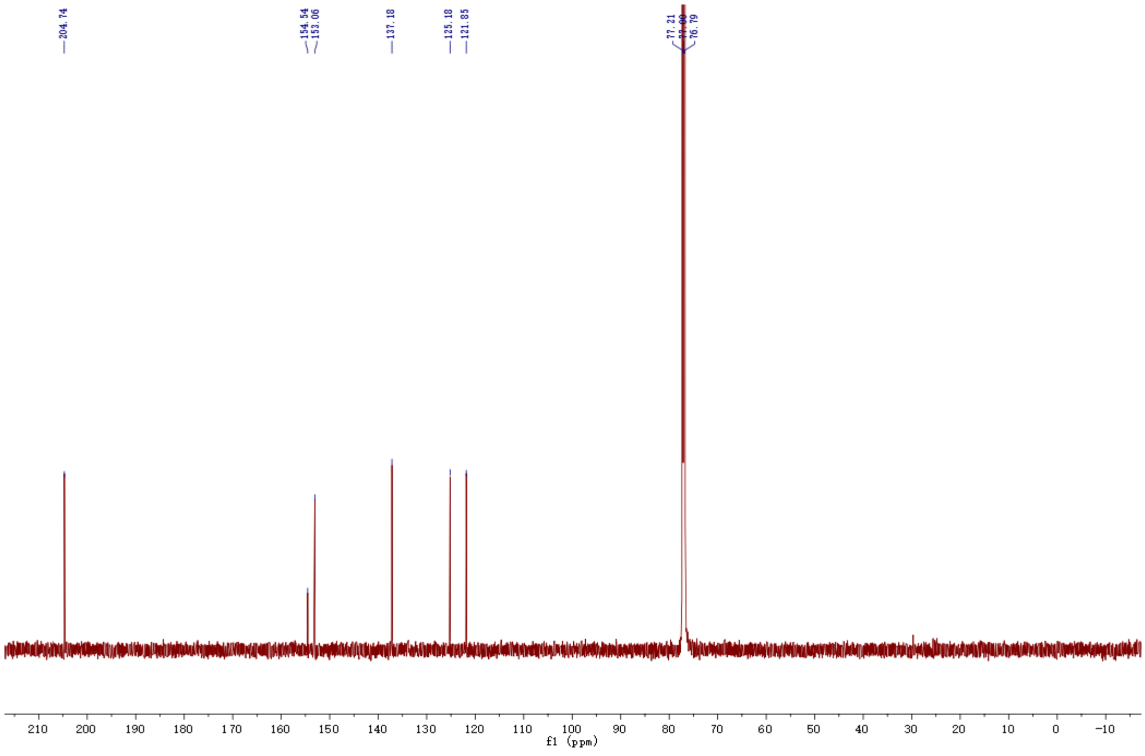

Supplement: SC-011-D0SC01763A-s002 [file SC-011-D0SC01763A-s002.pdf]
